# On Raman optical activity sign-switching between the ground and excited states leading to an unusual resonance ROA induced chirality†

**DOI:** 10.1039/d0sc05345g

**Published:** 2020-11-02

**Authors:** Ewa Machalska, Grzegorz Zajac, Malgorzata Baranska, Dorota Kaczorek, Robert Kawęcki, Piotr F. J. Lipiński, Joanna E. Rode, Jan Cz. Dobrowolski

**Affiliations:** Faculty of Chemistry, Jagiellonian University Gronostajowa 2 30-387 Cracow Poland m.baranska@uj.edu.pl; Jagiellonian Centre for Experimental Therapeutics (JCET), Jagiellonian University Bobrzynskiego 14 30-348 Cracow Poland; Siedlce University, Faculty of Science 3 Maja Street No 54 08-110 Siedlce Poland robert.kawecki@uph.edu.pl; Department of Neuropeptides, Mossakowski Medical Research Centre, Polish Academy of Sciences Pawińskiego 5 02-106 Warsaw Poland; Institute of Nuclear Chemistry and Technology 16 Dorodna-Street 03-195 Warsaw Poland j.dobrowolski@nil.gov.pl

## Abstract

Raman optical activity (ROA) spectra recorded for a chiral naphthalene diimide derivative (nBu-NDI–BINAM) dissolved in a series of solvents exhibit strong solute to solvent induced chirality with: (1) dominating bands of solvents, (2) *n*Bu-NDI–BINAM resonance ROA (RROA) bands which are barely visible, (3) monosignate RROA Solvent spectra with an unexpected sign concordant with that of the ECD band of the resonant electronic state, (4) bisignate RROA bands for a few solvents, and (5) superposition of non-resonant and resonant ROA bands of the chiral solvents. The unusual ROA enhancement was explained in terms of resonance energy transfer with resonant Raman emission. The surprising RROA sign-switching was found to be due to specific conformational equilibria where one solute conformer dominates in the ground and the other in the first excited singlet state, and, the signs of the related ECD bands of these two conformers are opposite.

## Abstract 2

Unusual solute to solvent induced chirality in ROA comes from specific conformer equilibria in the ground and the excited states.

## Graphical abstract

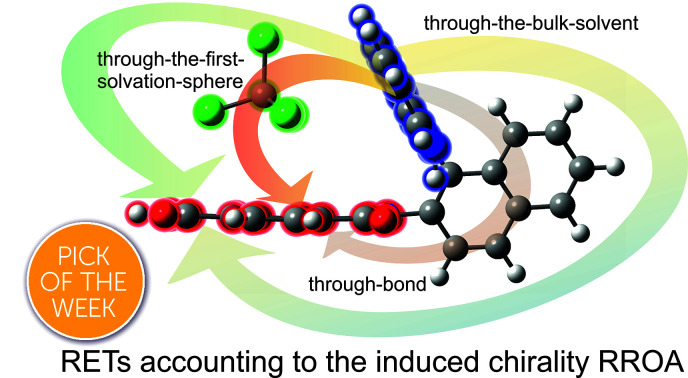

## Introduction

The role of chiral molecules in life sciences, chemistry and physics has steadily increased. Indeed, chirality accounts for structural and functional diversity of biological molecules, is essential for pharmacy and is important in materials sciences. The need for characterization of chiral substances motivated fast development of vibrational circular dichroism (VCD) and Raman optical activity (ROA)^1,2^ which have become complementary to much more sensitive electronic circular dichroism (ECD). This is due to the distinctiveness and specificity of weak but abundant vibrational bands contrasting with a low number of intense but broad electronic ones. Induced Chirality (IC, or chirality transfer, ChT) appears when in the presence of a chiral molecule an achiral one becomes active in chiroptical spectroscopy.^3,4^ In VCD, the effect has been known since the early 2000s,^5,6^ while in ROA, it was only rarely reported.^7–9^ In 2016, Šebestík *et al.* interpreted strong ROA chirality induced from chiral HQ helicene dye to achiral solvents^7^ in terms of transfer of energy from HQ to proximate solvent molecules sharing the HQ electronic space. Quite recently Li *et al.* interpreted strong ROA induced chirality, from a chiral Ni-complex to solvent molecules,^8^ by SERS-like transfer of energy from plasmons inducing a 100-fold ROA enhancement. In a newly published Wu *et al.* study,^9^ the induced chirality has been interpreted as arising from interference between ECD and ROA effects. Oddly enough, the effect has been observed even without physical contact between the solute and solvent. The reasoning has been, however, partially using magnetic ROA measurements which, so far, have been done exceptionally rarely and are not yet well-recognized.^10,11^

**Scheme 1:**
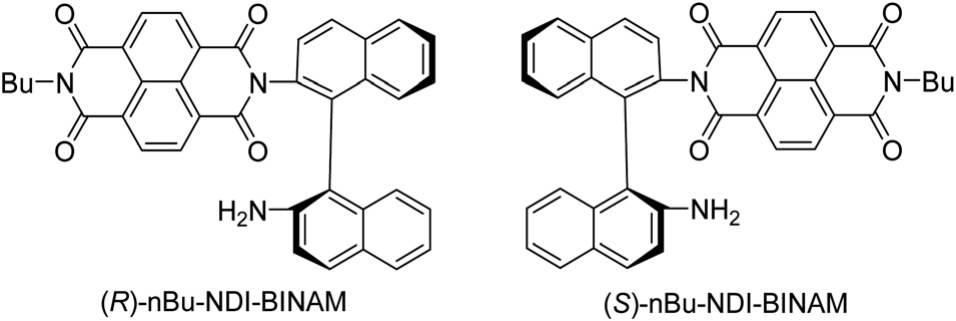
Enantiomers of *n*Bu-NDI-BINAM.

**Fig. 1:**
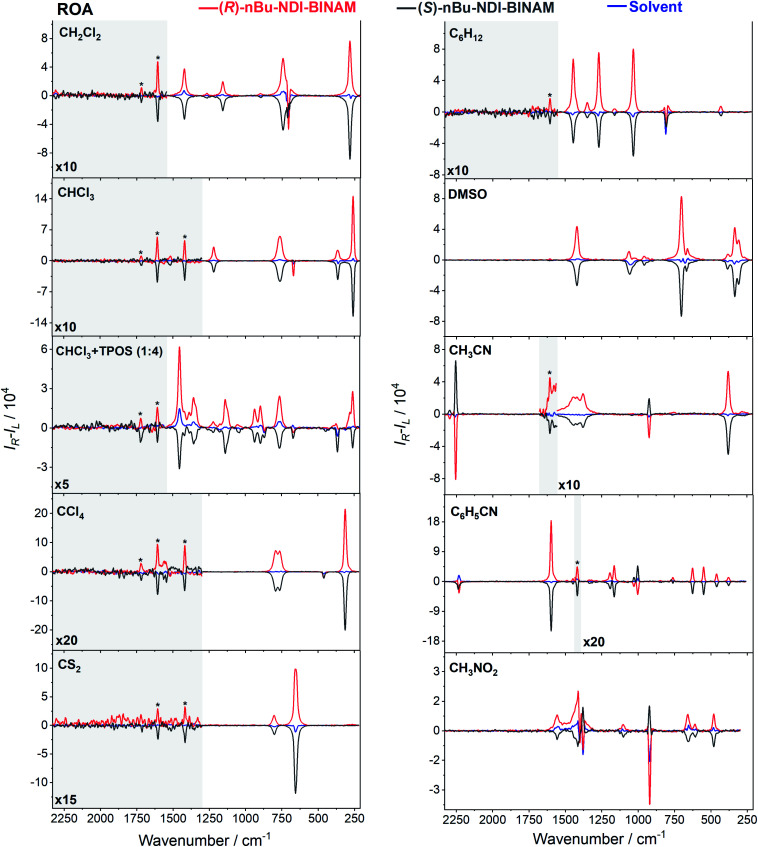
The ROA spectra of (*R*)- and (*S*)-*n*Bu-NDI–BINAM (denoted by an asterisk) dissolved in different solvents.

**Fig. 2:**
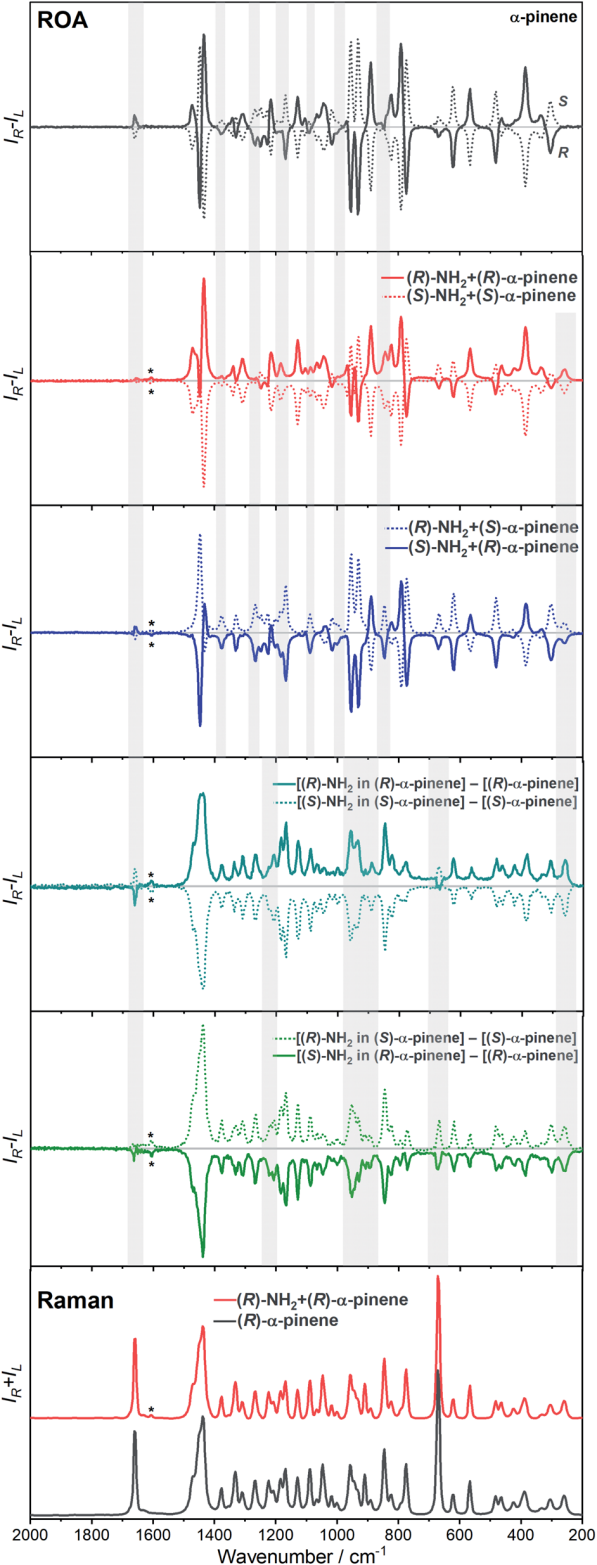
The ROA spectra of (*R*)- and (*S*)-*n*Bu-NDI–BINAM (*R*-NH_2_ and *S*-NH_2_, respectively) dissolved in (*S*)- and (*R*)-α-pinene. Asterisks denote the *n*Bu-NDI–BINAM bands. Grey belts highlight induced chirality marker bands.

Another astonishing example of induced chirality in ROA was found here when characterizing an atropisomeric naphthalenediimide derivative (*n*Bu-NDI–BINAM, Scheme 1), which is chiral due to binaphthalenylamine (BINAM) and naphthalenediimide (NDI) steric hindrance. Enantiomers of (*R*) and (*S*)-*n*Bu-NDI–BINAM were prepared by condensation of (*R*) or (*S*)-1,1′-binaphthyl-2,2′-diamine with *N*-butyl-1,4,5,8-naphthalenetetracarboxylic-1,8-anhydride-4,5-imide at 120 °C in pyridine (see the ESI† for details). The products were purified by column chromatography. The structure and purity were confirmed by NMR spectroscopy, MS and chiral HPLC (Fig. S1–S3†). The ROA spectra of *n*Bu-NDI–BINAM recorded in CH_2_Cl_2_, CHCl_3_, CCl_4_, CS_2_, C_6_H_12_, (*S*)- and (*R*)-α-pinene, CH_3_CN, C_6_H_5_CN, CH_3_NO_2_, DMSO and a 1 : 4 v/v mixture of CHCl_3_ and bulky TPOS (tetraisopropyl orthosilicate) (Fig. 1, 2, and S4–S13 and Tables S1–S3†) exhibit RROA features absent in the single electronic state (SES) theory.^12–14^

The SES theory of the resonance ROA (RROA) by Nafie^12–14^ says that the RROA spectrum: (i) is monosignate, (ii) has a sign opposite to that of the ECD maximum corresponding to the resonant single electronic state, (iii) has relative intensities as in the parent resonance Raman (RR) spectrum, and (iv) for all ROA bands, shows an intensity ratio of RROA/RR equal to the −ECD/2UV ratio of the appropriate electronic band.^12–14^ Nevertheless, the pre-resonance ROA can already be bisignate,^12–14^ similarly to RROA when more electronic states,^14–17^ or conformers are involved.^18,19^ Here, only the longest wavelength (*S*)-*n*Bu-NDI–BINAM ECD band (LWB) is near the 532 nm laser line and it has a negative sign (Fig. S14 and S15 and Table S4†). Because *n*Bu-NDI–BINAM seems to be rigid, the SES theory appears to be satisfactory, and monosignate RROA with a sign opposite to that of the LWB has been expected. Yet, the observed ROA spectra disagree with this pattern and reveal features of the ROA chirality transfer not observed, so far.

## Results and discussion

The ROA spectra of *n*Bu-NDI–BINAM in solutions (Fig. 1, 2) are astonishing and spectacular: First, the *n*Bu-NDI–BINAM ROA shows strong bands of achiral solvents. Second, the solute ROA bands are barely visible. Third, in all solvents but C_6_H_5_CN, CH_3_CN, CH_3_NO_2_, and (*S*)- and (*R*)-α-pinenes, the RROA and LWB ECD bands have the same sign. Fourth, ROA in C_6_H_5_CN, CH_3_CN, and CH_3_NO_2_ is bisignate and the LWB distance from the incident beam frequency plays no role. Fifth, the spectral subtraction reveals the bisignate ROA in (*S*)- and (*R*)-α-pinenes to be a superposition of the ordinary non-resonance and resonance induced chirality components (Fig. 2). The former is bisignate and identical with the common ROA of α-pinene, whereas the latter is monosignate with the sign concordant with the LWB. Definitely, the observed ROA effect is extremely strong (Fig. 1, 2 and S4†), repeatable (Fig. S5†) and free of artefacts (Fig. S8†). Note that BINAM itself – the chiral substituent of NDI – does not exhibit these features (Fig. S16†).

Previously, the ROA induced chirality effect with huge solvent and small solute bands as in Fig. 1 and 2 was explained by resonance energy transfer (RET) from the solute to solvent in the first solvation sphere,^7^ by the plasmon RET in Ni complex solutions,^8^ and by interference between ECD and ROA effects.^9^ In RET, the energy excess in the excited moiety (donor) is transferred to the acceptor one *via* virtual photon emission governed primarily by dipole–dipole couplings.^20^ We also see the RET mechanisms to be responsible for the massive ROA induced chirality, but first, look at the complexity of RETs that can occur here.

**Fig. 3:**
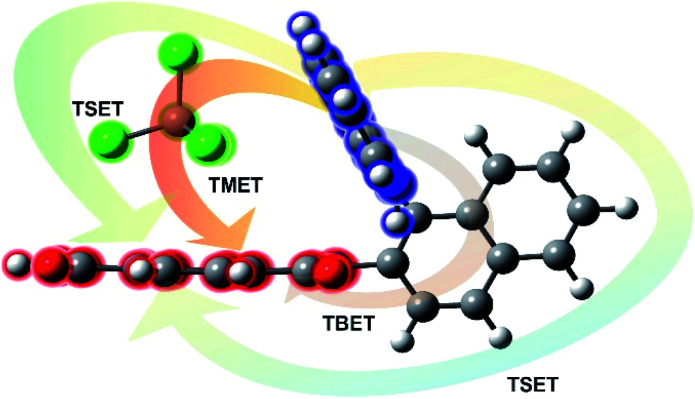
Scheme of different possibilities of the resonance energy transfer processes that may be competitive in the studied systems: TBET – through-bond energy transfer, TSET – through-space energy transfer, and TMET – through mediated molecule energy transfer.

**Fig. 4:**
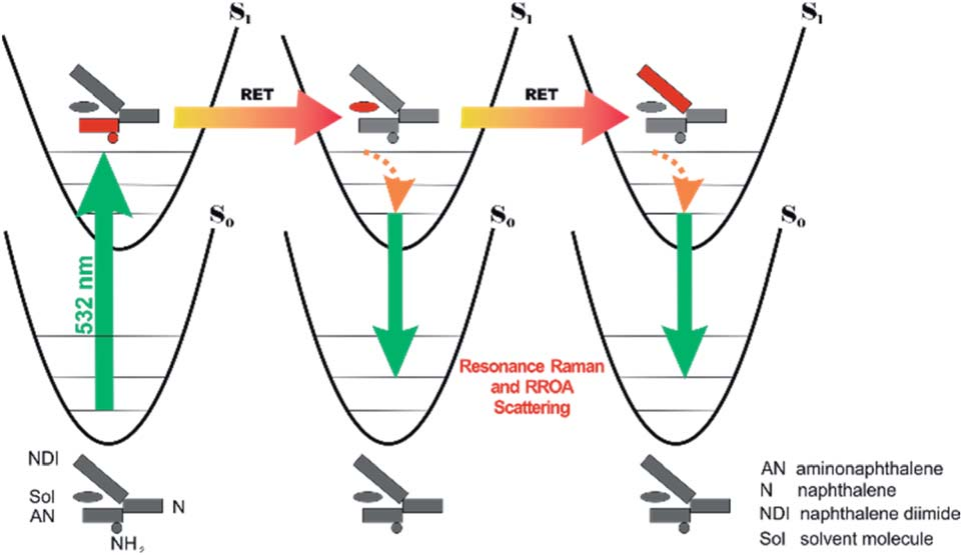
Illustration why resonance ROA induced chirality bands are observed in the *n*Bu-NDI–BINAM solutions. The grey and red segments denote deactivation and excitation, respectively. RET stands for resonance energy transfer.

All RETs important in the studied *n*Bu-NDI–BINAM system start with the aminonaphthalene (AN) donor excitation and end up at the NDI acceptor. The intermediate RET stages can be the following: (i) a direct resonance energy transfer from AN to NDI without an intermediate excitation; (ii) a through-bond energy transfer *via* naphthalene excitation; (iii) RETs through-the-first-solvation-sphere and through-the-bulk-solvent accompanied by excitation of a molecule from the first solvation sphere and molecules in bulk, respectively (Fig. 3). In RET processes the solvent molecules in the first and in the bulk spheres can behave differently,^20^ and estimation of their effectiveness needs careful quantum electrodynamics analysis. In the former, the solvent molecules share the solute electronic space^7^ in the common potential well. Thus, the donor resonance excitation is transferred to the solvent molecule remaining in resonance. Then, it is resonantly Raman-scattered from this molecule, or, after further RET, from the acceptor (Fig. 4). In the latter, the bulk solvent molecules are distanced from the solute and reside in their own potential wells. Thus, the non-resonance Raman scattering from the solvent molecule in its own potential well, described by Andrews,^21^ is expected (Fig. S17†). Eventually, it can be noted that in the HQ dye and Ni-complex,^7,8^ the donor and acceptor moieties are located in close proximity and all the above considerations can be valid. Indeed, it was concluded that the most strongly scattering ACN molecules were those from the first solvation sphere of HQ.^7^

The RROA and ECD sign concordance has not been observed until the last minute where the systems have been interpreted without conformational and excited state analyses.^9^ Wu *et al.* argued that the induced chirality in ROA comes from the interference of ECD and polarized Raman scattering, and showed that the different ECD profiles within the ROA range differently modified the induced chirality ROA bands.^9^ As a result, the induced chirality ROA bands of the solvent were monosignate and concordant with the sign of the involved ECD band. This was the case for their chiral Cu complexes for which constant ECD intensity in the scattering range was observed. In contrast, for the Ni the bisignate ROA induced chirality of the solvent resulted from a quick ECD intensity decrease in the range with a maximum at the excitation wavelength (532 nm). The effect may also occur for the *n*Bu-NDI–BINAM solutions. However, the presented induced chirality ROA bands are monosignate for *e.g.* CHCl_3_ and bisignate for *e.g.* CH_3_CN, which seems to not fully confirm the Wu *et al.* model, because the ECD profiles of these systems are fairly identical in the range of the ROA scattering (Fig. S14†).

**Fig. 5:**
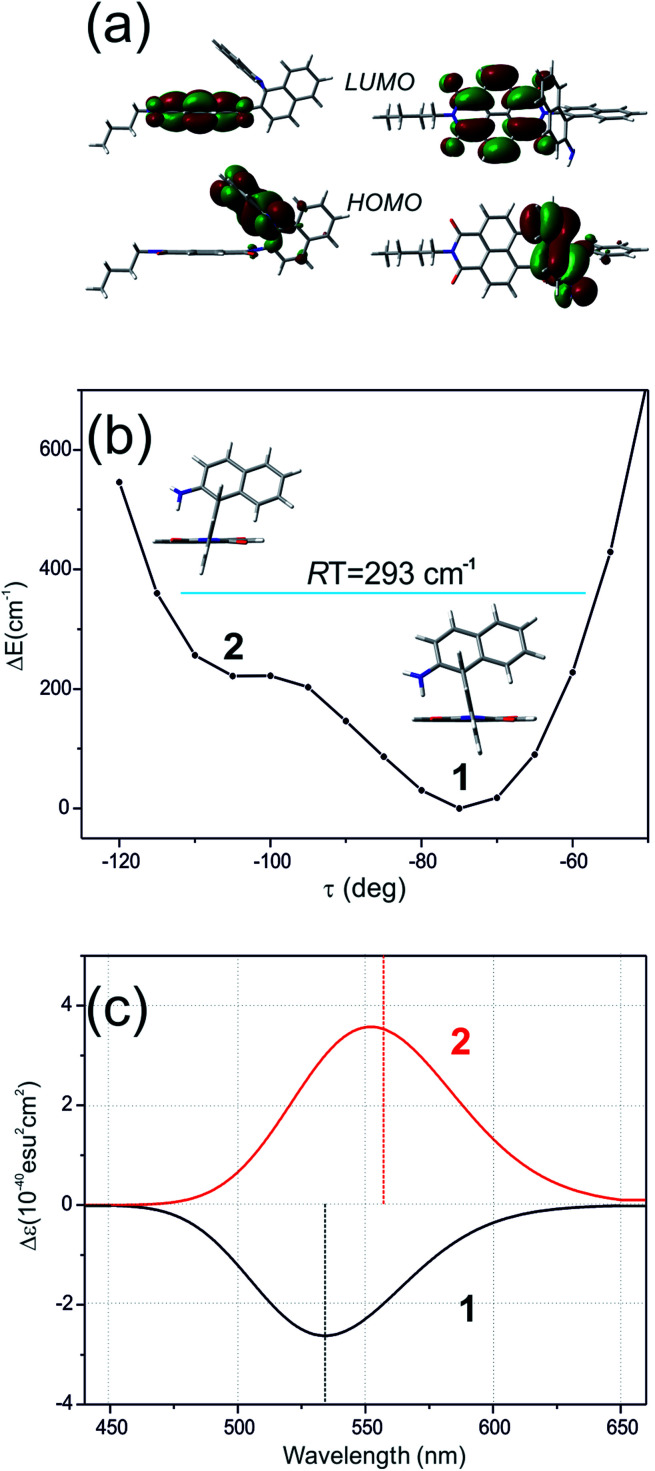
(a) The HOMO and LUMO in *n*Bu-NDI–BINAM. (b) The CAM-B3LYP/D3/TZV calculated (*S*)-NDI–BINAM potential energy profile against the naphthalene linker twist angle *τ*, and (c) ECD spectra of the stable (1, black) and unstable (2, red) conformers.

Here, the TD-DFT calculated ECD spectra of the single *n*Bu-NDI–BINAM molecule indicate that the LWB responsible for resonance is due to the HOMO → LUMO transition *i.e.* transition from AN to NDI (Fig. 5a, S18, and S19 and Tables S5 and S6†). Although it is known that RROA intensity can be “borrowed” from an absorption band distanced by more than 100 nm,^14^ here, knowing some similar systems without the LWB, we know that this is not the case. The close proximity of the π-electron withdrawing NDI and π-electron donating AN moieties enables the formation of the intramolecular electron donor–acceptor interaction stabilizing the *n*Bu-NDI–BINAM by additional *ca.* 17 kcal mol^−1^ (Fig. S20†) which is revealed only after including dispersion forces significantly reducing the distance between the NDI and AN planes (Fig. S21†).

**Fig. 6:**
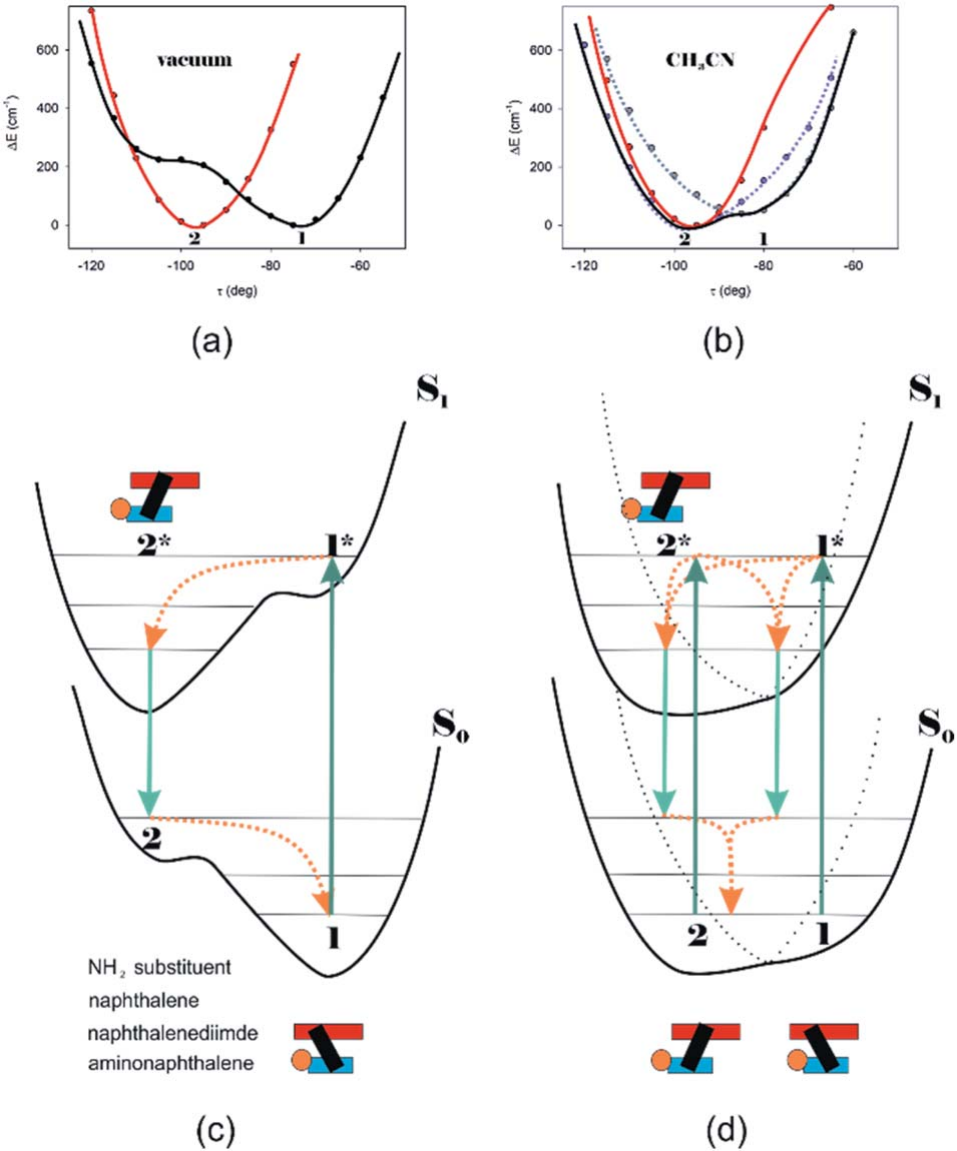
The (TD)-CAM-B3LYP/D3/TZV calculated S_0_ (black) and S_1_ (red) state potential energy profiles against the naphthalene twist *τ* angle for (a) NDI–BINAM in a vacuum also representing CCl_4_, CH_2_Cl_2_, CHCl_3_, CS_2_, C_6_H_12_, DMSO, and pinene solutions, and (b) CH_3_CN@(NDI–BINAM) also representing CH_3_NO_2_ and C_6_H_5_CN solutions where different Sol@(NDI–BINAM) produces profile asymmetry (grey and violet dotted branches). *ca.* 40 kcal mol^−1^ downshift of the S_1_ potentials is to highlight the differences. (c) and (d) the S_0_ and excited S_1_ asymmetric potential profiles illustrating the genesis of the RROA and ECD sign concordance, and (d) bisignate RROA: dotted branches denote the presence of different Sol@(NDI–BINAM) positions. Green and orange denote radiative and radiationless processes, respectively.

Different calculations congruently predicted one stable NDI–BINAM conformer 1; however, the second one 2 was found with the small TZV basis set (Fig. 5b). The linking naphthalene plane is twisted with respect to NDI in 1 and 2 by *ca.* −75 and −105 deg, respectively. The presence of 2 produces just the potential profile asymmetry (Fig. 5b). Still, at 300 K, the RT thermal energy factor is high enough for NDI–BINAM to adopt conformation 2. Surprisingly, for 2, the calculated LWB ECD band has a sign opposite to that of 1 (Fig. 5c). However, in the first excited singlet S_1_ state, the 2* conformer (*τ* ≈ −96.5°) appeared to be the only stable one (red curve, Fig. 6a and b).

To solve the puzzle of monosignate/bisignate induced chirality ROA bands we calculated the 1 : 1 solute–solvent complexes. The solvent molecule inside the AN–NDI gap positioned exactly between the HOMO and LUMO states, Sol@*n*Bu-NDI–BINAM, can play a distinguished role for the observed effect. The energy potential profiles for the ground and first excited singlet states of Sol@(NDI–BINAM), S_0_ and S_1_, respectively, demonstrated minima at *τ* ≈ −75° for all but C_6_H_5_CN, CH_3_CN, and CH_3_NO_2_ systems for which *τ* ≈ −90° and the NDI-AN distance is *ca.* 0.4 Å larger (Fig. 6a, b, S22, and S23 and Table S7†). Thus, these three flat molecules widen and penetrate the NDI–AN gap deeper than the spherical ones. In opposition, all the S_1_ profiles exhibit minima corresponding to the conformer 2* and the absence of the well-formed minima of 1* (Fig. 6a, b and S23†). In consequence, in the S_0_ and S_1_ states, different *n*Bu-NDI–BINAM conformers are engaged in the resonance Raman scattering (Fig. 6c and d). For CH_2_Cl_2_, CHCl_3_, CCl_4_, CS_2_, C_6_H_12_, DMSO, and CHCl_3_ + TPOS solvents, for which monosignate ROA bands are concordant with the LWB ECD band (Fig. 1), the explanation is the following: the green light vertically excites 1 (the only conformer in S_0_) to 1* which is an unstable conformer in the S_1_ state. 1* transforms to 2* in vibrational relaxations. Only 2* participates in the “emission part” of the Raman scattering. The sign of such “circularly polarized emission” is identical to that of the LWB ECD band of 2, which for NDI–BINAM is opposite to that of 1 (Fig. 5c). Thus, the RROA sign behaves as if it would be first inverted as predicted by the SES theory and then inverted again because of the opposite sign of the scattering from 2*. So, the atypical RROA sign-switch is a result of unusual conformational equilibria in the S_0_ and S_1_ states: one conformer dominates in the ground while the other in the excited state, plus the appropriate ECD band signs are opposite.

On the other hand, the bisignate instead of monosignate RROA spectra for C_6_H_5_CN, CH_3_CN, or CH_3_NO_2_ (Fig. 1) are due to few different locations of the Sol molecules with respect to the AN–NDI gap (Fig. 6 and S22†). As a result, the S_0_ and S_1_ potential contours are asymmetric and also include both conformers simultaneously (Fig. 6b). Therefore, 1 and 2 can be excited and the light may be scattered from 1* and 2* yielding the bisignate RROA pattern; nevertheless, in agreement with the SES theory, the resonance contributions to the RROA spectra are monosignate. Note that in the other solvents, the potentials inside and outside the gap are qualitatively the same and produce the same RROA pattern. Such an ROA sign-switching conformational change in the pathway between the ground and excited state potential surfaces is novel. It seems however that structural changes in this specific system can inspire the idea of the construction of an ROA-induced chirality signaling device. Indeed, fixing the ground state geometry would produce the same geometry in the excited state and the ROA signal sign opposite to that of the involved ECD band, while leaving the present conformational freedom would produce a signal concordant with the ECD band.

Interestingly, a simultaneous occurrence of the common ROA (panels 2 and 3, Fig. 2) and induced chirality RROA (panels 3 and 4, Fig. 2) for (*S*)- and (*R*)-α-pinene takes place. The bisignate ROA component, identical with the ROA of α-pinene, comes from the common ROA scattering of the bulk pinene. However, a resonance energy transfer through-the-bulk-solvent mechanism coupled with the Raman emission described by Andrews^21^ (Fig. S17†) can also contribute to this component. The monosignate induced chirality ROA spectrum from solvent molecules close to *n*Bu-NDI–BINAM has a resonance RROA character (Fig. 2). Moreover, it has the sign concordant with the LWB of *n*Bu-NDI–BINAM which is always negative or positive for the (*S*)- or (*R*)- enantiomers, respectively, regardless of whether it is dissolved in (*R*)- or (*S*)-pinene, (Fig. S14†). Note that presence of the monosignate resonance ROA contribution for pinenes implicates that the other induced chirality spectra (Fig. 1) have resonance rather than non-resonance character.

Additionally, the solvent molecules from both the nearest solute vicinity and those a bit more distanced participate in the RROA induced chirality effect. Note the presence of both CHCl_3_ and TPOS induced chirality RROA bands in the mixed solvent system (Fig. 2). *n*Bu-NDI–BINAM is insoluble in pure TPOS; thus in the first solvation sphere mostly the CHCl_3_ molecules are present while the TPOS ones are more distanced. If so, then the simultaneous presence of the CHCl_3_ and TPOS RROA signals indicates that both short- and middle-range RET processes, from CHCl_3_ and TPOS, respectively, occur.

## Conclusions

Studies of the chiral *n*Bu-NDI–BINAM to solvent ROA induced chirality confirm previous suggestions of the resonance energy transfer mechanism behind the extraordinary ROA enhancement. However, we argue for the RET mechanism similar to that described by Andrews where RET is coupled with Raman emission.^21^ Furthermore, we show reasons supporting the RROA rather than the non-resonance ROA effect. We have explained the unusual ROA sign-switching effect based on specific conformer equilibria in the ground and excited states where different conformers dominate in different states. Eventually, we argue for the RET mechanisms behind observed ROA induced chirality acting in short- and middle-ranges.

## Conflicts of interest

All the authors have given approval to the final version of the manuscript and declare no competing financial interest.

## Supplementary Material

SC-012-D0SC05345G-s001
