# Supplementary material for: On Raman optical activity sign-switching between the ground and excited states leading to an unusual resonance ROA induced chirality
Source: Chem Sci. 2020 Nov 2;12(3):911–6. doi: 10.1039/d0sc05345g (PMC8179112; doi:10.1039/d0sc05345g)
Supplement: SC-012-D0SC05345G-s001 [file SC-012-D0SC05345G-s001.pdf]

Electronic Supplementary Material (ESI) for Chemical Science.
This journal is © The Royal Society of Chemistry 2020

Supporting Information

On Raman optical activity sign-switching between the ground and excited states leading to an unusual resonance ROA induced chirality

Ewa Machalska,[a,b] Grzegorz Zajac,[b] Malgorzata Baranska,*[a,b] Dorota Kaczorek,[c] Robert Kawęcki,*[c] Piotr F. J. Lipiński,[d] Joanna E. Rode,[e] Jan Cz. Dobrowolski*[e]

[a] Faculty of Chemistry, Jagiellonian University, Gronostajowa 2, 30-387 Cracow (Poland)

[b] Jagiellonian Centre for Experimental Therapeutics (JCET), Jagiellonian University, Bobrzynskiego 14, 30-348 Cracow (Poland)

[c] Siedlce University, Faculty of Science, 3 Maja Street No 54, 08-110 Siedlce (Poland)

[d] Department of Neuropeptides, Mossakowski Medical Research Centre, Polish Academy of Sciences, 02-106 Warsaw (Poland)

[e] Institute of Nuclear Chemistry and Technology, 16 Dorodna-Street, 03-195 Warsaw (Poland)

Table of Contents

Experimental 3

1. Synthesis 3

Figure S1. ^{1}H NMR and ^{13}C NMR spectra of nBu-NDI-BINAM 4

Figure S2. High resolution mass spectrum of nBu-NDI-BINAM and its predicted isotopic pattern 5

Figure S3. Chiral HPLC chromatogram of nBu-NDI-BINAM 5

2. Raman and ROA measurements 6

Scheme S1. Structure of tetrapropyl orthosilicate (TPOS) 6

Table S1. Raman and ROA measurement parameters 6

Figure S4. Comparison of Raman and ROA spectra for $CHCl_3$ and (*R*)-nBu-NDI-BINAM in $CHCl_3$ measured at different power of laser 7

Figure S5. ROA spectra of nBu-NDI-BINAM enantiomers in CH_3CN and $CHCl_3$ obtained from two different measurements 7

Figure S6. Raman and ROA spectra of $CHCl_3$ and nBu-NDI-BINAM enantiomers in $CHCl_3$ averaged for 6, 12, 24 and 46 hours of acquisition 7

Figure S7. Set of ROA spectra of $CHCl_3$ and nBu-NDI-BINAM enantiomers in $CHCl_3$ averaged every 2 hour blocks 8

Figure S8. Comparison of shot noise floor values and ROA spectra for pure $CHCl_3$ and nBu-NDI-BINAM in $CHCl_3$ 8

Figure S9. Comparison of ROA spectra for nBu-NDI-BINAM enantiomers in $CHCl_3$ measured at different power of laser 9

Figure S10. Raman spectra of (*S*)-nBu-NDI-BINAM measured in different achiral solvents 11

Figure S11. Solvent corrected Raman spectra of nBu-NDI-BINAM 11

Figure S12. Raman spectra of solid (*S*)-nBu-NDI-BINAM. 12

Table S2. Assignments of Raman bands of nBu-NDI-BINAM in $CHCl_3$ 12

Table S3. List of induced and natural chirality ROA bands of nBu-NDI-BINAM measured in (α)-pinene 13

Figure S13. ROA spectra of nBu-NDI-BINAM measured in (α)-pinene 13

3. UV-Vis and ECD measurements 14

Figure S14. Juxtaposition of the ECD spectra of (*S*)-nBu-NDI-BINAM in different solvents 14

Figure S15. UV-Vis and ECD spectra of (*S*)- and (*R*)-nBu-NDI-BINAM in (α)-pinene and of (*S*)-nBu-NDI-BINAM in different achiral solvents 16

4. Analysis of the UV-Vis/ECD and Raman/ROA data 17

Table S4. UV-Vis, ECD, Raman, and ROA maxima of (*S*)-nBu-NDI-BINAM and the CID, *g*, κ ratios 17

5. Measurements of chiral BINAM 19

Figure S16. UV-Vis, ECD, Raman and ROA spectra of BINAM 19

6. Resonance Energy Transfer 20

Figure S17. Schematic explanation of the non-resonance Raman scattering in nBu-NDI-BINAM 21

Calculations 22

Scheme S2. Two nBu-NDI-BINAM conformers associated with the nBu chain geometry 22

Scheme S3. The dihedral angle τ changed in the (*S*)-NDI-BINAM energy scans 22

Scheme S4. Two models applied in the EET calculations of (1:1) Sol@NDI-BINAM complexes 23

Table S5. Calculated UV-Vis and ECD spectra of (*S*)-nBu-NDI-BINAM 24

Table S6. Influence of the solvent on experimental and calculated longest wavelength band location of (*S*)-nBu-NDI-BINAM 25

Figure S18. Calculated position of the charge-transfer LWB band of (S)-nBu-NDI-BINAM in different solvents 25

Figure S19. The most stable systems of nBu-NDI-BINAM surrounded by 11 or 9 solvent molecules 26

Figure S20. Structures of the NDI-BINAM analogs with the aminonaphthalene group attached in different positions of the linking naphthalene moiety 27

Figure S21. Calculated nBu-NDI-BINAM structure with and without the D3 correction for dispersion 27

Table S7. Energetics of the (1:1) complexes of (S)-NDI-BINAM with different solvents located in the NDI-AN cavity. 28

Figure S22. Structures of the most stable (1:1) complexes of (*S*)-NDI-BINAM with different solvents located in the NDI and AN cavity 31

Figure S23. Calculated ground state and the first excited singlet state potential energy profiles of the Sol@NDI-BINAM complexes 32

Table S8. Estimated values of the EET coupling components for the energy transfer in the first model for the Sol@(NDI-BINAM) systems 33

Table S9.Estimated values of the EET coupling components for the energy transfer in the second model for the Sol@(NDI-BINAM) systems 34

References 35

Appendix 37

Table S10. Cartesian coordinates of (*S*)-nBu-NDI-BINAM surrounded by 11 (CS_2, CCl_4, $CHCl_3$, CH_2Cl_2, CH_3CN) or 9 (C_6H_{12}) solvents molecules. 37

Table S11. Cartesian coordinates of the most stable (1:1) complexes of (*S*)-NDI-BINAM with different solvents 42

Experimental

1. Synthesis

All commercially obtained reagents were used as received unless otherwise stated. Column chromatography was performed using Acros Organics 60 Å silica gel with the indicated solvents. Nuclear magnetic resonance spectra were recorded on a 400 MHz instrument and were referenced to residual solvent signals (chloroform δ = 7.26 and 77.0 ppm for ^{1}H NMR and ^{13}C NMR, respectively). High-resolution mass spectra (HRMS) were obtained using electrospray ionization source. Microwave irradiation was performed in Prolabo Synthwave 402 reactor (open system).

Asymmetric synthesis of nBu-NDI-BINAM

***N*-Butyl-1,4,5,8-naphthalenetetracarboxylic-1,8-anhydride-4,5-imide**

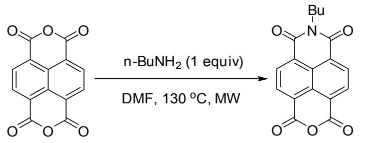

This compound was obtained by modified method of Parquette et al.[1] The suspension of 1,4,5,8-naphthalenetetracarboxylic dianhydride (0.500g, 1.87 mmol) in DMF (15 mL) was stirred at 130 °C for 15 min. The solution of n-butylamine (136 mg, 1,86 mmol) in DMF (3 mL) was slowly added over a period of 45 min. The clear solution was transferred to quartz tube and microwave irradiated at 130 °C for 35 min. DMF was evaporated and the residue was dissolved in CH_2Cl_2. The black precipitate was filtered off. The filtrate was concentrated to give beige crystals (668 mg) which were dried on high vacuum. The mixture contains 25mol% of *N,N′*-dibutyl substituted naphthalenediimide which may be removed by column chromatography on silica gel using CH_2Cl_2 as an eluent. Preferably, the mixture of imides may be used in the next step.

^{1}H NMR ($CDCl_3$) δ: 0.99 (t, J=7.0 Hz, 3H); 1.46 (sex, J=7.0 Hz, 2H); 1.73 (m, 2H); 4.21 (m, 2H); 8.82 (s, 4H). NMR data are in agreement with those published earlier[1].

(*S*)-*N*-(2'-Amino-[1,1']binaphthalen-2-yl)-*N*-butyl-naphthalene-1,4,5,8-tetracarboxylic acid bisimide [(*S*)-nBu-NDI-BINAM]

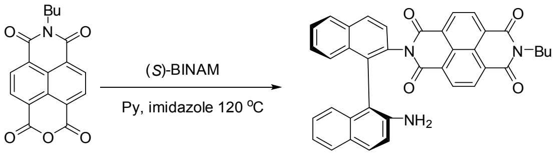

The solution of *N*-butyl-1,4,5,8-naphthalenetetracarboxylic-1,8-anhydride-4,5-imide (103 mg, 0.32 mmol), (*S*)-1,1′-binaphthyl-2,2′-diamine (82 mg, 0.29 mmol) and imidazole (186 mg, 2.7 mmol) in pyridine (5.5 mL) was heated in oil bath (120 °C) for 17 hours. The mixture was poured to aq. 10% HCl (35 mL) and centrifugated for 10 min at 6000 rpm. The supernatant was removed and the precipitate was washed 2 times with H_2O and once with MeOH in the same manner. The solid residue was dissolved in CH_2Cl_2 and dried with $MgSO_4$. Filtration and evaporation gave dark violet solid which was purified on silica gel using CH_2Cl_2/MeOH (200:1 v/v) as an eluent. Yield 99 mg (58%).

^{1}H NMR (400 MHz, $CDCl_3$) δ: 0.97 (t, *J*=7.36 Hz, 3H), 1.36-1.48 (m, 2H), 1.62-1.73 (m, 2H), 3.84 (br. s., 2H), 4.11-4.18 (m, 2H), 6.86 (d, *J*=8.92 Hz, 1H), 7.01-7.06 (m, 1H), 7.10-7.18 (m, 2H), 7.34-7.39 (m, 2H), 7.47 (dd, *J*=8.29, 5.39 Hz, 2H), 7.58 (ddd, *J*=8.14, 5.34, 2.70 Hz, 1H), 7.62 (d, *J*=8.50 Hz, 1H), 8.04 (d, *J*=8.29 Hz, 1H), 8.17 (d, *J*=8.71 Hz, 1H), 8.40 (d, *J*=7.46 Hz, 1H), 8.55 (d, *J*=7.67 Hz, 1H), 8.58 (d, *J*=7.46 Hz, 1H), 8.64 (d, *J*=7.46 Hz, 1H).

^{13}C NMR ($CDCl_3$), δ: 13.76, 20.28, 30.08, 40.65, 112.45, 118.26, 122.16, 125.26, 126.13, 126.42, 126.46, 126.48, 126.52, 126.68, 126.69, 126.85, 127.16, 127.19, 127.26, 127.61, 128.33, 129.48, 129.66, 130.64, 130.67, 130.77, 130.80, 133.13, 133.15, 133.64, 133.95, 134.04, 142.98, 162.18, 162.67, 162.72, 162.97.

$[\alpha]^{20}_{D}$= -223 (c= 0.28, CH_2Cl_2).

HRMS (ESI) m/z calcd for $C_{38}H_{28}N_3O_4$ $(M+H)^+$ 590.2074. Found: 590.2074.

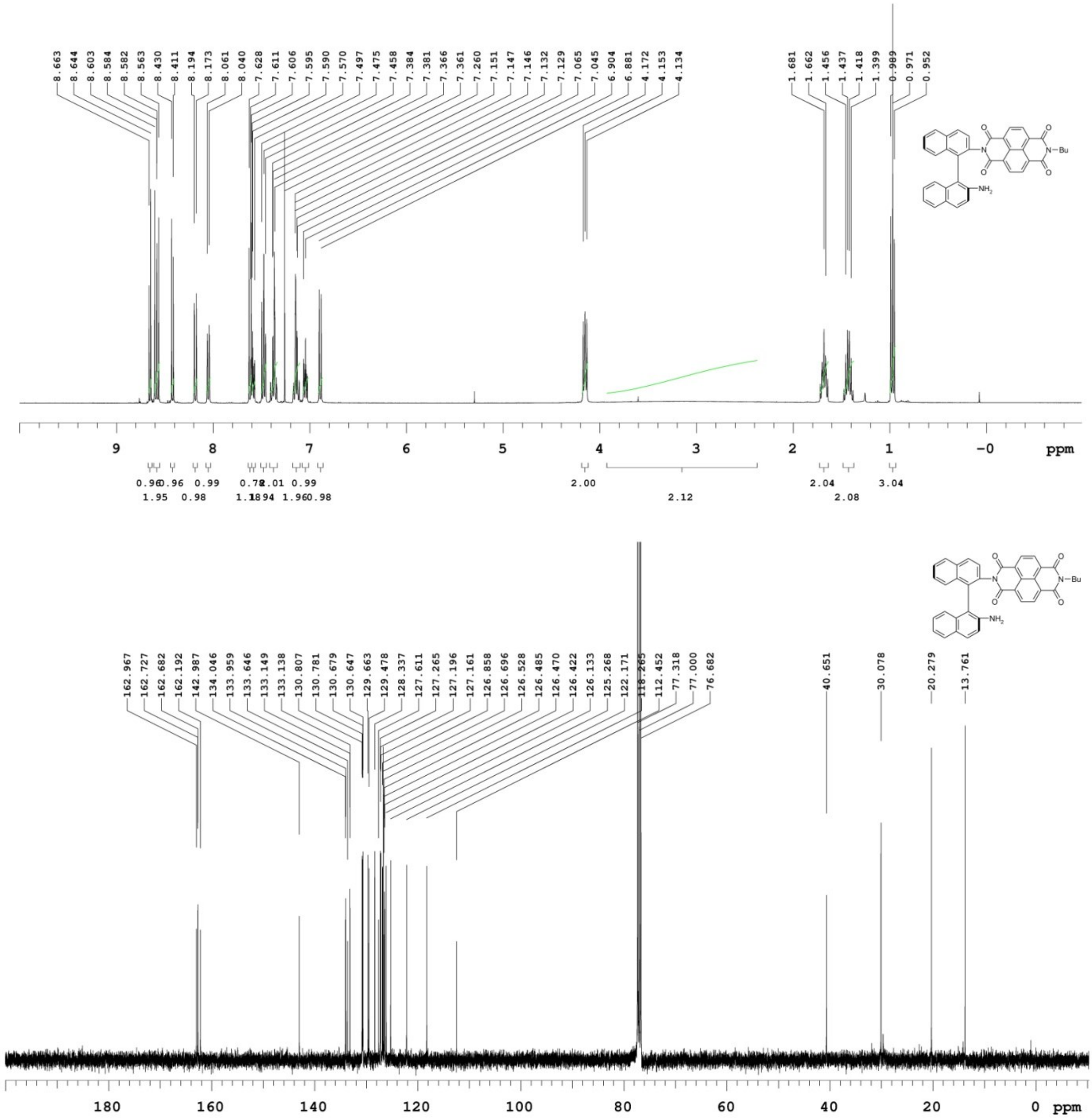

Figure S1. ^{1}H NMR and ^{13}C NMR spectra of nBu-NDI-BINAM.

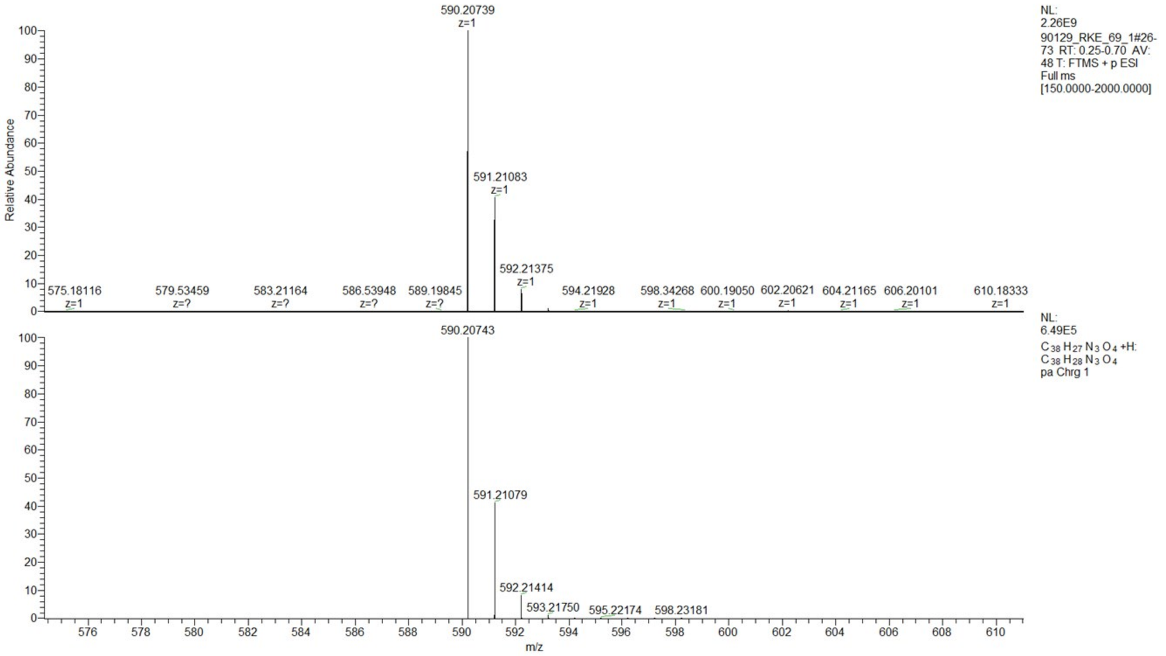

Figure S2. High resolution mass spectrum (ESI) of nBu-NDI-BINAM and its predicted isotopic pattern (bottom).

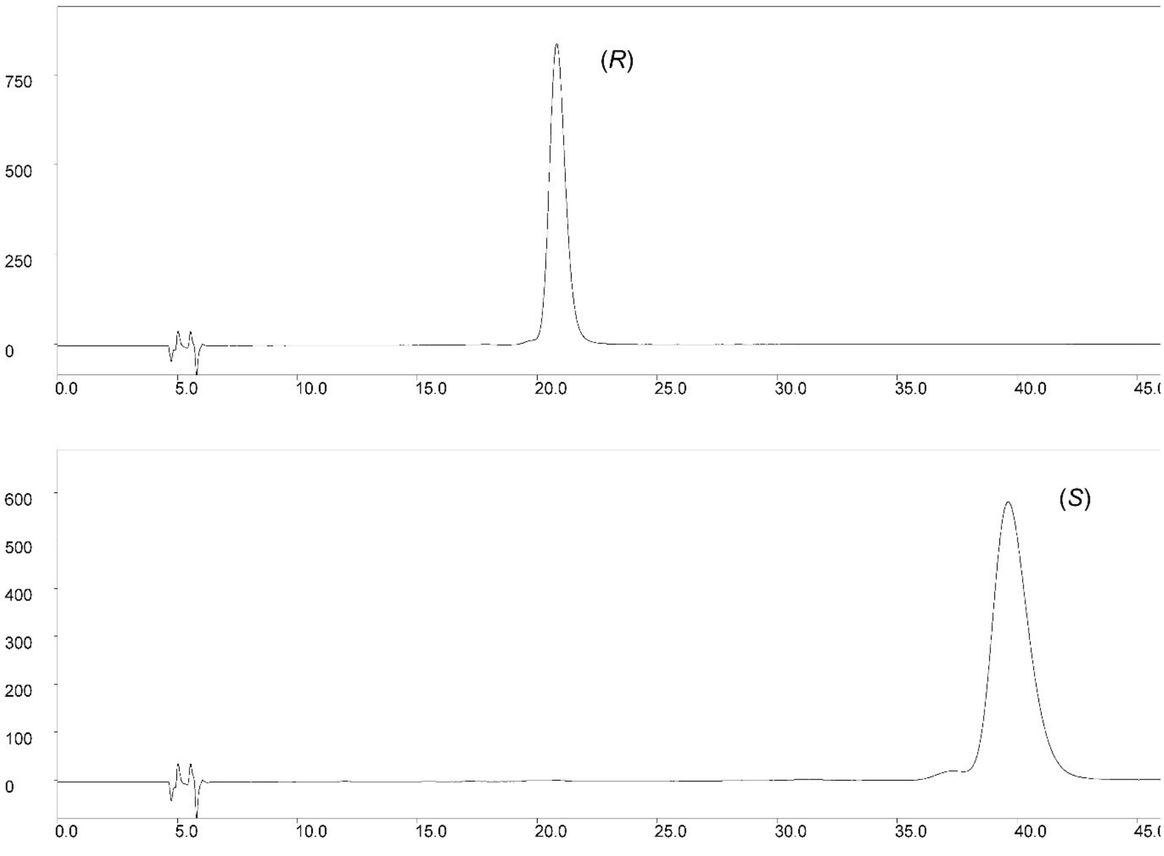

Figure S3. Chiral HPLC chromatogram of nBu-NDI-BINAM. HPLC conditions: Lux Amylose-1, 250x4.6 mm, cyclohexane, 0.1%DEA-isopropanol, 0.1%DEA 60:40, 0.5 mL/min, UV detection 225 nm. Retention times: (*R*)-nBu-NDI-BINAM, 20.81 min; (*S*)-nBu-NDI-BINAM, 39.63 min.

2. Raman and ROA measurements

Raman and ROA spectra of nBu-NDI-BINAM enantiomers in solvents: CH_2Cl_2, $CHCl_3$, CCl_4, CS_2, C_6H_{12}, (*R*)-α- and (*S*)-α-pinene, CH_3CN, C_6H_5CN, CH_3NO_2, DMSO and in the 1:4 v/v mixture of $CHCl_3$ and TPOS (tetrapropyl orthosilicate, $C_{12}H_{28}O_4Si$, **Scheme S1**) were measured using a Chiral*RAMAN-2X* spectrometer (*BioTools Inc.*) at a resolution of 7 cm^{-1} in the range of 2500-180 cm^{-1} employing the excitation wavelength of 532 nm. The solutions of nBu-NDI-BINAM (ca. c=3x10^{-3} mol/dm^3) were measured in ROA quartz optical cells with anti-reflective coating. Before measurements, the nBu-NDI-BINAM solutions were purified by using the activated charcoal and then they were filtered with MilliporeTM Millex® syringe filters (pore size 0.45 µm) to remove chemical impurities. The Raman and ROA spectra were collected in 2.8665 s of integration time. Other experimental conditions such as laser power and data collection time were matched for each sample and solvent individually (**Table S1**).

The power of laser has been chosen to detector saturation remained the same for each ROA/Raman measurement. To obtain equal level of detector saturation for solvents and samples, usually higher laser powers for nBu-NDI-BINAM solutions were used. Nevertheless, strong induced chirality effect for nBu-NDI-BINAM was observed even when the laser power was lower than that for pure solvents (**Figure S4**).

Also, for (*R*)- and (*S*)-nBu-NDI-BINAM in solvents, at least two different Raman/ROA experiments were conducted (over 50 experiments in total) and the obtained ROA spectra were very reproducible (**Figure S5**). The ROA measurements in organic solvents are often associated with polarisation artefacts. In the case presented here, one can notice that no variations in the intensity and sign of ROA signal is observed (**Figure S6** and **S7**). The ROA spectra of (*R*)- and (*S*)-nBu-NDI-BINAM in solvents are outside the square root of Raman intensity noise limit which clearly proves that obtained ROA spectra are not measurement artefacts (**Figure S8**). It can be also seen that pure solvents do not give a chiroptical signal.

Baseline of Raman and ROA spectra were subtracted by asymmetric least squares smoothing method. Then the ROA spectra were smoothed with the ten-point Savitzky–Golay procedure using OriginPro software.

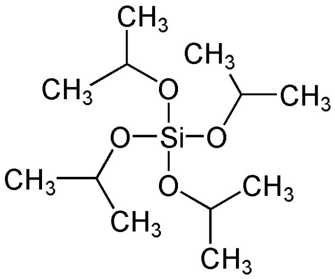

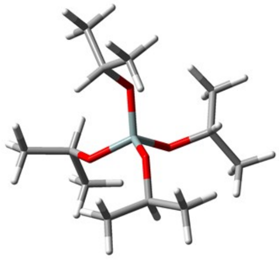

Scheme S1. Structure of tetrapropyl orthosilicate (TPOS).

Table S1. The Raman and ROA measurement parameters.

	Laser power [mW]			Data collection time [h]
	Solvent	(*S*)-nBu-NDI-BINAM + solvent	(*R*)-nBu-NDI-BINAM + solvent	
CH_2Cl_2	90	140	140	48
$CHCl_3$	46	140	140	48
CCl_4	26	160	160	24
CS_2	6	20	20	24
C_6H_{12}	60	70	70	24
(*S*)-α-pinene	56	246	200	24
(*R*)-α-pinene	56	60	170	24
CH_3CN	46	100	100	24
C_6H_5CN	20	50	50	24
CH_3NO_2	46	40	44	24
DMSO	24	60	60	24
$CHCl_3$ + TPOS (1:4)	60	170	170	24

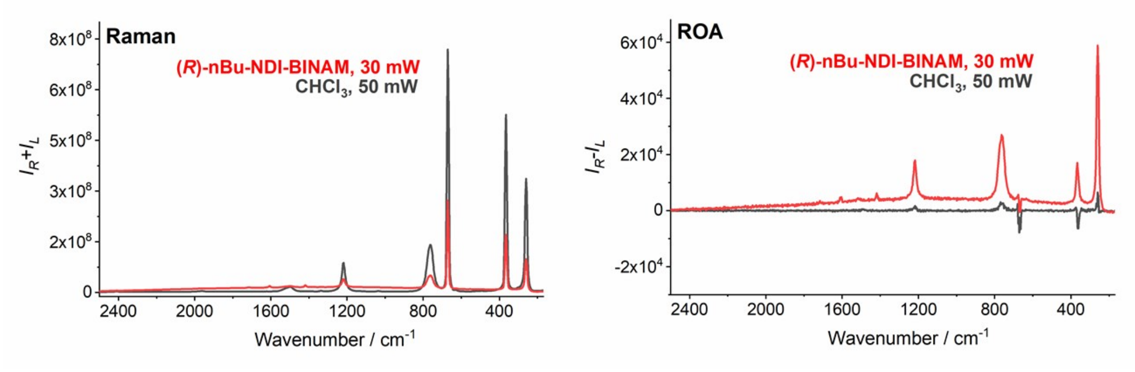

Figure S4. Comparison of Raman and ROA spectra for $CHCl_3$ and (*R*)-nBu-NDI-BINAM in $CHCl_3$ measured at different laser powers. Strong induced chirality effect for dissolved nBu-NDI-BINAM was observed even when the laser power was lower than that for pure solvent.

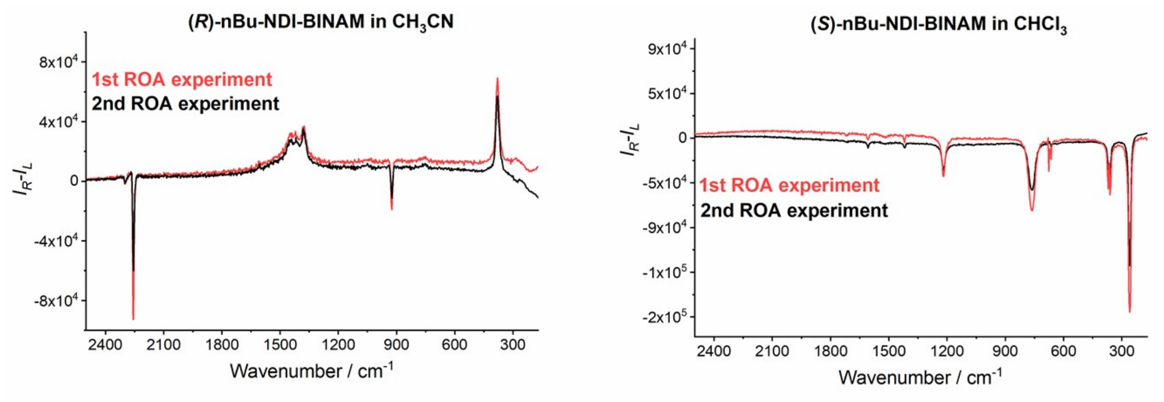

Figure S5. ROA spectra of nBu-NDI-BINAM enantiomers measured in CH_3CN and $CHCl_3$ obtained from two different measurements from two individual samples.

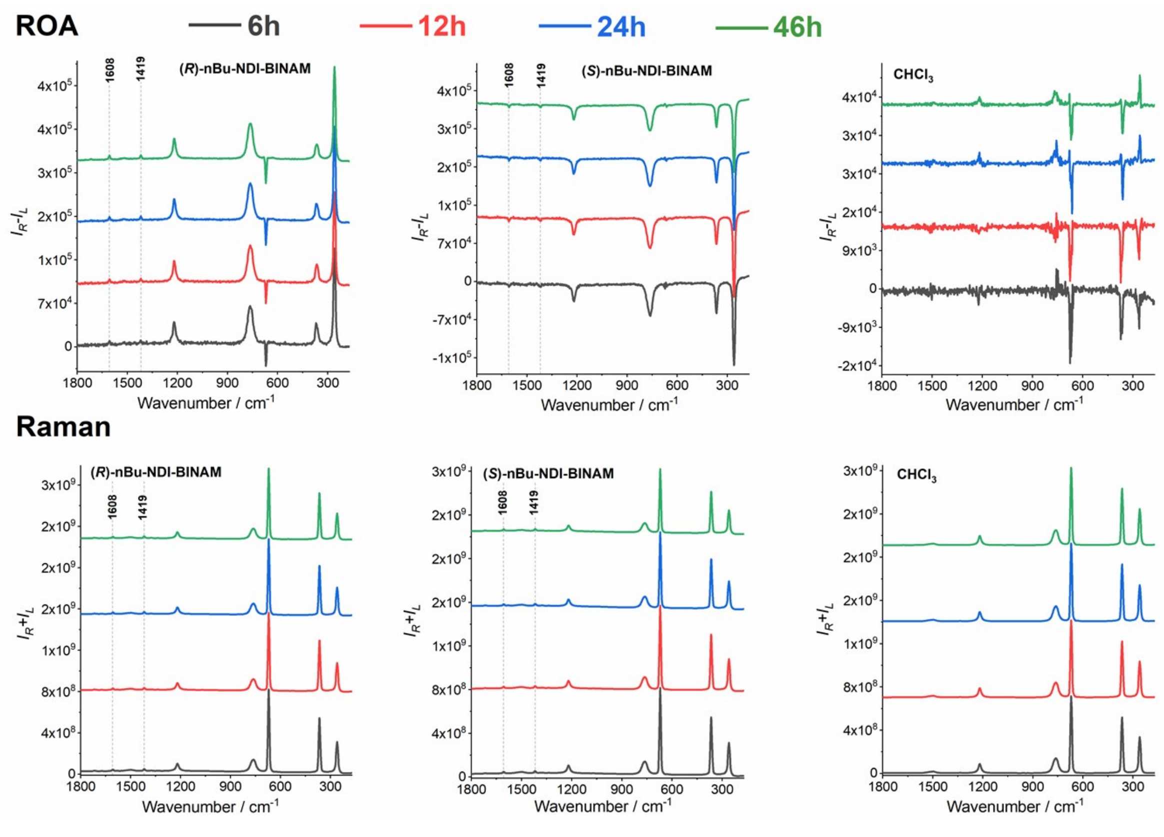

Figure S6. Raman and ROA spectra of $CHCl_3$ and nBu-NDI-BINAM enantiomers measured in $CHCl_3$. The spectra were averaged approximately over 72, 144, 288 and 552 blocks for 6, 12, 24 and 46 hours of acquisition, respectively. The grey dotted lines indicate the Raman/ROA bands of nBu-NDI-BINAM.

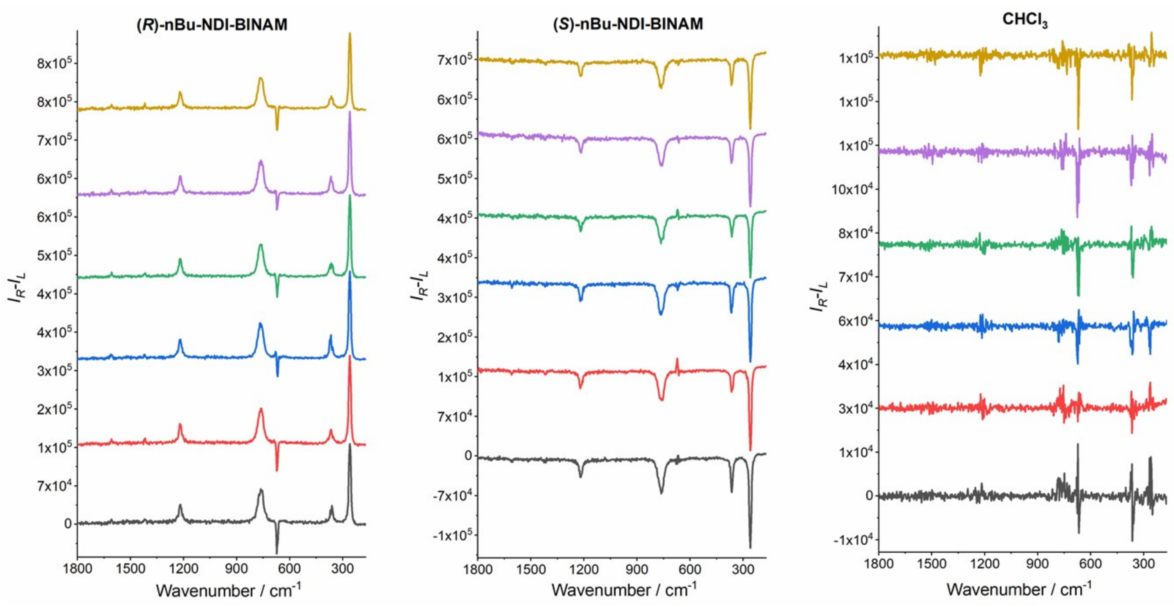

Figure S7. Set of six ROA spectra of $CHCl_3$ and nBu-NDI-BINAM enantiomers dissolved in $CHCl_3$. Each of presented ROA spectra were averaged every 2 hour blocks obtained within the whole 12 hours ROA measurement.

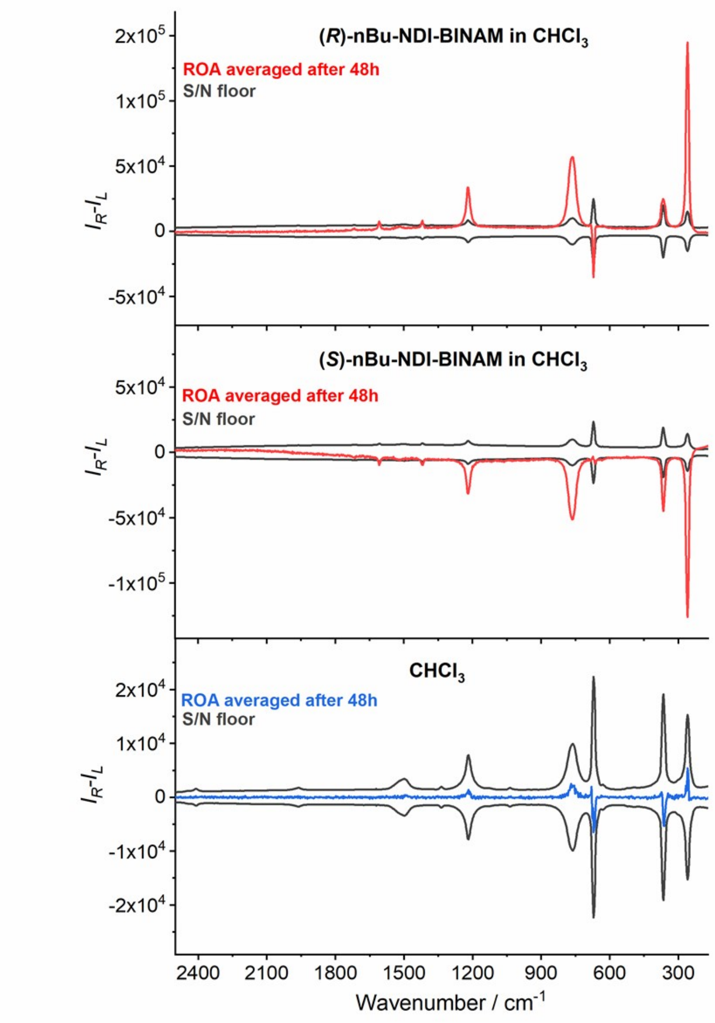

Figure S8. Comparison of shot noise floor (S/N) values and ROA spectra for pure $CHCl_3$ and nBu-NDI-BINAM dissolved in $CHCl_3$. The S/N value was obtained by calculating the square root of the Raman spectrum.

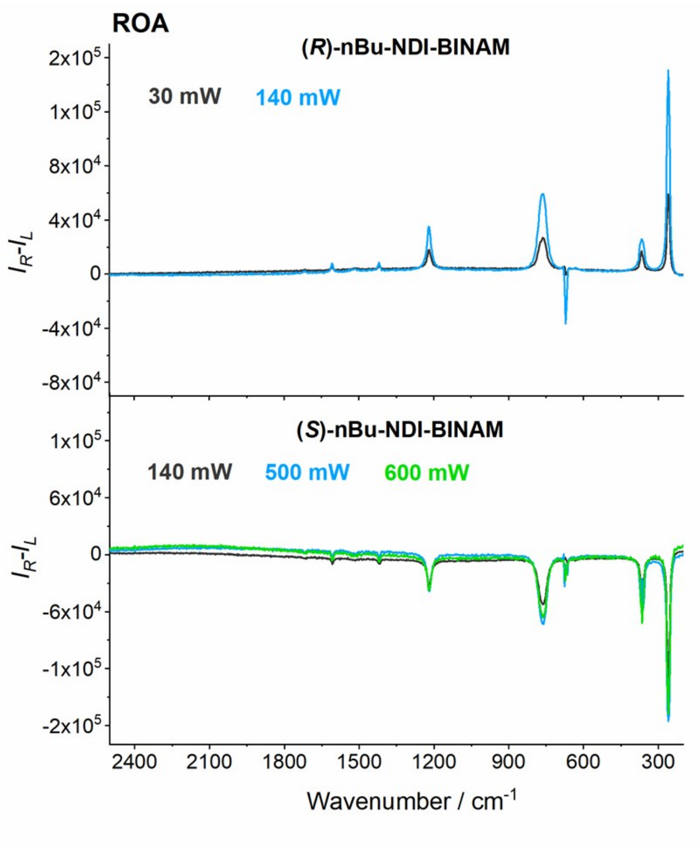

Figure S9. Comparison of ROA spectra for nBu-NDI-BINAM enantiomers in $CHCl_3$ measured at different power of laser. Due to limited stability of (*R*)-nBu-NDI-BINAM under exposure of ROA laser it was impossible to register ROA spectra at higher laser power, in contrast to (*S*)-nBu-NDI-BINAM.

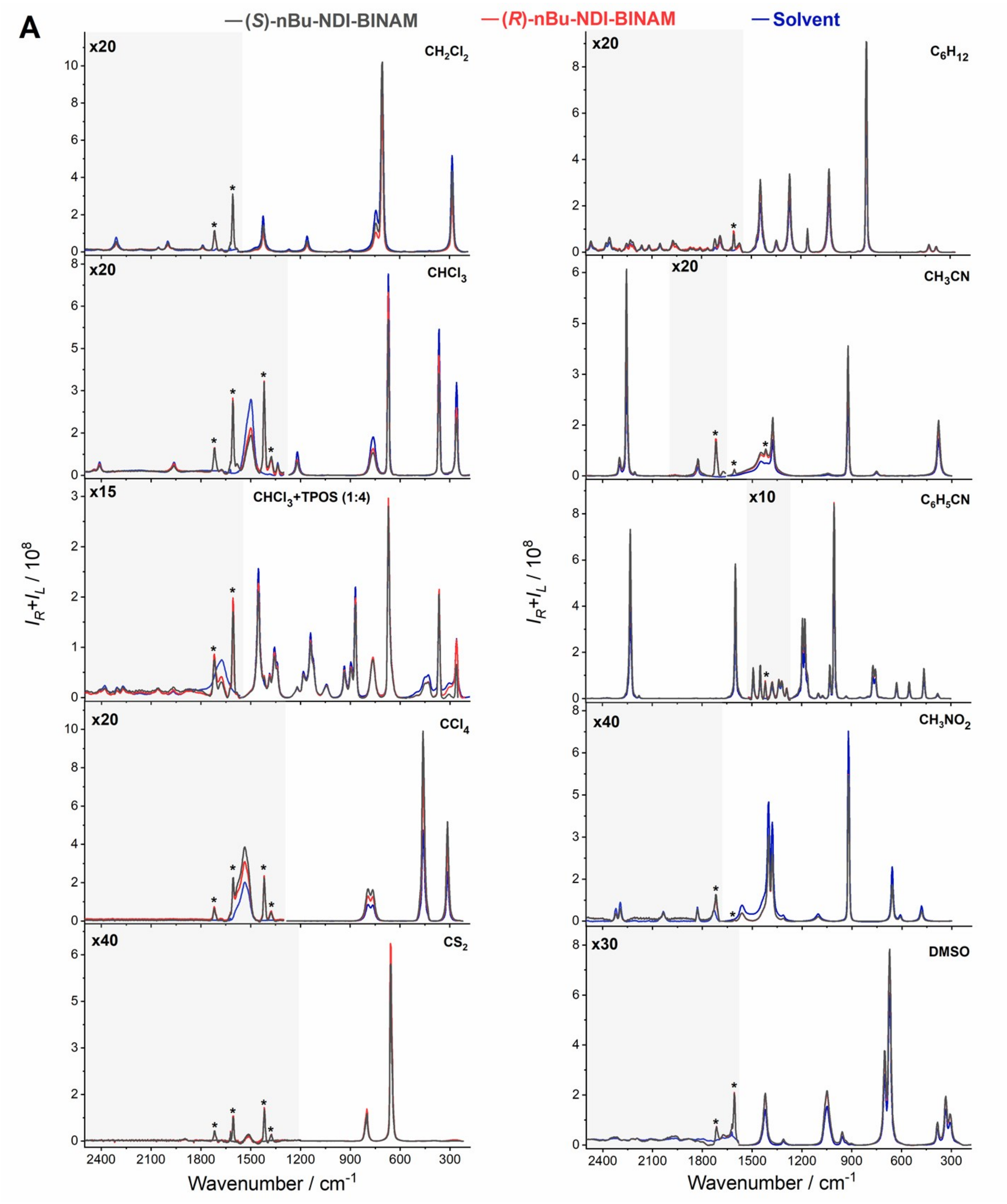
A
—(S)-nBu-NDI-BINAM
—(R)-nBu-NDI-BINAM
—Solvent
x20
CH2Cl2
x20
C6H12
x20
CHCl3
x20
CH3CN
x15
CHCl3+TPOS (1:4)
x10
C6H5CN
x20
CCl4
x40
CH3NO2
x40
CS2
x30
DMSO
IR+IL / 10^8
Wavenumber / cm-1

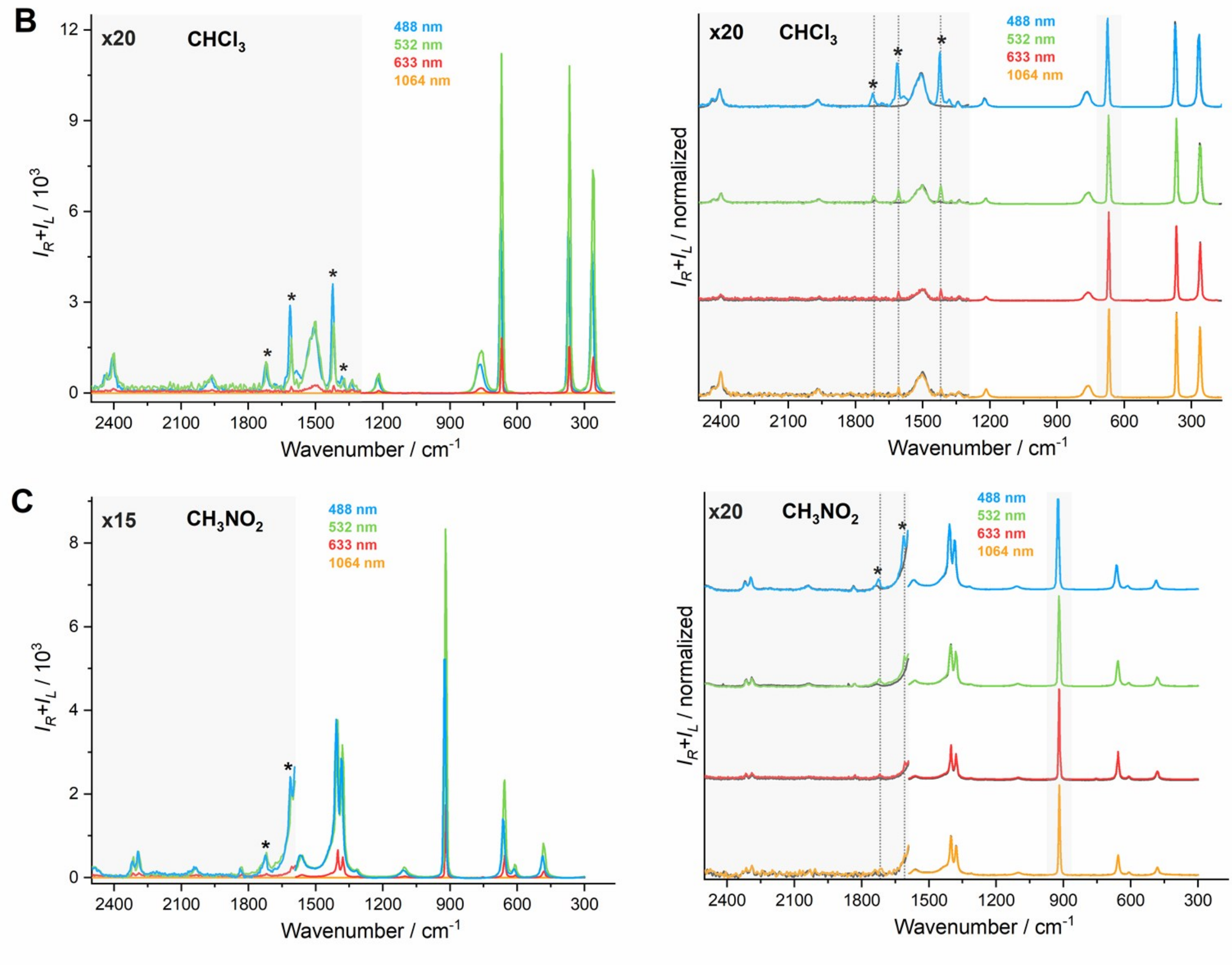

Figure S10. Raman spectra of (*S*)-nBu-NDI-BINAM measured in different achiral solvents **(A)** and in $CHCl_3$ **(B)** and CH_3NO_2 **(C)** solvents at different excitation wavelength – spectra were not normalised (left) or normalized to the most intense solvent band (right). Asterisks represent nBu-NDI-BINAM Raman bands.

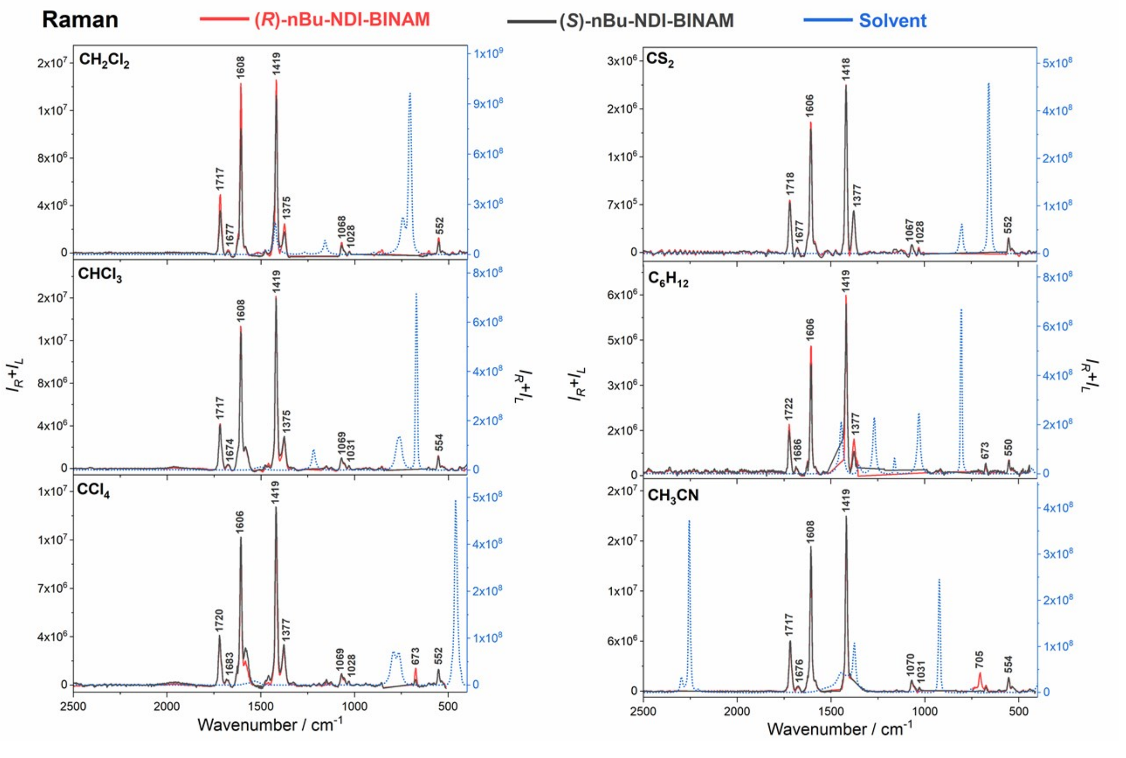

Figure S11. Solvent corrected Raman spectra of nBu-NDI-BINAM.

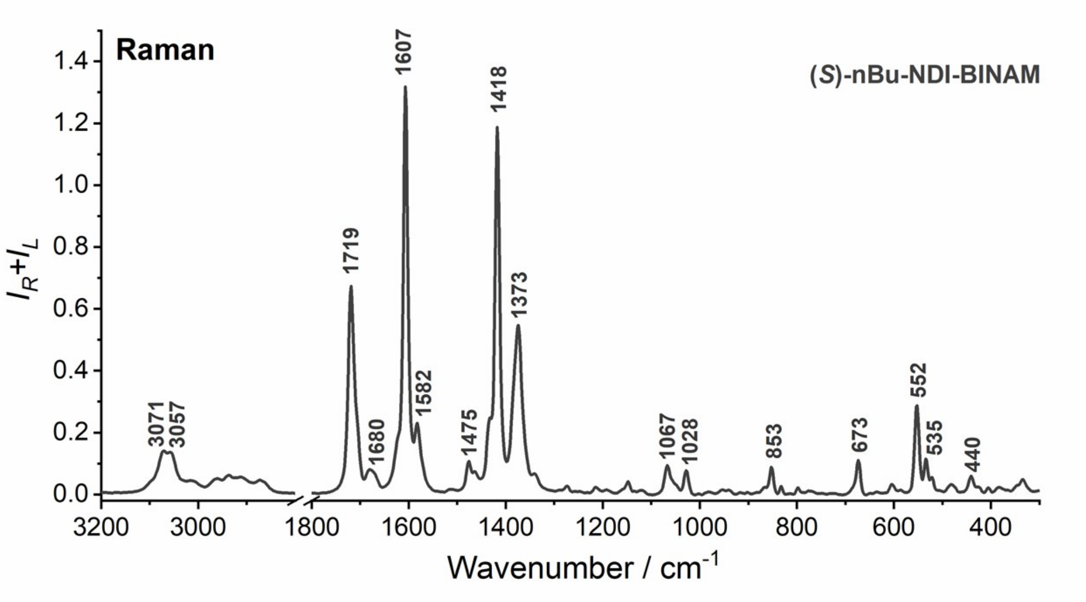

Figure S12. Raman spectra of solid (*S*)-nBu-NDI-BINAM.

Table S2. Assignments of Raman experimental bands of nBu-NDI-BINAM measured in $CHCl_3$ based on the CAM-B3LYP/D3/def2TZVP calculations. NDI, AN and N denotes naphthalenediimide, aminonaphthalene and naphthalene moieties.

**Experimental [cm^{-1}]**	**Calculations [cm^{-1}]**	**Assignments**
1717	1811	ν^s(C=O) symm stretch (NDI)
1674	1770	ν^{as}(C=O) asymm stretch (NDI)
1608	1685	ν_1(C=C) stretch (NDI)
1582	1663	ν(C=C) stretch (AN, N), ν(C-C) stretch (AN-N)
1419	1467	ν_2(C=C) stretch (NDI)
1375	1421	ν_2(C=C) stretch (N)
1069	1105	ν(C-C) stretch (NDI, AN), β(CH) bend (NDI, AN, N), ρ(NH_2) rocking (AN), ν(C-N) (NDI-nBu; NDI-N)
1054	1092	ν(C-C) stretch (nBu), ν(C-C) stretch (AN-N)
554	567	ring breathing (NDI)
536	550	ring deform (AN, N)
440	451	ring deform (AN)

Table S3. List of induced and natural chirality ROA bands of nBu-NDI-BINAM measured in chiral (*R*)-(α)- and (*S*)-(α)-pinene.

ROA band (cm^{-1})	(*S*)-nBu-NDI-BINAM Induced Chirality		(*R*)-nBu-NDI-BINAM Induced Chirality		*α*-pinene Natural Chirality	
	(*R*)-*α*-pinene	(*S*)-*α*-pinene	(*R*)-*α*-pinene	(*S*)-*α*-pinene	*R*	*S*
261	-	-	+	+	not presented	not presented
420	-	-	+	+	+	-
466	not presented	-	+	not presented	+	-
845	-	-	+	+	-	+
997	-	-	+	+	-	+
1066	not presented	-	+	not presented	+	-
1087	-	-	+	+	-	+
1130	not presented	-	+	not presented	+	-
1183	-	-	+	+	-	+
1268	-	-	+	+	-	+
1309	-	-	+	+	+	-
1375	-	-	+	+	-	+

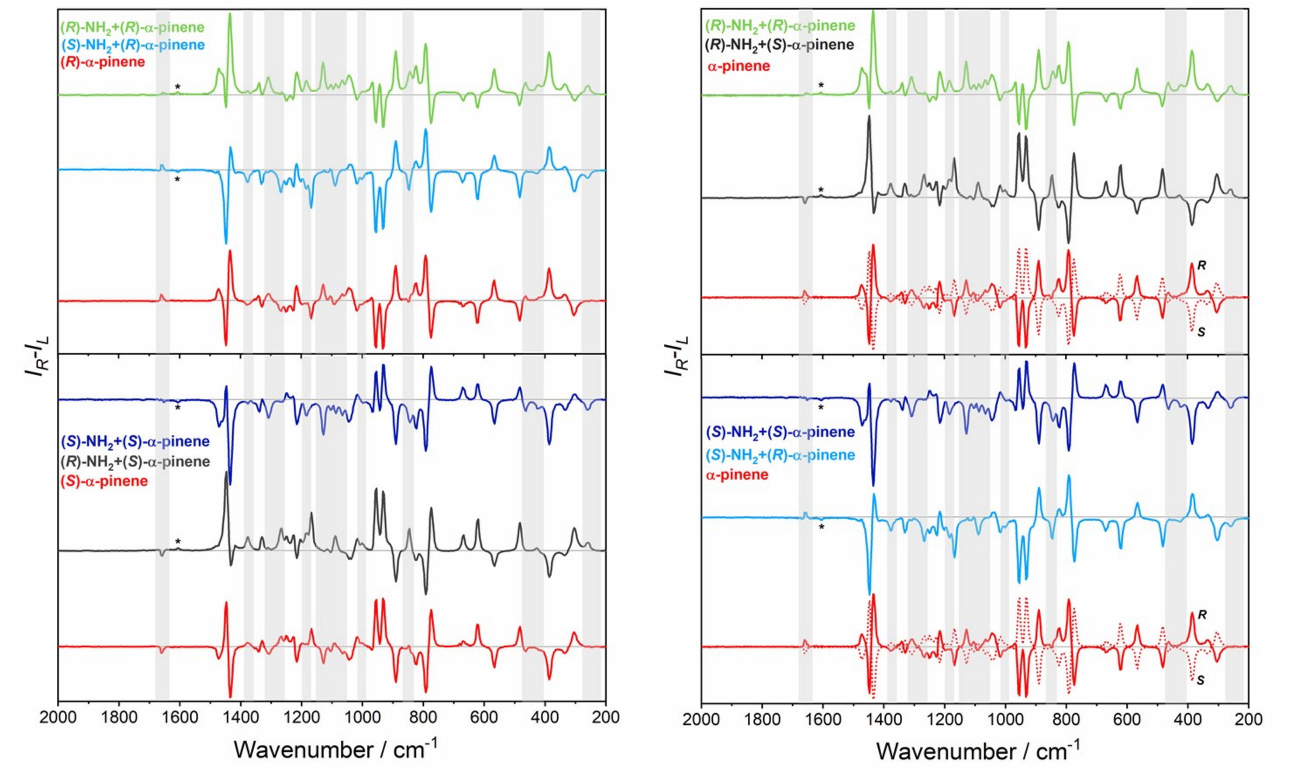

Figure S13. ROA spectra of nBu-NDI-BINAM measured in chiral (*R*)-(α)- and (*S*)-(α)-pinene. The grey belts highlight induced *vs* natural chirality bands. nBu-NDI-BINAM was denoted as (*R*)-NH_2 and (*S*)-NH_2.

3. UV-Vis and ECD measurements

The ECD and UV-Vis spectra of nBu-NDI-BINAM were recorded in the 380-700 nm spectral range at room temperature in spectroscopic grade solvents: CH_2Cl_2, $CHCl_3$, CCl_4, CS_2, C_6H_{12}, CH_3CN, C_6H_5CN, CH_3NO_2, DMSO, 1:4 v/v mixture of $CHCl_3$ and TPOS (tetrapropyl orthosilicate, $C_{12}H_{28}O_4Si$) and chiral (*R*)-α- and (*S*)-α-pinene. The solutions concentrations of *ca.* c_m=2.6·10^{-4} M were measured in the 1 cm quartz cell. The UV-Vis/ECD spectra of nBu-NDI-BINAM were also collected below 380 nm, where the lower limit was determined by the solvents and registrations sometimes required using 0.1 cm and 0.2 cm cuvettes. All spectra were recorded on *Jasco* J−1500 spectropolarimeter with a 100 nm min^{-1} scanning speed, a step size of 0.2 nm, a bandwidth of 1 nm, a response time of 1 s, an accumulation of 5 scans and were background-corrected using the respective solvent recorded under the same conditions.

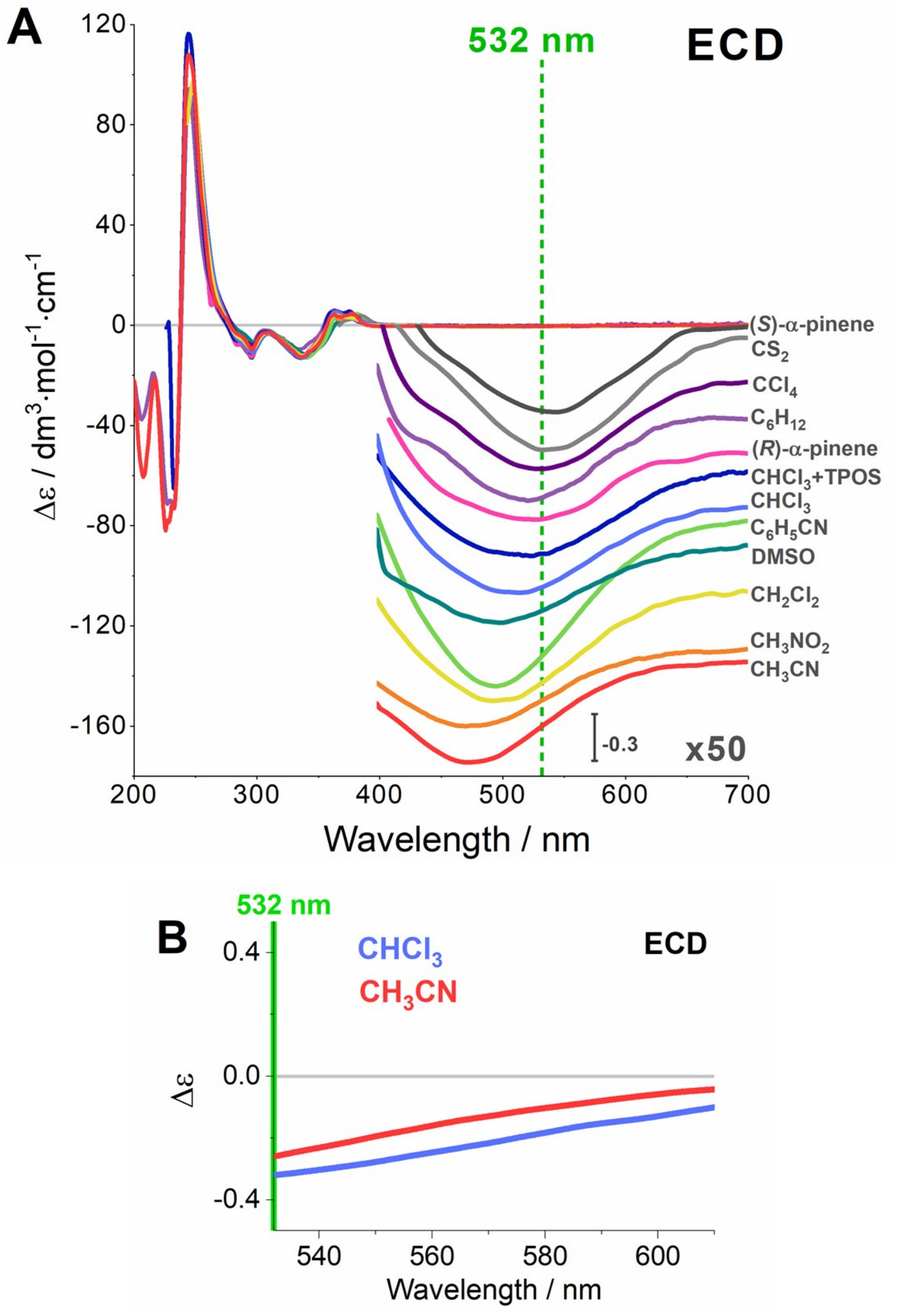

Figure S14. Juxtaposition of the ECD spectra of (*S*)-nBu-NDI-BINAM in different solvents (panel A). The bands at ca. 500 nm are spaced apart for clarity. The green dotted line indicates the excitation wavelength of the ROA laser. ECD spectra of **(*S*)-nBu-NDI-BINAM in $CHCl_3$ and CH_3CN presented in the 532-610 nm range (panel B).**

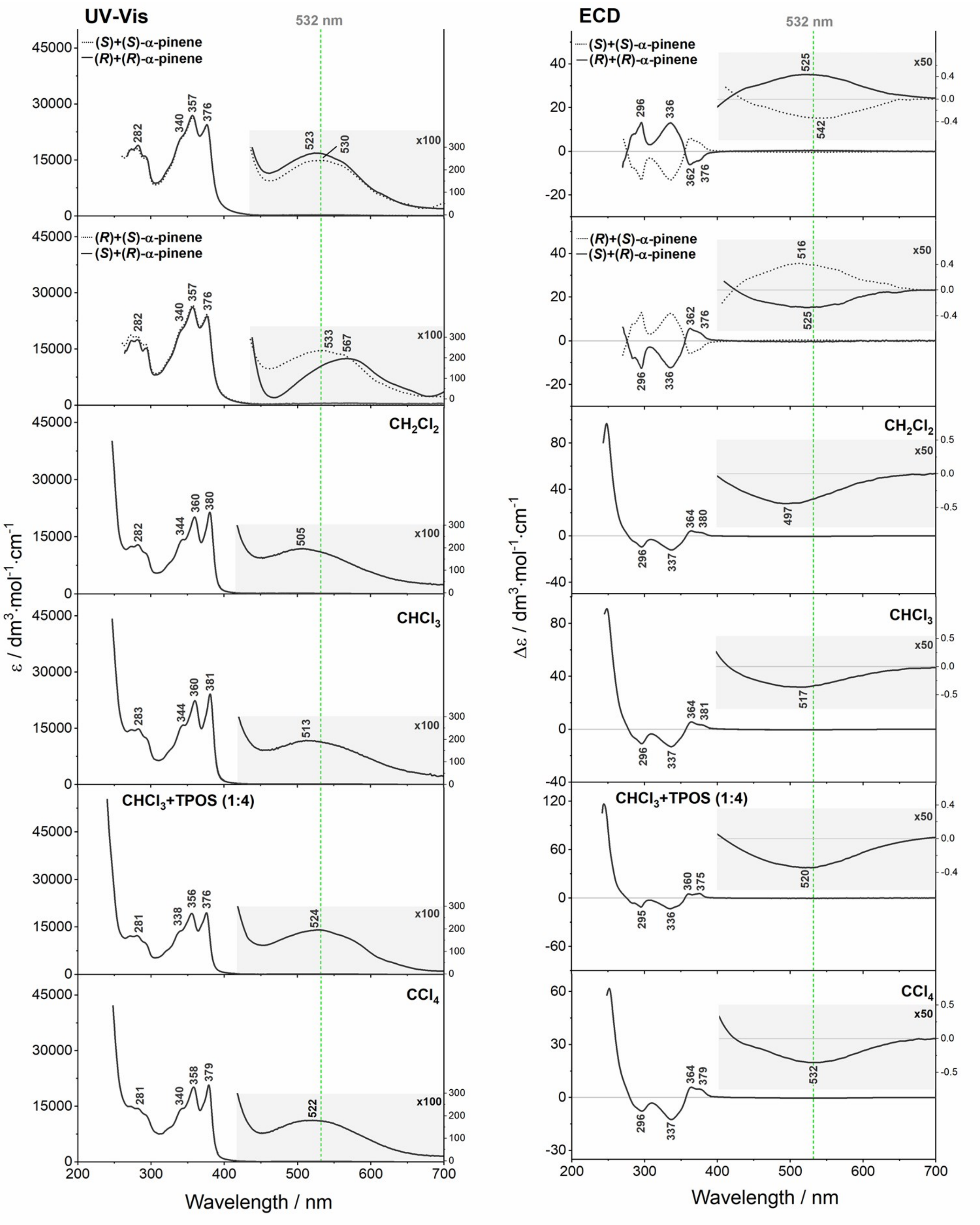
UV-Vis
ECD
532 nm
(S)+(S)-α-pinene
(R)+(R)-α-pinene
(R)+(S)-α-pinene
(S)+(R)-α-pinene
CH2Cl2
CHCl3
CHCl3+TPOS (1:4)
CCl4
x100
x50
ε / dm3·mol-1·cm-1
Δε / dm3·mol-1·cm-1
Wavelength / nm

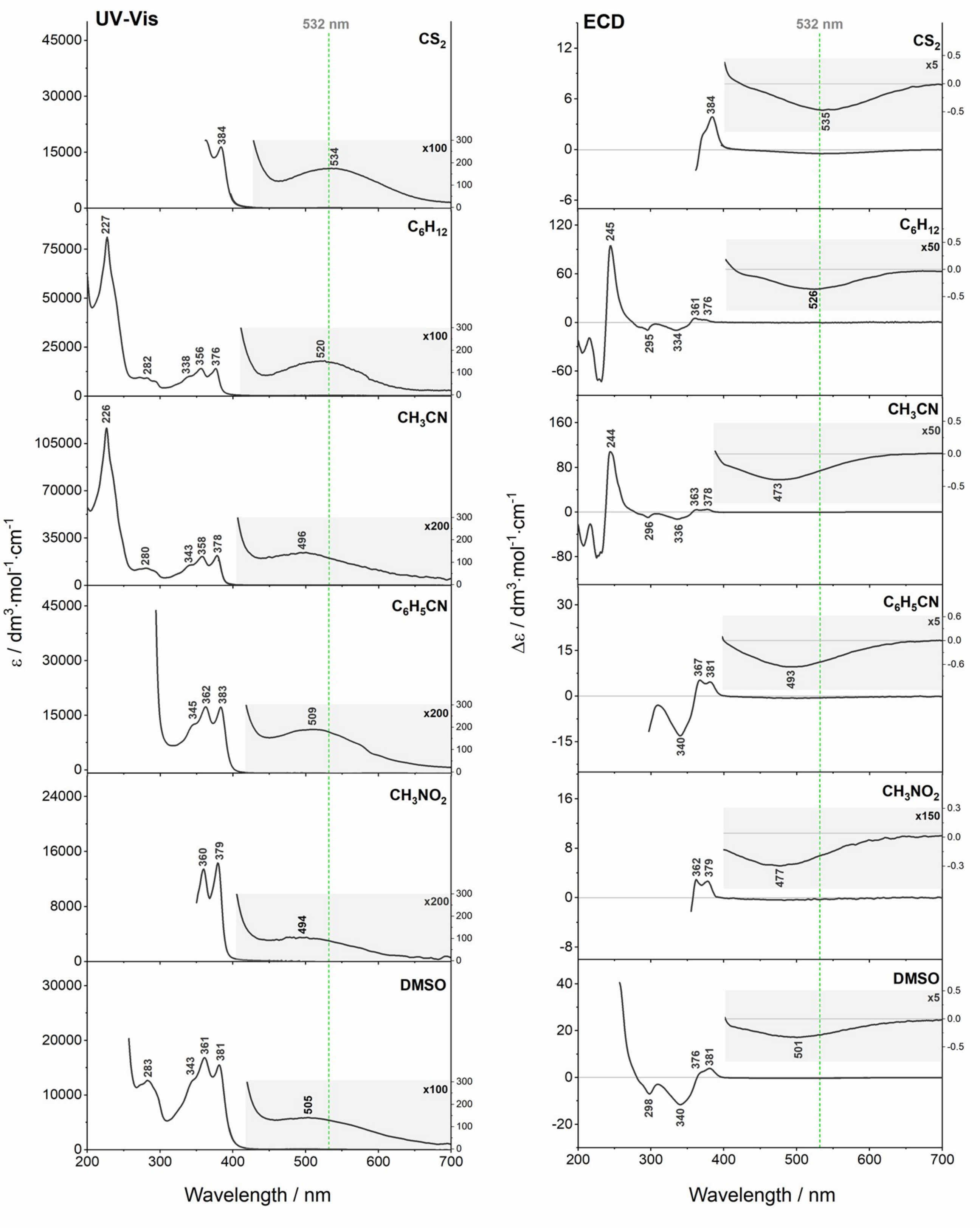

Figure S15. The detailed UV-Vis and ECD spectra of (*S*)- and (*R*)-nBu-NDI-BINAM measured in chiral (*R*)-(α)- and (*S*)-(α)-pinene and of (*S*)-nBu-NDI-BINAM in different achiral solvents. The green dotted line indicates the excitation wavelength of the ROA laser. The spectral range in which the longest wavelength band (LWB) of nBu-NDI-BINAM occurs is enlarged for clarity and is presented in the grey insets. Note that although the intensity of the LWB band in the UV-VIS and ECD spectra is significantly lower than the bands in the 380-340 nm range, its dissymmetry factor g^2 is large (**Table S4**). The phenomenon of the enhanced dissymmetry factor *g* is generally known for Charge Transfer complexes for which magnetic dipole transition moments are relatively large[3–5].

4. Analysis of the UV-Vis/ECD and Raman/ROA data

Table S4. The observed UV-Vis, ECD, Raman, and ROA maxima of (*S*)-nBu-NDI-BINAM and the CID=I_{ROA}/I_R, g=$I_{ECD}/I_{UV\text{-}Vis}$, and κ = g/2·CID ratios.

solvent	UV-Vis/ECD			Raman/ROA			
	λ_{max} (nm)	Assignment	$g \cdot 10^{-4}$	ν_{max} (cm^{-1})	Assignment	$CID \cdot 10^{-4}$	κ
				284	CCl_2 (scissoring) (A_1)	-2.09	6.1
				704	CCl_2 (symm. stretch) (A_1)	-0.01	----
				745	CCl_2 (asymm. stretch) (A_1)	-3.16	4.1
CH_2Cl_2	497	CT	-25.7	899	CH_2 (rocking) (B_1)	-2.57	5.0
	380	NDI	1.1	1160	torsion (A_2)	-3.40	3.8
				1269	CH_2 (wagging) (B_2)	na.	----
				1424	CH_2 (scissoring) (A_1)	-2.26	5.7
				1607	nBu-NDI-BINAM	-2.12	6.1
				260	CCl_2 (antisymm. bend) (E)	-6.31	1.6
				365	CHCl (symm. bend) (E)	-1.10	9.4
				667	CCl_3 (symm. stretch) (A_1)	na.	----
$CHCl_3$	517	CT	-20.7	763	CCl_3 (asymm. stretch) (E)	-5.76	1.8
	381	NDI	1.2	1220	CHCl (antisymm. bend) (E)	-5.07	2.0
				1419	nBu-NDI-BINAM	-2.76	3.8
				1607	nBu-NDI-BINAM	-4.30	2.4
				260	(CH_3Cl) CCl_2 (antisymm. bend) (E)	-3.16	2.2
				366	(CH_3Cl) CHCl (symm. bend) (E)	-1.21	5.8
				429	(TPOS) β(C-C-C) (symm. bend)	-0.39	18.1
				671	(CH_3Cl +TPOS) CCl_3 (symm. stretch) (A_1)+β_2(O-Si-O) (symm. bend)	-0.24	29.4
				764	(CH_3Cl +TPOS) CCl_3 (asymm. stretch) (E)+β_1(O-Si-O) (asymm. bend)	-2.70	2.6
				869	(TPOS) ν^s(O-Si) (symm. stretch)	-0.52	13.6
				897	(TPOS) ν^{as}_2(O-Si) (asymm. stretch)	-2.79	2.5
$CHCl_3$+TPOS	520	CT	-14.1	935	(TPOS) ρ(CH_3) (asymm. bend)	-3.18	2.2
(1:4)	375	NDI	3.2	1044	(TPOS) ν^{as}_1(O-Si) (asymm. stretch)	-1.48	4.8
				1139	(TPOS) ν(C-O)-ρ(C-C) (asymm. stretch)	-2.35	3.0
				1183	(TPOS) β(COSi) (antisymm. bend)	-0.40	17.6
				1221	(CH_3Cl) CHCl (antisymm. bend) (E)	-1.49	4.7
				1357	(TPOS) β(CH) (antisymm. bend)	-1.74	4.1
				1386	(TPOS) ω(CH_3) (symm. bend)	-1.18	6.0
				1454	(TPOS) β(CH_3) (antisymm. bend)	-2.00	3.5
				1607	nBu-NDI-BINAM	-2.90	2.4
				1720	nBu-NDI-BINAM	-6.54	1.1
				219	CCl_2 (symm. bend) (E)	na.	----
				313	CCl_2 (antisymm. bend) (F_2)	-4.15	2.4
				459	ν_1 (symm. stretch) (A_1)	-0.19	----
CCl_4	532	CT	-20.1	765	(ν_3/ν_1 + ν_4) (F_2)	-3.97	2.5
	379	NDI	2.0	792	(ν_3/ν_1 + ν_4) (F_2)	-4.01	2.5
				1418	nBu-NDI-BINAM	-3.73	2.7
				1607	nBu-NDI-BINAM	-3.46	2.9
				400	ν_2 (bend)	na.	----
				658	ν_1 (symm. stretch)	-1.98	6.8
CS_2	535	CT	-27.0	801	2ν_2 (RF bend)	-1.92	7.0
	384	NDI	2.3	1419	nBu-NDI-BINAM	-7.45	1.8
				1605	nBu-NDI-BINAM	-7.94	1.7
				383	CCC (symm. bend) (A_{1g})	na./0	----
				427	CCC (asymm. bend) (E_g)	-1.47	7.9
				785	CH_2 (rocking) (E_g)	na.	----
				806	(ring breath) (A_{1g})	na./0	----
	526	CT	-23.1	1031	CCC (asymm. stretch) (E_g)	-1.71	6.8
C_6H_{12}	376	NDI	2.3	1161	CCC (symm. stretch) (A_{1g})	-0.46	25.1
				1269	CH_2 (wagging) (E_g)	-1.45	8.0
				1349	CH_2 (deform) (E_g)	-1.02	11.3
				1445	CH_2 (scissoring) (E_g)	-1.36	8.5
				1465	CH_2 (scissoring) (A_{1g})	na.	----
				1606	nBu-NDI-BINAM	-6.24	1.9
				307	CSC (bend)	-2.67	4.6
				333	CSO (asymm. bend)	-2.62	4.6
				383	CSO (symm. bend)	-1.43	8.5
				671	CS (symm. stretch)	na./0	----
	501	CT	-24.3	700	CS (asymm. stretch)	-1.90	6.4
DMSO	381	NDI	2.5	957	CH_3 (rocking)	-1.33	9.1
				1047	SO (stretch)	-7.18	1.7
				1311	CH (symm. deform)	na./0	----
				1420	CH_3 (deform)	-1.63	7.4
				1607	nBu-NDI-BINAM	-2.03	6.0
				1714	nBu-NDI-BINAM	-1.14	10.7
				379	CCN (bend) (E)	-3.20	5.5
				924	CC (stretch) (A_1)	0.55	-32.3
CH_3CN	473	CT	-35.5	1378	CH_3 (bend) (A_1)	-1.00	17.8
	378	NDI	2.0	1446	CH_2 (bend) (E)	-1.53	11.6
				2256	CN (stretch) (A_1)	1.38	-12.9
				2300	CH_3 (bend) (A_1)+ CC (stretch) (A_1)	1.21	-14.7
				381	CCN (in plane bend) (B_2)	-5.99	3.6
				462	CC (stretch) + ring (deform) (A_1)	-1.10	19.7
				551	CN (in plane bend) (B_2)	-5.70	3.8
				627	ring (deform) (B_2)	-5.57	3.9
				755	ring (deform) + CH (wagging) (B_1)	na./0	----

				769	ring (deform) + CC (stretch) (A_1)	0.25	-86.8
C_6H_5CN	493	CT	-43.4	930	CH (deform) (B_2)	na./0	----
	381	NDI	2.4	1004	ring (deform) (A_1)	0.56	-38.8
				1030	CC (stretch) (A_1)	0.96	-22.6
				1166	CH (in plane bend) (A_1)	-4.52	4.8
				1181		na./0	----
				1195	CH (in plane bend) + CC (stretch) (A_1)	-0.53	40.9
				1599	CC (stretch) (A_1)	-2.46	8.8
				2233	CN (stretch) (A_1)	na./0	----
				1419	nBu-NDI-BINAM	-2.92	7.4
				482	NO_2 (rock) (B_1)	-2.73	4.7
				610	NO_2 (rock) (B_1)	-1.83	7.0
				658	NO_2 (symm. bend) (A_1)	-0.32	40.1
				922	CN (symm. stretch) (A_1)	0.24	-53.5
CH_3NO_2	477	CT	-25.7	1104	CH_3 (rock) (B_1)	-1.55	8.3
	379	NDI	1.9	1380	NO_2 (symm. stretch) (A_1)	0.44	-29.2
				1402	CH_3 (symm. bend) (A_1)	0.19	-67.6
				1560	NO_2 (asymm. stretch) (B_1)	-1.53	8.4
				1607	nBu-NDI-BINAM	-1.83	7.0
				308	CH_3 (wagging), CC (bend)	-3.65	3.6
				335	ring (deform), CH_2 (rock)	-3.61	3.7
				391	CH_3 (wagging)	-3.44	3.9
				468	ring (deform)	-2.26	5.9
				485	ring (deform)	-2.33	5.7
				568	ring (deform)	-0.58	22.8
				623	ring breathing	-2.03	6.5
				670	ring breathing	0.33	-40.1
				775	ring (deform)	-0.37	35.8
				845	ring (deform), CH (bend)	-2.34	5.7
	542	CT	-26.5	909	ring breathing	-0.48	27.6
(*S*)-α-pinene	376	NDI	2.4	957	ring (deform), CH_2 (twisting), CH_3 (asymm. deform)	-2.47	5.4
				1046	CH (bend), ring (deform), CH_2 (twisting)	-1.09	12.1
				1089	CH (bend), ring (deform), CH_2 (twisting)	-2.31	5.7
				1129	CH (bend)	-3.57	3.7
				1168	CCH_3 (stretch), CH (bend), CCC (bend), CH_2 (twisting),	-3.95	3.4
				1183	CCC (bend), CH (bend), ring (deform), CH_3 (asymm. deform)	-3.96	3.3
				1223	CH_2 (wagging), CH (bend), CH_2 (twisting)	-1.51	8.8
				1268	CH (bend), CH_2 (twisting)	-2.44	5.4
				1309	CH (bend), CH_2 (wagging)	-3.71	3.6
				1332	CC (stretch), CH (bend), CH_2 (wagging)	-0.86	15.4
				1377	CH_3 (symm. deform)	-2.00	6.6
				1438	CH_2 (scissoring)	-2.56	5.2
				1661	CC (stretch)	0.42	-31.5
				1607	nBu-NDI-BINAM	-4.73	2.8
				308	CH_3 (wagging), CC (bend)	-2.90	2.9
				335	ring (deform), CH_2 (rock)	-2.88	2.9
				391	CH_3 (wagging)	-2.50	3.3
				468	ring (deform)	-2.17	3.8
				485	ring (deform)	-2.42	3.4
				568	ring (deform)	-1.06	7.8
				623	ring breathing	-2.12	3.9
				670	ring breathing	-0.26	31.9
				775	ring (deform)	-0.50	16.6
				845	ring (deform), CH (bend)	-1.99	4.2
	525	CT	-16.6	909	ring breathing	-0.80	10.4
(*R*)-α-pinene	376	NDI	2.4	957	ring (deform), CH_2 (twisting), CH_3 (asymm. deform)	-2.05	4.0
				1046	CH (bend), ring (deform), CH_2 (twisting)	-1.02	8.1
				1089	CH (bend), ring (deform), CH_2 (twisting)	-1.84	4.5
				1129	CH (bend)	-3.56	2.3
				1168	CCH_3 (stretch), CH (bend), CCC (bend), CH_2 (twisting),	-3.14	2.6
				1183	CCC (bend), CH (bend), ring (deform), CH_3 (asymm. deform)	-3.21	2.6
				1223	CH_2 (wagging), CH (bend), CH_2 (twisting)	-1.80	4.6
				1268	CH (bend), CH_2 (twisting)	-2.41	3.4
				1309	CH (bend), CH_2 (wagging)	-2.70	3.1
				1332	CC (stretch), CH (bend), CH_2 (wagging)	-1.39	6.0
				1377	CH_3 (symm. deform)	-2.21	3.8
				1438	CH_2 (scissoring)	-2.81	3.0
				1661	CC (stretch)	na./0	----
				1607	nBu-NDI-BINAM	-6.24	1.3

5. Measurements of chiral BINAM

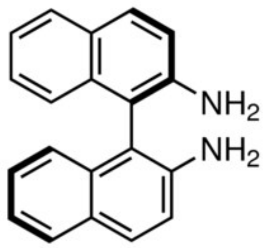

Structure of (*R*)-(+)-1,1′-Binaphthyl-2,2′-diamine ((*R*)-BINAM)

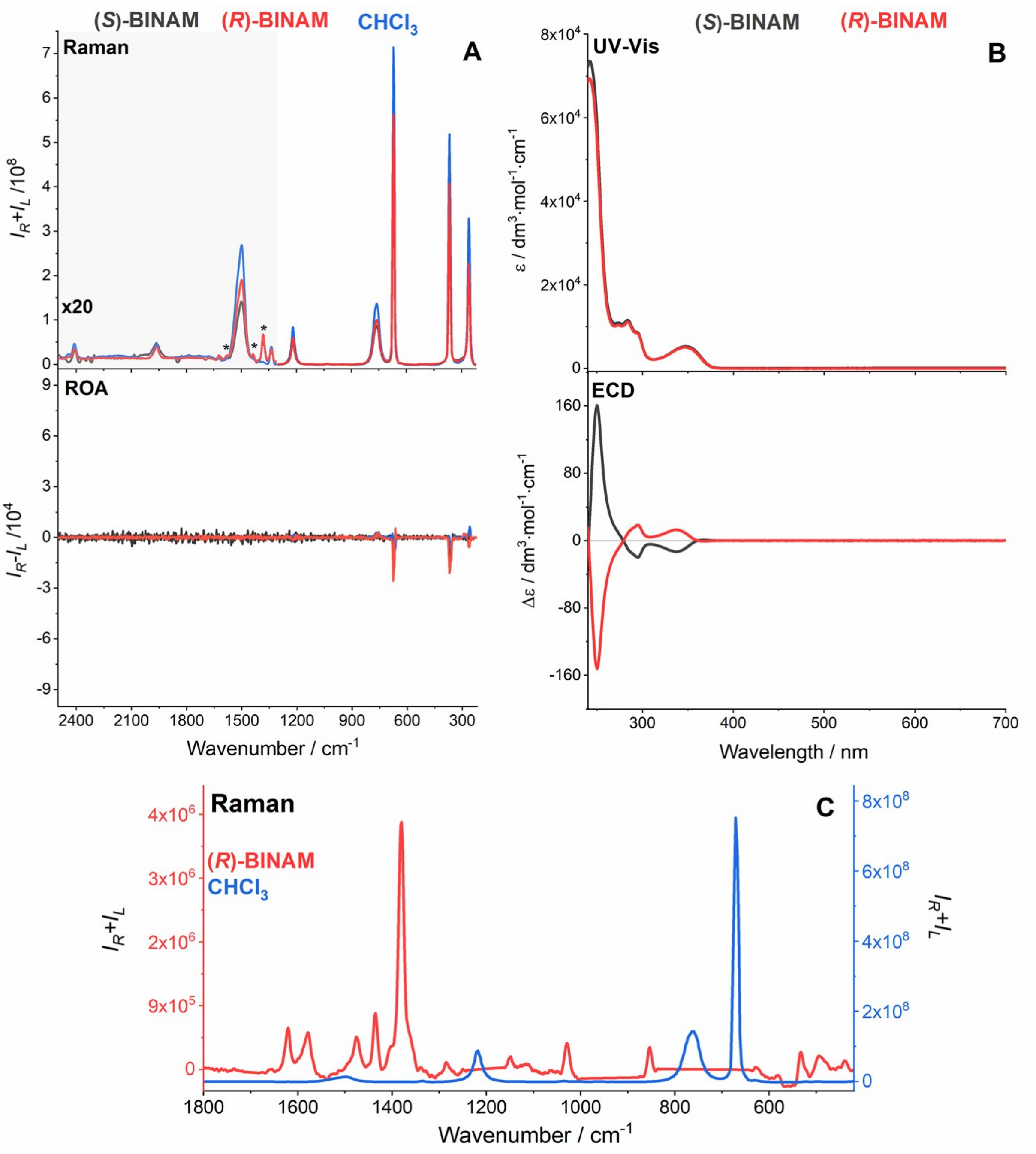

Figure S16. The UV-Vis, ECD, Raman and ROA spectra of BINAM which is a chiral part of nBu-NDI-BINAM enantiomers. The Raman and ROA as well as the UV-Vis and ECD spectra of the BINAM enantiomers (c=3x10^{-3} mol/dm^3, integration time of 2.8665s, 24 hours acquisition time and laser power of 34 mW) measured in chloroform are presented in panel **A** and **B**, respectively. Panel **C** shows the solvent corrected Raman spectra of the (*R*)-BINAM enantiomer. Asterisks represent the Raman bands of BINAM.

6. Resonance Energy Transfer

In nBu-NDI-BINAM, NDI is poor in π-electrons, naphthalene (N) and AN are rich in them, and nBu with no π-electrons plays a solubilizer role. The π-electron donating ability of the NH_2 substituent[6], causes that AN is a stronger π-electron donor than N. If NDI, N, and AN were free molecules in a solution then N···NDI or AN···NDI charge transfer (donor-acceptor) complexes would be formed. In consequence, a short-range RET from N or AN donors to NDI acceptor would appear. Also, a respective middle- or long-range RET mechanisms could have taken place if the donors and the acceptor were more separated in a sufficient concentration. Thus, short-, middle-, and long-range RET processes could occur altogether. Notice that in the nBu-NDI-BINAM molecule, the AN donor and NDI acceptor moieties are nearby, cannot dissociate, and are specifically arranged. Yet, the N-plane is nearly perpendicular to the NDI and AN ones. Thus, the π-electron delocalization can be only a little extended over the N rings. Still, the N linker is a better π-charge conductor than, e.g., a saturated hydrocarbon one for which the through bond contact between AN and NDI would be very restricted.

The adjacency of the AN donor, NDI acceptor, N coupling moiety, mediating solvent molecule Sol, and surrounding solvent molecules make consideration of all possible RET processes that can occur in diluted nBu-NDI-BINAM solutions very complex (**Figure 3**). Indeed, one has to consider the trough-space energy transfer (TSET) processes, i.e., the near-zone Förster radiationless resonance energy transfer (FRET) governed by the Coulomb (dipole-dipole) coupling decreasing with R^{-6} [7,8]. After excitation of the donor, the energy relaxation to the lower energy electronic state of donor goes through the transport of the energy excess to the acceptor by the emission of virtual photon facilitated by dipole-dipole couplings between the moieties. Notice, that the TSET process in a solution can be strongly perturbed by the surrounding medium. The long-range radiative energy transfer (uncorrelated photon emission at one site followed by its absorption at the other site) also occurs and decreases with R^{-2} [9]. This RET process is affected by the surrounding medium as well. However, a third molecule may additionally mediate the RET process be it strongly bound inside or outside the AN-NDI gap. The third body introduces ca. two dozens of mediating states to be considered in the quantum electrodynamics (QED) description of the process. It also adds the R^{-3} and R^{-1} dependences, and, for some molecular arrangements, it even produces a reduction of the overall rate of energy transfer[10]. An interplay of the relative distance between donor and acceptor, atomic transition frequency, the dimensionality of the system, and the neighboring matter, determine the efficiency of the energy transfer and make the RET coupling much more complicated than says the classical Förster theory[8]. The origin of these complications is deeply rooted in the nature of the virtual photons mediating the energy transfer process[8]. Let us add that in contrast to the semi-classical Förster theory predicting only an R^{-6} relation, the QED theory predicts three distance dependencies: R^{-2}, R^{-4}, and R^{-6}, exemplifying the long-, intermediate- and short-range regimes, respectively[10]. Moreover, a trough-bond energy transfer (TBET) between AN bridged with NDI by N "bond" can also take place. However, in bridged systems such a transfer behaves differently than FRET and can be efficient even without a spectral overlap between the donor and acceptor[11–14]. Furthermore, plasmon-coupled RET expressions developed within the classical electrodynamics formalism provided description which agrees with the QED analysis and predicted that in the presence of gold nanoparticles, a long-range RET can be increased by as much as 10^6-times and the RET rates increase over hundreds instead of tens of nm in the absence of them[10,15].

The QED theory behind all aspects of RET is not yet fully developed[10], and neither are the quantum-chemical computational algorithms which, for example, do not yet allow for direct modelling of the through-bond RET coupling and still include the environment only via the implicit solvent model[15–18]. Nevertheless, we approximately estimated the intramolecular RET in the NDI-BINAM molecule using the accessible routines[16–19] (pages S33-S35).

In 2000, D. L. Andrews formulated the theory of an energy transfer mechanism between three fluorophore sites (fluorophore can here be identified with donor, acceptor, and a solvent molecule) which coupled the fluorescence energy transfer with the Raman emission[20]. This accretive process of resonance energy transfer (ARET) with Raman emission was suggested to be very significant in strongly pumped systems. Andrews predicted the ARET with Raman emission to be effective pooling in multi-fluorophore systems uniquely associated with the Raman mediation and differs from the closely related cooperative pooling mechanism[21,22].

Because the AN→Sol and Sol→NDI RETs are predicted to occur in dissolved nBu-NDI-BINAM systems, the accretive process of resonance energy transfer with Raman emission can partially explain the enhancement of the observed ROA spectra as follows. In nBu-NDI-BINAM dissolved in a solvent, at least one solvent molecule is in the gap between the NDI and AN "covers" which is denoted by Sol@(nBu-NDI-BINAM). The donor AN moiety is excited with the green laser line. In the conventional energy transfer mechanism, the deactivation of AN is accompanied by the simultaneous activation of the NDI acceptor via virtual mediating photon (where "virtual" means that no retardation effects are present[10]). In the accretive RET process, the virtual mediating photon released by the excited donor AN moiety, excites the solvent molecule. In Sol@(nBu-NDI-BINAM) system it is placed between the initial HOMO state localized on AN and the final LUMO state localized on NDI. The electronic states of the solvent molecule are in the far-from-resonance conditions. Therefore the molecule is excited from the ground vibrational state and reaches the solvent virtual electronic states. The solvent molecule is deactivated to the first vibrational state of the ground electronic state in the non-resonance Stokes Raman Scattering. Then, RET from the solvent molecule to the NDI acceptor moiety occurs via virtual mediating photon. The ARET effect can be very strong in the case of Sol@(nBu-NDI-BINAM) while the RET processes to the other solvent molecules can be less effective because outside the AN-NDI gap the solvent molecules experience more thermal collisions. Moreover, the inside of the gap is strongly chiral whereas the outside is less chiral (in the sense of the chirality measures[23–25]). This is why the ROA ARET effect is very strong while the Raman effect may not be so enhanced because mostly classical Raman scattering from the bulk solvent is observed. However, we suggest that ARET explains our observations only partially, because we observe mostly the resonance effect from the solvent, while the Andrews mechanism suggests the non-resonance one.

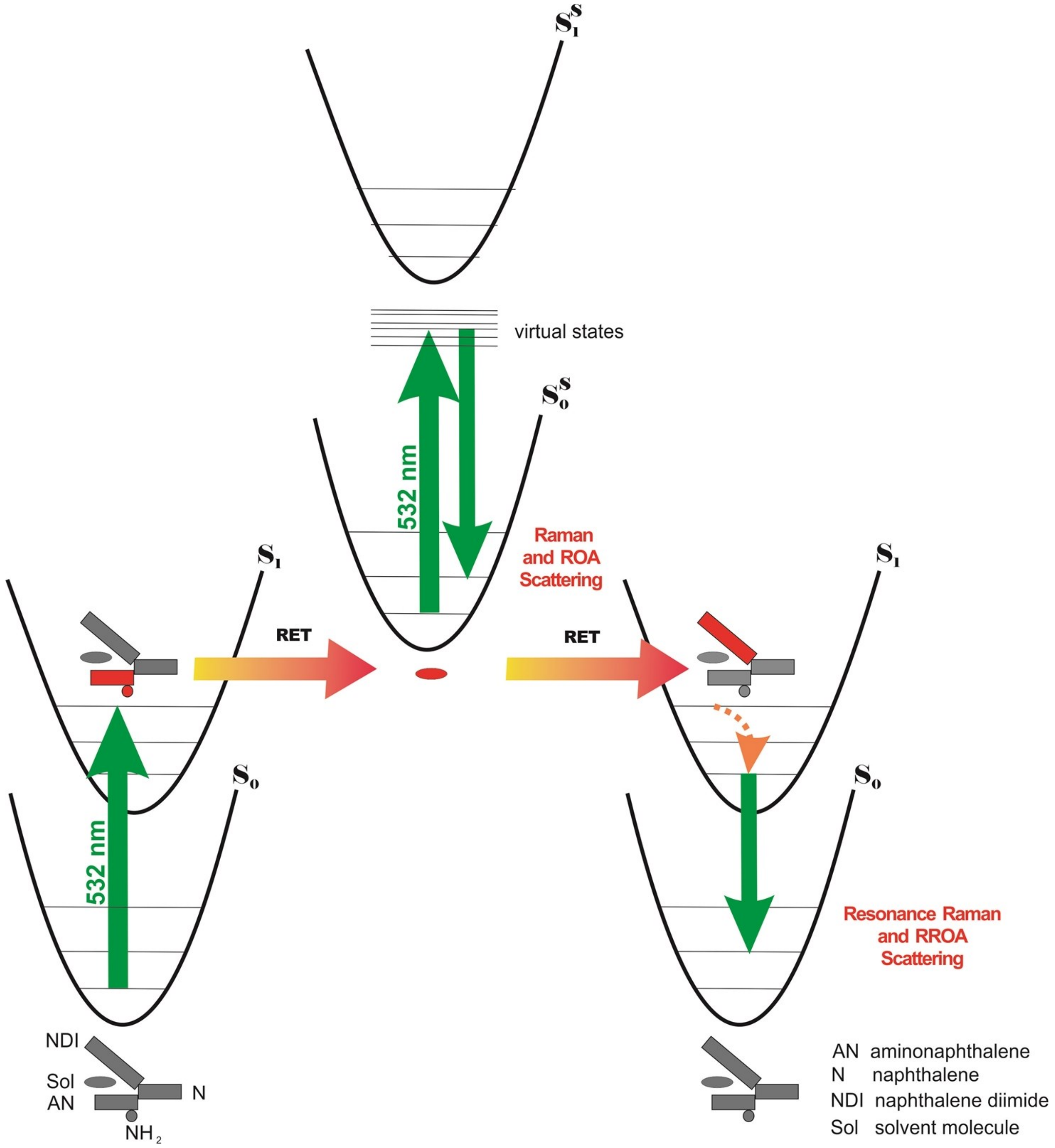

Figure S17. Schematic explanation of the non-resonance Raman scattering in nBu-NDI-BINAM as described by Andrews[20]. The segments in grey denote deactivated moieties while those in red denote the excited ones.

Calculations

General

Geometry optimization

nBu-NDI-BINAM is a quite rigid system stabilized by the NH···O intramolecular (hydrogen-bond-like) distorted contact yielding one preferred arrangement between the binaphtalenylamine (BINAM) group and the NDI core. On the other hand, full conformational analysis of nBu-NDI-BINAM performed by the *Conflex*[26,27] program in which the MMFF94s force field was implemented, provided many conformers as the result of the rotation about the CC single bonds of the nBu chain. All further calculations were performed using the *Gaussian 09* package of programs[28] and the structures were visualized by using *GaussView*[29]. The geometry optimizations were performed using the CAM-B3LYP[30] DFT functional, def2TZVP[31] basis sets, *implicit* PCM[32,33] solvent model and the ultrafine grid. The stationary structures were found by ascertaining that all harmonic frequencies were real. Within the set of 52 conformers localized by *Conflex* only two, exhibiting *all-trans* zig-zag chain (**Scheme S2**), were significant and equally abundant. Next, the two most stable conformers were re-calculated applying the Grimme's D3 dispersion correction[34] to account for interactions between the donor aminonaphtalene (AN) group and the acceptor NDI core. Indeed, the inclusion of the dispersion correction had significant influence on the nBu-NDI-BINAM geometry: the NDI and AN moieties were much closer to each other and thus in the further calculations this correction was always taken into account. Despite the fact that dispersion interactions had a significant influence on the nBu-NDI-BINAM geometry, it did not influence the calculated spectra significantly.

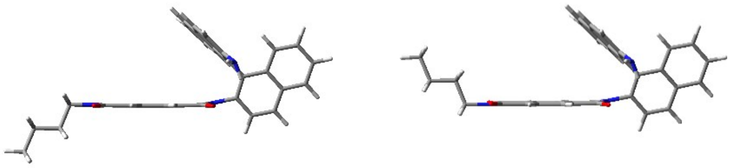

Scheme S2. Two nBu-NDI-BINAM conformers associated with the nBu chain geometry. Only the structure on the left side was used in further calculations.

Energy scans in the ground and excited states

The calculations were performed for model NDI-BINAM system, where the H atom was placed instead of the nBu group, and NDI-BINAM (1:1) complexes with a solvent molecule placed in the AN-NDI cavity. The τ angle defining the relative position of the AN *vs* NDI unit (**Scheme S3**) was changed in steps while the remaining geometrical parameters were relaxed. The calculations were performed at the CAM-B3LYP/tzv[35] level with the opt=modredundant option.

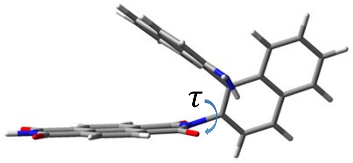

Scheme S3. The dihedral angle *τ* changed in the (*S*)-NDI-BINAM energy scans.

Hybrid models of solvation

In the *implicit* PCM[32,33] solvent model applied initially, the solvent is mimicked by a dielectric continuum with a dielectric constant surrounding a cavity whose shape and dimension is adjusted to the real geometric structure of the solute molecule. However, to better account for the influence of the particular solvents, the *hybrid* model[36,37], a combination of *implicit* and *explicit* supermolecular approaches, was also applied. In this model, a reasonable number of *explicit* solvent molecules (here eleven, or nine for cyclohexane) forms the single shell surrounding the solute and additionally a continuous solvent model (PCM) mimics the next solvation spheres. In such an approach both, short-range and long-range electrostatic corrections to interactions are taken into account. For the hybrid model, the B3LYP-D3/6-31G(d,p)[38] level was applied.

Spectra calculations

The UV-Vis and ECD spectra of nBu-NDI-BINAM were calculated considering the lowest 100 singlet states with the time-dependent DFT (TD-DFT) method[39]. The Coulomb-attenuating CAM-B3LYP[30] functional was applied due to its documented superiority over the B3LYP functional[40] especially for the charge transfer systems. A Gaussian band-shape was applied with the 0.20 eV FWHM.

Interaction energies

The interaction energies of the (1:1) Sol@NDI-BINAM complexes, in which one solvent molecule was placed between the AN and NDI moieties, were corrected for the basis-set superposition error (BSSE) by using the seven-point correction method ΔE_7 which includes the counterpoise method (ΔE_{CP})[41] and the deformation energy of the complex partners[42,43].

Resonance energy transfer

The fundamental theoretical treatment of the resonance energy transfer (RET) was introduced by Förster[44] and in the procedure implemented in *Gaussian16*, the excitation energy transfer rate between molecules (or their parts) is obtained. In our calculations, the (1:1) Sol@NDI-BINAM complexes were divided into three fragments. We considered two models for our systems (**Scheme S4**): 1) in which the naphthalene linking NDI and AN was deleted and the remaining AN, NDI and Sol were in singlet state; 2) the entire system was calculated, but then, the NDI and BINAM fragments were charged to avoid considering energy transfer between doublet states in which the role of the singlet solvent molecule inside the gap would be underestimated. In the EET calculations, the TD-DFT/CAM-B3LYP/D3/6-31g(d,p) level was applied.

Model one in EET calculations		Model two in EET calculations
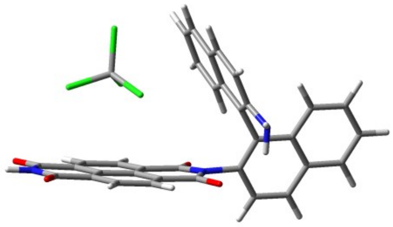	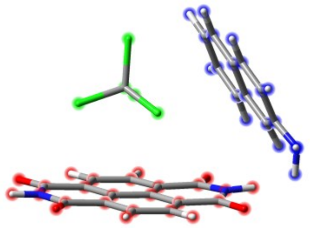	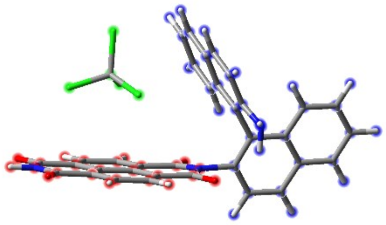
initial system geometry	*Fragment 1 (NDI, charge, multiplicity: 0 1)*<br>*Fragment 2 (solvent Sol, charge, multiplicity: 0 1)*<br>*Fragment 3 (AN, charge, multiplicity: 0 1)*	*Fragment 1 (NDI, charge, multiplicity: +1 1)*<br>*Fragment 2 (solvent Sol, charge, multiplicity: 0 1)*<br>*Fragment 3 (BINAM, charge, multiplicity: -1 1)*

Scheme S4. Two models applied in the EET calculations of (1:1) Sol@NDI-BINAM complexes.

Table S5. The CAM-B3LYP/D3/def2TZVP calculated UV-Vis and ECD spectra of (*S*)-nBu-NDI-BINAM.

The CAM-B3LYP/D3/def2TZVP calculated UV-Vis and ECD spectra of (*S*)-nBu-NDI-BINAM and comparison of the experimental spectra with those calculated with and without the correction for dispersion forces: the excitation energy (λ, nm), oscillator strength corresponding to the electronic excitation of interest (f, dimensionless), Rotatory Strengths (R) in cgs units, the percentage contribution of the given configuration to the resulting excited state transition (the highest values are listed only), 154 and 155 orbitals denote the HOMO and LUMO states.

λ[nm]	f	R	Transition	
460	0.0076	-5.7	154 -> 155	98%
346	0.0003	0.0	153 -> 155	74%
338	0.1557	21.1	152 -> 155	57%
331	0.2974	-10.9	150 -> 155	72%
308	0.0219	-46.3	148 -> 155	59%
307	0.0072	0.6	146 -> 155	45%
305	0.0894	30.8	154 -> 157	39%
298	0.0013	-1.5	144 -> 155	55%
291	0.0052	-13.4	154 -> 159	51%
282	0.0002	-4.2	151 -> 155	27%
281	0.0032	-7.2	142 -> 155	38%
273	0.0043	21.2	154 -> 156	48%
270	0.0076	8.2	140 -> 155	34%
269	0.0346	-28.1	153 -> 161	25%
266	0.0812	14.1	153 -> 157	46%
265	0.0057	-17.5	147 -> 155	32%
263	0.0067	-16.1	154 -> 162	27%
255	0.0152	9.7	149 -> 155	48%
249	0.0010	3.2	154 -> 158	91%
241	0.0063	11.6	149 -> 155	26%
238	0.0355	83.9	154 -> 161	50%
237	0.0021	14.8	150 -> 156	57%
233	0.0309	-84.1	154 -> 160	28%
231	0.0406	-1.6	153 -> 156	42%
228	0.0220	45.0	139 -> 155	45%
227	0.0013	-0.1	152 -> 156	36%
223	0.1521	215.8	153 -> 159	52%
221	0.1277	-23.6	152 -> 157	48%
218	0.0614	389.6	152 -> 159	28%
216	0.0084	10.2	148 -> 156	59%
216	0.0769	-65.9	145 -> 155	33%
215	0.0273	-20.3	145 -> 155	30%
212	0.5150	-572.4	150 -> 158	20%
212	0.3489	-493.2	152 -> 158	45%
210	0.9854	207.0	151 -> 156	37%
209	0.0737	-15.0	141 -> 155	45%
208	0.0738	37.6	154 -> 163	29%
206	0.0306	-40.8	153 -> 158	52%
204	0.4494	297.9	151 -> 157	24%
202	0.0112	-8.8	150 -> 160	37%
201	0.0048	4.8	127 -> 155	57%
201	0.0521	-21.1	144 -> 156	17%
200	0.0361	-33.9	154 -> 167	31%
200	0.0571	84.7	153 -> 160	30%
198	0.0269	-15.4	154 -> 167	20%
198	0.1416	-38.6	146 -> 156	17%
197	0.0222	33.8	152 -> 160	25%
196	0.1146	47.7	146 -> 156	14%
195	0.0287	-78.4	152 -> 160	31%
195	0.0150	-12.8	148 -> 158	40%
194	0.0128	-29.1	153 -> 163	22%
193	0.0239	37.8	142 -> 156	17%
193	0.0736	-71.6	152 -> 161	14%
192	0.0802	27.9	151 -> 161	23%
191	0.0027	-7.0	138 -> 155	77%
191	0.0872	14.7	147 -> 156	16%
190	0.0197	-28.5	152 -> 161	34%
190	0.0323	4.8	153 -> 162	25%
189	0.0845	116.6	151 -> 159	15%

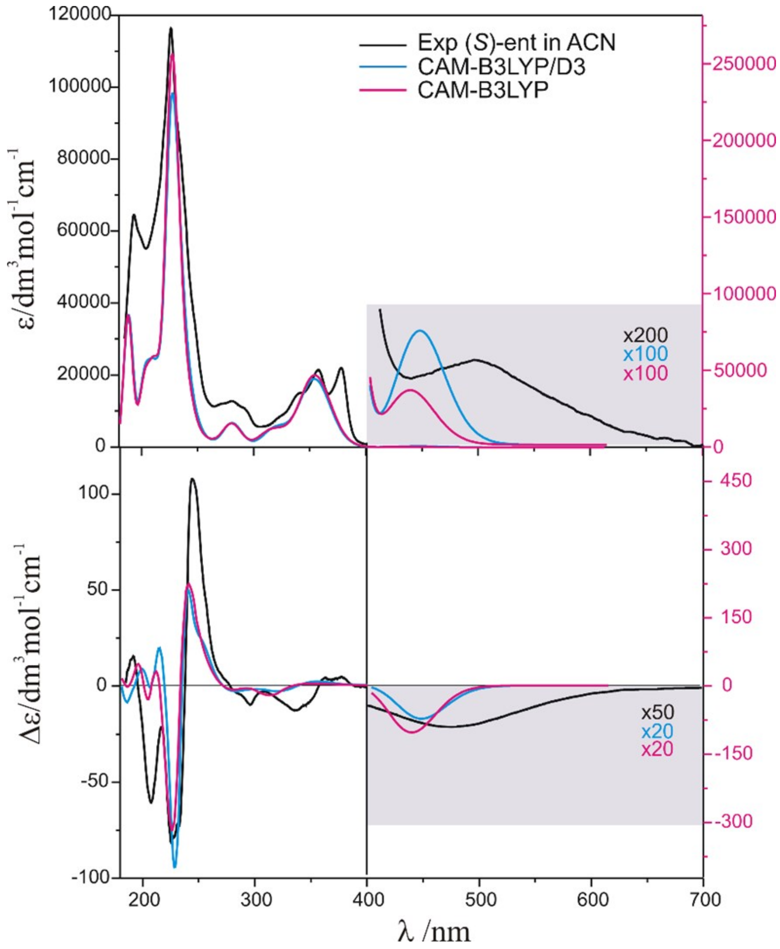

*the calculated spectra were shifted by 12 nm towards higher wavelengths to adjust the position of the most intense UV-Vis bands

Table S6. Influence of the solvent on experimental and calculated longest wavelength band location of (*S*)-nBu-NDI-BINAM.

The experimental positions of the (*S*)-nBu-NDI-BINAM longest wavelength ECD band (LWB) in different solvents and the CAM-B3LYP/D3/PCM calculated LWB positions predicted according to the *implicit* (PCM, def2TZVP) and *explicit* (hybrid, 6-31G(d,p)) solvent model. In the *explicit* model, 11 solvent molecules surrounded the solute (except for cyclohexane where 9 molecules were considered) and additionally the bulk solvent was described by the PCM model (**Figure S19**).

		PCM(def2TZVP)		Hybrid(6-31G(d,p))	
**solvent**	λ_{exp}	$\lambda_{PCM,def2}$	$R_{PCM,def2}$	λ_{hybrid}	R_{hybrid}
vacuum	-	460	-5.683	-	-
CS_2	534	451	-7.277	448	-1.462
CCl_4	532	452	-6.982	912*	-7,612
C_6H_{12}	520	453	-6.817	463	0.017
$CHCl_3$	517	444	-8.137	472	-6.780
DMSO	501	432	-10.506	*na*	*na*
CH_2Cl_2	497	439	-9.033	477	-2.801
C_6H_5CN	493	433	-10.277	*na*	*na*
CH_3NO_2	477	432	-10.316	*na*	*na*
CH_3CN	473	432	-10.246	464	-7.949

*The HOMO-1 (located at the naphtalene moiety -> LUMO transition is predicted for 11 CCl_4 molecules around nBu-NDI-BINAM, *na* – not available

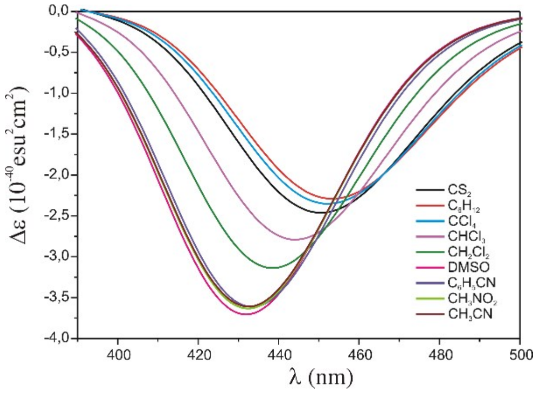

(a)

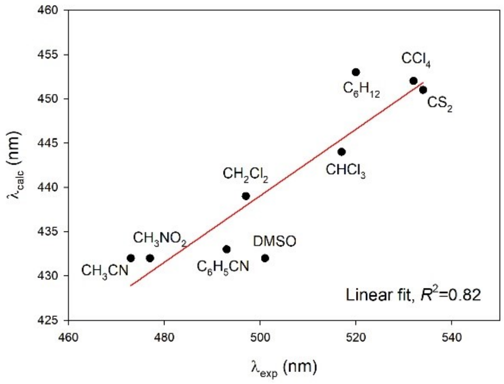

(b)

Figure S18. The calculated position of the charge-transfer LWB band of (S)-nBu-NDI-BINAM in different solvents and the linear correlation between experimental and computational position of the longest wavelength absorption band.

(a) The ECD spectrum in the 350-500 nm range corresponds to the HOMO-LUMO transition (the CAM-B3LYP/D3/IEFPCM/Def2TZVP level).
(b) The linear correlation between the experimental and computational LWB position.

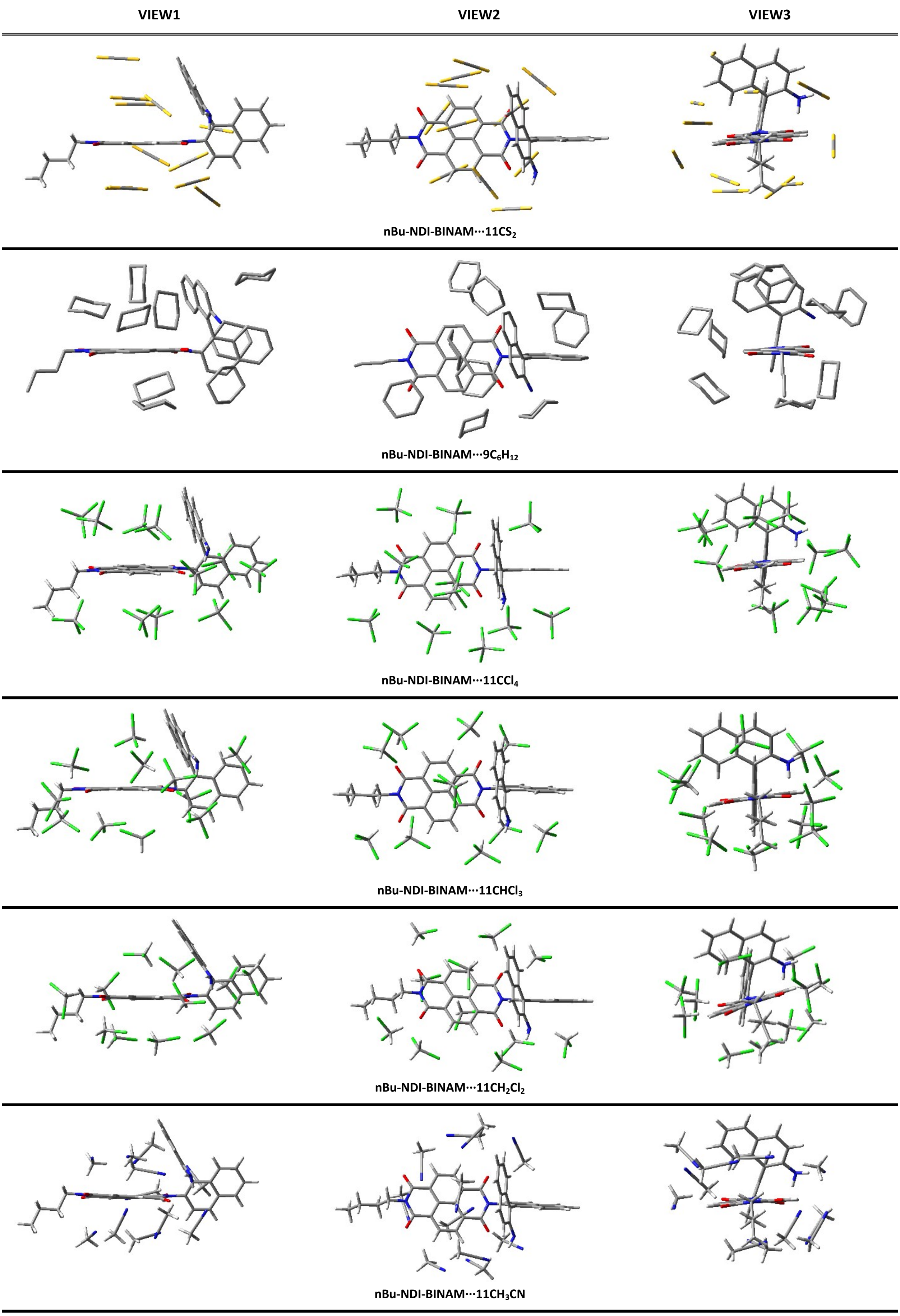

Figure S19. The most stable systems of nBu-NDI-BINAM surrounded by 11 solvent molecules (CS_2, CCl_4, $CHCl_3$, CHC_2Cl_2, CH_3CN) or 9 (C_6H_{12}) obtained at the CAM-B3LYP/D3/6-31G(d,p)/PCM level. In the nBu-NDI-BINAM···9C_6H_{12} system the hydrogens were omitted for clarity. The calculations show that, in most cases, there is only one solvent molecule in the AN-NDI cavity.

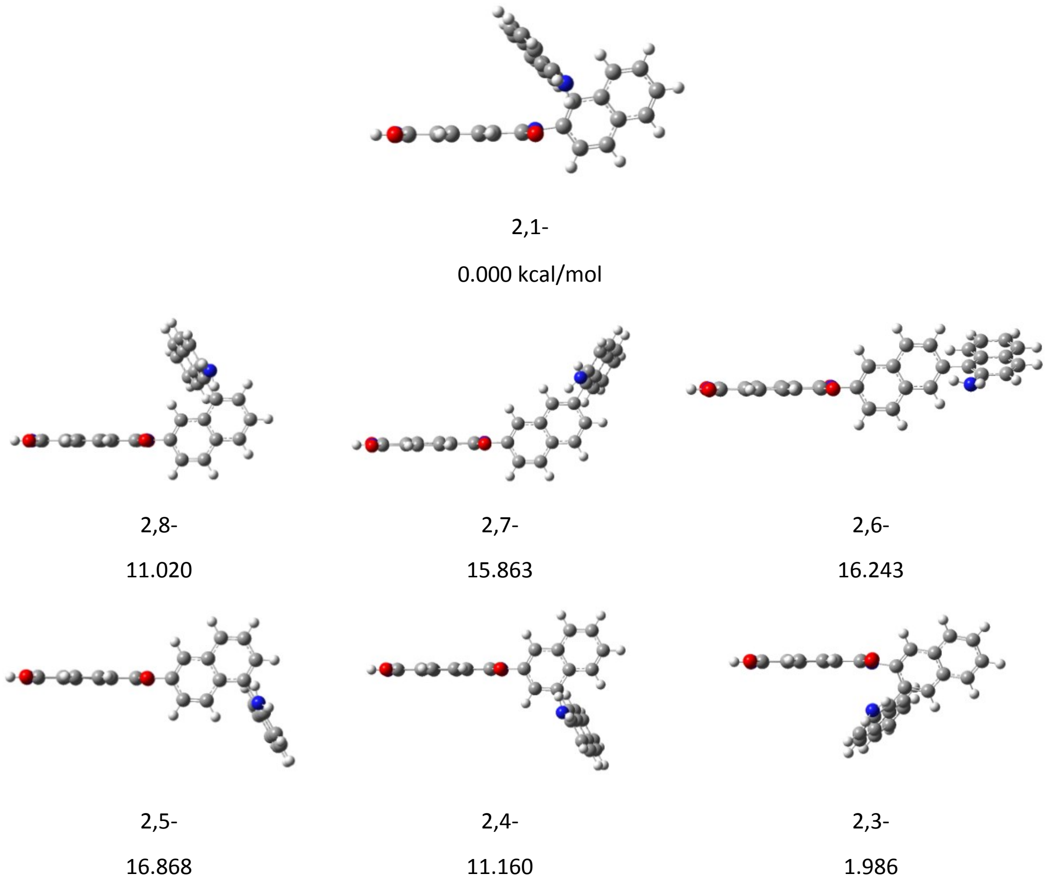

Figure S20. Structures of the NDI-BINAM analogs with the aminonaphthalene group attached in different positions of the linking naphthalene moiety.

Calculated structures and total energy differences between the NDI-BINAM analogs in which the aminonaphthalene moiety is attached in subsequent positions of the linking naphthalene. The calculations were performed at the CAM-B3LYP/D3/def2TZVP level.

Calculations of a series of naphthalenes disubstituted with NDI (always in position 2) and aminonaphthalene (AN) in any other position allows one to show both: the origin of LWB at ca. 500 nm and to estimate the extra stabilization energy appearing when the NDI and AN moieties are neighboring. As it was expected, LWB at ca. 500 nm is present only when the NDI and AN moieties are neighboring in positions 2 and 1 or 2 and 3 and the HOMO-LUMO gap is the smallest. The extra stabilization energy of the NDI and AN interaction was estimated based on homodesmotic isomerization reaction of the 2,5-isomer towards the 1,2-one. Notice that position 5 in naphthalene is symmetrical to 1 and for these two isomers the intramolecular interactions between AN and the naphthalene core are nearly the same. Thus, in the first approximation, the only energetic difference between these two isomers is due to the intramolecular interactions between NDI and AN in the studied 1,2-isomer and their absence in the 2,5-one. The stabilization energy predicted at the CAM-B3LYP/D3/def2TZVP level for molecules without the nBu chain attached to the N-atom of NDI is large and equals 16.9 kcal/mol (**Figure S20**).

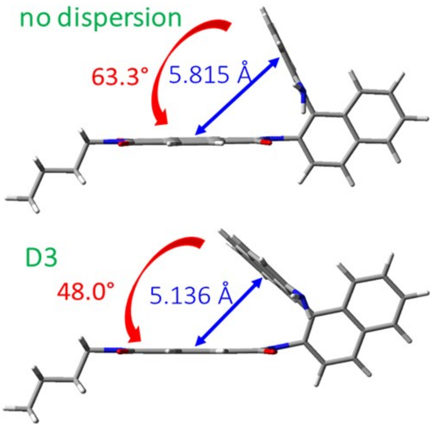

Figure S21. The CAM-B3LYP/def2TZVP calculated nBu-NDI-BINAM structure with and without correction for dispersion forces.

Table S7. Energetics of the (1:1) complexes of (S)-NDI-BINAM with different solvents located in the NDI-AN cavity.

The CAM-B3LYP/D3/631G(d,p) energies and crucial structural parameters of the (1:1) complexes formed between NDI-BINAM and the solvent incorporated between the NDI and AN plates in different arrangements, Sol@NDI-BINAM (**Figure S22**): referred to the most stable form (ΔE, kcal/mol), population of the complex (pop,%), interaction energies corrected for basis set superposition error (BSSE) in the Boys-Bernardi counterpoise procedure (ΔE_{CP2}, kcal/mol), interaction energies corrected for BSSE and monomers deformations upon complexation (ΔE_7, kcal/mol), BSSE (kcal/mol) and deformation energies (ΔE_{def}, kcal/mol). twist angle (τ, deg, see **Scheme S3**) and distance (d, Å) between NDI and AN moieties measured between the ring centers.

	E	ΔE	pop	ΔE_{CP2}	ΔE_7	BSSE	ΔE_{def}	τ	d
**CCl_4@NDI-BINAM**									
ccl4-a1	-3650.382410	0.0	100	-8.35	-7.78	3.52	0.56	-77.4	5.62
**$CHCl_3$@NDI-BINAM**									
chlo-a1	-3190.805868	0.00	93.99	-9.97	-8.76	2.87	1.21	-79.0	5.98
chlo-a2	-3190.803045	1.77	4.71	-7.80	-7.23	2.62	0.56	-77.2	
chlo-a3	-3190.801826	2.54	1.29	-6.63	-6.47	2.62	0.16	-75.8	
**CH_2Cl_2@NDI-BINAM**									
dcm-a1	-2731.220182	0.00	42.67	-8.63	-8.25	2.69	0.38	-76,2	5.47
dcm-a2	-2731.219738	0.28	26.65	-8.09	-7.57	2.57	0.52	-76.3	5.62
dcm-a3	-2731.219218	0.60	15.35	-8.84	-7.69	2.65	1.15	-85,0	6.01
dcm-a4	-2731.219217	0.61	15.34	-8.09	-7.57	2.76	0.52	-68.7	
**CS_2@NDI-BINAM**									
cs2-a1	-2605.989191	0.00	50.96	-5.81	-5.31	2.67	0.50	-77.8	5.66
cs2-a2	-2605.989099	0.06	46.22	-5.99	-5.72	2.21	0.28	-76.4	5.53
cs2-a3	-2605.986463	1.71	2.82	-4.52	-4.33	1.94	0.19	-69.4	
**C_6H_{12}@NDI-BINAM**									
cyhex-a1	-2007.304395	0.00	77.63	-8.94	-8.35	3.38	0.59	-78.2	5.66
cyhex-a2	-2007.302427	1.23	9.66	-8.08	-7.42	3.00	0.67	-79.2	
cyhex-a3	-2007.302320	1.30	8.62	-7.39	-7.18	3.20	0.21	-72.6	
cyhex-a4	-2007.301617	1.74	4.09	-7.77	-6.91	3.06	0.86	-80,1	
**DMSO@NDI-BINAM**									
dmso-a1	-2324.670476	0.00	69.70	-13.51	-12.37	5.65	1.14	-75.9	5.58
dmso-a2	-2324.668230	1.41	6.46	-12.39	-9.62	6.99	2.76	-91,9	
dmso-a3	-2324.668142	1.46	5.88	-13.73	-10.39	6.17	3.34	-107.6	
dmso-a4	-2324.667979	1.57	4.95	-12.38	-10.94	5.51	1.44	-79.2	
dmso-a5	-2324.667913	1.61	4.62	-12.55	-11.52	4.91	1.03	-75.3	
dmso-a6	-2324.667530	1.85	3.08	-11.81	-10.69	5.52	1.13	-74.0	
dmso-a7	-2324.667304	1.99	2.42	-12.74	-11.30	4.75	1.45	-76.1	
dmso-a8	-2324.667257	2.02	2.30	-12.08	-9.00	7.03	3.07	-90,0	
dmso-a9	-2324.665799	2.93	0.49	-10.18	-9.54	5.56	0.64	-69.5	
dmso-a10	-2324.664080	4.01	0.08	-9.50	-8.79	5.23	0.71	-79.4	
dmso-a11	-2324.662315	5.12	0.01	-10.30	-9.31	3.61	0.99	-80.4	
**CH_3CN@NDI-BINAM**									
acn-a1	-1904.233882	0.00	49.6	-9.52	-7.08	3.88	2.44	-94.4	6.49
acn-a2	-1904.233263	0.39	25.8	-8.07	-7.36	3.21	0.71	-78.4	5.70
acn-a3	-1904.233034	0.53	20.2	-8.15	-7.54	2.88	0.61	-78.8	5.63
acn-a4	-1904.231147	1.72	2.7	-6.90	-6.17	3.07	0.73	-78.2	
acn-a5	-1904.230659	2.02	1.6	-6.77	-6.43	2.50	0.34	-76.5	
**C_6H_5CN@NDI-BINAM**									
becn-a1	-2095.879777	0.00	95.27	-14.41	-12.63	6.00	1.78	-88.9	5.87
becn-a2	-2095.876628	1.98	3.38	-12.49	-11.62	5.03	0.87	-76.2	
becn-a3	-2095.874854	3.09	0.52	-11.12	-10.23	5.31	0.89	-79.9	
becn-a4	-2095.874454	3.34	0.34	-13.48	-10.49	4.80	2.99	-92.2	
becn-a5	-2095.874259	3.46	0.27	-11.76	-10.34	4.83	1.42	-80.6	5.87
becn-a6	-2095.874058	3.59	0.22	-11.45	-10.29	4.75	1.16	-77.6	
**CH_3NO_2@NDI-BINAM**									
meno2-a1	-2016.471180	0.00	40.19	-10.54	-8.33	5.88	2.21	-89.9	6.33
meno2-a2	-2016.470707	0.30	24.37	-10.69	-8.38	5.54	2.30	-94.4	6.40
meno2-a3	-2016.470453	0.46	18.62	-9.82	-8.48	5.26	1.34	-77.1	
meno2-a4	-2016.469879	0.82	10.14	-9.47	-8.61	4.77	0.87	-75.2	
meno2-a5	-2016.468711	1.55	2.94	-9.80	-8.41	4.26	1.39	-82.3	6.11
meno2-a6	-2016.468070	1.95	1.49	-9.59	-7.45	4.81	2.14	-102.2	
meno2-a7	-2016.467809	2.11	1.13	-7.70	-5.92	6.19	1.77	-81.9	
meno2-a8	-2016.467361	2.40	0.70	-9.91	-7.82	4.00	2.08	-86.0	
meno2-a9	-2016.466855	2.71	0.41	-7.76	-7.13	4.36	0.63	-86.0	
**(*S*)-(α)-pinene@NDI-BINAM**									
pin-a1	-2162.013543	0.00	33.91	-10.82	-10.24	4.68	0.58	-77.8	5.69
pin-a2	-2162.013309	0.15	26.47	-10.67	-10.08	4.68	0.58	-80.5	5.53
pin-a3	-2162.013046	0.31	20.03	-10.85	-9.83	4.72	1.02	-82.0	5.87
pin-a4	-2162.012648	0.56	13.15	-9.96	-9.68	4.64	0.28	-73.0	5.21
pin-a5	-2162.011192	1.47	2.81	-10.27	-9.09	4.35	1.18	-78.5	
pin-a6	-2162.010484	1.92	1.33	-10.31	-8.70	4.25	1.61	-88.0	
pin-a7	-2162.010479	1.92	1.32	-9.13	-8.64	4.29	0.49	-73.9	
pin-a8	-2162.009947	2.26	0.75	-9.16	-8.53	4.10	0.63	-80.4	
pin-a9	-2162.008049	3.45	0.10	-8.74	-7.95	3.50	0.79	-78.1	
pin-a10	-2162.007908	3.54	0.09	-7.70	-7.47	3.40	0.23	-72.7	
pin-a11	-2162.007181	3.99	0.04	-7.94	-7.43	3.94	0.51	-73.4	

VIEW1	VIEW2

CCl_4@NDI-BINAM

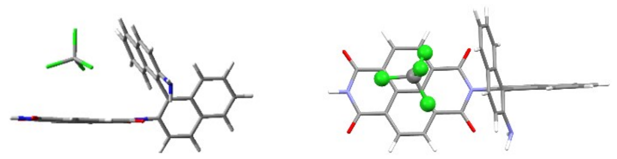

ccl4-a1

$CHCl_3$@NDI-BINAM

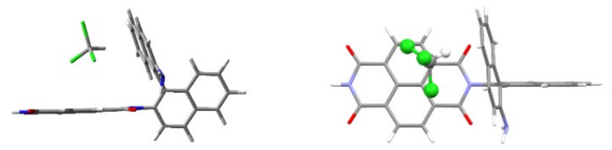

chlo-a1

CH_2Cl_2@NDI-BINAM

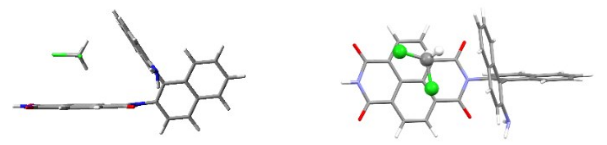

dcm-a1

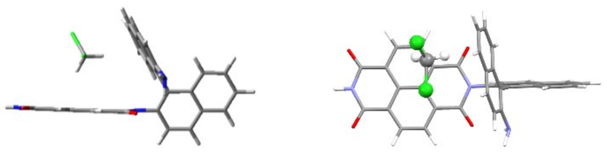

dcm-a2

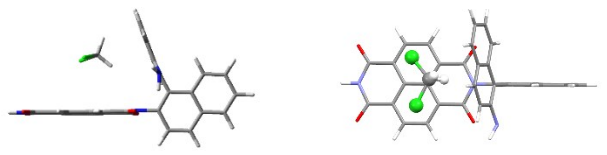

dcm-a3

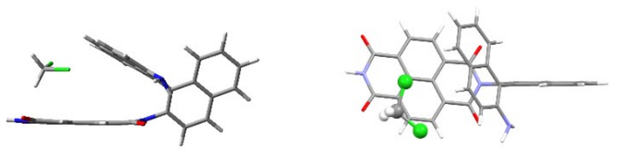

dcm-a4

CS_2@NDI-BINAM

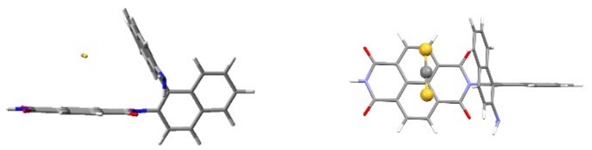

cs2-a1

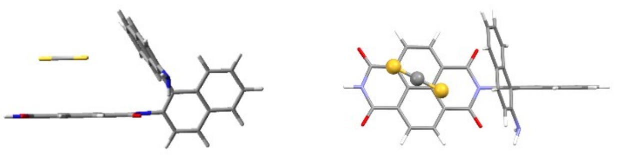

cs2-a2

C_6H_{12}@NDI-BINAM

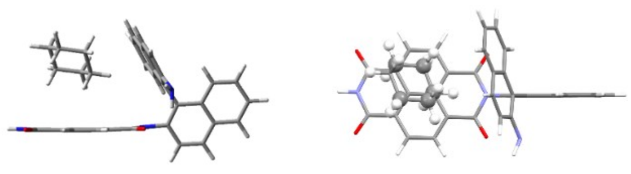

cyhex-a1 cyhex-a1

DMSO@NDI-BINAM

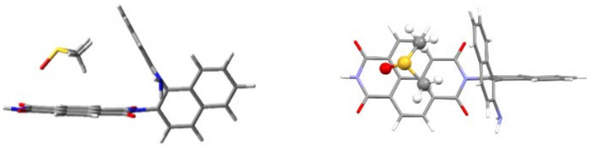

dmso-a1 dmso-a1

CH_3CN@NDI-BINAM

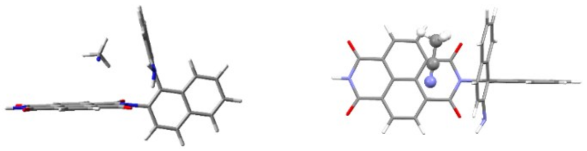

acn-a1

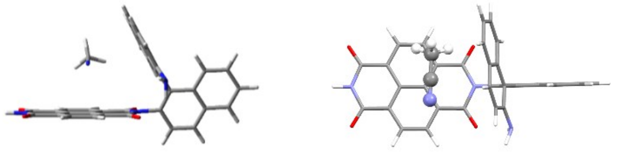

acn-a2

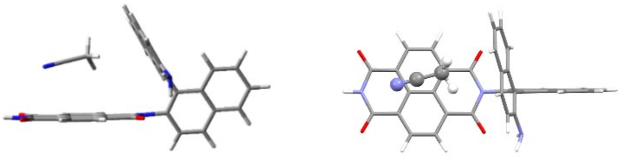

acn-a3

C_6H_5CN@NDI-BINAM

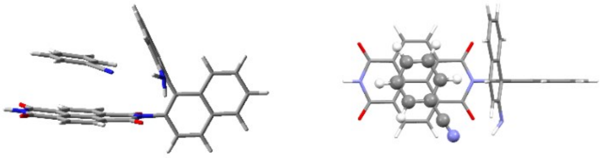

becn-a1

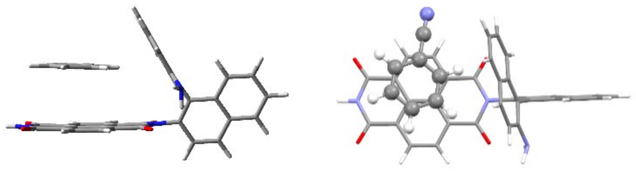

becn-a2

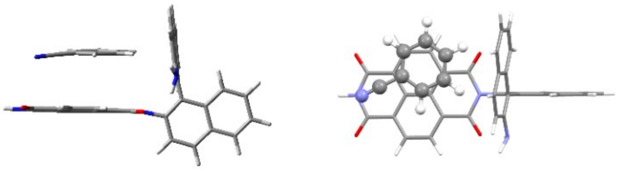

becn-a4

CH_3NO_2@NDI-BINAM

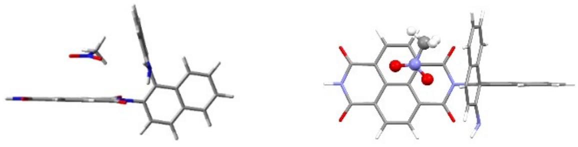

meno2-a1

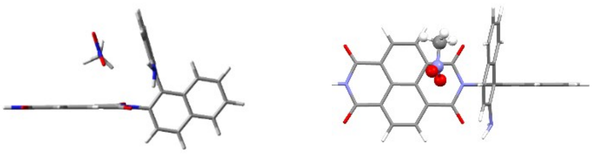

meno2-a2

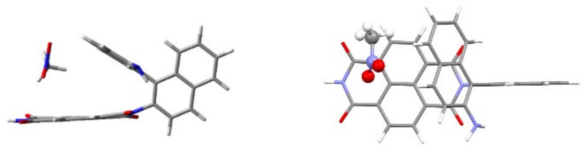

meno2-a3

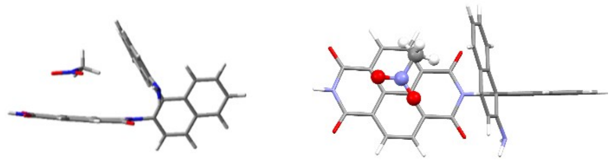

meno2-a4

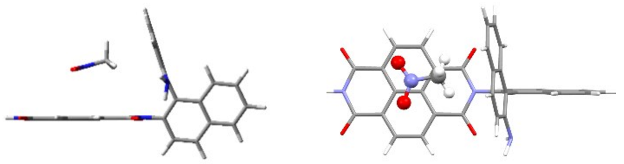

meno2-a5

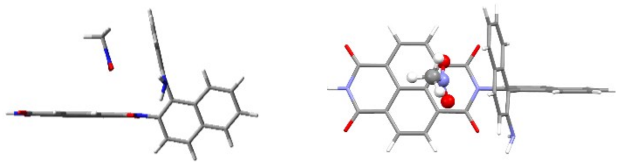

meno2-a7

(*S*)-α-pinene@NDI-BINAM

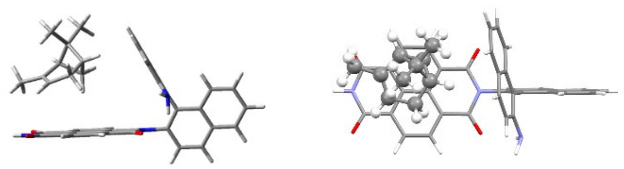

pin-a1 pin-a1

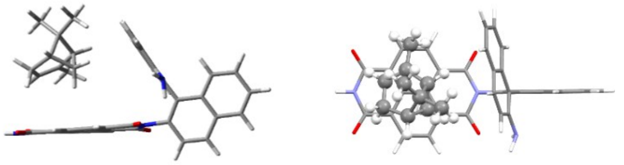

pin-a2 pin-a2

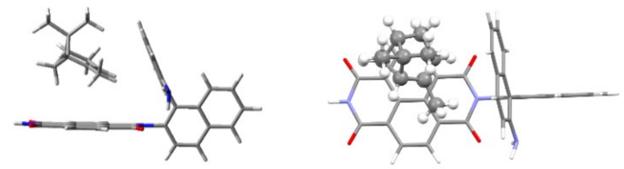

pin-a3 pin-a3

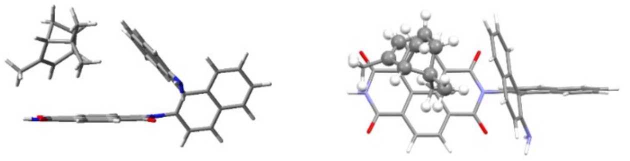

pin-a4 pin-a4

Figure S22. CAM-B3LYP/D3/6-31G(d,p) structures of the most stable (1:1) complexes of (*S*)-NDI-BINAM with different solvents located in the NDI and AN cavity, Sol@NDI-BINAM.

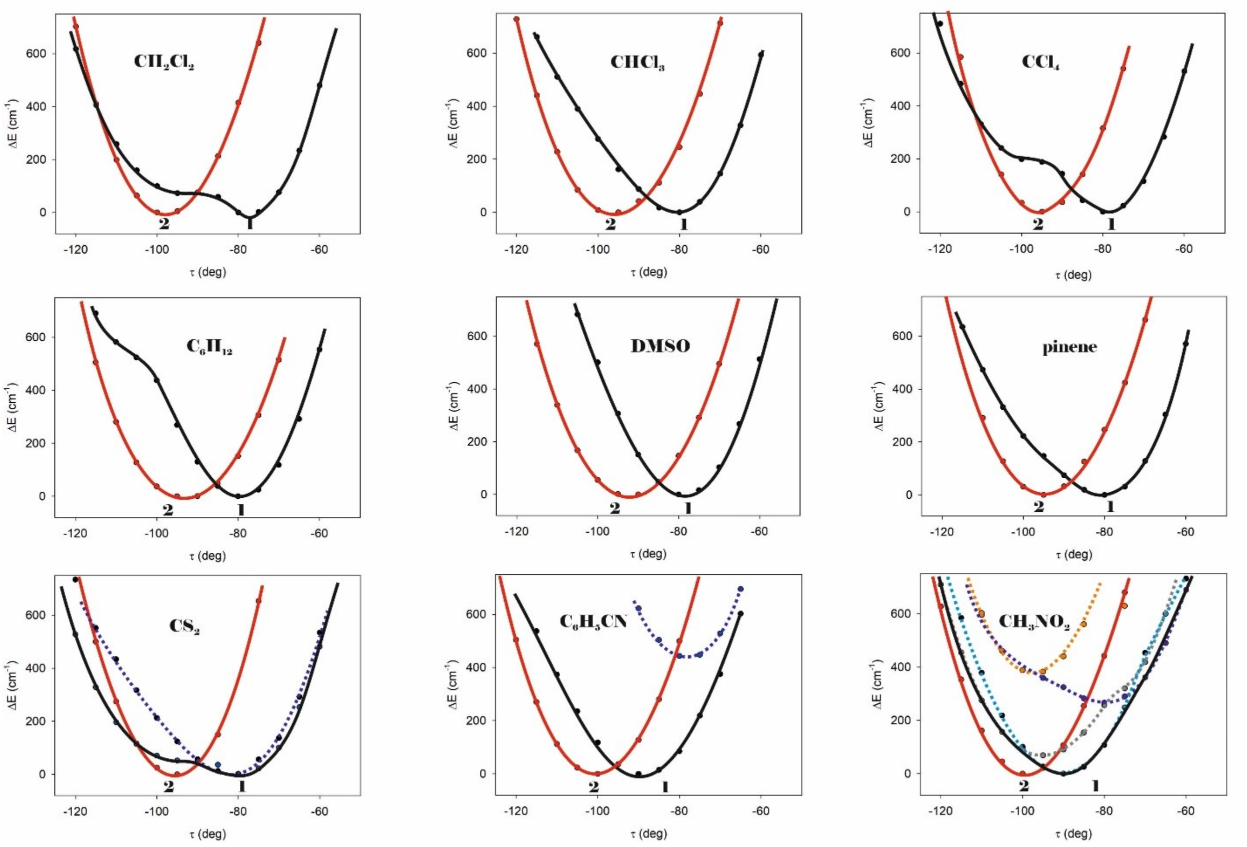

Figure S23. The calculated ground state (black) and the first excited singlet state (red) potential energy profiles of the Sol@NDI-BINAM complexes.

(TD)-CAM-B3LYP/D3/TZV calculated ground state (black) and the first excited singlet state (red) potential energy profiles against the naphthalene linker twist τ angle for the most stable (1:1) complexes of (*S*)-NDI-BINAM with solvent molecules trapped in the gap between AN and NDI planes. The excited states are placed ca. 40 kcal/mol above the ground one but to highlight the minimum positions corresponding to the conformer **2**, the curves were shifted to the ground state level. For CS_2, C_6H_5CN and CH_3NO_2 solvents different positions of the solvent molecule in the gap were obtained.

Comments: The curves correspond to the following ground state structures (**Table S7, Figure S22**): dcm-a1; chlo-a1; ccl4-a1; cyhex-a1; dmso-a1; pin-a1; cs2-a1(black) and cs2-a2(violet, dashed); becn-a1(black), becn-a2(violet, dashed); meno2-a1(black), meno2-a2(gray, dashed), meno2-a5(violet, dashed). Notice that using the N-CH_3 instead of N-H substition in NDI the abundance of becn-a2 and becn-a5 (benzonitrile) complexes significantly increases and three instead of one complexes can be present in the solution. In all considered complexes, but nitromethane, the solvent arrangement in the gap is similar in the ground and excited state. In contrast, in the most stable excited state of CH_3NO_2@NDI-BINAM, the NO_2 group points to the AN plane (red line) while the next excited state corresponds to the meno2-a2 geometry (orange, dashed line).

Table S8. The estimated values of the EET coupling components for the energy transfer in the first model for the Sol@(NDI-BINAM) systems.

The estimated (CAM-B3LYP/D3/TZVP) values of the EET coupling components for the AN→NDI energy transfer (eV) and the EET rate (|V²|·J) for the Sol@(NDI-BINAM) systems where NDI and AN moieties are free molecules (left) in the singlet states frozen in the geometry of the Sol@(NDI-BINAM) system (right) with the optimal position of the solvent molecule Sol between the NDI and AN planes. *The V_{exch} coupling term is separated into the exact-exchange* V_{eex} *and exchange-correlation* V_{xc} *contributions.*

Fragment 1 (NDI, charge, multiplicity: 0 1)
Fragment 2 (solvent Sol, charge, multiplicity: 0 1)
Fragment 3 (AN, charge, multiplicity: 0 1)

initial system geometry

Sol	EET	ΔW $\cdot 10^{-1}$	V_{Coul} $\cdot 10^{-2}$	V_{eex} $\cdot 10^{-5}$	V_{xc} $\cdot 10^{-5}$	V_{ovlp} $\cdot 10^{-7}$	V_{Tot} $\cdot 10^{-2}$	$W_{avg} \cdot V_{Ovlp}$ $\cdot 10^{-6}$	EET rate $\cdot 10^{-10}$
none		4.29	2.65	-17.3	-18.4	16.4	2.61	6.59	11.2
conf2		4.32	-2.38	45.7	19.9	17.5	2.31	6.87	9.39
C_6H_5CN		4.29	-2.26	-1.73	2.94	1.43	-2.26	0.57	0.73
CH_3CN		4.16	2.03	4.93	0.09	-7.49	2.04	-3.00	-3.10
CH_3NO_2		4.19	-2.07	-9.13	-1.27	8.92	-2.08	3.57	3.84
CS_2	AN→NDI	4.21	2.42	-2.41	-8.39	3.08	2.41	1.24	1.79
CCl_4		4.22	2.43	-1.98	-8.09	2.33	2.42	0.94	1.37
CCl_3H		4.14	-2.34	0.65	6.20	-1.08	-2.34	-0.43	-0.59
CCl_2H_2		4.21	-2.51	8.20	12.5	-7.87	-2.48	-3.15	-4.86
C_6H_{12}		4.24	-2.41	3.09	8.82	-3.78	-2.40	-1.52	-2.18
(S)-(α)-pinene		4.28	-2.40	4.94	9.77	-5.73	-2.38	-2.30	-3.25

Sol	EET	ΔW	V_{Coul} $\cdot 10^{-3}$	V_{eex} $\cdot 10^{-4}$	V_{xc} $\cdot 10^{-4}$	V_{ovlp} $\cdot 10^{-7}$	V_{Tot} $\cdot 10^{-3}$	$W_{avg} \cdot V_{Ovlp}$ $\cdot 10^{-6}$	EET rate $\cdot 10^{-11}$
C_6H_5CN		1.55	16.3	-7.80	-3.47	26.7	15.2	12.2	61.5
CH_3CN		4.12	-1.08	5.50	0.21	0.81	-0.51	-0.47	0.00
CH_3NO_2		0.34	1.26	5.42	0.72	2.79	1.88	-1.11	0.09
CS_2		0.04	0.00	-0.48	0.00	0.00	-0.05	0.00	0.00
CCl_4	Solvent→NDI	2.40	2.24	-1.83	-0.48	4.73	2.01	2.37	0.19
CCl_3H		2.80	-4.24	10.9	3.68	-31.2	-2.80	-16.2	-2.45
CCl_2H_2		3.29	-5.97	0.89	1.99	-20.3	-5.70	-11.1	-6.59
C_6H_{12}		4.95	8.54	-1.84	-1.40	8.75	8.22	5.49	5.92
(S)-(α)-pinene		2.72	-9.05	13.2	6.50	-70.2	-7.11	-36.2	-35.5

Sol	EET	ΔW	V_{Coul} $\cdot 10^{-3}$	V_{eex} $\cdot 10^{-4}$	V_{xc} $\cdot 10^{-4}$	V_{ovlp} $\cdot 10^{-6}$	V_{Tot} $\cdot 10^{-3}$	$W_{avg} \cdot V_{Ovlp}$ $\cdot 10^{-6}$	EET rate $\cdot 10^{-11}$
C_6H_5CN		-1.12	0.66	-1.19	-1.11	1.63	0.44	7.80	0.03
CH_3CN		-3.70	0.85	3.99	-0.44	0.85	1.21	5.14	0.13
CH_3NO_2		0.02	-5.10	2.19	1.07	1.59	-4.78	-6.69	3.63
CS_2		0.38	0.00	1.72	0.00	0.00	0.18	0.00	0.00
CCl_4	AN→ Solvent	-1.98	-0.13	6.77	1.24	-1.26	0.66	-6.57	-0.05
CCl_3H		-2.39	-0.32	3.83	1.11	-1.38	0.16	-7.48	0.00
CCl_2H_2		-2.87	3.42	-14.3	-2.44	3.89	1.76	22.0	1.21
C_6H_{12}		-4.53	-4.43	-3.08	-2.03	2.89	-4.93	18.8	7.02
(S)-(α)-pinene		-2.29	5.36	3.32	0.44	-0.58	5.73	-3.11	-1.90

Table S9. The estimated values of the EET coupling components for the energy transfer in the second model for the Sol@(NDI-BINAM) systems.

The estimated (CAM-B3LYP/D3/TZVP) values of the EET coupling components for the AN→NDI energy transfer (eV) and the EET rate ($|V^2|\cdot J$) for the Sol@(NDI-BINAM) systems where NDI and AN moieties are charged by +1 and -1 *e* and are frozen in the geometry of the Sol@(NDI-BINAM) system with the optimal position of the solvent molecule M between the NDI and AN planes. *The V_{exch} coupling term is separated into the exact-exchange* V_{eex} *and exchange-correlation* V_{xc} *contributions.*

Fragment 1 (NDI, charge, multiplicity: +1 1)
Fragment 2 (solvent Sol, charge, multiplicity: 0 1)
Fragment 3 (AN, charge, multiplicity: -1 1)

Sol	EET	ΔW	V_{Coul} $\cdot 10^{-2}$	V_{eex} $\cdot 10^{-3}$	V_{xc} $\cdot 10^{-4}$	V_{ovlp} $\cdot 10^{-6}$	V_{Tot} $\cdot 10^{-2}$	$W_{avg} \cdot V_{Ovlp}$ $\cdot 10^{-6}$	EET rate $\cdot 10^{-9}$
none		2.44	-1.15	-2.32	-1.48	6.46	-1.40	3.55	1.26
conf2		2.42	1.69	2.44	0.11	4.19	1.94	-4.19	-2.77
C_6H_5CN		2.51	-1.57	-2.90	0.45	3.56	-1.85	2.07	1.22
CH_3CN		2.53	1.78	2.27	-0.51	-7.04	2.00	-4.21	-2.82
CH_3NO_2		2.53	-1.70	-2.35	0.00	8.01	-1.93	4.78	3.00
CS_2	AN→NDI	2.48	1.25	2.84	1.36	-7.20	1.55	-4.13	-1.72
CCl_4		2.49	1.27	2.83	1.27	-6.89	1.56	-3.95	-1.68
CCl_3H		2.50	-1.30	-2.87	-1.08	6.98	-1.60	4.05	1.78
CCl_2H_2		2.47	-1.23	-2.67	-1.14	6.11	-1.51	3.47	1.39
C_6H_{12}		2.48	1.25	2.76	1.04	-6.04	1.53	-3.46	-1.42
(S)-(α)-pinene		2.48	-1.25	-2.60	-0.98	6.16	-1.52	3.52	1.42

Sol	EET	ΔW	V_{Coul} $\cdot 10^{-3}$	V_{eex} $\cdot 10^{-4}$	V_{xc} $\cdot 10^{-5}$	V_{ovlp} $\cdot 10^{-7}$	V_{Tot} $\cdot 10^{-3}$	$W_{avg} \cdot V_{Ovlp}$ $\cdot 10^{-7}$	EET rate $\cdot 10^{-11}$
C_6H_5CN		6.01	-0.05	-2.08	-8.85	4.25	-0.35	9.92	0.00
CH_3CN		8.58	-0.12	-8.67	-0.66	-0.03	-0.99	-0.10	0.00
CH_3NO_2		4.87	0.86	-0.15	-5.89	3.47	0.78	6.12	0.00
CS_2		4.51	0.00	10.4	0.02	-0.01	1.04	-0.02	0.00
CCl_4	Solvent→NDI	6.87	-0.93	0.52	2.11	-1.72	-0.02	-4.76	0.00
CCl_3H		7.27	2.47	-5.05	-20.1	16.2	1.77	47.9	0.00
CCl_2H_2		7.75	-0.40	0.81	3.82	-2.38	-0.28	-7.63	0.00
C_6H_{12}		9.41	1.03	-1.23	3.65	-4.70	0.94	-19.0	0.00
(S)-(α)-pinene		7.19	-14.1	-4.67	-0.25	-2.02	-14.5	-5.91	-0.04

Sol	EET	ΔW	V_{Coul} $\cdot 10^{-4}$	V_{eex} $\cdot 10^{-5}$	V_{xc} $\cdot 10^{-7}$	V_{ovlp} $\cdot 10^{-8}$	V_{Tot} $\cdot 10^{-4}$	$W_{avg} \cdot V_{Ovlp}$ $\cdot 10^{-6}$	EET rate $\cdot 10^{-11}$
C_6H_5CN		-3.51	-4.79	-0.24	1.67	-0.76	-4.81	-2.70	0.00
CH_3CN		-6.05	-0.31	1.80	8.28	-1.44	-012	-7.10	0.00
CH_3NO_2		-2.33	-1.11	3.28	5.66	-3.00	-0.77	-9.10	0.00
CS_2		-2.02	0.02	-1.07	-0.13	0.01	-0.09	0.00	0.00
CCl_4		-4.38	-0.16	0.30	-5.29	0.70	-0.13	2.80	0.00
CCl_3H	AN→ Solvent	-4.77	1.21	-0.08	11.5	-2.09	1.21	-8.80	0.00
CCl_2H_2		-5.28	-0.08	0.36	24.4	-2.69	-0.02	-11.9	0.00
C_6H_{12}		-6.93	4.22	-4.30	-24.0	5.85	3.77	30.9	0.00
(S)-(α)-pinene		-4.71	-8.83	0.49	9.33	-1.20	-8.77	-5.00	-0.00

The QED theory behind all aspects of RET is not yet fully developed[10], and neither are the quantum-chemical computational algorithms which, for example, do not yet allow for direct modelling of the through-bond RET coupling and still include the environment only via the implicit solvent model[15–18]. Nevertheless, we approximately estimated the intramolecular RET in the NDI-BINAM molecule using the accessible routines[16–19] applied to the following two model structures: (1) free NDI and AN molecules (not linked by N) frozen in the optimal NDI-BINAM geometry, and (2) NDI^{1+} and AN^{1-} charged fragments (linked by N) (which simulates an excess of charge at the moieties). In the two cases, the presence of one solvent molecule in its optimal position between NDI and AN was taken into consideration (**Tables S8, S9**).

At the first order of perturbation theory, the RET coupling between donor and acceptor, V_{EET}, is composed of three terms: V_{Coul}, V_{exch}, and V_{ovlp}[16]. The V_{Coul} Coulomb coupling is primarily determined by the dipole-dipole interaction between donor and acceptor moieties. The exchange V_{exch} coupling accounts for the electron indistinguishability and decays exponentially with

distance. The V_{ovlp} term results from the donor-acceptor orbitals overlap, is important in a short distances and also decays exponentially[16]. The estimated values of the V_{EET} components are gathered in **Tables S8** and **S9**. Although the EET scheme implemented here[45], as well as the use of the single reference DFT method and a small basis set, cannot account for numerous RET phenomena that may be present in our experimental systems, consideration of the RET coupling data allows for drawing the following qualitative conclusions:

I. The total RET coupling between AN and NDI moieties arranged as in the optimal NDI-BINAM structure is significant (a matter of ca. 10^{-2} eV) be the moieties free and neutral or connected by the N segment and charged by -1 and +1 e, or in doublet states.

II. The total coupling is determined by the Coulomb coupling, the V_{exch} term is one order of magnitude smaller when AN and NDI are charged and 2-3 orders smaller when they are neutral, while the V_{ovlp} one is next two orders of magnitude smaller.

III. The AN→Sol and Sol→NDI RETs, to and from the solvent molecule placed between the AN and NDI plates, Sol@(NDI-BINAM), are always accompanying the AN→ NDI RET and their total coupling is ca. one order smaller (ca. 10^{-3} eV). Again, the RET to and from Sol is predicted to be determined by the Coulomb coupling and the V_{exch}, and V_{ovlp} are much smaller.

IV. In the case of the C_6H_5CN, CH_3CN, and CH_3NO_2 molecules between the AN and NDI plates, the total coupling is either ca. 15 % smaller than those of the other solvents (**Table S8**) or ca. 25 % larger than the other systems (**Table S9**).

References

1 H. Shao, T. Nguyen, N. C. Romano, D. A. Modarelli and J. R. Parquette, *J. Am. Chem. Soc.*, 2009, **131**, 16374–16376.
2 M. Wakabayashi, S. Yokojima, T. Fukaminato, K. Shiino, M. Irie and S. Nakamura, *J. Phys. Chem. A*, 2014, **118**, 5046–5057.
3 T. Furo, T. Mori, T. Wada and Y. Inoue, *J. Am. Chem. Soc.*, 2005, **127**, 8242–8243.
4 J. L. Alonso-Gómez, P. Rivera-Fuentes, N. Harada, N. Berova and F. Diederich, *Angew. Chem. Int.*, 2009, **48**, 5545–5548.
5 Y. Saito, M. Satake, R. Mori, M. Okayasu, H. Masu, M. Tominaga, K. Katagiri, K. Yamaguchi, S. Kikkawa, H. Hikawa and I. Azumaya, *Org. Biomol. Chem*, 2020, **18**, 230–236.
6 W. P. Ozimiński and J. C. Dobrowolski, *J. Phys. Org. Chem.*, 2009, **22**, 769–778.
7 D. L. Andrews and J. S. Ford, *J. Chem. Phys.*, 2013, **139**, 14107.
8 D. Weeraddana, M. Premaratne and D. L. Andrews, *Phys. Rev. B.*, 2015, **92**, 35128.
9 A. Salam, *J. Chem. Phys.*, 2012, **136**, 14509.
10 G. A. Jones and D. S. Bradshaw, *Front. Phys.*, 2019, 7, 100.
11 S. Speiser, *Chem. Rev.*, 1996, **96**, 1953–1976.
12 D. Beljonne, C. Curutchet, G. D. Scholes and R. J. Silbey, *J. Phys. Chem. B*, 2009, **113**, 6583–6599.
13 J. Fan, M. Hu, P. Zhan and X. Peng, *Chem. Soc. Rev.*, 2013, **42**, 29–43.
14 S. Athanasopoulos, L. Alfonso Hernandez, D. Beljonne, S. Fernandez-Alberti and S. Tretiak, *J. Phys. Chem. Lett.*, 2017, **8**, 1688–1694.
15 W. Ding, L.-Y. Hsu and G. C. Schatz, *J. Chem. Phys.*, 2017, **146**, 64109.
16 Z.-Q. You and C.-P. Hsu, *Int. J. Quant. Chem.*, 2014, **114**, 102–115.
17 M. F. Iozzi, B. Mennucci, J. Tomasi and R. Cammi, *J. Chem. Phys.*, 2004, **120**, 7029–7040.
18 C. Curutchet and B. Mennucci, *J. Am. Chem. Soc.*, 2005, **127**, 16733–16744.
19 V. Russo, C. Curutchet and B. Mennucci, *J. Phys. Chem. B*, 2007, **111**, 853–863.
20 D. L. Andrews, *J. Raman Spectrosc.*, 2000, **31**, 791–796.
21 R. D. Jenkins and D. L. Andrews, *J. Phys. Chem. A*, 1998, **102**, 10834–10842.
22 R. D. Jenkins and D. L. Andrews, *Chem. Phys. Lett.*, 1999, **301**, 235–240.
23 D. Avnir, H. Z. Hel-Or and P. G. Mezey, in *Encyclopedia of Computational Chemistry*, 1998, p. 2890−2901.
24 M. H. Jamróz, J. E. Rode, S. Ostrowski, P. F. J. Lipiński and J. C. Dobrowolski, *J. Chem. Inform. Model.*, 2012, **52**, 1462–1479.
25 P. F. J. Lipiński and J. C. Dobrowolski, *RSC Adv.*, 2014, **4**, 47047–47055.
26 CONFLEX 7, Conflex Corp. Japan 2012.
27 H. Goto and E. Osawa, *J. Am. Chem. Soc.*, 1989, **111**, 8950–8951.
28 M. J. Frisch, G. W. Trucks, H. B. Schlegel, G. E. Scuseria, M. A. Robb, J. R. Cheeseman, G. Scalmani, V. Barone, B. Mennucci, G. A. Petersson, H. Nakatsuji, M. Caricato, X. Li, H. P. Hratchian, A. F. Izmaylov, J. Bloino, G. Zheng, J. L. Sonnenberg, M. Hada, M. Ehara, K. Toyota, R. Fukuda, J. Hasegawa, M. Ishida, T. Nakajima, Y. Honda, O. Kitao, H. Nakai, T. Vreven, J. A. Montgomery, Jr., J. E. Peralta, F. Ogliaro, M. Bearpark, J. J. Heyd, E. Brothers, K. N. Kudin, V. N. Staroverov, T. Keith, R. Kobayashi, J. Normand, K. Raghavachari, A. Rendell, J. C. Burant, S. S. Iyengar, J. Tomasi, M. Cossi, N. Rega, J. M. Millam, M. Klene, J. E. Knox, J. B. Cross, V. Bakken, C. Adamo, J. Jaramillo, R. Gomperts, R. E. Stratmann, O. Yazyev, A. J. Austin, R. Cammi, C. Pomelli, J. W. Ochterski, R. L. Martin, K. Morokuma, V. G. Zakrzewski, G. A. Voth, P. Salvador, J. J. Dannenberg, S. Dapprich, A. D. Daniels, O. Farkas, J. B. Foresman, J. V. Ortiz, J. Cioslowski, and D. J. Fox, Gaussian, Inc., Wallingford CT, 2013.
29 R. D. Dennington II, T. A. Keith, J. M. Millam, GaussView 6.0.16, Copyright, Semichem, Inc. 2000-2016.
30 T. Yanai, D. P. Tew and N. C. Handy, *Chem. Phys. Lett.*, 2004, **393**, 51–57.
31 F. Weigend and R. Ahlrichs, *PCCP*, 2005, **7**, 3297–3305.
32 B. Mennucci, *WIREs Comput. Mol. Sci.*, 2012, **2**, 386–404.
33 G. Scalmani and M. J. Frisch, *J. Chem. Phys.*, 2010, **132**, 114110.
34 S. Grimme, J. Antony, S. Ehrlich and H. Krieg, *J. Chem. Phys.*, 2010, **132**, 154104.
35 A. Schäfer, C. Huber and R. Ahlrichs, *J. Chem. Phys.*, 1994, **100**, 5829–5835.
36 K. H. Hopmann, K. Ruud, M. Pecul, A. Kudelski, M. Dračínský and P. Bouř, *J. Phys. Chem. B*, 2011, **115**, 4128–4137.
37 M. Kamiński, A. Kudelski and M. Pecul, *J. Phys. Chem. B*, 2012, **116**, 4976–4990.
38 M. S. Gordon, J. S. Binkley, J. A. Pople, W. J. Pietro and W. J. Hehre, *J. Am. Chem. Soc.*, 1982, **104**, 2797–2803.
39 J. Autschbach, T. Ziegler, S. J. A. van Gisbergen and E. J. Baerends, *J. Chem. Phys.*, 2002, **116**, 6930–6940.
40 F. E. Jorge, S. S. Jorge and R. N. Suave, *Chirality*, 2015, **27**, 23–31.
41 S. F. Boys and F. Bernardi, *Mol. Phys.*, 1970, **19**, 553–566.
42 L. Turi and J. J. Dannenberg, *J. Phys. Chem.*, 1993, **97**, 2488–2490.

43 J. E. Rode and J. C. Dobrowolski, *Chem. Phys. Lett.*, 2002, **360**, 123–132.
44 T. Förster, *Ann. Phys.*, 1948, **437**, 55–75.
45 Gaussian 16, Revision C.01, M. J. Frisch, G. W. Trucks, H. B. Schlegel, G. E. Scuseria, M. A. Robb, J. R. Cheeseman, G. Scalmani, V. Barone, G. A. Petersson, H. Nakatsuji, X. Li, M. Caricato, A. V. Marenich, J. Bloino, B. G. Janesko, R. Gomperts, B. Mennucci, H. P. Hratchian, J. V. Ortiz, A. F. Izmaylov, J. L. Sonnenberg, D. Williams-Young, F. Ding, F. Lipparini, F. Egidi, J. Goings, B. Peng, A. Petrone, T. Henderson, D. Ranasinghe, V. G. Zakrzewski, J. Gao, N. Rega, G. Zheng, W. Liang, M. Hada, M. Ehara, K. Toyota, R. Fukuda, J. Hasegawa, M. Ishida, T. Nakajima, Y. Honda, O. Kitao, H. Nakai, T. Vreven, K. Throssell, J. A. Montgomery, Jr., J. E. Peralta, F. Ogliaro, M. J. Bearpark, J. J. Heyd, E. N. Brothers, K. N. Kudin, V. N. Staroverov, T. A. Keith, R. Kobayashi, J. Normand, K. Raghavachari, A. P. Rendell, J. C. Burant, S. S. Iyengar, J. Tomasi, M. Cossi, J. M. Millam, M. Klene, C. Adamo, R. Cammi, J. W. Ochterski, R. L. Martin, K. Morokuma, O. Farkas, J. B. Foresman, and D. J. Fox, Gaussian, Inc., Wallingford CT, 2019.

Appendix

Table S10. Cartesian coordinates of (*S*)-nBu-NDI-BINAM surrounded by 11 (CS_2, CCl_4, $CHCl_3$, CH_2Cl_2, CH_3CN) or 9 (C_6H_{12}) solvents molecules.

The Cartesian Coordinates of the most stable systems of nBu-NDI-BINAM surrounded by 11 (CS_2, CCl_4, $CHCl_3$, CHC_2Cl_2, CH_3CN) or 9 (C_6H_{12}) solvents molecules obtained at the CAM-B3LYP/D3/6-31G(d,p)/PCM level.

nBu-NDI-BINAM···11CS_2	nBu-NDI-BINAM···9C_6H_{12}	nBu-NDI-BINAM···11CCl_4
C 2.588213 0.558396 1.441280	C 2.728705 2.991217 -0.139383	C 3.099553 -1.900407 -1.997362
C 1.205647 0.743941 1.617251	C 1.371499 2.715353 0.100161	C 1.733821 -2.221265 -2.092038
C 0.311183 0.089028 0.804025	C 0.970923 1.450194 0.461677	C 0.785010 -1.461272 -1.426260
C -0.132720 -1.468862 -1.046589	C 1.529557 -0.887114 0.980951	C 0.231158 0.481237 -0.012557
C 0.337509 -2.314196 -2.025596	C 2.479350 -1.866016 1.163400	C 0.641575 1.592381 0.704074
C 1.719334 -2.501610 -2.198414	C 3.843057 -1.580029 0.973347	C 2.006741 1.910734 0.802100
C 2.614169 -1.839443 -1.389591	C 4.239336 -0.321742 0.581749	C 2.955575 1.125061 0.169465
C 3.055326 -0.273571 0.450851	C 3.669615 1.997702 0.000754	C 3.511377 -0.810033 -1.247807
C 2.152660 -0.974330 -0.376542	C 3.281743 0.695862 0.383283	C 2.559369 -0.012371 -0.571746
C 0.767393 -0.791154 -0.199306	C 1.917822 0.414928 0.601067	C 1.182258 -0.341384 -0.660602
C 4.516053 -0.418384 0.238708	C 5.101755 2.297892 -0.248068	C 4.951228 -0.460066 -1.172724
C 4.072110 -2.035269 -1.581922	C 5.681734 -0.014361 0.413639	C 4.388757 1.495304 0.251534
C -1.137562 0.342508 0.956649	C -0.455665 1.188641 0.756109	C -0.653612 -1.798226 -1.552452
C -1.589788 -1.264887 -0.885086	C 0.097109 -1.193359 1.211618	C -1.209074 0.166194 -0.124687
O 4.515705 -2.824090 -2.396657	O 6.552330 -0.826790 0.669725	O 4.761747 2.481636 0.878329
O 5.330581 0.178086 0.919486	O 5.481384 3.402424 -0.587273	O 5.815075 -1.110429 -1.749446
O -1.584044 1.088499 1.805700	O -1.293277 2.068708 0.743342	O -1.053217 -2.712558 -2.255948
O -2.408807 -1.884374 -1.539393	O -0.285548 -2.303713 1.531020	O -2.076613 0.889997 0.351124
N 4.924470 -1.269349 -0.787632	N 6.006092 1.248017 -0.077158	N 5.298816 0.665141 -0.413443
N -1.990689 -0.318697 0.061991	N -0.803155 -0.129879 1.080837	N -1.557640 -0.992569 -0.829083
H 3.302530 1.074259 2.069066	H 3.050018 3.983615 -0.432706	H 3.844565 -2.498073 -2.508720
H 0.824056 1.412185 2.378099	H 0.623475 3.491205 0.008548	H 1.404500 -3.064268 -2.685969
H -0.373599 -2.825604 -2.660571	H 2.160562 -2.853963 1.466294	H -0.100369 2.211368 1.188606
H 2.100176 -3.161896 -2.968154	H 4.595393 -2.341581 1.138472	H 2.332785 2.777204 1.363439
C -3.402648 -0.071147 0.210238	C -2.167874 -0.362897 1.488445	C -2.971252 -1.258928 -1.008517
C -4.072051 0.697241 -0.709147	C -3.051319 -1.020566 0.667377	C -3.707968 -1.885989 -0.022016
C -4.043366 -0.639991 1.330647	C -2.523191 0.101523 2.774304	C -3.547632 -0.827681 -2.227023
C -5.472727 0.925568 -0.501720	C -4.379765 -1.248833 1.162592	C -5.102450 -2.128351 -0.273037
C -5.376012 -0.441872 1.530708	C -3.783190 -0.106195 3.248852	C -4.883247 -1.031608 -2.463919
H -3.452945 -1.224215 2.022670	H -1.778192 0.620839 3.365167	H -2.915824 -0.336625 -2.958392
C -6.236244 1.727365 -1.387708	C -5.356651 -1.937873 0.398640	C -5.925435 -2.802639 0.668902
C -6.124628 0.349697 0.623539	C -4.739900 -0.791506 2.460135	C -5.693218 -1.687793 -1.500821
H -5.872046 -0.879487 2.391199	H -4.064576 0.244166 4.236621	H -5.331716 -0.698956 -3.394906
C -7.569221 1.947799 -1.164803	C -6.612215 -2.160045 0.898891	C -7.261019 -3.017571 0.414151
H -5.744183 2.169180 -2.245460	H -5.091371 -2.294215 -0.588536	H -5.486075 -3.142906 1.598842
C -7.506097 0.593336 0.825588	C -6.048292 -1.033527 2.947998	C -7.075984 -1.916528 -1.728504
C -8.214235 1.375009 -0.046668	C -6.965711 -1.704641 2.187479	C -7.846119 -2.564653 -0.791517
H -8.135902 2.567980 -1.851328	H -7.344072 -2.694325 0.302920	H -7.874638 -3.528680 1.149331
H -7.990792 0.148381 1.689419	H -6.306314 -0.673797 3.939458	H -7.513658 -1.569516 -2.659810
H -9.270973 1.557740 0.118052	H -7.964772 -1.886841 2.568807	H -8.903056 -2.730465 -0.973863
C -3.404334 1.274651 -1.913305	C -2.661082 -1.514899 -0.687817	C -3.144056 -2.295771 1.302203
C -2.666449 2.492746 -1.828525	C -2.569185 -0.617843 -1.794757	C -2.585151 -3.600381 1.478181
C -3.601980 0.667600 -3.145528	C -2.462509 -2.874060 -0.883836	C -3.335872 -1.465992 2.408194
C -2.427233 3.150503 -0.592506	C -2.790497 0.777236 -1.661088	C -2.409826 -4.502641 0.393305
C -2.161138 3.096535 -3.014316	C -2.284726 -1.124023 -3.094168	C -2.223961 -4.047796 2.792270
C -3.073755 1.274273 -4.319274	C -2.161563 -3.362357 -2.185538	C -2.978298 -1.929420 3.707266
C -1.746350 4.339452 -0.545555	C -2.733266 1.610106 -2.746288	C -1.910914 -5.770137 0.600626
H -2.785374 2.697017 0.322289	H -3.007626 1.189102 -0.685820	H -2.669705 -4.180559 -0.606400
C -1.463746 4.326654 -2.932918	C -2.238439 -0.237668 -4.197739	C -1.683521 -5.347861 2.965527
C -2.384680 2.448047 -4.254938	C -2.078264 -2.517488 -3.251574	C -2.444348 -3.177167 3.891207
H -3.239950 0.787076 -5.275192	H -1.999736 -4.427929 -2.316669	H -3.138000 -1.271846 4.556773
C -1.261348 4.943448 -1.726180	C -2.454853 1.105188 -4.034045	C -1.532559 -6.199572 1.894011
H -1.585807 4.823114 0.412028	H -2.917938 2.669459 -2.611484	H -1.798385 -6.443794 -0.242021
H -1.089209 4.771848 -3.850350	H -2.035306 -0.646888 -5.182920	H -1.400170 -5.662828 3.966656
H -1.994892 2.900944 -5.161620	H -1.853559 -2.907548 -4.239749	H -2.174189 -3.509884 4.889192
H -0.732514 5.889274 -1.672096	H -2.420542 1.777159 -4.885052	H -1.123082 -7.194894 2.035218
N -4.361232 -0.486428 -3.263711	N -2.608806 -3.774370 0.160085	N -3.941725 -0.215501 2.279313
H -4.267966 -1.114462 -2.474441	H -2.277904 -3.432540 1.052375	H -3.708890 0.264451 1.415834
H -4.220482 -0.983622 -4.131991	H -2.269712 -4.705696 -0.031030	H -3.795008 0.397029 3.071171
C 6.369004 -1.439849 -0.989426	C 7.428151 1.534554 -0.309181	C 0.953497 5.844838 -0.859530
C 6.934765 -2.549053 -0.112408	C 8.128686 2.014369 0.954951	Cl 0.490543 5.784182 -2.591794
H 6.840251 -0.485413 -0.756812	H 7.475425 2.293821 -1.088885	Cl -0.290571 4.991604 0.119122
H 6.520125 -1.667106 -2.044190	H 7.884750 0.615893 -0.677740	Cl 1.054745 7.556999 -0.326599
C 8.427268 -2.761832 -0.343557	C 9.606931 2.303105 0.712282	C 6.906907 -5.313416 0.027770
H 6.754242 -2.294588 0.937488	H 7.626323 2.919268 1.314995	Cl 8.564839 -5.189599 0.720226
H 6.389565 -3.476788 -0.322030	H 8.021427 1.247778 1.729887	Cl 5.735667 -5.631074 1.351446
C 8.998698 -3.867068 0.538548	C 10.320665 2.784703 1.971065	Cl 6.860877 -6.676224 -1.146922
H 8.599813 -3.004465 -1.399107	H 10.094955 1.396010 0.335432	C 5.526843 -1.642064 3.002799
H 8.960738 -1.823023 -0.151418	H 9.703960 3.056765 -0.078616	Cl 7.133169 -2.279441 2.527884
H 10.068253 -4.007944 0.362253	H 11.377947 2.985504 1.779568	Cl 5.604417 0.153095 3.068396
H 8.861663 -3.631768 1.598569	H 9.868025 3.706365 2.349793	Cl 5.070936 -2.287507 4.612429
H 8.498515 -4.820934 0.343898	H 10.260751 2.035101 2.766211	C 6.528464 6.190070 -1.242480
C 1.934987 -4.870503 0.336924	C 1.510389 0.852525 -4.317032	Cl 6.055993 5.802192 -2.936760
S 3.172107 -5.422168 -0.423075	C 2.124037 0.383366 -2.997472	Cl 5.710002 7.704922 -0.732456
S 0.699369 -4.303358 1.090690	C 1.618525 -1.008954 -2.612272	Cl 8.310184 6.410851 -1.159326
C 3.557777 -2.174617 3.513899	C 1.840371 -2.021581 -3.737614	C -4.920473 -5.148776 -3.357467
S 2.858596 -1.243553 4.540724	C 1.241459 -1.541222 -5.059662	Cl -6.079359 -4.272295 -4.422786

S	4.248436	-3.098397	2.471425
C	-1.877728	-0.865982	4.281511
S	-0.861067	-1.866054	3.658071
S	-2.900620	0.130270	4.884644
C	1.922186	2.291808	4.917752
S	3.309610	2.812227	4.447110
S	0.532463	1.762609	5.367125
C	3.827882	5.515843	-2.765241
S	5.365150	5.403160	-2.968216
S	2.291663	5.615561	-2.553516
C	2.337414	1.780209	-2.431675
S	3.714041	1.358372	-3.031447
S	0.968347	2.201956	-1.847106
C	-0.633140	-1.112056	-5.107599
S	0.498461	-0.062594	-4.959358
S	-1.763930	-2.173942	-5.237613
C	-3.204459	-4.700370	-2.759871
S	-4.592564	-4.062793	-3.036748
S	-1.819347	-5.349204	-2.480075
C	-3.803772	-3.938647	2.250107
S	-4.490742	-3.661566	3.618306
S	-3.113759	-4.175503	0.880411
C	-3.320065	3.468304	3.031561
S	-4.688592	2.863026	2.620757
S	-1.941464	4.065994	3.433813
C	2.740635	3.995623	0.860030
S	1.330979	4.419357	1.358078
S	4.139422	3.539933	0.362791
C	1.768190	-0.155480	-5.438247
H	2.116990	-1.353274	-1.700330
H	3.217625	0.356838	-3.094479
H	1.896575	1.102107	-2.205016
H	0.427994	0.965143	-4.182701
H	1.901781	1.839224	-4.587822
H	2.916797	-2.181251	-3.872290
H	1.412878	-2.988814	-3.457058
H	1.454485	-2.262783	-5.855853
H	0.151719	-1.491564	-4.958352
H	2.848535	-0.219318	-5.627105
H	1.306030	0.185751	-6.370908
H	0.545926	-0.953674	-2.384446
C	2.476153	-5.639874	-2.301055
C	3.247174	-6.291963	-1.150067
C	3.079614	-5.508925	0.155150
C	1.599061	-5.334401	0.498245
C	0.855983	-4.645386	-0.645610
C	1.001837	-5.438195	-1.944503
H	3.610820	-6.010312	0.971077
H	2.874464	-7.313862	-1.000114
H	4.307715	-6.381021	-1.408424
H	2.923818	-4.664380	-2.524458
H	2.571136	-6.243406	-3.210006
H	1.153018	-6.322712	0.672562
H	1.467371	-4.775940	1.431171
H	-0.193387	-4.505096	-0.384603
H	1.265687	-3.637291	-0.792388
H	0.521952	-6.419037	-1.823541
H	0.476305	-4.930872	-2.760454
H	3.541122	-4.518649	0.038369
C	-5.851953	-0.465311	-3.080747
C	-5.855354	-1.941614	-3.479137
C	-7.127295	-2.645723	-3.001750
C	-8.386927	-1.920578	-3.481551
C	-8.371981	-0.446406	-3.069004
C	-7.108350	0.250244	-3.575926
H	-7.136578	-3.689007	-3.335460
H	-5.791260	-2.017509	-4.573177
H	-4.966227	-2.442755	-3.081896
H	-5.807406	-0.390316	-1.987061
H	-4.955111	0.027386	-3.461771
H	-8.442129	-1.983587	-4.576634
H	-9.282657	-2.417772	-3.093513
H	-9.268612	0.061904	-3.439941
H	-8.400704	-0.378528	-1.972745
H	-7.115018	0.251369	-4.674451
H	-7.093644	1.298671	-3.261501
H	-7.130235	-2.667263	-1.905274
C	-1.188265	-3.464665	5.875335
C	-0.620061	-3.805056	4.495892
C	-1.645320	-4.541178	3.632872
C	-2.940098	-3.733206	3.521099
C	-3.520236	-3.416139	4.899325
C	-2.496966	-2.678183	5.764563
H	-1.221373	-4.730169	2.640406
H	-0.337135	-2.883033	3.977097
H	0.294025	-4.400681	4.597737
H	-1.376491	-4.395247	6.427202
H	-0.453766	-2.897978	6.458372
H	-2.720348	-2.785635	3.016017
H	-3.677595	-4.256850	2.904098
H	-4.431683	-2.818797	4.791703
H	-3.809768	-4.351436	5.396797
H	-2.294958	-1.701512	5.306229
H	-2.906949	-2.480190	6.760950
H	-1.865494	-5.521252	4.076805
C	-4.704933	2.724719	1.213016
C	-4.782719	3.494370	-0.106763
C	-5.736854	2.819507	-1.096056
C	-7.119817	2.598449	-0.479514
C	-7.019149	1.804118	0.823895
C	-6.091076	2.503002	1.818357
H	-5.816972	3.413890	-2.013531
H	-5.134082	4.516052	0.092325
H	-3.780239	3.589011	-0.539631
H	-4.234962	1.751049	1.038696
H	-4.057573	3.253668	1.918132
H	-7.585818	3.571683	-0.273604
H	-7.773437	2.080525	-1.189560
H	-8.012618	1.660045	1.262591
H	-6.624267	0.803402	0.608242
H	-6.525988	3.472644	2.097057
H	-6.009885	1.915389	2.738540
H	-5.320543	1.850521	-1.390848
C	0.752574	-0.303149	5.223498
Cl	-4.588093	-6.774492	-4.061005
Cl	-5.635426	-5.334693	-1.725730
C	-3.150829	4.236211	3.331421
Cl	-1.906555	4.219676	4.631743
Cl	-3.269264	5.882439	2.635949
Cl	-4.737875	3.750401	4.022577
C	6.730726	1.029379	-0.374188
C	7.173586	1.750461	-1.650581
H	7.296221	0.106586	-0.233144
H	6.866273	1.664493	0.499896
C	8.594151	2.310175	-1.528448
H	7.112508	1.052445	-2.492366
H	6.475490	2.569809	-1.855928
C	9.059594	3.005410	-2.810697
H	8.628621	3.021030	-0.692421
H	9.287717	1.497419	-1.274633
H	10.071375	3.408348	-2.701437
H	9.064163	2.308864	-3.656548
H	8.395766	3.837662	-3.067378
C	0.557488	-0.627874	3.889423
Cl	-0.704801	0.642554	3.740622
Cl	2.173963	0.148649	3.854209
Cl	0.351463	-1.503956	5.442297
C	-5.193128	3.802655	-1.061764
Cl	-3.717082	4.824239	-1.188100
Cl	-5.949902	4.044042	0.543618
Cl	-6.358874	4.281248	-2.347853
C	1.763725	-5.759206	-0.701649
Cl	1.572375	-4.514099	0.579552
Cl	1.709304	-7.388893	0.049934
Cl	3.346397	-5.528561	-1.521944
C	-8.341501	0.971909	1.267102
Cl	-8.415088	1.379088	-0.482056
Cl	-8.785464	2.430251	2.234329
Cl	-9.527973	-0.338298	1.619111
C	-0.056044	2.010828	-3.776906
Cl	1.550712	2.211612	-3.001115
Cl	-0.254585	0.298205	-4.299356
Cl	-1.345671	2.419785	-2.605509
Cl	2.548074	5.056747	-0.629544
Cl	6.036103	4.845306	-0.172656
Cl	4.297129	-2.140444	1.792901
Cl	6.492751	-3.785752	-0.804428
Cl	0.453420	-5.607220	-1.910234
Cl	-3.400973	-4.219672	-3.234258
Cl	-0.170875	3.088658	-5.205214
Cl	-4.745909	2.082184	-1.275009
Cl	-2.682958	3.066726	2.056823
Cl	0.410361	-1.781933	2.525759
Cl	-6.692170	0.432608	1.703117

	C 0.839519 0.947613 4.348680	
	C 2.200336 1.633316 4.474787	
	C 3.339140 0.656875 4.172514	
	C 3.255101 -0.594735 5.049188	
	C 1.891601 -1.276104 4.916145	
	H 2.250839 2.499302 3.805980	
	H 0.686592 0.660928 3.301077	
	H 0.035814 1.646613 4.599929	
	H 0.805923 -0.006167 6.279113	
	H -0.214807 -0.795117 5.084780	
	H 3.279824 0.354907 3.120838	
	H 4.308517 1.149770 4.302110	
	H 4.056730 -1.293435 4.786905	
	H 3.414781 -0.311358 6.097812	
	H 1.773992 -1.650628 3.891757	
	H 1.832096 -2.150240 5.572683	
	H 2.314848 2.017311 5.496790	
	C 0.699835 5.612003 3.257570	
	C -0.112890 4.316831 3.290366	
	C -1.571910 4.550654 2.895334	
	C -1.681681 5.252313 1.539775	
	C -0.867014 6.547530 1.511554	
	C 0.593447 6.298560 1.895385	
	H -2.103384 3.595207 2.863159	
	H 0.329360 3.592658 2.594689	
	H -0.056154 3.865420 4.286039	
	H 0.321724 6.292695 4.031800	
	H 1.748261 5.409141 3.502911	
	H -1.321684 4.567459 0.763759	
	H -2.731823 5.455836 1.303122	
	H -0.924623 7.013963 0.522437	
	H -1.302690 7.263467 2.221175	
	H 1.062686 5.657543 1.137614	
	H 1.154642 7.238989 1.894745	
	H -2.057823 5.168250 3.662759	
	C 7.544247 -3.658026 -1.988419	
	C 8.078262 -2.300778 -2.450226	
	C 7.130742 -1.655761 -3.463897	
	C 5.717597 -1.514978 -2.894428	
	C 5.180113 -2.851524 -2.376418	
	C 6.144071 -3.514296 -1.389652	
	H 7.510829 -0.677000 -3.776169	
	H 8.172992 -1.645528 -1.574515	
	H 9.079746 -2.407442 -2.880352	
	H 7.506637 -4.342472 -2.846784	
	H 8.224455 -4.106139 -1.256628	
	H 5.737227 -0.788060 -2.074284	
	H 5.042815 -1.105065 -3.653287	
	H 4.200899 -2.705630 -1.907829	
	H 5.019520 -3.524595 -3.229159	
	H 6.215939 -2.898975 -0.486449	
	H 5.755204 -4.491958 -1.085694	
	H 7.093328 -2.280613 -4.366130	
	C -1.214391 6.214282 -2.425680	
	C 0.202203 6.791947 -2.386059	
	C 1.234571 5.708826 -2.065479	
	C 1.131818 4.526795 -3.032021	
	C -0.288063 3.960381 -3.076603	
	C -1.306394 5.050659 -3.413767	
	H 2.246496 6.127999 -2.078790	
	H 0.436234 7.234518 -3.363164	
	H 0.265022 7.601757 -1.651182	
	H -1.486893 5.852279 -1.425605	
	H -1.936571 6.995715 -2.684279	
	H 1.414853 4.863693 -4.037900	
	H 1.844608 3.741977 -2.756438	
	H -0.346772 3.143029 -3.800217	
	H -0.541642 3.521330 -2.102659	
	H -1.111830 5.422423 -4.428623	
	H -2.322015 4.640829 -3.422440	
	H 1.055468 5.357642 -1.042012	
nBu-NDI-BINAM···11$CHCl_3$	nBu-NDI-BINAM···11CH_2Cl_2	nBu-NDI-BINAM···11CH_3CN
C -2.507725 2.135931 -0.142641	C -2.518300 2.422813 -0.542674	C -2.493960 2.253516 -1.142899
C -1.147496 2.323728 -0.440867	C -1.139823 2.517662 -0.800170	C -1.116472 2.145908 -1.405522
C -0.258794 1.281213 -0.322072	C -0.312203 1.441601 -0.567641	C -0.414702 1.031689 -1.015889
C 0.161632 -1.104908 0.096511	C -0.030684 -0.917338 0.071279	C -0.396904 -1.212322 -0.010009
C -0.307560 -2.350057 0.444795	C -0.567168 -2.090195 0.546331	C -1.056315 -2.227823 0.642800
C -1.656455 -2.529625 0.786090	C -1.934221 -2.164517 0.866527	C -2.409973 -2.080187 0.990606
C -2.529209 -1.468311 0.730289	C -2.751830 -1.076513 0.672478	C -3.096573 -0.941818 0.642099
C -2.960676 0.904685 0.271124	C -3.048074 1.252942 -0.048505	C -3.145924 1.253403 -0.459437
C -2.071634 -0.185884 0.367203	C -2.223157 0.130999 0.171527	C -2.446294 0.095918 -0.054831
C -0.710671 0.001237 0.061815	C -0.846586 0.218835 -0.107896	C -1.076945 -0.027618 -0.358117
C -4.388839 0.706432 0.596174	C -4.495708 1.161951 0.251753	C -4.587668 1.381446 -0.140492
C -3.970453 -1.684359 0.975732	C -4.190102 -1.155628 1.012839	C -4.527267 -0.801257 1.003990
C 1.169754 1.490373 -0.641825	C 1.148996 1.574378 -0.752380	C 1.047675 0.959165 -1.238586
C 1.573738 -0.925638 -0.288562	C 1.395452 -0.846791 -0.303645	C 1.024421 -1.363560 -0.380873

O	-4.428474	-2.802094	1.174207	O	-4.692388	-2.186301	1.437600	O	-5.130821	-1.667474	1.609718
O	-5.200753	1.619683	0.544053	O	-5.265906	2.075328	-0.001188	O	-5.251033	2.332639	-0.531100
O	1.618677	2.585299	-0.933067	O	1.673317	2.641958	-1.027039	O	1.682469	1.883859	-1.717749
O	2.359815	-1.861063	-0.341741	O	2.105113	-1.833874	-0.373588	O	1.613189	-2.424197	-0.284890
N	-4.798393	-0.571701	0.959312	N	-4.957814	-0.013100	0.833436	N	-5.163843	0.381620	0.626200
N	1.995899	0.361843	-0.615078	N	1.915400	0.422943	-0.586309	N	1.682038	-0.221123	-0.850517
H	-3.202096	2.959808	-0.244110	H	-3.164749	3.273191	-0.714493	H	-3.038524	3.133249	-1.463846
H	-0.794175	3.286740	-0.780445	H	-0.718636	3.436994	-1.185043	H	-0.601745	2.948309	-1.908861
H	0.358671	-3.198906	0.435774	H	0.072823	-2.951760	0.681364	H	-0.522250	-3.136340	0.890653
H	-2.020526	-3.508376	1.070847	H	-2.353254	-3.080868	1.262300	H	-2.929153	-2.862756	1.527922
C	3.368858	0.504264	-1.048877	C	3.338072	0.540602	-0.794914	C	3.099885	-0.330853	-1.084670
C	4.385415	0.626215	-0.131822	C	4.195584	0.399848	0.270232	C	3.963415	-0.347386	-0.015632
C	3.609091	0.452499	-2.439914	C	3.791608	0.816195	-2.103349	C	3.540242	-0.432388	-2.422192
C	5.729205	0.701823	-0.616675	C	5.597972	0.555290	0.030566	C	5.365203	-0.460470	-0.286484
C	4.890045	0.513374	-2.909881	C	5.128054	0.950375	-2.342633	C	4.874283	-0.545762	-2.687792
H	2.768734	0.360136	-3.117210	H	3.065603	0.935820	-2.899085	H	2.798223	-0.422410	-3.211459
C	6.830850	0.842857	0.265343	C	6.546244	0.474937	1.081955	C	6.326664	-0.469925	0.756987
C	5.979997	0.634330	-2.014366	C	6.063097	0.828636	-1.284780	C	5.819849	-0.559561	-1.631074
H	5.082283	0.470534	-3.976312	H	5.487102	1.166749	-3.342796	H	5.225892	-0.625561	-3.711590
C	8.109536	0.910571	-0.219714	C	7.882089	0.651996	0.835770	C	7.663727	-0.572132	0.478937
H	6.646873	0.899456	1.332066	H	6.194478	0.279417	2.088057	H	5.983060	-0.392546	1.781634
C	7.314483	0.703945	-2.484920	C	7.452109	0.997406	-1.509266	C	7.210665	-0.665602	-1.886377
C	8.356468	0.839394	-1.608963	C	8.342976	0.911578	-0.474381	C	8.113050	-0.672400	-0.857214
H	8.940811	1.012114	0.468650	H	8.593889	0.596630	1.653014	H	8.385685	-0.576257	1.288749
H	7.491901	0.649805	-3.554303	H	7.793878	1.203990	-2.518531	H	7.545408	-0.740690	-2.916425
H	9.375661	0.891155	-1.976300	H	9.404144	1.045836	-0.654349	H	9.175116	-0.753714	-1.063062
C	4.115268	0.668174	1.332849	C	3.694958	0.083542	1.639633	C	3.476485	-0.258800	1.392747
C	3.802549	1.908168	1.963383	C	3.129004	1.103310	2.461803	C	3.167204	1.012595	1.964601
C	4.242212	-0.482522	2.091390	C	3.823404	-1.207436	2.128243	C	3.395777	-1.407760	2.169130
C	3.794526	3.136206	1.250841	C	3.011138	2.450543	2.027786	C	3.264442	2.221373	1.226716
C	3.546432	1.941303	3.362064	C	2.687863	0.784098	3.775332	C	2.767725	1.100309	3.327764
C	3.989153	-0.436675	3.488795	C	3.371000	-1.513465	3.441095	C	2.960627	-1.303757	3.524969
C	3.523297	4.317324	1.887750	C	2.491934	3.415707	2.850158	C	3.015018	3.435913	1.814258
H	4.032207	3.133229	0.194961	H	3.353294	2.715655	1.036002	H	3.539070	2.176074	0.181135
C	3.231597	3.175997	3.985813	C	2.143792	1.800213	4.598265	C	2.507456	2.365738	3.904608
C	3.640705	0.731615	4.098733	C	2.819464	-0.551489	4.232835	C	2.659367	-0.098894	4.079842
H	4.075428	-1.353021	4.064141	H	3.468172	-2.533510	3.798938	H	2.882438	-2.213970	4.108954
C	3.215637	4.341107	3.266392	C	2.048544	3.092677	4.152061	C	2.635973	3.517113	3.170493
H	3.553426	5.246533	1.329987	H	2.430554	4.440797	2.503433	H	3.124670	4.337056	1.222698
H	3.016400	3.179243	5.049946	H	1.809964	1.534501	5.597107	H	2.207167	2.406806	4.947787
H	3.440121	0.749705	5.165104	H	2.473579	-0.800336	5.231470	H	2.336419	-0.041139	5.114997
H	2.980058	5.282874	3.749724	H	1.641626	3.868378	4.792454	H	2.443506	4.483168	3.625769
N	4.690058	-1.675452	1.526493	N	4.436402	-2.206166	1.380209	N	3.783110	-2.647944	1.704860
H	4.415656	-1.815463	0.561817	H	4.284978	-2.134563	0.380606	H	3.777926	-2.765930	0.699155
H	4.502965	-2.496733	2.085813	H	4.249492	-3.144660	1.706551	H	3.328546	-3.423480	2.173423
C	-3.630273	-4.536850	-1.372617	C	-4.535423	-4.453771	-0.807581	C	-5.589454	0.285854	-3.380539
H	-3.897972	-4.326375	-0.339770	H	-5.170835	-5.333400	-0.776303	H	-6.605845	0.153633	-3.755216
Cl	-4.814113	-5.675620	-2.036066	Cl	-2.863808	-4.981523	-1.149421	C	1.011460	5.174907	-2.737957
Cl	-3.669425	-2.988723	-2.248026	Cl	-5.169432	-3.351830	-2.054854	H	1.448776	5.199311	-3.737698
Cl	-1.991615	-5.230574	-1.385952	C	-4.897970	1.846252	-3.241658	C	-3.726391	4.588483	1.299180
C	-6.667338	2.400724	-1.900116	H	-5.096892	2.336701	-2.292083	H	-4.387512	4.430488	0.444723
H	-6.715355	1.982947	-0.900040	Cl	-3.433050	2.572870	-3.954912	C	-3.481477	-5.021241	-0.997847
Cl	-8.291394	2.375287	-2.610465	Cl	-4.701208	0.101458	-2.930442	H	-4.352804	-5.677734	-1.021860
Cl	-6.049004	4.058665	-1.744729	C	-5.550188	5.155108	-0.019171	C	4.746145	2.966142	-2.191561
Cl	-5.542056	1.390254	-2.837549	H	-5.967553	4.179904	0.208552	H	5.471589	2.483152	-1.533888
C	-4.996153	4.481099	1.685369	Cl	-4.135822	5.402576	1.034205	C	1.173624	-5.570178	0.064122
H	-5.240227	3.665218	1.012195	Cl	-5.102315	5.156020	-1.748602	H	1.728930	-4.677495	0.350569
Cl	-6.486111	5.376818	2.040368	C	-7.112089	-3.947309	2.428766	C	-6.573642	0.544491	1.004031
Cl	-4.319576	3.747274	3.157550	H	-6.190841	-3.379966	2.339446	C	-7.518466	0.005727	-0.061752
Cl	-3.796285	5.517213	0.883350	Cl	-7.711915	-4.256019	0.776829	H	-6.740262	1.608555	1.169460
C	-6.892427	-4.605341	1.386665	Cl	-8.258508	-2.988539	3.403042	H	-6.712276	0.014925	1.945557
H	-6.202601	-3.806216	1.132427	C	4.380374	4.383891	-1.321394	C	-8.981384	0.135978	0.349449
Cl	-8.017114	-4.841619	0.034061	H	3.754261	3.496506	-1.291587	H	-7.349012	0.554103	-0.994916
Cl	-5.912603	-6.058711	1.673663	Cl	5.114629	4.491849	-2.943531	H	-7.274247	-1.044148	-0.255045
Cl	-7.758104	-4.111543	2.860087	Cl	5.601241	4.267293	-0.028250	C	-9.932175	-0.389257	-0.721200
C	3.635098	3.996771	-2.721320	C	2.336917	-4.974497	0.357672	H	-9.143569	-0.407824	1.287849
H	3.187108	3.241645	-2.081560	H	1.707140	-5.756362	-0.056212	H	-9.206681	1.188406	0.560256
Cl	3.316081	3.531276	-4.407168	Cl	1.793416	-4.648313	2.029968	H	-10.976292	-0.290066	-0.413097
Cl	2.850387	5.545100	-2.331405	Cl	4.029347	-5.525440	0.304510	H	-9.805684	0.159586	-1.659788
Cl	5.375278	4.069242	-2.385895	C	-6.398063	-0.099827	1.129263	H	-9.742118	-1.447095	-0.928004
C	2.569024	-4.591180	1.190674	C	-7.185400	-0.603523	-0.073038	C	-0.459942	2.099000	2.260571
H	2.562170	-3.807848	0.437269	H	-6.722592	0.898217	1.420619	H	-0.429566	2.903241	2.995390
Cl	2.106834	-3.828978	2.735865	H	-6.513445	-0.767329	1.982365	C	2.778871	-3.631102	-3.105143
Cl	1.380875	-5.821275	0.711353	C	-8.682308	-0.677692	0.207839	C	1.463954	6.570426	0.936021
Cl	4.206525	-5.260273	1.278807	H	-6.995951	0.060191	-0.923140	H	2.322301	6.407252	0.279714
C	-6.235207	-0.777228	1.206520	H	-6.812807	-1.594220	-0.350542	C	-1.821499	-2.306285	-3.193280
C	-6.978772	-1.130103	-0.076398	C	-9.458543	-1.231741	-0.981848	H	-2.372724	-1.853981	-2.367808
H	-6.617549	0.148960	1.632388	H	-8.855272	-1.311071	1.084625	C	-0.785925	-3.619973	3.886803
H	-6.329203	-1.563111	1.955221	H	-9.055936	0.320884	0.465842	H	-0.937717	-3.861485	4.940416
C	-8.488521	-1.202143	0.130131	H	-10.531478	-1.272858	-0.776330	H	1.044536	7.560658	0.749752
H	-6.747010	-0.377823	-0.837593	H	-9.309952	-0.612286	-1.872106	H	1.790122	6.513008	1.976167
H	-6.604416	-2.082451	-0.468881	H	-9.123129	-2.245303	-1.222077	H	5.216358	3.166110	-3.155720
C	-9.231698	-1.487451	-1.170056	C	-1.133425	1.957105	3.080596	H	3.892774	2.299447	-2.327162
H	-8.720330	-1.976704	0.869785	H	-0.822683	2.122084	2.053452	H	-1.529946	-4.146519	3.285049
H	-8.838534	-0.253957	0.555887	Cl	-0.948280	0.227807	3.458051	H	-0.887250	-2.540146	3.745169
H	-10.310862	-1.542067	-1.006663	Cl	-2.817780	2.516346	3.243509	H	-4.907645	0.399495	-4.225032

H	-9.042250	-0.701269	-1.907732
H	-8.908668	-2.438800	-1.602482
C	0.165053	1.104141	3.856162
H	0.981293	1.589830	3.328277
Cl	0.231989	-0.631425	3.481461
Cl	-1.359864	1.815621	3.290393
Cl	0.402260	1.395935	5.590209
C	3.962125	-2.977087	-2.675855
H	3.412261	-2.113897	-2.319602
Cl	2.948314	-4.408777	-2.385535
Cl	5.470266	-3.052808	-1.742168
Cl	4.285192	-2.761290	-4.406719
C	0.621840	5.590567	0.397837
H	1.341073	4.892150	-0.023586
Cl	-0.134160	4.802571	1.794572
Cl	1.476790	7.071704	0.873889
Cl	-0.581186	5.952069	-0.865122
C	7.760516	-2.279951	1.668779
H	6.811339	-1.785482	1.451847
Cl	8.536302	-2.709601	0.130102
Cl	7.365645	-3.736683	2.612104
Cl	8.775980	-1.171749	2.617957
C	-0.739903	-0.146796	-4.052936
H	-0.982691	-0.352277	-5.090612
Cl	-2.256869	0.160440	-3.184639
Cl	0.309120	1.287380	-4.010350
Cl	0.097241	-1.575583	-3.400980
C	3.348943	-2.514491	-3.126184
H	2.810407	-1.707587	-2.644170
Cl	2.293828	-3.952700	-3.105938
Cl	4.867164	-2.761343	-2.234825
C	0.625361	5.420720	0.316494
H	1.064232	4.473252	0.012608
Cl	-0.465187	5.121769	1.694941
Cl	1.951372	6.545184	0.715953
C	7.604524	-2.811794	1.165491
H	6.623399	-2.349162	1.270762
Cl	7.978472	-2.936486	-0.573683
Cl	7.528715	-4.410915	1.958272
C	-1.296757	-0.442082	-3.577291
H	-1.696198	-0.399248	-4.586029
Cl	-1.688928	-2.020112	-2.869676
Cl	0.466707	-0.155158	-3.672544
H	2.255636	-4.045345	-0.199139
H	3.589980	-2.287056	-4.160395
H	-0.507437	2.521408	3.764668
H	-1.728212	0.337087	-2.955845
H	-6.968820	-4.904354	2.921450
H	-6.252756	5.965862	0.148375
H	-5.716206	1.976827	-3.943465
H	0.039791	5.867654	-0.482167
H	-4.538128	-3.916794	0.136796
H	8.391762	-2.232902	1.638819
H	3.798686	5.285628	-1.150844
H	-5.550079	1.185711	-2.763081
H	1.293109	4.239463	-2.248501
H	1.410780	6.007652	-2.158564
H	-4.057896	5.461957	1.862900
H	-2.704512	4.748741	0.945786
H	-3.817262	-3.981012	-1.029847
H	-2.848953	-5.223064	-1.864028
H	2.002892	-2.940305	-2.776422
H	2.309254	-4.561075	-3.428356
H	-1.228797	2.326034	1.524786
H	0.510678	2.048729	1.766120
H	1.866192	-6.390934	-0.129065
H	0.495698	-5.843907	0.873727
H	3.326412	-3.186775	-3.937471
H	-2.456348	-2.301425	-4.080623
H	-1.555079	-3.332772	-2.933177
C	0.540764	-4.048801	3.470567
N	1.590690	-4.390377	3.129371
C	-5.202086	-0.873367	-2.587883
N	-4.891263	-1.786007	-1.950764
C	-0.752377	0.833261	2.916542
N	-0.982544	-0.176147	3.428844
C	0.396478	-5.280818	-1.130687
N	-0.225933	-5.050060	-2.076819
C	-0.601376	-1.557833	-3.447981
N	0.374402	-0.967254	-3.635106
C	3.687372	-3.890743	-1.998392
N	4.406243	-4.104736	-1.119281
C	-3.765415	3.401740	2.141406
N	-3.785849	2.437373	2.778748
C	-0.439479	5.268897	-2.829221
N	-1.592581	5.312514	-2.894759
C	-2.710937	-5.262251	0.213021
N	-2.090969	-5.432415	1.173694
C	0.454994	5.552493	0.679303
N	-0.345330	4.747772	0.462619
C	4.279969	4.209619	-1.595212
N	3.891436	5.189037	-1.120431

Table S11. Cartesian coordinates of the most stable (1:1) complexes of (*S*)-NDI-BINAM with different solvents.

The Cartesian Coordinates of the (1:1) complexes formed between NDI-BINAM and the solvent incorporated between the NDI and AN plates calculated at the CAM-B3LYP/D3/6-31G(d,p) level.

ccl4-a1	chlo-a1	chlo-a2
C -2.962600 -1.646751 -2.460445	C -3.474119 -1.219235 -2.409718	C -2.756542 -0.520008 -2.305004
C -1.558130 -1.701102 -2.501476	C -2.072339 -1.295290 -2.483227	C -1.367530 -0.403105 -2.489227
C -0.834750 -1.771987 -1.334022	C -1.327181 -1.453678 -1.337925	C -0.502570 -1.019256 -1.616651
C -0.762320 -1.897790 1.120788	C -1.207813 -1.734258 1.102115	C -0.118637 -2.400912 0.381840
C -1.416904 -1.948579 2.329471	C -1.836983 -1.812110 2.322665	C -0.614938 -3.157696 1.417880
C -2.820862 -1.891887 2.376462	C -3.236223 -1.700864 2.405712	C -2.002688 -3.321843 1.573878
C -3.549434 -1.789860 1.215193	C -3.986350 -1.527616 1.266701	C -2.874920 -2.713324 0.701754
C -3.621910 -1.665790 -1.254323	C -4.108405 -1.291449 -1.191935	C -3.258770 -1.264473 -1.263563
C -2.899272 -1.754804 -0.040978	C -3.362694 -1.456756 -0.001164	C -2.387410 -1.922106 -0.364186
C -1.491097 -1.809124 -0.084972	C -1.958430 -1.550913 -0.079289	C -0.995487 -1.779788 -0.533814
C -5.103763 -1.587260 -1.221163	C -5.587586 -1.181116 -1.122057	C -4.723208 -1.362974 -1.059893
C -5.030710 -1.711877 1.277004	C -5.463909 -1.414241 1.365386	C -4.338317 -2.890125 0.873261
C 0.647174 -1.777293 -1.391850	C 0.151071 -1.519550 -1.431146	C 0.961187 -0.877625 -1.816052
C 0.720310 -1.914949 1.089589	C 0.270233 -1.830100 1.033459	C 1.347566 -2.212177 0.247015
O -5.664371 -1.732371 2.311016	O -6.074543 -1.460123 2.412324	O -4.841079 -3.555969 1.752571
O -5.797601 -1.507365 -2.212641	O -6.298862 -1.038218 -2.094109	O -5.533555 -0.725336 -1.708742
O 1.257040 -1.761361 -2.440200	O 0.739384 -1.453298 -2.490348	O 1.435245 -0.312682 -2.777993
O 1.383789 -2.028086 2.102952	O 0.947832 -2.046644 2.020491	O 2.136077 -2.715499 1.023838
N -5.670398 -1.611806 0.047745	N -6.127450 -1.251290 0.155950	N -5.130677 -2.226489 -0.059908
N 1.323592 -1.789551 -0.163015	N 0.853615 -1.668542 -0.225147	N 1.782911 -1.418094 -0.816143
H -6.681888 -1.563866 0.079655	H -7.135725 -1.170801 0.213576	H -6.130764 -2.285682 0.092256
C 2.762113 -1.707305 -0.207366	C 2.288533 -1.780445 -0.318184	C 3.184643 -1.085411 -0.864831
C 3.385570 -0.527180 0.116339	C 3.096756 -0.734752 0.052395	C 3.659730 -0.105719 -0.024801
C 3.475999 -2.859592 -0.598003	C 2.806287 -2.999691 -0.807340	C 4.013263 -1.770168 -1.775461
C 4.815958 -0.480748 0.053007	C 4.515983 -0.902721 -0.071397	C 5.052074 0.213151 -0.076331
C 4.837093 -2.824292 -0.663389	C 4.152710 -3.169392 -0.926778	C 5.342454 -1.467587 -1.837092
C 5.538364 0.698246 0.367920	C 5.423037 0.129382 0.279814	C 5.620213 1.205258 0.762785
C 5.542034 -1.637681 -0.341022	C 5.042344 -2.127413 -0.564327	C 5.896094 -0.473943 -0.991585
C 6.904711 0.724520 0.293391	C 6.773922 -0.047450 0.147624	C 6.954891 1.498867 0.693564
C 6.956485 -1.578998 -0.406618	C 6.445596 -2.281176 -0.687137	C 7.274522 -0.146391 -1.038059
C 7.624397 -0.426048 -0.097277	C 7.294161 -1.266062 -0.339887	C 7.793080 0.816313 -0.216266
C 2.626694 0.689394 0.532064	C 2.568159 0.556876 0.582924	C 2.747734 0.597872 0.923807
C 2.082662 1.572363 -0.448667	C 2.160915 1.593378 -0.308380	C 1.907066 1.655881 0.464220
C 2.546354 1.011636 1.878965	C 2.609717 0.794022 1.949721	C 2.716527 0.211678 2.254997
C 2.134478 1.288486 -1.839032	C 2.064313 1.393418 -1.711669	C 1.919641 2.104319 -0.883248
C 1.481012 2.795078 -0.039175	C 1.845677 2.882741 0.206278	C 1.019876 2.295093 1.373203
C 1.924145 2.229717 2.271465	C 2.268184 2.082554 2.448561	C 1.836169 0.874897 3.154805
C 1.624357 2.170216 -2.756762	C 1.673138 2.410889 -2.543308	C 1.083154 3.105936 -1.300448
C 0.975612 3.689056 -1.012631	C 1.470477 3.920100 -0.682100	C 0.171133 3.329383 0.912794
C 1.419624 3.091522 1.345463	C 1.913349 3.091728 1.606970	C 1.014924 1.873663 2.726865
C 1.044802 3.389700 -2.346618	C 1.377256 3.693367 -2.030871	C 0.195155 3.729525 -0.397674
N 3.119131 0.203823 2.848883	N 3.047672 -0.176182 2.833846	N 3.554798 -0.781223 2.739958
H 3.091903 -0.784347 2.629976	H 2.810381 -1.121913 2.560435	H 3.769461 -1.502363 2.062804
H 2.784673 0.379486 3.785121	H 2.810990 0.000678 3.799051	H 3.249872 -1.187672 3.612603
H -3.548555 -1.578635 -3.369820	H -4.076919 -1.092088 -3.301634	H -3.450655 -0.028615 -2.977273
H -1.021495 -1.674101 -3.442674	H -1.555003 -1.224476 -3.432780	H -0.955141 0.176263 -3.306634
H -0.827578 -2.021256 3.235980	H -1.232238 -1.953200 3.210724	H 0.085050 -3.617825 2.105262
H -3.352828 -1.918150 3.320523	H -3.747636 -1.749620 3.360176	H -2.409511 -3.921782 2.379895
H 2.922426 -3.757174 -0.848253	H 2.113537 -3.784794 -1.087948	H 3.574505 -2.524862 -2.417561
H 5.396324 -3.704085 -0.965523	H 4.558687 -4.102825 -1.303466	H 5.991927 -1.985173 -2.535908
H 4.987591 1.580952 0.668690	H 5.028025 1.065199 0.655128	H 4.975307 1.725292 1.460913
H 7.441121 1.635720 0.536578	H 7.451993 0.754560 0.419946	H 7.375233 2.260624 1.341751
H 7.499916 -2.469066 -0.709466	H 6.833887 -3.222245 -1.065066	H 7.911330 -0.675407 -1.740792
H 8.707576 -0.391367 -0.151685	H 8.367222 -1.393138 -0.438730	H 8.849688 1.059375 -0.260321
H 2.574404 0.357018 -2.173874	H 2.283809 0.415925 -2.123247	H 2.587661 1.630894 -1.591758
H 1.870274 2.468673 3.329859	H 2.308831 2.252255 3.520639	H 1.824981 0.563502 4.195419
H 1.674235 1.927813 -3.813576	H 1.594295 2.227772 -3.609972	H 1.106867 3.424592 -2.337524
H 0.524683 4.618305 -0.677366	H 1.238486 4.897225 -0.269580	H -0.505855 3.800039 1.619706
H 0.952826 4.019606 1.662014	H 1.663943 4.071353 2.003488	H 0.339527 2.360261 3.424337
H 0.651311 4.079553 -3.085625	H 1.077679 4.491136 -2.702020	H -0.460451 4.523127 -0.740307
C -2.570195 2.146091 0.150841	C -1.585088 2.617070 -0.108968	C -4.583363 1.983079 -0.184201
Cl -1.730554 1.459205 1.558043	Cl -1.409302 1.610804 1.341759	Cl -4.842881 1.017657 1.291728
Cl -1.800871 1.564511 -1.334562	Cl -2.866480 1.988909 -1.167278	Cl -5.635991 3.410760 -0.205332
Cl -2.491013 3.922719 0.218721	H -0.649048 2.577325 -0.658238	Cl -2.874824 2.436717 -0.337630
Cl -4.281801 1.635939 0.177336	Cl -1.883085 4.312319 0.321342	H -4.859245 1.352961 -1.026328
**chlo-a3**	**dcm-a1**	**dcm-a2**
C -2.754302 -1.839280 -2.349478	C 3.302735 -0.845317 2.436923	C -3.461528 -0.706698 -2.467090
C -1.348954 -1.882686 -2.345193	C 1.907397 -0.982980 2.538531	C -2.064355 -0.819302 -2.577865
C -0.657103 -1.767456 -1.162221	C 1.155490 -1.258930 1.418244	C -1.308436 -1.159433 -1.478644
C -0.650906 -1.466640 1.278535	C 1.015752 -1.687316 -1.000015	C -1.161583 -1.760433 0.901372
C -1.338427 -1.307422 2.459485	C 1.635432 -1.832178 -2.218587	C -1.775942 -1.988401 2.109506
C -2.743680 -1.285214 2.462991	C 3.031437 -1.697329 -2.324664	C -3.172128 -1.866050 2.229162
C -3.441747 -1.418592 1.286112	C 3.787740 -1.421369 -1.211438	C -3.934014 -1.524140 1.138138
C -3.446734 -1.692714 -1.171114	C 3.925320 -0.988410 1.218967	C -4.081499 -0.933271 -1.258948
C -2.757130 -1.574187 0.058179	C 3.175060 -1.280180 0.056173	C -3.325287 -1.290427 -0.117730
C -1.347328 -1.603404 0.058223	C 1.774903 -1.415227 0.159376	C -1.924487 -1.407331 -0.233174
C -4.929801 -1.634185 -1.187246	C 5.397483 -0.812903 1.118555	C -5.557602 -0.797587 -1.149170
C -4.926148 -1.395123 1.301623	C 5.257950 -1.253258 -1.338843	C -5.406791 -1.393048 1.275837
C 0.825033 -1.814068 -1.174998	C -0.319091 -1.360393 1.536447	C 0.168327 -1.243816 -1.605548
C 0.832258 -1.486321 1.291693	C -0.460717 -1.791139 -0.909843	C 0.312828 -1.879717 0.794331

O	-5.588229	-1.281897	2.312028	O	5.858266	-1.358024	-2.387374	O	-6.005224	-1.577938	2.313939
O	-5.595482	-1.691120	-2.199977	O	6.109614	-0.560439	2.066618	O	-6.274747	-0.498961	-2.080208
O	1.462223	-2.014725	-2.187313	O	-0.893595	-1.263789	2.600759	O	0.739387	-1.051256	-2.658293
O	1.469570	-1.404454	2.324339	O	-1.149918	-2.061009	-1.874541	O	0.998579	-2.245887	1.728858
N	-5.532132	-1.507489	0.057775	N	5.926011	-0.964136	-0.156285	N	-6.081706	-1.038923	0.113500
N	1.469768	-1.605553	0.054005	N	-1.031133	-1.556235	0.345083	N	0.882527	-1.551419	-0.439550
H	-6.544032	-1.461666	0.054915	H	6.927740	-0.836009	-0.236038	H	-7.086807	-0.943854	0.199324
C	2.907978	-1.514826	0.035489	C	-2.469677	-1.537674	0.433173	C	2.320763	-1.581215	-0.539977
C	3.504328	-0.283526	0.169309	C	-3.160408	-0.439294	-0.019099	C	3.062466	-0.559324	0.001071
C	3.651649	-2.701369	-0.126718	C	-3.113920	-2.658161	0.997411	C	2.911385	-2.675173	-1.206599
C	4.933022	-0.217229	0.149949	C	-4.588382	-0.448131	0.088396	C	4.488177	-0.622157	-0.120750
C	5.014427	-2.648164	-0.150569	C	-4.472873	-2.674708	1.106074	C	4.267402	-2.742245	-1.329789
C	5.624151	1.013958	0.283273	C	-5.376965	0.644727	-0.353324	C	5.327198	0.391702	0.407755
C	5.689857	-1.409882	-0.012445	C	-5.244521	-1.574316	0.656161	C	5.090226	-1.721151	-0.792115
C	6.991648	1.055872	0.255401	C	-6.740171	0.618554	-0.235403	C	6.687206	0.316279	0.274471
C	7.105007	-1.332826	-0.035529	C	-6.657787	-1.571565	0.762696	C	6.501460	-1.771079	-0.912049
C	7.742705	-0.129786	0.095097	C	-7.390497	-0.501536	0.327784	C	7.283657	-0.776839	-0.391752
C	2.690324	0.954500	0.343280	C	-2.467980	0.736057	-0.625297	C	2.429689	0.591608	0.709551
C	2.063298	1.570468	-0.780964	C	-1.871887	1.736646	0.199305	C	1.903678	1.695235	-0.027394
C	2.554773	1.508822	1.607199	C	-2.466664	0.882793	-2.004863	C	2.432733	0.621066	2.096114
C	2.178182	1.046954	-2.096261	C	-1.834186	1.631747	1.615612	C	1.869074	1.714426	-1.447356
C	1.292701	2.751299	-0.597454	C	-1.296173	2.891457	-0.399514	C	1.399779	2.830355	0.667550
C	1.792252	2.698155	1.770692	C	-1.885456	2.044169	-2.586356	C	1.912563	1.759019	2.774593
C	1.542338	1.647192	-3.150796	C	-1.248708	2.606896	2.380692	C	1.365948	2.795531	-2.125902
C	0.652032	3.349962	-1.708437	C	-0.713949	3.889094	0.418700	C	0.895628	3.933259	-0.061763
C	1.184152	3.294748	0.707472	C	-1.326388	3.013333	-1.811417	C	1.419155	2.823717	2.085566
C	0.766344	2.811049	-2.961367	C	-0.683315	3.754129	1.782794	C	0.876645	3.923129	-1.432209
N	3.183849	0.952662	2.711375	N	-3.070757	-0.052424	-2.828707	N	2.986617	-0.409444	2.837889
H	3.261580	-0.055785	2.666873	H	-3.015281	-1.001406	-2.480472	H	2.884264	-1.322332	2.412633
H	2.790761	1.241995	3.595510	H	-2.781969	-0.001489	-3.794818	H	2.699589	-0.423992	3.805756
H	-3.315339	-1.917827	-3.273572	H	3.910073	-0.613813	3.304249	H	-4.073025	-0.434498	-3.319787
H	-0.787150	-2.001792	-3.264135	H	1.397595	-0.864992	3.487604	H	-1.557634	-0.633120	-3.517570
H	-0.773395	-1.200814	3.377881	H	1.024790	-2.039176	-3.089489	H	-1.162057	-2.257128	2.960980
H	-3.301352	-1.160757	3.383949	H	3.533726	-1.795940	-3.280101	H	-3.670910	-2.035002	3.176593
H	3.121257	-3.639972	-0.236850	H	-2.510124	-3.489020	1.343193	H	2.269407	-3.445341	-1.618175
H	5.597636	-3.554747	-0.277297	H	-4.979313	-3.530693	1.540612	H	4.732172	-3.578223	-1.842768
H	5.047569	1.922507	0.407885	H	-4.879648	1.503559	-0.787121	H	4.871353	1.229974	0.920198
H	7.505217	2.006093	0.357899	H	-7.327937	1.463872	-0.577634	H	7.313988	1.101488	0.683994
H	7.673434	-2.249586	-0.160701	H	-7.147677	-2.437187	1.198465	H	6.949665	-2.615373	-1.427275
H	8.826572	-0.081861	0.075344	H	-8.472080	-0.509152	0.414452	H	8.363283	-0.824081	-0.489538
H	2.761014	0.149217	-2.259789	H	-2.262338	0.758891	2.092896	H	2.235049	0.856588	-1.998144
H	1.698525	3.123591	2.765732	H	-1.897572	2.145750	-3.667699	H	1.920701	1.765028	3.860736
H	1.638209	1.220893	-4.144185	H	-1.227298	2.497626	3.460158	H	1.346032	2.782012	-3.210884
H	0.062164	4.247272	-1.543933	H	-0.292343	4.769451	-0.059395	H	0.509197	4.786341	0.487856
H	0.597129	4.196805	0.851886	H	-0.882973	3.891185	-2.271492	H	1.024540	3.683560	2.618157
H	0.267824	3.273960	-3.806449	H	-0.236438	4.525077	2.401987	H	0.477669	4.768895	-1.981146
C	-3.669325	2.422699	0.171187	C	2.334341	2.152209	0.310338	C	-1.999673	2.121234	-0.315643
Cl	-4.661185	1.930281	1.559061	Cl	4.052650	2.444720	0.675675	H	-0.958121	2.140694	-0.624331
Cl	-4.283841	1.707877	-1.336109	H	1.764411	3.041425	0.561959	Cl	-2.059594	1.720590	1.418293
H	-3.750236	3.501031	0.074752	H	2.001265	1.306354	0.904987	Cl	-2.717766	3.705017	-0.683069
Cl	-1.959308	2.031077	0.421354	Cl	2.048624	1.776125	-1.403484	H	-2.565248	1.367517	-0.853972
dcm-a3				dcm-a4				cs2-a1			
C	-3.497496	-0.328386	-2.588748	C	-2.583225	-3.304635	-0.719051	C	-3.500050	-0.735043	-2.485355
C	-2.104642	-0.513581	-2.631102	C	-1.192561	-3.347737	-0.520794	C	-2.107305	-0.918141	-2.551531
C	-1.427185	-0.925098	-1.507243	C	-0.578461	-2.409571	0.275442	C	-1.387577	-1.167665	-1.406825
C	-1.442072	-1.604414	0.858946	C	-0.718464	-0.429258	1.728185	C	-1.311704	-1.515056	1.026583
C	-2.135431	-1.857334	2.019910	C	-1.466867	0.552683	2.331708	C	-1.959530	-1.601166	2.236625
C	-3.528138	-1.676744	2.065574	C	-2.855357	0.612385	2.120464	C	-3.351464	-1.415646	2.309288
C	-4.207110	-1.246994	0.950148	C	-3.474163	-0.311429	1.312219	C	-4.075748	-1.151016	1.171162
C	-4.191891	-0.560304	-1.425425	C	-3.338085	-2.319641	-0.125434	C	-4.151906	-0.805314	-1.276885
C	-3.518221	-0.990503	-0.258218	C	-2.728161	-1.345563	0.699010	C	-3.433044	-1.066318	-0.086490
C	-2.120696	-1.172578	-0.301984	C	-1.334751	-1.397252	0.904258	C	-2.036259	-1.249631	-0.155794
C	-5.661815	-0.352212	-1.389409	C	-4.801664	-2.271104	-0.355102	C	-5.621135	-0.600642	-1.216730
C	-5.677706	-1.047758	1.013020	C	-4.926023	-0.204500	1.040306	C	-5.544282	-0.949441	1.259787
C	0.041851	-1.112223	-1.566681	C	0.899265	-2.425057	0.414463	C	0.083863	-1.334348	-1.490539
C	0.029012	-1.777125	0.832843	C	0.754337	-0.438811	1.905041	C	0.159863	-1.692564	0.969399
O	-6.341688	-1.244034	2.008877	O	-5.623471	0.710114	1.445053	O	-6.170276	-1.002666	2.297348
O	-6.313471	0.017453	-2.343248	O	-5.410911	-3.067947	-1.036122	O	-6.310197	-0.371032	-2.188070
O	0.680599	-0.966331	-2.587055	O	1.593596	-3.276707	-0.098520	O	0.683183	-1.298595	-2.544342
O	0.653193	-2.164568	1.803687	O	1.323497	0.334527	2.649498	O	0.813722	-1.965024	1.958224
N	-6.267793	-0.614427	-0.167202	N	-5.465586	-1.217669	0.270070	N	-6.180094	-0.689216	0.052397
N	0.677194	-1.470047	-0.365510	N	1.468058	-1.366252	1.137202	N	0.763694	-1.533698	-0.279518
H	-7.270187	-0.471730	-0.132601	H	-6.458463	-1.150051	0.080330	H	-7.182204	-0.549065	0.103172
C	2.106931	-1.652008	-0.412096	C	2.874451	-1.119545	0.950232	C	2.200536	-1.633162	-0.344300
C	2.947241	-0.661719	0.031072	C	3.236030	-0.040078	0.173078	C	2.976108	-0.574971	0.061674
C	2.586425	-2.875501	-0.927442	C	3.819381	-1.984141	1.533642	C	2.754778	-2.833026	-0.837698
C	4.360043	-0.893628	-0.036503	C	4.627103	0.213521	-0.026962	C	4.399673	-0.710001	-0.023241
C	3.927780	-3.105423	-0.997464	C	5.151168	-1.753557	1.340209	C	4.108346	-2.970465	-0.923129
C	5.296791	0.077675	0.399663	C	5.081169	1.309033	-0.805362	C	5.272286	0.336077	0.370918
C	4.848980	-2.123397	-0.554841	C	5.589118	-0.653638	0.561623	C	4.965171	-1.915912	-0.519844
C	6.642413	-0.161246	0.322838	C	6.418898	1.528827	-0.989476	C	6.629768	0.190450	0.275242
C	6.247579	-2.341219	-0.620161	C	6.967719	-0.396876	0.351869	C	6.374693	-2.036706	-0.604554
C	7.125937	-1.383883	-0.191985	C	7.374539	0.667505	-0.404542	C	7.189886	-1.008962	-0.216785
C	2.453938	0.636311	0.578515	C	2.186592	0.847911	-0.410685	C	2.386171	0.690660	0.588926
C	2.236111	1.745214	-0.294879	C	1.389719	0.404190	-1.510150	C	1.944779	1.710958	-0.306483
C	2.345000	0.798013	1.952955	C	1.926543	2.074646	0.181524	C	2.361055	0.907963	1.958470

C 2.310678 1.622317 -1.707804	C 1.619016 -0.835455 -2.162545	C 1.948474 1.538442 -1.715835
C 1.937217 3.024667 0.251109	C 0.310611 1.208937 -1.968237	C 1.498288 2.958259 0.212881
C 2.030642 2.083138 2.480606	C 0.865185 2.882706 -0.312707	C 1.902332 2.157958 2.460591
C 2.081312 2.702230 -2.519156	C 0.800811 -1.261290 -3.176849	C 1.533132 2.544844 -2.548583
C 1.709952 4.121095 -0.614969	C -0.512129 0.744918 -3.021735	C 1.085598 3.980137 -0.674573
C 1.854901 3.156983 1.661047	C 0.079814 2.459470 -1.340794	C 1.494656 3.146751 1.617822
C 1.776552 3.967929 -1.973916	C -0.282661 -0.469739 -3.612262	C 1.101455 3.784270 -2.029452
N 2.609639 -0.240241 2.826930	N 2.686767 2.540715 1.243614	N 2.835924 -0.042558 2.848437
H 2.382580 -1.162106 2.474173	H 3.055165 1.804708 1.832553	H 2.682405 -0.997690 2.549629
H 2.258366 -0.102672 3.763003	H 2.222802 3.244060 1.800464	H 2.531373 0.094195 3.801273
H -4.046473 0.002092 -3.462977	H -3.083327 -4.033879 -1.346056	H -4.082351 -0.531960 -3.376827
H -1.540892 -0.331709 -3.538524	H -0.579482 -4.103369 -0.997458	H -1.576365 -0.858273 -3.494338
H -1.583810 -2.186500 2.892458	H -0.962871 1.284597 2.951069	H -1.373942 -1.806556 3.124884
H -4.089337 -1.863368 2.973850	H -3.451398 1.394652 2.575176	H -3.876879 -1.473050 3.255654
H 1.869666 -3.615258 -1.265426	H 3.468512 -2.824102 2.120597	H 2.086752 -3.627913 -1.148609
H 4.305717 -4.042398 -1.394006	H 5.891864 -2.412992 1.781374	H 4.545174 -3.888398 -1.303435
H 4.928781 1.015993 0.796227	H 4.345718 1.968894 -1.249774	H 4.843728 1.255857 0.749510
H 7.344633 0.593739 0.660491	H 6.751185 2.370701 -1.587799	H 7.282523 1.001762 0.579978
H 6.608444 -3.284782 -1.018449	H 7.695349 -1.064384 0.803798	H 6.794942 -2.962561 -0.985896
H 8.195154 -1.560265 -0.246688	H 8.432304 0.854181 -0.558703	H 8.267906 -1.111134 -0.286654
H 2.521875 0.653739 -2.143628	H 2.442639 -1.459923 -1.838090	H 2.270927 0.592364 -2.132999
H 1.953854 2.197994 3.557801	H 0.670720 3.838402 0.165449	H 1.892578 2.314639 3.535471
H 2.130662 2.580290 -3.596259	H 0.992561 -2.218830 -3.650477	H 1.541121 2.385895 -3.622177
H 1.479859 5.089236 -0.178426	H -1.333149 1.374701 -3.351156	H 0.751875 4.925820 -0.256632
H 1.631989 4.132446 2.084717	H -0.749776 3.071498 -1.678156	H 1.151365 4.096163 2.018103
H 1.595756 4.811946 -2.630853	H -0.922569 -0.820080 -4.415230	H 0.781097 4.572590 -2.702426
C -1.233061 2.198450 0.798391	C -4.105837 3.035251 -0.183837	C -2.073986 2.242735 0.378530
Cl -2.365587 2.409548 -0.555379	Cl -2.745713 3.827262 0.651419	S -2.173458 2.431908 -1.158037
Cl -2.040676 1.676067 2.296411	H -4.710791 2.513522 0.554949	S -1.970397 2.049237 1.917923
H -0.747433 3.149088 0.985690	H -4.674148 3.803068 -0.701396	
H -0.496010 1.447191 0.524593	Cl -3.570026 1.839122 -1.391855	
cs2-a2	cs2-a3	cyhex-a1
C 3.277272 -1.211375 2.407948	C -2.798694 -1.512910 -2.233052	C -3.141526 -1.683922 -2.362463
C 1.876818 -1.330062 2.451394	C -1.401495 -1.611176 -2.353448	C -1.737972 -1.768593 -2.377456
C 1.151552 -1.398434 1.284723	C -0.611266 -1.644806 -1.228629	C -1.030717 -1.735328 -1.198070
C 1.069926 -1.405612 -1.172825	C -0.392386 -1.622752 1.221555	C -0.989629 -1.591091 1.256438
C 1.717800 -1.347585 -2.384934	C -0.971670 -1.557696 2.467203	C -1.659234 -1.492985 2.453914
C 3.117559 -1.226363 -2.434323	C -2.367410 -1.445842 2.593181	C -3.062778 -1.412160 2.475132
C 3.849832 -1.169041 -1.271959	C -3.163669 -1.409071 1.472783	C -3.776106 -1.431790 1.299387
C 3.931792 -1.164443 1.199708	C -3.383903 -1.448444 -0.990801	C -3.816081 -1.571150 -1.169618
C 3.206351 -1.229721 -0.013363	C -2.591862 -1.488181 0.181051	C -3.110623 -1.551220 0.056607
C 1.801899 -1.347841 0.033126	C -1.191257 -1.589462 0.057517	C -1.703056 -1.632494 0.038778
C 5.410772 -1.037864 1.164165	C -4.856076 -1.301596 -0.874967	C -5.295993 -1.451828 -1.166742
C 5.328437 -1.042807 -1.335477	C -4.634551 -1.269394 1.616581	C -5.255742 -1.310277 1.331135
C -0.325225 -1.521452 1.348149	C 0.864013 -1.682046 -1.374713	C 0.451090 -1.783346 -1.233989
C -0.408974 -1.519463 -1.137955	C 1.084849 -1.689692 1.103862	C 0.493791 -1.623983 1.247276
O 5.954517 -0.981030 -2.372517	O -5.203153 -1.183679 2.684769	O -5.900879 -1.201522 2.352487
O 6.105551 -0.970305 2.155973	O -5.604958 -1.229623 -1.826801	O -5.975181 -1.459830 -2.171287
O -0.925029 -1.619882 2.397560	O 1.410499 -1.739397 -2.456169	O 1.072409 -1.913882 -2.267318
O -1.071104 -1.613903 -2.153801	O 1.809002 -1.785181 2.075140	O 1.144063 -1.618630 2.275249
N 5.972779 -1.005284 -0.106061	N -5.345207 -1.236520 0.423608	N -5.877523 -1.323058 0.089056
N -1.009817 -1.515709 0.122887	N 1.611686 -1.606412 -0.189853	N 1.113564 -1.658542 -0.003389
H 6.980957 -0.911877 -0.139566	H -6.347432 -1.116100 0.510318	H -3.714447 -1.693669 -3.282542
C -2.450029 -1.553606 0.175865	C 3.018854 -1.328527 -0.317393	H -1.189021 -1.845155 -3.308618
C -3.170240 -0.415767 -0.097172	C 3.468994 -0.074428 0.030955	H -1.081761 -1.463178 3.370251
C -3.066358 -2.775429 0.516967	C 3.875851 -2.329852 -0.813796	H -3.605894 -1.319898 3.408663
C -4.598660 -0.488676 -0.035082	C 4.863354 0.207334 -0.109486	C 2.554677 -1.630739 -0.036839
C -4.425969 -2.853994 0.582264	C 5.207524 -2.067780 -0.955933	C 3.220694 -0.443727 0.147431
C -5.415456 0.639554 -0.302092	C 5.407943 1.472439 0.229732	C 3.227769 -2.848637 -0.269971
C -5.226572 -1.717006 0.308497	C 5.735313 -0.799333 -0.608787	C 4.652729 -0.459737 0.100617
C -6.779185 0.550351 -0.229976	C 6.744769 1.722053 0.079494	C 4.589712 -2.873481 -0.317277
C -6.641085 -1.777191 0.373289	C 7.115653 -0.508921 -0.751012	H 2.642271 -3.749086 -0.415290
C -7.401512 -0.670944 0.110631	C 7.610563 0.721121 -0.415399	C 5.417968 0.721175 0.277060
C -2.502715 0.869009 -0.458009	C 2.525584 0.949047 0.571443	C 5.337146 -1.683309 -0.134164
C -1.954345 1.709149 0.557174	C 1.592813 1.608166 -0.284974	H 5.117087 -3.804698 -0.498216
C -2.463152 1.265152 -1.786654	C 2.523295 1.215835 1.932878	C 6.785065 0.686099 0.222214
C -1.965545 1.347657 1.930483	C 1.547602 1.372786 -1.684752	H 4.899861 1.655249 0.456005
C -1.375320 2.958179 0.200270	C 0.655604 2.524957 0.266477	C 6.753461 -1.687167 -0.183287
C -1.888096 2.521866 -2.124942	C 1.603243 2.162646 2.462192	C 7.463115 -0.530790 -0.009422
C -1.409091 2.166213 2.877848	C 0.604273 1.978691 -2.473068	H 7.354229 1.599725 0.358218
C -0.812284 3.782856 1.203273	C -0.297059 3.145663 -0.576085	H 7.264481 -2.628218 -0.363575
C -1.371402 3.339524 -1.165079	C 0.699041 2.787828 1.658695	H 8.547369 -0.544305 -0.049759
C -0.821196 3.397695 2.516962	C -0.335546 2.873578 -1.918317	C 2.508913 0.843567 0.402419
N -3.023349 0.487180 -2.788390	N 3.417361 0.595448 2.791521	C 1.986295 1.611422 -0.681485
H -2.952018 -0.508286 -2.617974	H 3.671420 -0.341152 2.504000	C 2.443481 1.335179 1.698242
H -2.703029 0.718387 -3.717673	H 3.131945 0.607864 3.760121	C 2.008597 1.145391 -2.022742
H 3.864068 -1.153031 3.317439	H -3.436409 -1.471442 -3.108570	C 1.421671 2.892607 -0.428497
H 1.344757 -1.367028 3.394715	H -0.921473 -1.645255 -3.324322	C 1.880319 2.620889 1.931733
H 1.126373 -1.392635 -3.291753	H -0.329045 -1.581932 3.339261	C 1.477937 1.897795 -3.037730
H 3.643710 -1.174395 -3.380571	H -2.840169 -1.379970 3.566400	H 2.432896 0.172630 -2.238684
H -2.441021 -3.634570 0.730040	H 3.456226 -3.291406 -1.083336	C 0.888323 3.647761 -1.499842
H -4.910663 -3.788432 0.846737	H 5.878780 -2.829455 -1.339753	C 1.400105 3.374883 0.904249
H -4.939002 1.576152 -0.564744	H 4.743697 2.238593 0.610859	H 1.848319 2.997657 2.950051
H -7.389026 1.423675 -0.436272	H 7.145550 2.695369 0.342502	C 0.908525 3.163481 -2.780804
H -7.109482 -2.720567 0.637593	H 7.772842 -1.283963 -1.133922	H 1.497188 1.513832 -4.052607
H -8.483825 -0.727421 0.163848	H 8.668738 0.932866 -0.528738	H 0.457789 4.621581 -1.283366

H -2.403202 0.402474 2.226410	H 2.260000 0.688777 -2.129558	H 0.974678 4.354298 1.100629
H -1.874431 2.823764 -3.168360	H 1.623862 2.367312 3.528761	H 0.493242 3.747178 -3.595545
H -1.421658 1.861776 3.919461	H 0.584593 1.769878 -3.537828	N 2.977607 0.628723 2.764165
H -0.368876 4.729415 0.907173	H -1.002106 3.844605 -0.134743	H 2.902521 -0.376612 2.669327
H -0.934666 4.294909 -1.440994	H -0.010174 3.493343 2.081484	H 2.651580 0.935507 3.669095
H -0.383522 4.033889 3.278939	H -1.075499 3.348650 -2.553651	C -2.360739 1.787070 -0.862441
C 3.201711 2.242556 -0.354830	C -5.173684 2.059184 -0.372557	C -1.794216 1.782587 0.557400
S 1.799303 2.144875 -1.010509	S -6.725869 2.050095 -0.310129	C -2.071939 3.111449 1.258701
S 4.603818 2.337683 0.316160	S -3.619444 2.054425 -0.440641	C -3.571808 3.415389 1.276703
		C -4.159640 3.401103 -0.136843
		C -3.861632 2.080711 -0.850948
		H -1.669087 3.097145 2.277012
		H -2.270408 0.980451 1.133090
		H -0.721891 1.571522 0.543159
		H -1.840468 2.553393 -1.449894
		H -2.160777 0.834861 -1.360185
		H -4.082395 2.655602 1.883978
		H -3.763058 4.380562 1.757690
		H -5.239097 3.582416 -0.102325
		H -3.720455 4.224611 -0.715361
		H -4.382974 1.267897 -0.325776
		H -4.259898 2.097727 -1.871018
		H -1.545551 3.913346 0.724614
		H -6.886881 -1.236144 0.100253
cyhex-a2	cyhex-a3	cyhex-a4
C -3.287126 -1.466981 -2.441915	C -2.683633 -2.514530 -1.984962	C 3.288469 -1.250478 2.484925
C -1.882399 -1.520557 -2.481639	C -1.277644 -2.526234 -1.966967	C 1.882758 -1.286123 2.504398
C -1.160216 -1.611278 -1.314252	C -0.597752 -2.143526 -0.834834	C 1.176993 -1.388259 1.328347
C -1.090640 -1.753742 1.139603	C -0.613592 -1.338215 1.489202	C 1.141103 -1.546014 -1.125418
C -1.745626 -1.789199 2.348766	C -1.312227 -0.946568 2.607973	C 1.814796 -1.612371 -2.323448
C -3.148863 -1.714673 2.395415	C -2.717699 -0.922697 2.591742	C 3.220408 -1.585722 -2.347972
C -3.876661 -1.616445 1.232656	C -3.404037 -1.297183 1.460509	C 3.932628 -1.493770 -1.175372
C -3.947925 -1.499573 -1.236666	C -3.387226 -2.120465 -0.871797	C 3.967331 -1.324315 1.291847
C -3.226424 -1.595810 -0.023479	C -2.708902 -1.725979 0.306113	C 3.263034 -1.423871 0.068598
C -1.818429 -1.657755 -0.066436	C -1.299306 -1.738412 0.321370	C 1.853130 -1.450293 0.090250
C -5.429481 -1.410646 -1.204118	C -4.870521 -2.088017 -0.909669	C 5.451739 -1.293290 1.283522
C -5.357592 -1.523132 1.294226	C -4.885614 -1.218162 1.435701	C 5.416409 -1.454617 -1.213563
C 0.321629 -1.659267 -1.372720	C 0.884876 -2.132330 -0.843478	C -0.304364 -1.465113 1.368969
C 0.391580 -1.800027 1.108894	C 0.870407 -1.310937 1.504961	C -0.342418 -1.606355 -1.113556
O -5.990270 -1.529217 2.329046	O -5.554103 -0.813546 2.364659	O 6.065375 -1.501181 -2.237109
O -6.122052 -1.327851 -2.196221	O -5.529874 -2.422032 -1.871094	O 6.130367 -1.216053 2.285546
O 0.930809 -1.665082 -2.421264	O 1.532302 -2.509757 -1.797204	O -0.923901 -1.483610 2.411140
O 1.051845 -1.923240 2.123230	O 1.499973 -1.010484 2.500424	O -0.986887 -1.733033 -2.137356
N -5.996072 -1.424083 0.064740	N -5.482427 -1.628819 0.251413	N 6.038577 -1.359251 0.025185
N 0.998319 -1.699774 -0.144189	N 1.514934 -1.636354 0.307912	N -0.966434 -1.537900 0.133526
H -3.871631 -1.388401 -3.351362	H -3.236172 -2.805814 -2.870846	H 3.860297 -1.168413 3.402071
H -1.344743 -1.486078 -3.421909	H -0.706341 -2.825157 -2.837849	H 1.332126 -1.238951 3.436526
H -1.157187 -1.862910 3.255667	H -0.755788 -0.643848 3.487035	H 1.239429 -1.688439 -3.238489
H -3.680844 -1.727303 3.339757	H -3.283744 -0.593437 3.455401	H 3.766430 -1.632184 -3.283141
C 2.439662 -1.712003 -0.188851	C 2.938889 -1.428192 0.237033	C -2.403921 -1.660888 0.169343
C 3.145519 -0.572822 0.110574	C 3.419739 -0.141768 0.159073	C -3.202626 -0.585231 -0.129383
C 3.071403 -2.921580 -0.547658	C 3.785263 -2.555128 0.232027	C -2.933579 -2.921082 0.521685
C 4.575930 -0.631341 0.055987	C 4.835907 0.046284 0.076674	C -4.623498 -0.765123 -0.079700
C 4.431730 -2.986497 -0.604581	C 5.136198 -2.385447 0.151538	C -4.283408 -3.101631 0.573463
H 2.456038 -3.783354 -0.779185	H 3.341923 -3.542354 0.284019	H -2.248797 -3.729149 0.752616
C 5.379792 0.499663 0.348866	C 5.414068 1.338615 -0.008456	C -5.518923 0.295101 -0.371480
C 5.218919 -1.846693 -0.304681	C 5.696093 -1.085999 0.072011	C -5.163152 -2.031316 0.274777
H 4.927334 -3.911497 -0.881679	H 5.798437 -3.245347 0.144534	H -4.699588 -4.066424 0.845511
C 6.744966 0.425146 0.285632	C 6.770763 1.495099 -0.093945	C -6.873201 0.105673 -0.312929
H 4.892710 1.426982 0.624265	H 4.759885 2.201843 -0.004961	H -5.110842 1.261074 -0.642201
C 6.634274 -1.892211 -0.359636	C 7.097265 -0.890002 -0.015457	C -6.569692 -2.196327 0.324943
C 7.381959 -0.783533 -0.071605	C 7.624307 0.369518 -0.096358	C -7.407283 -1.153533 0.038135
H 7.344357 1.300698 0.512100	H 7.196402 2.490915 -0.159741	H -7.543455 0.928520 -0.538391
H 7.113774 -2.826299 -0.636725	H 7.744583 -1.761886 -0.018699	H -6.969521 -3.168505 0.597414
H 8.465142 -0.828646 -0.117300	H 8.698365 0.508363 -0.163778	H -8.482959 -1.289862 0.080159
C 2.476864 0.706678 0.489288	C 2.498620 1.032498 0.186620	C -2.644571 0.746561 -0.503089
C 2.010940 1.601783 -0.519968	C 1.726192 1.384614 -0.961116	C -2.248521 1.671579 0.509146
C 2.402699 1.065786 1.827058	C 2.392938 1.780638 1.349996	C -2.606571 1.116041 -1.839112
C 2.054266 1.275753 -1.901775	C 1.786082 0.647910 -2.173672	C -2.266275 1.339984 1.889969
C 1.493740 2.874013 -0.148810	C 0.852473 2.505460 -0.905006	C -1.829222 2.979295 0.140762
C 1.872058 2.337853 2.180844	C 1.542108 2.919872 1.377074	C -2.184142 2.428837 -2.189255
C 1.595559 2.155176 -2.846900	C 0.993217 0.980205 -3.240963	C -1.870468 2.248143 2.836485
H 2.436227 0.309180 -2.205862	H 2.456421 -0.199531 -2.246347	H -2.576569 0.347711 2.192377
C 1.040056 3.764875 -1.150848	C 0.053310 2.828623 -2.027496	C -1.432386 3.896127 1.142923
C 1.445877 3.212305 1.227338	C 0.801304 3.268007 0.288963	C -1.818689 3.327284 -1.233433
H 1.825972 2.608250 3.231922	H 1.486205 3.506190 2.289728	H -2.166497 2.707120 -3.239149
C 1.082895 3.416617 -2.474692	C 0.109818 2.078895 -3.172389	C -1.446968 3.542759 2.466090
H 1.629593 1.877614 -3.895464	H 1.049728 0.391547 -4.150972	H -1.883764 1.966831 3.884554
H 0.654062 4.733995 -0.848011	H -0.613684 3.683713 -1.960340	H -1.111683 4.888811 0.839795
H 1.053775 4.182748 1.516228	H 0.148790 4.135173 0.329876	H -1.500142 4.325546 -1.518380
H 0.728917 4.104853 -3.234947	H -0.512414 2.330359 -4.024740	H -1.138776 4.251837 3.227239
N 2.897373 0.240287 2.824161	N 3.133047 1.465476 2.479548	N -3.034685 0.255076 -2.838101
H 2.799877 -0.748192 2.627439	H 3.295585 0.472694 2.592077	H -2.844083 -0.720857 -2.646874
H 2.563662 0.463136 3.750574	H 2.772932 1.869803 3.331957	H -2.725571 0.511375 -3.764490
C -2.833113 3.177265 -1.160005	C -4.171835 2.657396 -1.511451	C 2.238406 3.371264 0.813272
C -2.914556 1.821673 -0.456584	C -3.278634 1.543428 -0.964819	C 1.598969 3.379958 -0.576035

C	-2.011655	1.774501	0.777322	C	-2.612814	1.964608	0.345195	C	2.649709	3.283928	-1.682371
C	-2.303321	2.930013	1.736198	C	-3.646024	2.395910	1.387253	C	3.530411	2.048703	-1.490031
C	-2.230681	4.283002	1.026043	C	-4.546027	3.506463	0.840277	C	4.182099	2.050306	-0.105852
C	-3.150438	4.321914	-0.195917	C	-5.210923	3.085967	-0.472686	C	3.129863	2.142566	1.000792
H	-2.134004	0.822438	1.300899	H	-1.981348	1.159709	0.732313	H	2.165345	3.257235	-2.664412
H	-3.954362	1.633473	-0.152802	H	-3.898771	0.656219	-0.785017	H	0.910415	2.530290	-0.658126
H	-2.640684	1.024099	-1.153191	H	-2.518720	1.258314	-1.699394	H	0.987614	4.278620	-0.705640
H	-1.818560	3.309670	-1.556007	H	-3.546509	3.521430	-1.774635	H	2.845546	4.278025	0.937875
H	-3.514331	3.200498	-2.017373	H	-4.664867	2.334222	-2.434318	H	1.459245	3.399469	1.580991
H	-3.308513	2.801536	2.159969	H	-4.269479	1.538372	1.670243	H	2.906297	1.151859	-1.591082
H	-1.600491	2.903942	2.575894	H	-3.143991	2.728872	2.302393	H	4.294097	1.989576	-2.273003
H	-2.481972	5.091891	1.720554	H	-5.305005	3.781239	1.580080	H	4.803210	1.158769	0.025222
H	-1.199107	4.455987	0.697272	H	-3.939748	4.404831	0.661876	H	4.860584	2.909880	-0.029676
H	-4.193923	4.236268	0.135997	H	-5.887108	2.242903	-0.276198	H	2.501574	1.243287	0.976525
H	-3.062707	5.286488	-0.707394	H	-5.830480	3.899706	-0.864074	H	3.610386	2.161093	1.984781
H	-0.964782	1.826725	0.456938	H	-1.938785	2.803979	0.142114	H	3.280317	4.182768	-1.665545
H	-7.006637	-1.359182	0.096644	H	-6.494124	-1.579899	0.227180	H	7.051256	-1.331670	0.009069
dmso-a1				dmso-a2				dmso-a3			
C	-3.282378	-1.141185	-2.429268	C	3.721628	0.388308	2.360854	C	3.192719	0.927312	2.504115
C	-1.884703	-1.285903	-2.498483	C	2.323590	0.233115	2.391636	C	1.810454	0.673446	2.470867
C	-1.143254	-1.418928	-1.346352	C	1.677816	-0.425932	1.372218	C	1.299044	-0.250133	1.588163
C	-1.037765	-1.575090	1.109240	C	1.759123	-1.671930	-0.738419	C	1.637430	-1.893696	-0.206640
C	-1.676761	-1.576865	2.328328	C	2.479054	-2.186510	-1.789301	C	2.477586	-2.580070	-1.048224
C	-3.072724	-1.418691	2.398285	C	3.875058	-2.017859	-1.840606	C	3.863300	-2.348927	-1.004781
C	-3.809582	-1.265708	1.249557	C	4.531549	-1.348913	-0.834666	C	4.388476	-1.429845	-0.126381
C	-3.916763	-1.125486	-1.211030	C	4.453498	-0.120584	1.312592	C	4.043989	0.247313	1.662341
C	-3.179308	-1.284929	-0.015681	C	3.810222	-0.818630	0.262210	C	3.540475	-0.713932	0.753979
C	-1.780236	-1.433942	-0.085211	C	2.411475	-0.978875	0.302651	C	2.151928	-0.957409	0.714608
C	-5.389814	-0.925241	-1.144769	C	5.924655	0.065986	1.276592	C	5.500716	0.529244	1.697926
C	-5.282506	-1.071477	1.334998	C	6.003325	-1.176986	-0.895624	C	5.848748	-1.173542	-0.105880
C	0.332587	-1.506310	-1.429361	C	0.196620	-0.560262	1.406465	C	-0.165278	-0.502317	1.566246
C	0.435950	-1.701097	1.051890	C	0.290134	-1.881050	-0.682504	C	0.173291	-2.113969	-0.296313
O	-5.883780	-1.017285	2.386749	O	6.698022	-1.594938	-1.798482	O	6.647100	-1.738400	-0.824017
O	-6.078667	-0.745450	-2.126746	O	6.552891	0.655433	2.131660	O	6.013576	1.344214	2.436254
O	0.931616	-1.494020	-2.484975	O	-0.471475	-0.070582	2.292196	O	-0.920743	0.020864	2.355807
O	1.110925	-1.884128	2.047940	O	-0.291430	-2.538743	-1.527117	O	-0.314976	-2.895525	-1.088618
N	-5.945474	-1.006143	0.120917	N	6.563454	-0.484069	0.172204	N	6.267664	-0.208358	0.804311
N	1.025765	-1.585143	-0.210425	N	-0.389100	-1.343016	0.405575	N	-0.632376	-1.390101	0.588517
H	-3.879872	-1.017839	-3.325135	H	4.246315	0.910954	3.152716	H	3.613432	1.657001	3.186621
H	-1.365042	-1.277834	-3.449651	H	1.731158	0.635507	3.205413	H	1.124723	1.197520	3.126375
H	-1.079484	-1.691627	3.225380	H	1.949700	-2.719182	-2.570287	H	2.046631	-3.281167	-1.752570
H	-3.589926	-1.400052	3.350571	H	4.457341	-2.412204	-2.665669	H	4.540627	-2.878110	-1.665436
C	2.464768	-1.587815	-0.276509	C	-1.788993	-1.666914	0.549092	C	-2.052238	-1.641649	0.545452
C	3.164593	-0.464309	0.091123	C	-2.779900	-0.877616	0.017861	C	-2.908228	-0.708941	0.010779
C	3.103027	-2.760477	-0.731856	C	-2.076268	-2.846194	1.273070	C	-2.506804	-2.868511	1.074890
C	4.594166	-0.499469	0.007084	C	-4.141833	-1.285937	0.211892	C	-4.308617	-1.015167	-0.011950
C	4.462800	-2.803735	-0.816797	C	-3.366437	-3.237984	1.468401	C	-3.837391	-3.162595	1.069182
H	2.492798	-3.610109	-1.014908	H	-1.248731	-3.425425	1.667739	H	-1.779417	-3.563038	1.479247
C	5.392067	0.617476	0.363729	C	-5.223751	-0.532941	-0.312622	C	-5.258722	-0.120080	-0.567938
C	5.242995	-1.678437	-0.451163	C	-4.433879	-2.468788	0.943352	C	-4.772414	-2.246294	0.526583
H	4.963551	-3.700357	-1.167968	H	-3.590072	-4.142017	2.025974	H	-4.195307	-4.102839	1.476408
C	6.756380	0.564100	0.269141	C	-6.518535	-0.931237	-0.115656	C	-6.592402	-0.428419	-0.578198
H	4.902022	1.517879	0.713226	H	-5.006708	0.365764	-0.876392	H	-4.910607	0.814955	-0.988894
C	6.657444	-1.702895	-0.535981	C	-5.784305	-2.854978	1.131057	C	-6.159601	-2.534989	0.503796
C	7.399118	-0.608631	-0.184584	C	-6.805224	-2.104618	0.615453	C	-7.051265	-1.647688	-0.033670
H	7.350741	1.428756	0.545309	H	-7.332302	-0.341974	-0.525277	H	-7.303661	0.268336	-1.009231
H	7.141046	-2.609298	-0.887727	H	-5.992188	-3.760688	1.693043	H	-6.500562	-3.478060	0.920488
H	8.481564	-0.637428	-0.253971	H	-7.836116	-2.408706	0.764791	H	-8.111372	-1.878833	-0.046574
C	2.486826	0.773247	0.580373	C	-2.511218	0.372812	-0.749484	C	-2.420332	0.585182	-0.547282
C	1.955694	1.722915	-0.342153	C	-2.592969	1.637909	-0.098314	C	-2.627892	1.801372	0.175976
C	2.465069	1.040320	1.942676	C	-2.309734	0.308405	-2.118851	C	-1.805793	0.609440	-1.790002
C	1.942813	1.494052	-1.744502	C	-2.814218	1.757465	1.299952	C	-3.216658	1.823852	1.467590
C	1.432408	2.956709	0.137627	C	-2.477503	2.834603	-0.858947	C	-2.221674	3.039952	-0.391775
C	1.926383	2.273250	2.405911	C	-2.166691	1.515015	-2.860312	C	-1.428353	1.864216	-2.352331
C	1.442277	2.433540	-2.607342	C	-2.930886	2.987678	1.893806	C	-3.397445	3.003166	2.140132
H	2.322148	0.556023	-2.131075	H	-2.862415	0.858278	1.900844	H	-3.497591	0.886856	1.931053
C	0.934079	3.912257	-0.780874	C	-2.600410	4.089955	-0.216566	C	-2.423658	4.242490	0.326865
C	1.440714	3.200704	1.534331	C	-2.253239	2.730274	-2.255732	C	-1.628210	3.031363	-1.681032
H	1.925346	2.471474	3.473901	H	-2.004021	1.447321	-3.932217	H	-0.998862	1.864388	-3.348579
C	0.937820	3.662394	-2.128241	C	-2.829785	4.172645	1.131777	C	-3.002541	4.231334	1.567428
H	1.439912	2.231509	-3.673462	H	-3.097431	3.050934	2.964385	H	-3.844330	2.992034	3.129068
H	0.554661	4.854823	-0.394798	H	-2.513051	4.990410	-0.818561	H	-2.110964	5.177866	-0.129382
H	1.050331	4.144515	1.905112	H	-2.155022	3.640057	-2.840856	H	-1.341182	3.977051	-2.132942
H	0.558822	4.402338	-2.825497	H	-2.927272	5.139093	1.615223	H	-3.155024	5.157578	2.111523
N	3.017179	0.160388	2.856738	N	-2.320552	-0.892095	-2.805348	N	-1.573843	-0.519449	-2.555566
H	2.925539	-0.813703	2.595485	H	-2.005056	-1.698224	-2.279626	H	-1.733071	-1.402349	-2.089514
H	2.726014	0.312870	3.811266	H	-1.885526	-0.856033	-3.715286	H	-0.650967	-0.502584	-2.977553
H	-6.938683	-0.816741	0.175109	H	7.568659	-0.362629	0.138901	H	7.264328	-0.027536	0.825810
S	-3.373622	2.539770	0.169449	S	0.988335	2.549337	-0.899027	S	1.870570	1.559441	-2.445277
C	-2.093181	2.110158	1.376438	C	0.699140	3.145936	0.784994	C	1.814458	2.375054	-0.830463
H	-2.533106	2.205747	2.369602	H	-0.360484	3.023012	1.000213	H	0.765550	2.442161	-0.541722
H	-1.765335	1.082153	1.217852	H	1.286804	2.556133	1.488605	H	2.373000	1.795543	-0.095765
H	-1.247465	2.791189	1.269910	H	0.970559	4.202204	0.840684	H	2.234768	3.378599	-0.926935
C	-2.374146	2.219859	-1.304470	C	2.801554	2.644308	-0.834846	C	3.671909	1.389778	-2.530912
H	-1.968209	1.208766	-1.271624	H	3.171242	2.054284	0.005834	H	4.016333	0.803335	-1.677662
H	-3.032041	2.322870	-2.168107	H	3.180917	2.229644	-1.769629	H	3.907876	0.861160	-3.454840

H -1.561787 2.946349 -1.359166	H 3.113242 3.686664 -0.739375	H 4.136711 2.377839 -2.538501
O -4.437471 1.467324 0.238527	O 0.641306 1.079285 -0.920087	O 1.322003 0.158662 -2.270148
dmso-a4	dmso-a5	dmso-a6
C 3.181637 -0.403000 2.457850	C -3.310630 -1.117894 -2.393602	C -2.991565 -2.193393 -2.020738
C 1.785164 -0.508021 2.591789	C -1.910553 -1.227599 -2.481709	C -1.586691 -2.273435 -1.995384
C 1.028221 -1.014928 1.560092	C -1.153892 -1.385602 -1.344673	C -0.891045 -1.918336 -0.863312
C 0.884922 -1.973446 -0.702996	C -1.013617 -1.599433 1.103806	C -0.883649 -1.098930 1.457606
C 1.507855 -2.407078 -1.850376	C -1.635896 -1.641713 2.331303	C -1.571929 -0.660234 2.566042
C 2.908526 -2.340368 -1.968100	C -3.033354 -1.508542 2.422595	C -2.973755 -0.548253 2.529780
C 3.664574 -1.829360 -0.942344	C -3.789235 -1.350541 1.285401	C -3.666227 -0.887037 1.391253
C 3.799541 -0.817692 1.301761	C -3.932855 -1.166114 -1.171011	C -3.678901 -1.749990 -0.918056
C 3.046874 -1.380411 0.247201	C -3.176592 -1.339334 0.011944	C -2.984431 -1.376310 0.255268
C 1.646450 -1.462742 0.371891	C -1.774347 -1.447726 -0.077036	C -1.579173 -1.468063 0.284961
C 5.267963 -0.645893 1.141179	C -5.407113 -0.994399 -1.082804	C -5.163232 -1.669760 -0.949959
C 5.143452 -1.749407 -1.078028	C -5.261885 -1.166627 1.395163	C -5.142444 -0.715079 1.339980
C -0.446887 -1.060042 1.689055	C 0.319643 -1.458441 -1.450280	C 0.587330 -1.991974 -0.857060
C -0.591352 -2.018500 -0.604691	C 0.462944 -1.694338 1.025554	C 0.596525 -1.166143 1.492960
O 5.743587 -2.121401 -2.063229	O -5.846758 -1.125933 2.457626	O -5.773840 -0.171963 2.225460
O 5.953733 -0.047341 1.946073	O -6.117479 -0.829865 -2.051455	O -5.828202 -1.977978 -1.915146
O -1.026931 -0.719257 2.699175	O 0.903961 -1.426651 -2.513857	O 1.227533 -2.400662 -1.803367
O -1.288739 -2.470607 -1.493971	O 1.153882 -1.877786 2.010590	O 1.232917 -0.898725 2.495094
N 5.819164 -1.253059 0.028791	N -5.941636 -1.081744 0.193603	N -5.765166 -1.254758 0.231577
N -1.158886 -1.502445 0.564237	N 1.033098 -1.552989 -0.242645	N 1.233841 -1.543509 0.308051
H 3.791279 0.017356 3.249736	H -3.920139 -0.976487 -3.278594	H -3.553866 -2.469877 -2.905049
H 1.278747 -0.176870 3.491021	H -1.402830 -1.177727 -3.437950	H -1.028338 -2.611177 -2.860723
H 0.896081 -2.793973 -2.656987	H -1.024624 -1.763158 3.218000	H -1.009203 -0.393008 3.452877
H 3.413535 -2.675488 -2.866582	H -3.537641 -1.512237 3.382018	H -3.530023 -0.184718 3.386432
C -2.596444 -1.433418 0.630907	C 2.470784 -1.559037 -0.335641	C 2.673163 -1.490248 0.278164
C -3.254341 -0.409521 -0.006339	C 3.186341 -0.452692 0.053609	C 3.302132 -0.270104 0.203943
C -3.276126 -2.428650 1.363162	C 3.092037 -2.719577 -0.843411	C 3.385731 -2.706494 0.315285
C -4.682666 -0.366432 0.077863	C 4.613710 -0.493160 -0.064567	C 4.733134 -0.246774 0.168325
C -4.636302 -2.394470 1.452836	C 4.449131 -2.768257 -0.959862	C 4.748736 -2.694981 0.280202
H -2.697510 -3.205422 1.849450	H 2.470321 -3.555542 -1.141608	H 2.830223 -3.635587 0.363061
C -5.436803 0.655380 -0.553243	C 5.427054 0.607059 0.308891	C 5.459444 0.968812 0.088506
C -5.374308 -1.366618 0.814308	C 5.244550 -1.659918 -0.575862	C 5.457127 -1.469789 0.206011
H -5.169610 -3.154003 2.015650	H 4.935949 -3.655846 -1.351279	H 5.307044 -3.625401 0.305832
C -6.801679 0.681166 -0.455457	C 6.788421 0.549320 0.180584	C 6.827329 0.967187 0.048123
H -4.911591 1.417285 -1.116308	H 4.951271 1.498627 0.698304	H 4.910569 1.902053 0.059178
C -6.788208 -1.312569 0.895688	C 6.656476 -1.689689 -0.694355	C 6.873534 -1.437672 0.164844
C -7.487493 -0.313237 0.276413	C 7.413178 -0.611677 -0.325321	C 7.545100 -0.248597 0.087909
H -7.363298 1.470432 -0.944140	H 7.394331 1.401490 0.470175	H 7.366700 1.906525 -0.014358
H -7.305892 -2.081511 1.461322	H 7.125831 -2.586958 -1.086522	H 7.415438 -2.378263 0.193751
H -8.569892 -0.280894 0.345355	H 8.493526 -0.644283 -0.420887	H 8.629627 -0.235209 0.055918
C -2.525265 0.638731 -0.780026	C 2.527179 0.768047 0.605415	C 2.531467 1.008084 0.177793
C -1.993303 1.785371 -0.118390	C 1.927114 1.729101 -0.260392	C 1.892670 1.449426 -1.019213
C -2.440575 0.530351 -2.160893	C 2.567217 0.995883 1.974594	C 2.465446 1.782958 1.326961
C -2.052597 1.942994 1.292679	C 1.833286 1.533458 -1.664818	C 1.913881 0.686482 -2.217339
C -1.392392 2.824884 -0.881557	C 1.401671 2.935778 0.281545	C 1.202087 2.693058 -1.032692
C -1.814474 1.568865 -2.906018	C 2.040502 2.208652 2.498882	C 1.788221 3.033089 1.290746
C -1.546213 3.062620 1.898369	C 1.231457 2.465449 -2.468322	C 1.256739 1.117858 -3.339265
H -2.487964 1.154694 1.894191	H 2.226192 0.622830 -2.099745	H 2.439995 -0.259738 -2.233748
C -0.894488 3.977397 -0.226998	C 0.801282 3.887965 -0.578643	C 0.543784 3.117057 -2.212418
C -1.316330 2.676640 -2.289211	C 1.489113 3.147915 1.680563	C 1.184544 3.472393 0.150927
H -1.752252 1.468623 -3.985827	H 2.093287 2.378358 3.570457	H 1.762567 3.636875 2.193474
C -0.964601 4.099139 1.135619	C 0.707985 3.660064 -1.926283	C 0.558092 2.344909 -3.343072
H -1.598510 3.155140 2.978318	H 1.161405 2.284259 -3.535812	H 1.276125 0.507913 -4.236511
H -0.454652 4.766599 -0.831190	H 0.419980 4.809387 -0.145600	H 0.020458 4.069431 -2.201104
H -0.852191 3.464109 -2.876059	H 1.097444 4.071472 2.097528	H 0.674867 4.432086 0.139465
H -0.582870 4.987065 1.628954	H 0.247274 4.395421 -2.577893	H 0.043854 2.673820 -4.239803
N -3.005220 -0.537635 -2.837310	N 3.164164 0.090955 2.835313	N 3.090871 1.386533 2.498752
H -2.985662 -1.410172 -2.323655	H 3.055518 -0.874307 2.548843	H 3.094376 0.382977 2.633938
H -2.663073 -0.661671 -3.779028	H 2.914190 0.213818 3.805776	H 2.756065 1.860443 3.325163
H 6.812189 -1.104654 -0.102705	H -6.937396 -0.912219 0.263291	H -6.764882 -1.103656 0.175480
S 3.769997 2.403770 -0.733139	S -2.962119 1.909965 -0.292855	S -3.860955 2.481892 -0.387155
C 2.068286 2.085771 -0.206632	C -3.021957 3.671360 -0.700026	C -4.429554 2.908371 1.279817
H 1.545031 1.620855 -1.043116	H -3.604748 3.770771 -1.616292	H -5.491886 3.143750 1.210072
H 2.057968 1.417183 0.653582	H -3.514018 4.214879 0.109029	H -4.302504 2.045930 1.936441
H 1.575666 3.026890 0.042087	H -2.003934 4.030601 -0.862196	H -3.879331 3.776234 1.649173
C 4.388944 2.979801 0.869234	C -2.075550 2.079878 1.273420	C -2.125130 2.195190 0.037666
H 4.328866 2.162771 1.590379	H -2.654891 2.717727 1.944287	H -2.048155 1.453835 0.833022
H 5.434912 3.256177 0.734067	H -1.987650 1.082318 1.703580	H -1.621085 1.834416 -0.859195
H 3.812969 3.846106 1.200854	H -1.081225 2.488693 1.091235	H -1.664682 3.134434 0.348786
O 4.428975 1.066752 -0.986641	O -4.372919 1.485160 0.050313	O -4.474210 1.140342 -0.716184
dmso-a7	dmso-a8	dmso-a9
C 3.326582 -1.312973 2.407053	C 3.775342 0.302051 2.363007	C 2.718397 -2.677945 1.754769
C 1.931306 -1.485510 2.456197	C 2.380677 0.124472 2.409418	C 1.315761 -2.769669 1.702718
C 1.199464 -1.544620 1.291506	C 1.727565 -0.504040 1.375176	C 0.631855 -2.291502 0.610359
C 1.111713 -1.545668 -1.168464	C 1.792099 -1.655644 -0.789066	C 0.639185 -1.213546 -1.599772
C 1.755045 -1.447262 -2.379435	C 2.503012 -2.118597 -1.870102	C 1.326237 -0.636220 -2.641800
C 3.147509 -1.252193 -2.430213	C 3.895627 -1.929633 -1.934265	C 2.724304 -0.510182 -2.579792
C 3.876919 -1.172238 -1.270410	C 4.557658 -1.290579 -0.912691	C 3.415062 -0.975165 -1.484515
C 3.967457 -1.199304 1.195806	C 4.495634 -0.150510 1.283081	C 3.415653 -2.099797 0.719709
C 3.242318 -1.301254 -0.012821	C 3.846073 -0.813281 0.214224	C 2.732392 -1.597698 -0.413551
C 1.844693 -1.473478 0.037340	C 2.451383 -0.999650 0.270891	C 1.327475 -1.703536 -0.468871
C 5.430051 -0.930302 1.155234	C 5.963704 0.057724 1.232736	C 4.892955 -1.994916 0.788987
C 5.338260 -0.907378 -1.332869	C 6.026016 -1.095538 -0.988479	C 4.882372 -0.787688 -1.404192

C -0.278656 -1.623179 1.357742	C 0.248562 -0.642774 1.415325	C -0.852910 -2.345616 0.594594
C -0.358571 -1.696269 -1.129637	C 0.327596 -1.883016 -0.720996	C -0.842538 -1.285437 -1.653381
O 5.946931 -0.769753 -2.372290	O 6.712394 -1.469418 -1.916761	O 5.529179 -0.170653 -2.228644
O 6.107702 -0.796521 2.152946	O 6.597725 0.617862 2.103022	O 5.563112 -2.418392 1.705770
O -0.886898 -1.651043 2.408096	O -0.414658 -0.170892 2.317063	O -1.493037 -2.852138 1.490560
O -1.024308 -1.845230 -2.137762	O -0.260133 -2.515590 -1.580260	O -1.467627 -0.941114 -2.636459
N 5.988340 -0.866824 -0.108970	N 6.592949 -0.438001 0.097542	N 5.492027 -1.341809 -0.287729
N -0.960769 -1.641531 0.131461	N -0.344395 -1.387615 0.393281	N -1.487156 -1.743289 -0.500793
H 3.915988 -1.233678 3.313262	H 4.304928 0.800094 3.167145	H 3.272670 -3.051108 2.608418
H 1.404882 -1.543427 3.401921	H 1.798556 0.483243 3.250346	H 0.749860 -3.203986 2.518317
H 1.164426 -1.507354 -3.286095	H 1.969109 -2.626094 -2.664628	H 0.764875 -0.271023 -3.493461
H 3.666350 -1.144069 -3.375596	H 4.470848 -2.283170 -2.782402	H 3.280372 -0.037569 -3.381329
C -2.400112 -1.648169 0.182982	C -1.744902 -1.709804 0.531697	C -2.892550 -1.451409 -0.377828
C -3.098694 -0.508212 -0.132401	C -2.733479 -0.913867 0.006356	C -3.256621 -0.139373 -0.169736
C -3.040842 -2.844273 0.569220	C -2.034360 -2.895532 1.244217	C -3.830511 -2.497916 -0.456425
C -4.529202 -0.550773 -0.065652	C -4.096479 -1.323320 0.194507	C -4.647275 0.163351 -0.046741
C -4.401189 -2.894612 0.637756	C -3.324998 -3.287925 1.434032	C -5.160810 -2.217721 -0.331996
H -2.431530 -3.706678 0.813003	H -1.207758 -3.479093 1.634397	H -3.475978 -3.510351 -0.607140
C -5.325999 0.581690 -0.372385	C -5.177679 -0.564941 -0.323934	C -5.106128 1.489379 0.160955
C -5.180202 -1.753142 0.323305	C -4.390683 -2.512830 0.914261	C -5.603302 -0.886974 -0.128008
H -4.903381 -3.809424 0.935988	H -3.550380 -4.196903 1.982771	H -5.896679 -3.013640 -0.387505
C -6.691172 0.521014 -0.295454	C -6.472918 -0.964504 -0.132657	C -6.442697 1.755495 0.281825
H -4.834519 1.499918 -0.669565	H -4.960372 0.339353 -0.878401	H -4.375972 2.287456 0.220904
C -6.595361 -1.784552 0.391582	C -5.741524 -2.900227 1.096363	C -6.980780 -0.576798 0.000485
C -7.335930 -0.674796 0.089813	C -6.761444 -2.144673 0.586550	C -7.392283 0.712169 0.200237
H -7.284556 1.397774 -0.532867	H -7.285689 -0.370689 -0.537659	H -6.778523 2.774794 0.440964
H -7.080501 -2.709007 0.690249	H -5.950412 -3.811166 1.649445	H -7.703586 -1.384816 -0.061815
H -8.418983 -0.709161 0.145988	H -7.792688 -2.449686 0.731469	H -8.449127 0.938137 0.297575
C -2.423667 0.758614 -0.545869	C -2.469979 0.345569 -0.749618	C -2.218596 0.931745 -0.096186
C -1.953527 1.680579 0.436213	C -2.557249 1.603072 -0.084602	C -1.410483 1.078578 1.072925
C -2.367258 1.089957 -1.892839	C -2.299574 0.297135 -2.124213	C -2.010966 1.759646 -1.189179
C -1.982424 1.388532 1.826502	C -2.752128 1.705380 1.318321	C -1.583371 0.266512 2.224288
C -1.459518 2.953391 0.031719	C -2.489762 2.807971 -0.837662	C -0.386259 2.064725 1.100379
C -1.838702 2.353129 -2.281552	C -2.196017 1.512596 -2.857447	C -0.988469 2.748599 -1.138715
C -1.566432 2.312632 2.749449	C -2.889791 2.926366 1.925384	C -0.770521 0.414484 3.318859
H -2.327415 0.415546 2.155266	H -2.763588 0.800143 1.912125	H -2.361692 -0.486916 2.230570
C -1.051957 3.891484 1.011104	C -2.638203 4.054285 -0.182685	C 0.427435 2.206866 2.251064
C -1.413395 3.254367 -1.353427	C -2.298368 2.720247 -2.240099	C -0.200168 2.895660 -0.036759
H -1.801744 2.597358 -3.339306	H -2.061147 1.457619 -3.933970	H -0.844526 3.389372 -2.004543
C -1.105687 3.584878 2.345538	C -2.840742 4.119513 1.171688	C 0.246732 1.391664 3.338326
H -1.596116 2.063274 3.805072	H -3.035475 2.976522 2.999677	H -0.920040 -0.224841 4.183329
H -0.697569 4.864898 0.682037	H -2.596210 4.962165 -0.779050	H 1.196205 2.973651 2.236082
H -1.039401 4.224739 -1.668483	H -2.246515 3.636739 -2.821948	H 0.593218 3.636786 -0.005723
H -0.792234 4.309895 3.089136	H -2.959648 5.078885 1.664482	H 0.873674 1.502680 4.217223
N -2.878860 0.247929 -2.863728	N -2.316232 -0.896913 -2.822246	N -2.796801 1.662604 -2.327898
H -2.782606 -0.736879 -2.647873	H -1.973795 -1.703203 -2.313717	H -3.135550 0.727534 -2.514389
H -2.563223 0.450427 -3.801015	H -1.911693 -0.846656 -3.745464	H -2.377886 2.066117 -3.153258
H 6.974530 -0.640004 -0.143543	H 7.595646 -0.300489 0.054109	H 6.498813 -1.237943 -0.234513
S 2.669774 1.879031 -0.355456	S 1.490870 2.289885 -0.337870	S 3.850199 3.223714 0.322384
C 2.109463 2.098184 1.347674	C 1.210799 3.685928 -1.455475	C 3.646136 1.596988 1.099398
H 2.244995 1.144129 1.854019	H 1.644269 3.425545 -2.421537	H 3.742090 1.739285 2.176177
H 2.725678 2.858669 1.832007	H 0.134601 3.830797 -1.559134	H 2.656718 1.192823 0.878532
H 1.056063 2.379213 1.360856	H 1.694687 4.580396 -1.057436	H 4.436894 0.933004 0.746592
C 2.450570 3.600105 -0.870212	C 0.662561 3.002417 1.106375	C 3.453102 2.695364 -1.365386
H 3.058867 4.246825 -0.234768	H -0.306385 3.412998 0.824973	H 2.452279 2.257905 -1.380004
H 2.786066 3.679169 -1.904668	H 0.508074 2.193435 1.820282	H 3.471351 3.588920 -1.990455
H 1.392031 3.857405 -0.797079	H 1.304674 3.772971 1.539259	H 4.202551 1.982333 -1.717666
O 4.157752 1.612362 -0.296310	O 0.623673 1.157132 -0.841435	O 2.713553 4.099388 0.795230
dmso-a10	dmso-a11	acn-a1
C 3.362717 -1.065099 2.493749	C 3.466152 -0.884269 2.496623	C 3.808007 1.006349 2.141851
C 1.961626 -1.188760 2.526071	C 2.070552 -1.050685 2.547420	C 2.422611 0.779861 2.222011
C 1.256815 -1.378525 1.360532	C 1.361556 -1.305678 1.396494	C 1.804976 -0.057848 1.322270
C 1.217393 -1.636587 -1.084226	C 1.311816 -1.678608 -1.035120	C 1.932792 -1.571977 -0.607082
C 1.886579 -1.717594 -2.283419	C 1.974556 -1.785347 -2.235733	C 2.671268 -2.186455 -1.590410
C 3.288448 -1.609484 -2.321144	C 3.369908 -1.618469 -2.292116	C 4.055739 -1.957479 -1.682776
C 4.001047 -1.417992 -1.160034	C 4.082065 -1.348260 -1.147444	C 4.681317 -1.121471 -0.788616
C 4.039086 -1.138964 1.298169	C 4.131525 -0.975267 1.296585	C 4.554061 0.392765 1.163110
C 3.334569 -1.333307 0.085732	C 3.424091 -1.246851 0.101412	C 3.941394 -0.479441 0.232570
C 1.928930 -1.448363 0.120749	C 2.024845 -1.415543 0.155289	C 2.554165 -0.709875 0.320668
C 5.518835 -1.009776 1.274653	C 5.605099 -0.783663 1.251295	C 6.014344 0.648584 1.078286
C 5.480765 -1.293489 -1.211350	C 5.555390 -1.161609 -1.219796	C 6.143133 -0.886059 -0.893840
C -0.221167 -1.522599 1.413390	C -0.113365 -1.442891 1.461181	C 0.336543 -0.260060 1.399697
C -0.263955 -1.749989 -1.061484	C -0.165406 -1.802497 -1.000224	C 0.477004 -1.831741 -0.513677
O 6.124200 -1.350940 -2.237408	O 6.190001 -1.240063 -2.249487	O 6.851307 -1.398806 -1.734483
O 6.194486 -0.836790 2.266214	O 6.280531 -0.552942 2.230996	O 6.614810 1.382825 1.834912
O -0.831714 -1.536693 2.459887	O -0.727082 -1.391442 2.505916	O -0.351808 0.312728 2.221363
O -0.905933 -1.933071 -2.077408	O -0.816479 -2.039908 -2.000283	O -0.088653 -2.612896 -1.256169
N 6.101410 -1.098660 0.015968	N 6.178521 -0.892313 -0.008678	N 6.673639 -0.019082 0.054650
N -0.884539 -1.644487 0.183920	N -0.781274 -1.623136 0.239855	N -0.226382 -1.157839 0.487609
H 3.933387 -0.912802 3.402799	H 4.039949 -0.674403 3.391984	H 7.670413 0.149511 -0.011749
H 1.413885 -1.137933 3.459770	H 1.528931 -0.970630 3.482665	C -1.618256 -1.500511 0.669179
H 1.310544 -1.864400 -3.189415	H 1.397538 -1.985319 -3.130874	C -2.623083 -0.789741 0.060672
H 3.831266 -1.670376 -3.257554	H 3.907021 -1.689686 -3.230923	C -1.879108 -2.603936 1.512293
C -2.326417 -1.699203 0.215094	C -2.219896 -1.707656 0.288153	C -3.976942 -1.201855 0.299076
C -3.053799 -0.580065 -0.110383	C -2.985372 -0.631421 -0.086900	C -3.161447 -2.997369 1.750392
C -2.933274 -2.917377 0.584162	C -2.785697 -2.920760 0.734769	C -5.074011 -0.529635 -0.298290

C	-4.481422	-0.670386	-0.077269
C	-4.293312	-3.011225	0.623259
H	-2.302334	-3.761747	0.837051
C	-5.303661	0.438070	-0.402929
C	-5.101756	-1.894810	0.293202
H	-4.772126	-3.942617	0.908348
C	-6.667555	0.333758	-0.360704
H	-4.830099	1.370133	-0.686546
C	-6.516631	-1.970606	0.326335
C	-7.282944	-0.883279	0.007329
H	-7.282891	1.191213	-0.612064
H	-6.980646	-2.910105	0.611354
H	-8.365489	-0.951663	0.036355
C	-2.390015	0.699531	-0.492735
C	-1.933399	1.601382	0.516036
C	-2.266764	1.033139	-1.832733
C	-2.055200	1.316044	1.902488
C	-1.337230	2.835616	0.137613
C	-1.653462	2.266511	-2.192230
C	-1.609843	2.207347	2.843678
H	-2.493311	0.375938	2.213849
C	-0.884047	3.735339	1.133199
C	-1.200746	3.133834	-1.242964
H	-1.548941	2.506388	-3.246886
C	-1.019437	3.431372	2.461732
H	-1.712415	1.965279	3.896861
H	-0.389567	4.644817	0.811347
H	-0.689418	4.047635	-1.523402
H	-0.668886	4.123476	3.220473
N	-2.768710	0.212477	-2.831780
H	-2.732762	-0.775139	-2.613908
H	-2.389428	0.397086	-3.749031
H	7.110583	-1.010015	-0.009480
S	2.605540	3.194984	-0.484825
C	2.100833	2.179670	0.933023
H	2.130011	2.817967	1.815988
H	1.085676	1.804707	0.799926
H	2.813649	1.360654	1.044000
C	2.189071	1.966199	-1.752261
H	1.125450	1.727458	-1.694908
H	2.411491	2.411583	-2.722671
H	2.801627	1.074659	-1.608476
O	1.636574	4.341338	-0.629949

C	-4.411171	-0.760386	-0.017014
C	-4.140280	-3.052993	0.804948
H	-2.124763	-3.730404	1.022091
C	-5.275812	0.303376	-0.380523
C	-4.987491	-1.979577	0.432510
H	-4.585394	-3.981087	1.149115
C	-6.634815	0.161481	-0.300675
H	-4.842055	1.234711	-0.723154
C	-6.398045	-2.095965	0.502207
C	-7.205136	-1.051143	0.144435
H	-7.280764	0.986564	-0.581840
H	-6.825652	-3.032533	0.847309
H	-8.284103	-1.149961	0.202196
C	-2.395706	0.654900	-0.563869
C	-2.011990	1.662704	0.371895
C	-2.355994	0.919549	-1.926467
C	-2.030362	1.441331	1.775711
C	-1.611236	2.945008	-0.096938
C	-1.946465	2.206630	-2.377407
C	-1.668844	2.434795	2.648634
H	-2.321222	0.469406	2.154662
C	-1.247303	3.950287	0.831510
C	-1.596334	3.183438	-1.494875
H	-1.928329	2.401433	-3.445886
C	-1.274980	3.706434	2.178566
H	-1.686743	2.238852	3.715863
H	-0.938193	4.918690	0.450263
H	-1.288245	4.160154	-1.855390
H	-0.993506	4.482008	2.882780
N	-2.776759	-0.017311	-2.852960
H	-2.604319	-0.978795	-2.586498
H	-2.484559	0.166243	-3.801338
H	7.181025	-0.750921	-0.050467
S	1.907184	3.141410	-0.480327
C	1.505058	1.712307	-1.523798
H	1.465256	2.057487	-2.557164
H	2.311183	0.985936	-1.414129
H	0.539972	1.289095	-1.242193
C	1.791591	2.252193	1.096267
H	2.531769	1.450538	1.093262
H	2.029711	2.963188	1.887689
H	0.782477	1.865617	1.245341
O	3.365479	3.440758	-0.708350

C	-4.244223	-2.307448	1.151151
C	-6.360308	-0.930850	-0.056309
C	-5.586318	-2.698350	1.382819
C	-6.622577	-2.026332	0.794795
C	-2.377143	0.378698	-0.833655
C	-2.518928	1.704658	-0.325765
C	-2.160371	0.173430	-2.188227
C	-2.751792	1.967160	1.051206
C	-2.449827	2.813498	-1.213971
C	-2.066555	1.297546	-3.057850
C	-2.912131	3.249578	1.507615
C	-2.627086	4.125675	-0.712934
C	-2.213629	2.565660	-2.590196
C	-2.855444	4.346621	0.620266
N	-2.111347	-1.091884	-2.739590
H	-1.802338	-1.835492	-2.126277
H	-1.665606	-1.142732	-3.643272
H	4.311115	1.665579	2.840014
H	1.818869	1.260442	2.983055
H	2.164691	-2.844132	-2.286701
H	4.652239	-2.430913	-2.454143
H	-1.040144	-3.123126	1.962399
H	-3.367350	-3.842584	2.399441
H	-4.876686	0.309182	-0.954032
H	-7.186154	-0.404137	-0.522890
H	-5.775490	-3.544100	2.037155
H	-7.646983	-2.333282	0.978568
H	-2.773605	1.136787	1.745726
H	-1.888048	1.120705	-4.114285
H	-3.085272	3.423517	2.564831
H	-2.581119	4.957223	-1.411057
H	-2.149495	3.408607	-3.271972
H	-2.993908	5.355482	0.994832
C	0.593756	3.135596	0.385216
C	0.856109	2.210283	-0.709350
N	1.070365	1.449151	-1.551601
H	0.083796	2.603719	1.191523
H	-0.059417	3.940764	0.047475
H	1.533076	3.550582	0.755735

acn-a2

C	-3.658634	-0.296104	-2.490568
C	-2.276504	-0.534913	-2.576475
C	-1.566143	-0.895195	-1.453708
C	-1.499405	-1.415343	0.949679
C	-2.143485	-1.536522	2.156755
C	-3.525031	-1.289588	2.250853
C	-4.242281	-0.929179	1.136432
C	-4.309122	-0.418452	-1.283274
C	-3.601031	-0.796733	-0.118684
C	-2.215100	-1.041460	-0.209154
C	-5.767353	-0.145998	-1.200125
C	-5.699752	-0.671515	1.245514
C	-0.099120	-1.096866	-1.552613
C	-0.038888	-1.650451	0.871573
O	-6.329514	-0.772347	2.276533
O	-6.443263	0.188976	-2.150536
O	0.501684	-0.986016	-2.601384
O	0.607550	-2.032704	1.827649
N	-6.326409	-0.298287	0.060563
N	0.571951	-1.410230	-0.364084
H	-7.320942	-0.116938	0.127501
C	2.004832	-1.550232	-0.438012
C	2.814167	-0.549114	0.040542
C	2.520123	-2.729795	-1.015518
C	4.232814	-0.725419	-0.053990
C	3.868419	-2.905265	-1.112328
C	5.139086	0.259363	0.415076
C	4.758969	-1.911042	-0.635538
C	6.491226	0.075167	0.309795
C	6.163787	-2.072676	-0.728738
C	7.012052	-1.103621	-0.267640
C	2.268363	0.697482	0.654125
C	1.894831	1.803133	-0.167111
C	2.226008	0.814340	2.035951
C	1.905249	1.733637	-1.586384
C	1.506706	3.031739	0.437563
C	1.813011	2.043445	2.623135
C	1.559753	2.821062	-2.348519
C	1.169825	4.138557	-0.377143
C	1.475150	3.112477	1.853070
C	1.195221	4.043247	-1.743914
N	2.638757	-0.217420	2.860889

acn-a3

C	-3.513466	-0.215151	-2.482039
C	-2.124558	-0.418905	-2.574723
C	-1.414655	-0.859085	-1.480942
C	-1.354642	-1.539766	0.883680
C	-2.010927	-1.774509	2.069961
C	-3.401843	-1.585565	2.160991
C	-4.116069	-1.164487	1.065674
C	-4.172285	-0.463131	-1.301601
C	-3.465656	-0.925179	-0.167092
C	-2.071638	-1.114988	-0.257019
C	-5.637301	-0.222994	-1.208161
C	-5.581647	-0.939805	1.180143
C	0.052210	-1.042501	-1.586221
C	0.114798	-1.715173	0.809819
O	-6.206596	-1.123650	2.202450
O	-6.304852	0.187692	-2.133651
O	0.658017	-0.880697	-2.624832
O	0.768430	-2.106297	1.758768
N	-6.210993	-0.522785	0.016863
N	0.724440	-1.402915	-0.408220
H	-7.201027	-0.325593	0.099391
C	2.161513	-1.492824	-0.476201
C	2.931354	-0.480998	0.043776
C	2.723586	-2.631768	-1.089514
C	4.355525	-0.601866	-0.041420
C	4.078512	-2.754757	-1.178773
C	5.221447	0.397172	0.471629
C	4.928691	-1.747121	-0.658509
C	6.580138	0.264344	0.374703
C	6.339284	-1.855640	-0.742168
C	7.148007	-0.874314	-0.238123
C	2.329443	0.720466	0.693650
C	1.901658	1.830153	-0.094823
C	2.267414	0.785228	2.078483
C	1.931493	1.808317	-1.515294
C	1.434429	3.012051	0.544856
C	1.778602	1.968385	2.701208
C	1.511330	2.889192	-2.245288
C	1.024182	4.118273	-0.238183
C	1.387596	3.043879	1.961499
C	1.054513	4.062532	-1.606188
N	2.728541	-0.252014	2.870563

acn-a4

C	-3.663015	-0.232666	-2.425902
C	-2.277948	-0.454505	-2.530032
C	-1.557442	-0.858152	-1.432140
C	-1.467780	-1.452380	0.954151
C	-2.104200	-1.633376	2.161554
C	-3.486710	-1.406869	2.272304
C	-4.213429	-1.006969	1.173747
C	-4.305903	-0.418365	-1.226797
C	-3.584281	-0.827791	-0.080096
C	-2.195863	-1.051906	-0.187148
C	-5.766697	-0.177342	-1.125744
C	-5.672419	-0.755962	1.306517
C	-0.096396	-1.068645	-1.553264
C	0.000786	-1.647142	0.865561
O	-6.281370	-0.878015	2.349076
O	-6.464092	0.154805	-2.059657
O	0.494163	-0.969427	-2.607308
O	0.660527	-2.008288	1.822624
N	-6.312922	-0.364017	0.140921
N	0.594051	-1.384538	-0.369012
H	-7.308548	-0.193668	0.219309
C	2.025869	-1.520359	-0.465803
C	2.842622	-0.529388	0.020653
C	2.532170	-2.686588	-1.077686
C	4.259965	-0.700228	-0.105557
C	3.878422	-2.858413	-1.201914
C	5.174753	0.277070	0.362777
C	4.776299	-1.872640	-0.721518
C	6.524661	0.097706	0.225704
C	6.179115	-2.030149	-0.846431
C	7.035489	-1.068902	-0.384261
C	2.310924	0.702758	0.675334
C	1.878703	1.813158	-0.111717
C	2.344163	0.800596	2.059774
C	1.800569	1.754742	-1.528382
C	1.509949	3.029352	0.529038
C	1.981063	2.027721	2.682904
C	1.349683	2.827606	-2.251206
C	1.056675	4.122067	-0.248556
C	1.594651	3.104502	1.942089
C	0.968842	4.026050	-1.610882
N	2.793718	-0.249517	2.843397

H 2.462597 -1.141978 2.487664	H 2.600412 -1.171049 2.465227	H 2.571766 -1.165090 2.471927
H 2.313800 -0.140825 3.813770	H 2.403665 -0.224421 3.825971	H 2.546964 -0.177578 3.819550
H -4.234819 -0.007238 -3.362230	H -4.086808 0.144512 -3.328657	H -4.242998 0.096337 -3.280048
H -1.744095 -0.430028 -3.514596	H -1.588417 -0.227931 -3.497128	H -1.754801 -0.300893 -3.466237
H -1.564185 -1.811854 3.029664	H -1.432291 -2.099025 2.926896	H -1.514945 -1.941162 3.017376
H -4.045077 -1.371800 3.198087	H -3.932653 -1.754575 3.090735	H -4.004440 -1.536644 3.215856
H 1.826478 -3.478673 -1.380017	H 2.060641 -3.391542 -1.486942	H 1.832685 -3.426994 -1.448003
H 4.275254 -3.807968 -1.556919	H 4.521561 -3.625996 -1.650638	H 4.277990 -3.750647 -1.673349
H 4.740855 1.163108 0.859671	H 4.787568 1.270498 0.943117	H 4.786042 1.172867 0.831036
H 7.170032 0.839554 0.673241	H 7.227986 1.038678 0.771976	H 7.209440 0.857160 0.588266
H 6.553661 -2.982515 -1.175297	H 6.765573 -2.734768 -1.216035	H 6.560647 -2.930216 -1.319179
H 8.086194 -1.236925 -0.344209	H 8.226862 -0.966900 -0.307615	H 8.107988 -1.198501 -0.485860
H 2.177334 0.802523 -2.068569	H 2.272534 0.914909 -2.023257	H 2.078479 0.840487 -2.037846
H 1.775510 2.115068 3.705950	H 1.737572 2.005768 3.786033	H 2.032271 2.096034 3.765899
H 1.573505 2.739655 -3.430679	H 1.535217 2.842807 -3.329171	H 1.280825 2.751912 -3.331375
H 0.888924 5.070103 0.106744	H 0.682968 5.015765 0.271157	H 0.772190 5.038955 0.260186
H 1.164419 4.042591 2.319035	H 1.029525 3.942817 2.455242	H 1.328715 4.036092 2.433971
H 0.936563 4.897891 -2.360241	H 0.735719 4.913797 -2.198386	H 0.605690 4.862286 -2.198056
C -1.772630 2.455374 -0.571267	C -1.894100 2.436687 0.305349	C -1.560066 1.938981 1.407723
C -1.836918 2.082872 0.836243	C -3.304421 2.252139 0.620680	C -2.086746 2.274421 0.090871
N -1.895837 1.774108 1.947608	N -4.420477 2.085090 0.866060	N -2.516961 2.524482 -0.951082
H -2.631886 2.042647 -1.103421	H -1.626946 1.862926 -0.584407	H -0.616128 1.401012 1.309719
H -0.853671 2.073546 -1.019499	H -1.268116 2.104375 1.135550	H -2.276544 1.314765 1.945548
H -1.770509 3.541124 -0.672189	H -1.677205 3.487181 0.109279	H -1.371162 2.848047 1.979882
acn-a5	becn-a1	becn-a2
C 3.592090 -1.148978 2.346135	C -3.156028 -0.043661 -2.798223	C 3.263596 -0.552320 2.476867
C 2.200446 -1.353458 2.365420	C -1.765717 -0.245471 -2.837390	C 1.864602 -0.622402 2.608599
C 1.487458 -1.372629 1.189370	C -1.127435 -0.864322 -1.787651	C 1.094127 -1.072005 1.562235
C 1.418959 -1.180910 -1.261047	C -1.217441 -1.944691 0.420614	C 0.915481 -1.929125 -0.736468
C 2.068772 -0.985953 -2.457942	C -1.943520 -2.383750 1.503237	C 1.514970 -2.310354 -1.915512
C 3.460934 -0.787817 -2.484097	C -3.336214 -2.195359 1.540940	C 2.910368 -2.234754 -2.055080
C 4.183703 -0.787665 -1.313656	C -3.979175 -1.567222 0.500473	C 3.686441 -1.782547 -1.013393
C 4.250454 -0.970876 1.152221	C -3.886841 -0.466845 -1.712937	C 3.867238 -0.927356 1.300688
C 3.537528 -0.984921 -0.070128	C -3.256310 -1.128266 -0.633700	C 3.095776 -1.405245 0.214096
C 2.141412 -1.185590 -0.047647	C -1.861039 -1.325555 -0.673230	C 1.694842 -1.479163 0.350291
C 5.720465 -0.756177 1.142388	C -5.343108 -0.189806 -1.651634	C 5.336048 -0.784925 1.146707
C 5.653911 -0.573070 -1.352061	C -5.437187 -1.304935 0.585200	C 5.154163 -1.653754 -1.191176
C 0.019661 -1.589496 1.227164	C 0.348673 -1.014182 -1.816644	C -0.381641 -1.094367 1.699276
C -0.054592 -1.368715 -1.248505	C 0.260158 -2.063636 0.436727	C -0.562546 -1.974688 -0.620889
O 6.280706 -0.403037 -2.375810	O -6.125680 -1.615802 1.534587	O 5.734806 -1.923684 -2.222307
O 6.403359 -0.733723 2.143755	O -5.954367 0.404895 -2.514995	O 6.067595 -0.345071 2.008425
O -0.574076 -1.820125 2.258495	O 1.017375 -0.694347 -2.776321	O -0.946707 -0.778873 2.724650
O -0.704937 -1.399448 -2.275133	O 0.859507 -2.580938 1.358982	O -1.265270 -2.405549 -1.515909
N 6.285014 -0.577117 -0.114890	N -5.986825 -0.650712 -0.510437	N 5.845886 -1.184386 -0.083449
N -0.659750 -1.506691 0.002165	N 0.944981 -1.526681 -0.656827	N -1.113627 -1.489726 0.566356
H 7.287371 -0.429028 -0.130694	H -6.980638 -0.461378 -0.457731	H 6.847446 -1.084497 -0.197799
C -2.097084 -1.623610 0.039457	C 2.385214 -1.575998 -0.619788	C -2.550655 -1.457778 0.675420
C -2.874628 -0.504558 -0.137491	C 3.091884 -0.547360 -0.046400	C -3.265953 -0.481890 0.025058
C -2.648125 -2.901982 0.265701	C 3.014945 -2.705543 -1.183682	C -3.168898 -2.445396 1.471232
C -4.297684 -0.658152 -0.092332	C 4.521649 -0.641951 -0.026053	C -4.691686 -0.480125 0.169224
C -4.001811 -3.057137 0.312882	C 4.374467 -2.802665 -1.171982	C -4.524214 -2.452173 1.614162
C -5.172522 0.444781 -0.263911	C 5.326965 0.374566 0.548281	C -5.505137 0.495088 -0.462094
C -4.860460 -1.943696 0.135435	C 5.162811 -1.776540 -0.593952	C -5.320158 -1.472410 0.969941
C -6.529740 0.277543 -0.210807	C 6.691611 0.268126 0.555346	C -6.864688 0.483429 -0.305014
C -6.269919 -2.084178 0.183202	C 6.577367 -1.857300 -0.570654	C -6.730199 -1.459218 1.110903
C -7.087209 -1.000491 0.014163	C 7.326447 -0.859399 -0.010002	C -7.487124 -0.504703 0.488907
C -2.278140 0.841264 -0.382588	C 2.437438 0.656708 0.547601	C -2.613363 0.555181 -0.828263
C -1.767738 1.617984 0.700936	C 2.222266 1.818464 -0.256870	C -2.032269 1.720134 -0.243102
C -2.273871 1.357436 -1.670622	C 2.148454 0.684397 1.906326	C -2.655124 0.418355 -2.208646
C -1.731344 1.130103 2.034280	C 2.474060 1.828282 -1.654613	C -1.920135 1.889646 1.163606
C -1.267742 2.926874 0.454987	C 1.727221 3.010322 0.341818	C -1.532278 2.756566 -1.081310
C -1.778494 2.672622 -1.896500	C 1.674654 1.896502 2.491121	C -2.133230 1.455566 -3.031707
C -1.194500 1.884567 3.044703	C 2.222325 2.945722 -2.406677	C -1.336804 3.011926 1.694238
C -0.726028 3.685002 1.521436	C 1.478754 4.147752 -0.462328	C -0.959152 3.913890 -0.501279
C -1.302706 3.432262 -0.870025	C 1.480785 3.014457 1.739283	C -1.603614 2.586918 -2.486828
C -0.679485 3.174535 2.791614	C 1.716558 4.121470 -1.811154	C -0.855290 4.042859 0.858108
N -2.792679 0.639260 -2.735824	N 2.361929 -0.401348 2.731897	N -3.250062 -0.681290 -2.804924
H -2.665390 -0.361819 -2.657725	H 2.370196 -1.299433 2.265133	H -3.141121 -1.539869 -2.279334
H -2.506008 0.976032 -3.643338	H 1.755179 -0.423219 3.541513	H -3.002604 -0.812635 -3.774854
H 4.168755 -1.128095 3.263804	H -3.673067 0.461627 -3.605928	H 3.879713 -0.170717 3.282457
H 1.666011 -1.496816 3.297216	H -1.170290 0.095151 -3.676527	H 1.373169 -0.297166 3.517632
H 1.484906 -0.984823 -3.370856	H -1.415365 -2.834781 2.334533	H 0.886677 -2.654984 -2.728356
H 3.988202 -0.630735 -3.418150	H -3.920630 -2.513400 2.396629	H 3.398857 -2.513946 -2.981567
H -1.978983 -3.742846 0.406321	H 2.397912 -3.481593 -1.622125	H -2.546550 -3.182162 1.965516
H -4.437194 -4.035627 0.488870	H 4.868517 -3.666816 -1.604628	H -5.008952 -3.206054 2.226259
H -4.746257 1.425145 -0.437629	H 4.842180 1.239471 0.984406	H -5.030602 1.253787 -1.072047
H -7.184477 1.132478 -0.342929	H 7.291886 1.055140 0.999616	H -7.470842 1.238774 -0.794077
H -6.688275 -3.070820 0.358346	H 7.055273 -2.729035 -1.007556	H -7.197895 -2.222359 1.725749
H -8.165124 -1.118536 0.053202	H 8.409075 -0.930985 0.002769	H -8.566029 -0.502498 0.604332
H -2.115102 0.140403 2.248218	H 2.837284 0.926281 -2.130710	H -2.281841 1.109656 1.822372
H -1.790763 3.066647 -2.908753	H 1.467198 1.904681 3.555815	H -2.179211 1.335952 -4.110497
H -1.167516 1.483179 4.052760	H 2.411806 2.923836 -3.475042	H -1.238367 3.105958 2.770421
H -0.329087 4.671781 1.304245	H 1.093950 5.044975 0.014391	H -0.583792 4.691620 -1.160164
H -0.912869 4.427009 -1.060402	H 1.113755 3.924953 2.203780	H -1.218341 3.372887 -3.129834
H -0.255625 3.758365 3.601748	H 1.522290 4.997943 -2.420448	H -0.396447 4.922705 1.295463
C 1.849893 2.152636 -0.362355	C -1.308808 0.461386 2.934129	C 2.577355 2.578307 1.822713

C 1.945209 3.601192 -0.484505	N -0.703582 -0.104473 3.741776	N 2.423815 2.868017 2.932133
N 2.001008 4.750425 -0.582050	C -2.038200 1.156894 1.912096	C 2.754039 2.184460 0.453984
H 1.026362 1.790834 -0.980756	C -3.401376 1.419221 2.074375	C 1.639793 1.794452 -0.290046
H 1.643893 1.882599 0.675364	C -1.371523 1.558569 0.751450	C 4.031305 2.165778 -0.115107
H 2.779178 1.681743 -0.686759	C -4.090316 2.088463 1.072535	C 1.801247 1.397525 -1.610607
	H -3.911153 1.093468 2.974069	H 0.661601 1.800747 0.171368
	C -2.066466 2.240608 -0.235973	C 4.182955 1.756905 -1.432014
	H -0.321450 1.329813 0.628706	H 4.890227 2.456601 0.479520
	C -3.423747 2.502768 -0.077961	C 3.069884 1.376526 -2.179086
	H -5.149467 2.289535 1.190950	H 0.930266 1.105933 -2.187578
	H -1.541242 2.559636 -1.129353	H 5.171085 1.732473 -1.878737
	H -3.968934 3.024848 -0.857475	H 3.197631 1.056011 -3.207732
becn-a3	becn-a4	becn-a5
C -3.226018 -0.201888 -2.443110	C 3.498549 0.123198 2.391751	C 3.137671 -1.029293 2.512879
C -1.832431 -0.285187 -2.606802	C 2.092340 0.072383 2.406539	C 1.735312 -1.106149 2.566543
C -1.061456 -0.909301 -1.655180	C 1.411197 -0.566611 1.397761	C 1.010767 -1.339415 1.420580
C -0.878776 -2.132663 0.471793	C 1.433270 -1.820702 -0.717630	C 0.933972 -1.755938 -1.002478
C -1.474405 -2.694259 1.576073	C 2.133456 -2.372159 -1.767160	C 1.589065 -1.932257 -2.199699
C -2.869069 -2.627159 1.737114	C 3.537415 -2.302227 -1.797517	C 2.990122 -1.838791 -2.260574
C -3.646746 -1.999472 0.792967	C 4.221843 -1.690622 -0.773922	C 3.716719 -1.577540 -1.123154
C -3.827316 -0.753234 -1.337389	C 4.203255 -0.457986 1.365718	C 3.792819 -1.180540 1.313894
C -3.057449 -1.425028 -0.358095	C 3.524091 -1.114935 0.312612	C 3.069308 -1.432270 0.125138
C -1.658928 -1.496102 -0.518022	C 2.115668 -1.174498 0.335312	C 1.663539 -1.517474 0.182369
C -5.291124 -0.609822 -1.147257	C 5.687671 -0.375911 1.345891	C 5.272814 -1.056373 1.262806
C -5.112960 -1.896480 0.992535	C 5.708174 -1.633889 -0.807823	C 5.188687 -1.386942 -1.211774
C 0.413197 -0.921733 -1.806922	C -0.070451 -0.628927 1.436759	C -0.468212 -1.382284 1.488856
C 0.597500 -2.157530 0.343735	C -0.043139 -1.934835 -0.680377	C -0.547929 -1.780025 -0.965958
O -5.702577 -2.361705 1.945136	O 6.372811 -2.110199 -1.702266	O 5.802237 -1.416151 -2.258653
O -6.018775 -0.014602 -1.915344	O 6.334620 0.193806 2.199678	O 5.962842 -0.874618 2.241364
O 0.972611 -0.451851 -2.776190	O -0.723400 -0.136058 2.331615	O -1.076767 -1.274517 2.532875
O 1.308673 -2.722992 1.152029	O -0.663861 -2.546225 -1.532345	O -1.215932 -1.988108 -1.962078
N -5.798876 -1.202323 0.001267	N 6.303552 -1.016494 0.282663	N 5.831535 -1.152815 -0.006438
N 1.144607 -1.487838 -0.754979	N -0.699330 -1.330621 0.392672	N -1.149631 -1.543504 0.271762
H -6.799503 -1.119165 0.135286	H 7.311042 -0.922913 0.236364	H 6.829137 -0.981583 -0.059371
C 2.581025 -1.404993 -0.836650	C -2.107375 -1.614620 0.550747	C -2.590158 -1.565415 0.332307
C 3.249684 -0.463785 -0.092474	C -3.078695 -0.820550 -0.004476	C -3.318829 -0.469648 -0.058661
C 3.246474 -2.303472 -1.696600	C -2.421096 -2.762802 1.313427	C -3.198037 -2.744941 0.813858
C 4.677014 -0.408773 -0.199803	C -4.450937 -1.186196 0.203835	C -4.747991 -0.541289 0.033458
C 4.604255 -2.255619 -1.808796	C -3.720043 -3.115231 1.522124	C -4.555337 -2.823409 0.903912
C 5.443964 0.530416 0.535679	C -5.515345 -0.422991 -0.340669	C -5.576187 0.547146 -0.341810
C 5.354403 -1.310282 -1.065434	C -4.769619 -2.337318 0.973545	C -5.365296 -1.726185 0.518381
C 6.806688 0.571352 0.414668	C -6.818949 -0.782334 -0.126741	C -6.938422 0.460815 -0.239940
C 6.766276 -1.243224 -1.168030	C -6.128201 -2.683680 1.177408	C -6.778095 -1.785713 0.609893
C 7.477893 -0.324724 -0.446058	C -7.132084 -1.924557 0.641467	C -7.549257 -0.718265 0.239870
C 2.544333 0.486377 0.817941	C -2.784819 0.401505 -0.807524	C -2.691633 0.781017 -0.580556
C 2.026216 1.718143 0.315538	C -2.831477 1.684271 -0.183299	C -2.242027 1.796009 0.316577
C 2.486098 0.212270 2.176538	C -2.644698 0.300706 -2.185185	C -2.675951 1.007054 -1.950581
C 2.037490 2.040059 -1.068190	C -2.983595 1.834710 1.221140	C -2.211470 1.604303 1.723943
C 1.482763 2.675699 1.218225	C -2.740254 2.860984 -0.977235	C -1.809977 3.051217 -0.196635
C 1.914893 1.170511 3.061006	C -2.518971 1.490295 -2.960183	C -2.237786 2.267877 -2.446493
C 1.547218 3.240079 -1.517496	C -3.053926 3.079624 1.790209	C -1.761802 2.594677 2.558702
C 0.999176 3.910185 0.724167	C -2.812359 4.132023 -0.358604	C -1.357001 4.054564 0.692641
C 1.441153 2.358990 2.600144	C -2.575250 2.720945 -2.378561	C -1.833877 3.255845 -1.599185
C 1.028666 4.194319 -0.616044	C -2.972401 4.245666 0.997044	C -1.328593 3.836393 2.044194
N 3.029006 -0.945696 2.705613	N -2.703350 -0.916118 -2.835076	N -3.149153 0.058357 -2.839658
H 2.985834 -1.746656 2.087642	H -2.377318 -1.716879 -2.307369	H -2.987630 -0.899265 -2.552221
H 2.686856 -1.178167 3.626720	H -2.353252 -0.914990 -3.781059	H -2.878656 0.209938 -3.800115
H -3.846617 0.312697 -3.167101	H 4.049969 0.625404 3.178322	H 3.725785 -0.838413 3.403256
H -1.338996 0.158154 -3.463696	H 1.522616 0.535529 3.203459	H 1.200278 -0.974924 3.499867
H -0.846467 -3.162823 2.323893	H 1.580151 -2.864009 -2.558689	H 1.000544 -2.119208 -3.090329
H -3.352356 -3.047584 2.611274	H 4.104607 -2.728269 -2.616995	H 3.519968 -1.943319 -3.200452
H 2.658352 -3.017789 -2.261106	H -1.607911 -3.348903 1.727268	H -2.565134 -3.572792 1.112276
H 5.126217 -2.940623 -2.469429	H -3.964961 -3.994400 2.109552	H -5.031063 -3.725672 1.274813
H 4.930446 1.216687 1.197935	H -5.280624 0.453126 -0.932241	H -5.112049 1.453354 -0.710831
H 7.377691 1.296771 0.984671	H -7.619268 -0.185479 -0.551421	H -7.555545 1.304582 -0.530371
H 7.272427 -1.936737 -1.832955	H -6.356396 -3.565324 1.768824	H -7.236510 -2.697048 0.981990
H 8.558722 -0.281589 -0.532080	H -8.169606 -2.197541 0.803689	H -8.630310 -0.773330 0.314688
H 2.425904 1.317773 -1.775736	H -3.012092 0.949657 1.844144	H -2.530336 0.654896 2.135903
H 1.862676 0.934056 4.119265	H -2.401591 1.398556 -4.036182	H -2.243964 2.434936 -3.519835
H 1.565755 3.458528 -2.580606	H -3.166379 3.169808 2.865794	H -1.740727 2.420239 3.629653
H 0.593023 4.627348 1.431750	H -2.737725 5.017840 -0.982829	H -1.024884 5.002305 0.278851
H 1.010522 3.077866 3.290514	H -2.491708 3.615390 -2.988724	H -1.506214 4.212516 -1.994916
H 0.652573 5.142826 -0.984842	H -3.029780 5.223556 1.463196	H -0.976128 4.609785 2.718241
C -1.984073 0.953081 1.961042	C 4.321403 1.863313 -1.067377	C 3.817029 2.027526 -0.184035
N -1.486862 0.349438 2.813421	N 5.373540 1.533160 -1.418235	C 2.207051 1.554482 -1.906091
C -2.610005 1.686031 0.896711	C 3.011821 2.223720 -0.607520	C 2.788861 2.112488 0.761052
C -3.996807 1.873940 0.905964	C 2.868755 3.027711 0.526414	C 1.185502 1.626497 -0.966218
C -1.833321 2.190840 -0.147117	C 1.885842 1.723625 -1.266973	H 1.976828 1.328260 -2.941400
C -4.599926 2.566997 -0.132360	C 1.597630 3.319078 0.998317	H 3.029905 2.323876 1.796586
H -4.587728 1.471740 1.721618	H 3.751331 3.403766 1.031661	H 0.159624 1.454420 -1.268510
C -2.448198 2.895285 -1.173317	C 0.620048 2.018672 -0.783558	C 3.525760 1.753290 -1.522763
H -0.762872 2.031440 -0.154637	H 2.012467 1.092854 -2.139993	H 4.332191 1.679985 -2.244099
C -3.825589 3.080910 -1.169636	C 0.475105 2.812070 0.351396	C 5.185245 2.183700 0.220692
H -5.675850 2.699708 -0.139818	H 1.480248 3.937510 1.881594	N 6.294371 2.274569 0.536755
H -1.838965 3.293189 -1.977442	H -0.255294 1.622502 -1.283384	C 1.474447 1.913633 0.365739
H -4.302905 3.621965 -1.979751	H -0.513847 3.027601 0.734881	H 0.671056 1.977099 1.091224

becn-a6

C	-3.045434	-1.215806	-2.551352
C	-1.643118	-1.304316	-2.584676
C	-0.930110	-1.441788	-1.415955
C	-0.884137	-1.676687	1.031698
C	-1.551688	-1.761450	2.231906
C	-2.955059	-1.691811	2.267930
C	-3.669254	-1.533134	1.104240
C	-3.714746	-1.272803	-1.351851
C	-3.005887	-1.451796	-0.141838
C	-1.598954	-1.530265	-0.176634
C	-5.188388	-1.082562	-1.315716
C	-5.150519	-1.425369	1.158507
C	0.551615	-1.460018	-1.459543
C	0.596386	-1.732902	1.012954
O	-5.790959	-1.523007	2.181771
O	-5.853616	-0.835960	-2.300589
O	1.174968	-1.384014	-2.497749
O	1.247541	-1.917375	2.024196
N	-5.771391	-1.187973	-0.062158
N	1.216106	-1.559099	-0.226659
H	-6.771261	-1.027117	-0.020337
C	2.657637	-1.558991	-0.256682
C	3.355183	-0.445618	0.143366
C	3.300198	-2.731410	-0.708283
C	4.786609	-0.492161	0.097852
C	4.661167	-2.784752	-0.757573
C	5.582454	0.613971	0.490908
C	5.439436	-1.670201	-0.356778
C	6.948492	0.551075	0.433667
C	6.855345	-1.704754	-0.402863
C	7.594957	-0.620645	-0.017160
C	2.679585	0.797617	0.619551
C	2.229799	1.775811	-0.317127
C	2.597346	1.050209	1.981821
C	2.279766	1.560946	-1.720940
C	1.719722	3.019632	0.150071
C	2.061507	2.290313	2.432217
C	1.850579	2.525841	-2.595914
C	1.291977	3.997109	-0.779508
C	1.649427	3.242031	1.548486
C	1.354767	3.761920	-2.127182
N	3.088554	0.151239	2.911896
H	2.998020	-0.819153	2.637388
H	2.758538	0.300453	3.854112
H	-3.620901	-1.074658	-3.459037
H	-1.098111	-1.241462	-3.519383
H	-0.972530	-1.878773	3.140296
H	-3.498344	-1.751598	3.204079
H	2.692027	-3.573876	-1.016747
H	5.164211	-3.681230	-1.105790
H	5.089687	1.513611	0.838401
H	7.541262	1.407477	0.737526
H	7.341792	-2.610482	-0.752383
H	8.678668	-0.656874	-0.056853
H	2.647185	0.614764	-2.099149
H	2.004700	2.474011	3.501372
H	1.894803	2.334855	-3.663319
H	0.904441	4.938286	-0.400412
H	1.253318	4.186504	1.909727
H	1.021932	4.515814	-2.832517
C	-5.264595	1.954787	1.064558
N	-6.325797	1.925367	1.524182
C	-3.956919	1.956766	0.472868
C	-3.831647	2.073342	-0.913844
C	-2.820196	1.811378	1.275818
C	-2.569888	2.034436	-1.490267
H	-4.720187	2.173040	-1.527379
C	-1.565068	1.774538	0.687333
H	-2.932609	1.718357	2.350017
C	-1.439529	1.880686	-0.695975
H	-2.468025	2.113207	-2.567009
H	-0.678194	1.662295	1.301479
H	-0.459123	1.839653	-1.154481

meno2-a1

C	-3.740389	0.635580	-2.258906
C	-2.349731	0.447896	-2.346926
C	-1.689475	-0.298403	-1.397912
C	-1.735782	-1.663169	0.642564
C	-2.436786	-2.215850	1.688478
C	-3.823509	-2.009660	1.797630
C	-4.490105	-1.263240	0.855018
C	-4.447565	0.083540	-1.218137
C	-3.791027	-0.690678	-0.233042
C	-2.400459	-0.892342	-0.334155
C	-5.911695	0.309323	-1.118851
C	-5.953997	-1.050209	0.980389
C	-0.214392	-0.440182	-1.472340
C	-0.276578	-1.897380	0.531654
O	-6.627202	-1.504629	1.881415
O	-6.549840	0.955959	-1.922808
O	0.445548	0.122863	-2.324096
O	0.321321	-2.621115	1.306609
N	-6.528959	-0.281207	-0.023826
N	0.390389	-1.264569	-0.519374
H	-4.275784	1.223460	-2.995462
H	-1.774207	0.889928	-3.152111
H	-1.899099	-2.807122	2.420153
H	-4.390287	-2.431564	2.619648
C	1.800260	-1.534991	-0.677822
C	2.753266	-0.769450	-0.051734
C	2.137527	-2.623285	-1.512717
C	4.131472	-1.105749	-0.262199
C	3.444494	-2.946311	-1.723427
H	1.337464	-3.188066	-1.978356
C	5.177157	-0.370811	0.353062
C	4.475797	-2.197179	-1.104863
H	3.709387	-3.780302	-2.365571
C	6.488147	-0.700730	0.138046
H	4.919621	0.458490	1.000192
C	5.841962	-2.513967	-1.307959
C	6.827310	-1.783514	-0.702372
H	7.274383	-0.127155	0.617618
H	6.090908	-3.349917	-1.954826
H	7.870595	-2.033907	-0.864391
C	2.421392	0.383988	0.834427
C	2.433103	1.710946	0.312040
C	2.238124	0.173702	2.192609
C	2.638203	1.979864	-1.067391
C	2.261202	2.817370	1.188923
C	2.033856	1.291935	3.050436
C	2.678402	3.267360	-1.537586
H	2.736546	1.148599	-1.754201
C	2.317979	4.135296	0.675264
C	2.050148	2.563378	2.567903
H	1.881622	1.110589	4.110499
C	2.523572	4.363356	-0.661196
H	2.834195	3.445342	-2.596827
H	2.198309	4.966286	1.365335
H	1.906550	3.403744	3.240893
H	2.569927	5.376994	-1.045460
N	2.325254	-1.086195	2.755550
H	2.052427	-1.854068	2.154818
H	1.912109	-1.167875	3.672556
H	-7.527281	-0.128348	0.055153
C	-0.796974	2.997575	-0.583572
H	-0.224464	2.367048	-1.262638
H	-0.104363	3.637197	-0.039556
H	-1.574040	3.562954	-1.088926
O	-0.728002	1.328041	1.013542
O	-2.658273	2.191986	0.555685
N	-1.449880	2.107516	0.408603

meno2-a2

C	3.760692	0.664787	2.274598
C	2.370139	0.458749	2.326007
C	1.744627	-0.287845	1.355041
C	1.863483	-1.655708	-0.680033
C	2.599713	-2.206691	-1.702348
C	3.987981	-1.990978	-1.770715
C	4.620449	-1.235130	-0.812191
C	4.504379	0.121814	1.253074
C	3.883554	-0.660870	0.250832
C	2.491652	-0.872427	0.311283
C	5.969515	0.355646	1.199466
C	6.087133	-1.018003	-0.889073
C	0.271743	-0.470209	1.404229
C	0.404765	-1.912579	-0.605670
O	6.792117	-1.474786	-1.763904
O	6.577172	1.014444	2.017167
O	-0.414745	0.060345	2.254056
O	-0.162297	-2.634586	-1.405423
N	6.624909	-0.239053	0.128879
N	-0.294451	-1.310665	0.440420
H	4.270020	1.252845	3.029676
H	1.768156	0.886535	3.119451
H	2.088857	-2.807371	-2.445539
H	4.582680	-2.415188	-2.571599
C	-1.694145	-1.640518	0.582313
C	-2.678380	-0.861064	0.025001
C	-1.987051	-2.805538	1.325505
C	-4.042071	-1.263581	0.211465
C	-3.280281	-3.191687	1.513927
H	-1.164472	-3.378198	1.739478
C	-5.116952	-0.520084	-0.339966
C	-4.341768	-2.432092	0.962891
H	-3.510887	-4.084756	2.086029
C	-6.413922	-0.914221	-0.149283
H	-4.892972	0.367350	-0.918898
C	-5.694438	-2.813815	1.143387
C	-6.708890	-2.072947	0.601843
H	-7.223046	-0.333247	-0.579282
H	-5.909436	-3.708173	1.720554
H	-7.741513	-2.373533	0.745793
C	-2.387826	0.372392	-0.760741
C	-2.491529	1.652827	-0.140796
C	-2.125501	0.277789	-2.119180
C	-2.772645	1.804396	1.243893
C	-2.321434	2.831028	-0.919227
C	-1.931337	1.468456	-2.877099
C	-2.878913	3.048254	1.809929
H	-2.874025	0.918453	1.857715
C	-2.440134	4.101268	-0.305917
C	-2.031958	2.695832	-2.301404
H	-1.708886	1.377976	-3.936120
C	-2.715487	4.215135	1.031530
H	-3.090078	3.136498	2.870840
H	-2.306942	4.986735	-0.921264
H	-1.877044	3.591157	-2.895116
H	-2.808850	5.192798	1.492772
N	-2.112253	-0.934120	-2.781163
H	-1.864613	-1.744939	-2.228646
H	-1.639399	-0.927628	-3.672249
H	7.625131	-0.084273	0.083177
C	0.633195	3.006616	0.654652
H	1.624219	2.979493	1.108328
H	-0.046837	2.335676	1.173535
H	0.248645	4.020018	0.608543
O	1.014863	3.351357	-1.605305
O	0.753924	1.314129	-0.927532
N	0.800777	2.522490	-0.739994

meno2-a3

C	2.981655	0.034046	2.421794
C	1.600520	-0.148277	2.616134
C	0.867841	-0.877215	1.709215
C	0.748244	-2.180220	-0.375424
C	1.376409	-2.761551	-1.451921
C	2.768405	-2.640987	-1.610055
C	3.507647	-1.920629	-0.702472
C	3.611017	-0.533688	1.339072
C	2.883019	-1.313690	0.411501
C	1.493221	-1.463137	0.586578
C	5.056016	-0.292588	1.108448

meno2-a4

C	-3.435857	-0.941336	-2.434723
C	-2.048676	-1.160771	-2.500683
C	-1.316944	-1.313013	-1.344833
C	-1.214481	-1.431831	1.111562
C	-1.844979	-1.368364	2.330485
C	-3.230138	-1.131860	2.401035
C	-3.965508	-0.977374	1.252354
C	-4.069218	-0.875973	-1.215065
C	-3.342560	-1.058709	-0.015805
C	-1.951451	-1.276446	-0.083975
C	-5.524611	-0.578553	-1.154334

meno2-a5

C	-3.631314	-0.186986	-2.490368
C	-2.237196	-0.349381	-2.555464
C	-1.534984	-0.761608	-1.445660
C	-1.498813	-1.436323	0.921153
C	-2.166945	-1.672890	2.100571
C	-3.560527	-1.507279	2.170455
C	-4.266572	-1.111855	1.060108
C	-4.302695	-0.440041	-1.318413
C	-3.605122	-0.868745	-0.165626
C	-2.205917	-1.029931	-0.232168
C	-5.771879	-0.233460	-1.252208

C	4.971856	-1.772654	-0.892727	C	-5.419279	-0.687331	1.338468	C	-5.735715	-0.914584	1.152894
C	-0.598428	-1.007678	1.889605	C	0.156354	-1.459918	-1.427533	C	-0.065550	-0.931214	-1.531333
C	-0.729622	-2.232682	-0.271972	C	0.251341	-1.631178	1.053500	C	-0.030138	-1.619231	0.861235
O	5.588866	-2.275526	-1.807058	O	-6.026674	-0.601123	2.383893	O	-6.377672	-1.107038	2.163821
O	5.720272	0.479458	1.776392	O	-6.209467	-0.393474	-2.138642	O	-6.443628	0.127652	-2.195088
O	-1.177013	-0.605472	2.875847	O	0.750091	-1.482016	-2.486101	O	0.554617	-0.753518	-2.559205
O	-1.419554	-2.782934	-1.108953	O	0.917223	-1.846098	2.047944	O	0.612100	-2.019852	1.814754
N	5.622066	-1.007236	0.070095	N	-6.066617	-0.529764	0.119579	N	-6.351554	-0.491594	-0.017132
N	-1.305119	-1.579312	0.820637	N	0.845943	-1.546219	-0.210633	N	0.593941	-1.316885	-0.352381
H	3.570569	0.627227	3.112239	H	-4.026940	-0.799992	-3.332429	H	-4.198308	0.150626	-3.349935
H	1.086493	0.293665	3.461467	H	-1.527944	-1.191782	-3.450718	H	-1.690728	-0.150787	-3.470031
H	0.773772	-3.296543	-2.176438	H	-1.250535	-1.484367	3.228815	H	-1.595697	-1.983198	2.967443
H	3.279337	-3.093052	-2.452347	H	-3.737784	-1.051613	3.355105	H	-4.100406	-1.675666	3.094882
C	-2.718530	-1.308595	0.749135	C	2.284843	-1.576440	-0.273019	C	2.014340	-1.550644	-0.447777
C	-3.126429	-0.253987	-0.037155	C	2.999092	-0.461105	0.092919	C	2.912354	-0.629066	0.029446
C	-3.622165	-2.107172	1.474145	C	2.906258	-2.760394	-0.721465	C	2.423757	-2.760036	-1.050952
C	-4.523617	0.022614	-0.128534	C	4.428045	-0.518644	0.015792	C	4.310909	-0.919685	-0.095559
C	-4.960187	-1.843919	1.400569	C	4.265769	-2.823839	-0.800678	C	3.750153	-3.045371	-1.175766
H	-3.235562	-2.916346	2.082207	H	2.284609	-3.602006	-1.003557	H	1.664859	-3.444610	-1.412410
C	-5.022108	1.082973	-0.927666	C	5.240519	0.587350	0.373600	C	5.304335	-0.020347	0.368792
C	-5.445123	-0.780227	0.599331	C	5.061076	-1.709000	-0.435407	C	4.727957	-2.135064	-0.702760
H	-5.669890	-2.448971	1.955876	H	4.754713	-3.729066	-1.146394	H	4.072512	-3.971963	-1.639971
C	-6.365520	1.332777	-0.997259	C	6.604308	0.513124	0.286168	C	6.634688	-0.313667	0.235203
H	-4.316147	1.688916	-1.483471	H	4.761347	1.495328	0.718634	H	4.992880	0.907385	0.832693
C	-6.830606	-0.494041	0.505386	C	6.475443	-1.754797	-0.513073	C	6.112400	-2.410846	-0.825131
C	-7.281317	0.536492	-0.272966	C	7.231481	-0.670565	-0.161110	C	7.046343	-1.522234	-0.367589
H	-6.733257	2.146795	-1.613097	H	7.210348	1.369325	0.563267	H	7.380387	0.387147	0.595636
H	-7.528032	-1.110406	1.064760	H	6.947359	-2.669415	-0.859427	H	6.417171	-3.343026	-1.291274
H	-8.343987	0.746210	-0.337506	H	8.313730	-0.715837	-0.224792	H	8.104203	-1.742276	-0.466620
C	-2.119522	0.565132	-0.775741	C	2.334240	0.786256	0.574799	C	2.499292	0.653640	0.671236
C	-1.404743	1.601451	-0.102638	C	1.820764	1.741510	-0.353046	C	2.266999	1.813581	-0.126359
C	-1.844895	0.284401	-2.105660	C	2.300533	1.055418	1.935644	C	2.479815	0.745512	2.056635
C	-1.671410	1.951818	1.247841	C	1.810626	1.510869	-1.755294	C	2.234343	1.762334	-1.545770
C	-0.384903	2.315651	-0.789426	C	1.307762	2.981791	0.121803	C	2.050304	3.071637	0.502785
C	-0.819565	1.009782	-2.776824	C	1.754504	2.287321	2.394336	C	2.240014	2.008982	2.668910
C	-0.956924	2.937207	1.876500	C	1.335462	2.460761	-2.623418	C	1.982772	2.889471	-2.284094
H	-2.438203	1.412985	1.790373	H	2.176119	0.565960	-2.139078	H	2.380875	0.812146	-2.044055
C	0.325207	3.340822	-0.119209	C	0.847368	3.949921	-0.801465	C	1.810179	4.220219	-0.289834
C	-0.106926	1.979697	-2.139653	C	1.283143	3.216860	1.519986	C	2.050830	3.130602	1.919363
H	-0.602121	0.762762	-3.811974	H	1.727755	2.477536	3.463252	H	2.231050	2.067138	3.753480
C	0.053345	3.644869	1.189873	C	0.858627	3.701997	-2.149387	C	1.769975	4.136167	-1.655934
H	-1.176923	3.179956	2.911038	H	1.337217	2.257172	-3.689401	H	1.948120	2.821296	-3.366510
H	1.087983	3.881921	-0.670098	H	0.480897	4.898015	-0.416918	H	1.651130	5.171829	0.210226
H	0.698839	2.496790	-2.651754	H	0.876487	4.154131	1.887581	H	1.883121	4.087509	2.405312
H	0.597258	4.437932	1.693711	H	0.506107	4.451629	-2.850232	H	1.577214	5.019411	-2.255409
N	-2.561539	-0.671599	-2.807878	N	2.841537	0.176635	2.857557	N	2.762958	-0.346827	2.855207
H	-2.907763	-1.433299	-2.238424	H	2.760540	-0.797594	2.594342	H	2.462642	-1.235262	2.472403
H	-2.090186	-1.022437	-3.629047	H	2.529617	0.325612	3.806177	H	2.486590	-0.245409	3.820654
H	6.607497	-0.841404	-0.095589	H	-7.053218	-0.306512	0.171458	H	-7.352379	-0.345589	0.039158
C	3.756958	2.940951	0.427601	C	-1.999225	2.142908	-0.961230	C	-0.965370	2.269882	0.388840
H	3.964402	4.003773	0.333885	H	-2.583011	1.696989	-1.761684	H	-0.688969	2.087116	-0.646475
H	4.554484	2.392031	0.923281	H	-1.009106	1.707606	-0.864684	H	-0.423826	1.637501	1.087112
H	2.800878	2.786188	0.930517	H	-1.917265	3.219461	-1.099852	H	-0.804651	3.318840	0.631011
O	3.891794	1.228079	-1.132942	O	-2.084720	1.744751	1.321003	O	-2.804449	1.605606	1.630817
O	3.121285	3.138275	-1.793207	O	-3.960049	1.947291	0.255597	O	-3.137369	2.235972	-0.414358
N	3.587800	2.395832	-0.944685	N	-2.738016	1.922619	0.307719	N	-2.417448	2.008165	0.544817
meno2-a6				meno2-a7				meno2-a8			
C	3.464915	0.885254	2.406376	C	-3.856925	-0.372141	-2.468735	C	-3.763200	0.585872	-2.085196
C	2.090535	0.591644	2.446656	C	-2.465400	-0.568239	-2.518415	C	-2.369372	0.469995	-2.236249
C	1.544604	-0.285057	1.537214	C	-1.766507	-0.847430	-1.369051	C	-1.637650	-0.295825	-1.358729
C	1.802771	-1.783617	-0.395933	C	-1.727053	-1.232812	1.057669	C	-1.551866	-1.835267	0.555976
C	2.604037	-2.381112	-1.338366	C	-2.389473	-1.325987	2.258830	C	-2.189994	-2.506126	1.575037
C	3.985423	-2.117107	-1.362410	C	-3.779616	-1.128046	2.316387	C	-3.575508	-2.361728	1.755807
C	4.542374	-1.249066	-0.453571	C	-4.488593	-0.842511	1.172521	C	-4.305835	-1.565605	0.904033
C	4.274133	0.289763	1.465715	C	-4.528386	-0.457287	-1.271633	C	-4.401955	-0.066154	-1.059634
C	3.735850	-0.621615	0.526424	C	-3.828793	-0.747744	-0.075618	C	-3.674334	-0.878411	-0.157412
C	2.354414	-0.903967	0.560453	C	-2.433698	-0.945755	-0.129494	C	-2.280667	-1.005276	-0.320626
C	5.725773	0.605125	1.433714	C	-5.995284	-0.243490	-1.228708	C	-5.870575	0.078391	-0.895563
C	5.997906	-0.961676	-0.498917	C	-5.955071	-0.631172	1.244425	C	-5.771612	-1.424701	1.101406
C	0.086905	-0.565310	1.579405	C	-0.296061	-1.033535	-1.435203	C	-0.162692	-0.360288	-1.491368
C	0.339152	-2.017839	-0.423639	C	-0.258437	-1.442713	1.014445	C	-0.094588	-2.012562	0.361801
O	6.760789	-1.463250	-1.296771	O	-6.598097	-0.689389	2.272276	O	-6.385619	-1.973939	1.992204
O	6.262020	1.380037	2.197438	O	-6.672460	0.010610	-2.203266	O	-6.571172	0.736213	-1.634356
O	-0.633664	-0.119000	2.445648	O	0.317309	-0.988132	-2.480560	O	0.447799	0.310584	-2.301270
O	-0.187406	-2.734877	-1.252657	O	0.373588	-1.754621	2.006347	O	0.556516	-2.797043	1.026857
N	6.453016	-0.054973	0.452467	N	-6.572634	-0.351047	0.031536	N	-6.417031	-0.610833	0.181401
N	-0.423482	-1.372386	0.553878	N	0.362440	-1.309647	-0.227197	N	0.510440	-1.237350	-0.632070
H	3.912298	1.578442	3.109615	H	-4.423923	-0.147968	-3.365100	H	-4.349004	1.204864	-2.753704
H	1.437785	1.049386	3.180940	H	-1.919863	-0.493529	-3.451522	H	-1.850121	0.996326	-3.028836
H	2.146221	-3.039261	-2.067102	H	-1.816241	-1.546849	3.151153	H	-1.602978	-3.143406	2.225771
H	4.631316	-2.578578	-2.100592	H	-4.317472	-1.194080	3.255298	H	-4.094002	-2.873193	2.558563
C	-1.841406	-1.639859	0.570649	C	1.764206	-1.641206	-0.313895	C	1.926277	-1.419352	-0.848451
C	-2.730001	-0.729813	0.052695	C	2.738000	-0.748719	0.058210	C	2.852939	-0.729811	-0.106134
C	-2.257411	-2.860589	1.144619	C	2.073775	-2.931293	-0.800855	C	2.298976	-2.339311	-1.852865
C	-4.126797	-1.050442	0.099939	C	4.109011	-1.156938	-0.058918	C	4.241681	-0.968327	-0.369692
C	-3.583141	-3.170477	1.200910	C	3.371352	-3.328409	-0.916310	C	3.616572	-2.568271	-2.115142

H	-1.504663	-3.536300	1.534653	H	1.258928	-3.588659	-1.082559	H	1.518831	-2.851323	-2.405051
C	-5.110783	-0.174061	-0.425466	C	5.176688	-0.294426	0.298553	C	5.261463	-0.301271	0.356092
C	-4.551857	-2.275653	0.681923	C	4.423453	-2.452860	-0.549506	C	4.622108	-1.891036	-1.381553
H	-3.911712	-4.106088	1.642068	H	3.613688	-4.317664	-1.291484	H	3.910000	-3.272329	-2.887281
C	-6.440093	-0.495999	-0.368285	C	6.478853	-0.698100	0.174462	C	6.582783	-0.537309	0.087572
H	-4.792998	0.757419	-0.877283	H	4.944683	0.694785	0.672963	H	4.976335	0.400016	1.130634
C	-5.935141	-2.579371	0.727455	C	5.780703	-2.842782	-0.664929	C	5.998197	-2.113903	-1.635838
C	-6.859985	-1.710694	0.216216	C	6.787703	-1.986937	-0.311745	C	6.958159	-1.453054	-0.919600
H	-7.178186	0.186500	-0.776289	H	7.281465	-0.022912	0.452312	H	7.349385	-0.018040	0.653056
H	-6.246372	-3.518088	1.175894	H	6.005258	-3.836314	-1.041459	H	6.275423	-2.820920	-2.411991
H	-7.916934	-1.952759	0.255794	H	7.824136	-2.294570	-0.404659	H	8.009288	-1.630374	-1.122062
C	-2.299199	0.559277	-0.563468	C	2.445717	0.619188	0.578341	C	2.476685	0.254262	0.950374
C	-2.427870	1.780880	0.168299	C	2.267896	1.707678	-0.323222	C	2.386305	1.641138	0.626488
C	-1.881764	0.580387	-1.887067	C	2.486659	0.846854	1.946819	C	2.366132	-0.160687	2.269739
C	-2.834792	1.808000	1.528563	C	2.185012	1.521257	-1.729180	C	2.536187	2.118554	-0.703040
C	-2.140190	3.019953	-0.467953	C	2.167105	3.033482	0.183507	C	2.172169	2.596015	1.659472
C	-1.605387	1.832915	-2.509088	C	2.387170	2.178317	2.436211	C	2.115937	0.805910	3.285891
C	-2.945002	2.993373	2.205871	C	1.991840	2.584521	-2.569117	C	2.494537	3.461094	-0.977797
H	-3.028277	0.871630	2.036051	H	2.238263	0.517881	-2.132781	H	2.664890	1.404172	-1.506651
C	-2.262720	4.227851	0.259797	C	1.986362	4.114473	-0.714774	C	2.132041	3.975421	1.342709
C	-1.733854	3.004330	-1.827351	C	2.247121	3.232442	1.584524	C	2.026348	2.131662	2.991878
H	-1.282589	1.835033	-3.545550	H	2.438019	2.342589	3.508745	H	2.017305	0.465304	4.312580
C	-2.658887	4.221110	1.570300	C	1.891536	3.899852	-2.063400	C	2.296000	4.406398	0.052545
H	-3.251087	2.987639	3.247014	H	1.911898	2.413586	-3.637599	H	2.615580	3.800999	-2.001449
H	-2.039249	5.163020	-0.246279	H	1.925790	5.121396	-0.309080	H	1.974073	4.687960	2.147461
H	-1.516836	3.946099	-2.323314	H	2.189764	4.245011	1.975374	H	1.849510	2.854345	3.783010
H	-2.752282	5.151242	2.120897	H	1.748013	4.732724	-2.743882	H	2.272536	5.465891	-0.180108
N	-1.773617	-0.561865	-2.654993	N	2.698378	-0.188268	2.841478	N	2.565964	-1.477767	2.637601
H	-1.709242	-1.438130	-2.154045	H	2.280442	-1.066515	2.556420	H	2.311772	-2.166309	1.940212
H	-1.078541	-0.504004	-3.385661	H	2.457181	0.036053	3.795896	H	2.211302	-1.722009	3.550183
H	7.444857	0.150448	0.425251	H	-7.574672	-0.207499	0.070220	H	-7.417437	-0.510838	0.305680
C	1.321853	2.175833	-0.833523	C	-1.098199	3.650011	0.743868	C	-0.792212	3.120602	-0.465170
H	1.295285	3.257946	-0.911303	H	-2.177907	3.777925	0.826537	H	-0.437447	2.442864	-1.242656
H	2.218984	1.828217	-0.322332	H	-0.702148	4.213094	-0.096548	H	-0.242574	2.954583	0.455976
H	0.430685	1.783310	-0.348434	H	-0.601186	3.891015	1.678825	H	-0.730981	4.149334	-0.808041
O	1.199010	0.404005	-2.312510	O	-0.941496	1.850483	-0.697827	O	-2.481660	1.995510	0.653162
O	1.541842	2.375880	-3.136962	O	-0.645416	1.479388	1.416328	O	-3.043214	3.322253	-0.963297
N	1.356171	1.613551	-2.207411	N	-0.860690	2.211609	0.464202	N	-2.220729	2.801268	-0.227255
meno2-a9				pin-a1				pin-a2			
C	-3.499300	-0.492245	-2.455056	C	-2.582445	-1.852332	-2.485097	C	-2.475551	-2.251452	-2.354802
C	-2.111816	-0.706259	-2.540597	C	-1.182124	-1.733033	-2.528329	C	-1.070312	-2.196383	-2.330082
C	-1.400608	-1.082465	-1.427260	C	-0.446281	-1.803388	-1.368573	C	-0.404832	-2.086651	-1.132082
C	-1.330959	-1.625743	0.970630	C	-0.344441	-2.044985	1.076873	C	-0.451609	-1.903788	1.320961
C	-1.980618	-1.804718	2.171591	C	-0.984057	-2.221308	2.282132	C	-1.161538	-1.887931	2.499585
C	-3.369656	-1.610790	2.258612	C	-2.384125	-2.347027	2.329409	C	-2.562831	-1.999215	2.482756
C	-4.087661	-1.234175	1.145449	C	-3.123397	-2.299402	1.171514	C	-3.234643	-2.109345	1.287820
C	-4.154735	-0.665131	-1.260566	C	-3.224098	-2.048311	-1.285172	C	-3.190473	-2.219871	-1.181744
C	-3.443065	-1.053399	-0.100307	C	-2.488481	-2.121930	-0.079249	C	-2.526479	-2.129279	0.064080
C	-2.050876	-1.259367	-0.186689	C	-1.085588	-1.991431	-0.123993	C	-1.120115	-2.040245	0.084857
C	-5.619487	-0.441106	-1.181149	C	-4.702376	-2.162111	-1.249328	C	-4.672527	-2.255953	-1.218597
C	-5.552865	-1.009244	1.254608	C	-4.602082	-2.411916	1.232178	C	-4.716718	-2.185050	1.277786
C	0.061672	-1.294419	-1.530377	C	1.030949	-1.686713	-1.432653	C	1.076200	-2.025883	-1.121388
C	0.145883	-1.765034	0.911263	C	1.132962	-1.912629	1.042543	C	1.024095	-1.750911	1.350257
O	-6.175695	-1.137470	2.288027	O	-5.227536	-2.521681	2.266528	O	-5.399297	-2.150297	2.281029
O	-6.306797	-0.117268	-2.125670	O	-5.409226	-2.072503	-2.232498	O	-5.322097	-2.254436	-2.243698
O	0.657319	-1.248813	-2.584945	O	1.629839	-1.587685	-2.482574	O	1.741709	-2.164885	-2.125426
O	0.805392	-2.048669	1.894287	O	1.809624	-1.993671	2.050011	O	1.636777	-1.604434	2.391127
N	-6.181930	-0.632037	0.077603	N	-5.250907	-2.381174	0.005949	N	-5.297215	-2.287891	0.021649
N	0.746114	-1.538249	-0.326269	N	1.716610	-1.700599	-0.208572	N	1.683538	-1.785796	0.120476
H	-4.073482	-0.186818	-3.322056	H	-3.179393	-1.790185	-3.387638	H	-3.017964	-2.314303	-3.291035
H	-1.580093	-0.572479	-3.475129	H	-0.659033	-1.582272	-3.465263	H	-0.489550	-2.229872	-3.244374
H	-1.396455	-2.083058	3.040889	H	-0.386926	-2.257481	3.185640	H	-0.618477	-1.779014	3.430877
H	-3.898969	-1.743136	3.195372	H	-2.904494	-2.476367	3.271435	H	-3.138560	-1.982848	3.400964
C	2.184415	-1.604058	-0.391424	C	3.148269	-1.535585	-0.257790	C	3.110524	-1.581317	0.123998
C	2.932639	-0.537277	0.043547	C	3.709497	-0.335223	0.103596	C	3.609366	-0.303658	0.200734
C	2.768436	-2.776897	-0.913313	C	3.922341	-2.633966	-0.687027	C	3.943675	-2.715949	0.041890
C	4.359246	-0.634640	-0.039640	C	5.134574	-0.209824	0.043084	C	5.030234	-0.131898	0.203896
C	4.125314	-2.878761	-0.997023	C	5.279710	-2.523478	-0.751140	C	5.298713	-2.562440	0.040193
H	2.120463	-3.578812	-1.247409	H	3.417687	-3.551587	-0.966461	H	3.486470	-3.696431	-0.024531
C	5.205985	0.421821	0.382603	C	5.792660	0.994903	0.398883	C	5.625338	1.152859	0.282823
C	4.954770	-1.814307	-0.563942	C	5.920950	-1.312664	-0.388452	C	5.876960	-1.270987	0.121839
H	4.585789	-3.775496	-1.399287	H	5.884691	-3.361540	-1.082352	H	5.950193	-3.428098	-0.025172
C	6.566751	0.310265	0.288529	C	7.155641	1.096343	0.328046	C	6.986402	1.295867	0.278868
H	4.755996	1.322702	0.781301	H	5.195103	1.835873	0.728777	H	4.980746	2.021078	0.346814
C	6.367045	-1.899824	-0.646378	C	7.330308	-1.176416	-0.449474	C	7.282597	-1.089392	0.121045
C	7.156626	-0.863321	-0.230343	C	7.935589	-0.000583	-0.099939	C	7.826563	0.163358	0.197623
H	7.199194	1.129138	0.614982	H	7.642894	2.025888	0.603145	H	7.425627	2.286109	0.339271
H	6.810008	-2.805849	-1.048949	H	7.920630	-2.025381	-0.781146	H	7.919936	-1.966293	0.057303
H	8.236924	-0.938411	-0.298685	H	9.015385	0.093606	-0.150904	H	8.904075	0.291353	0.195730
C	2.309063	0.701446	0.597053	C	2.879289	0.818165	0.557718	C	2.713135	0.886386	0.286770
C	1.811614	1.714211	-0.277821	C	2.239079	1.666734	-0.394180	C	2.146447	1.450209	-0.895581
C	2.298205	0.900360	1.971063	C	2.786055	1.096353	1.913407	C	2.479262	1.474316	1.520879
C	1.790852	1.554830	-1.688771	C	2.300426	1.424217	-1.792438	C	2.353095	0.884901	-2.181928
C	1.315655	2.932779	0.265022	C	1.516380	2.806034	0.054555	C	1.348642	2.623427	-0.802964
C	1.824834	2.136126	2.494782	C	2.058233	2.240023	2.344388	C	1.686401	2.653916	1.593438
C	1.263697	2.527307	-2.496378	C	1.664041	2.252329	-2.679353	C	1.774467	1.436209	-3.294954

H 2.174822 0.641608 -2.125174	H 2.842033 0.560403 -2.156857	H 2.957501 -0.008274 -2.276280
C 0.784677 3.921799 -0.598549	C 0.881591 3.649464 -0.887499	C 0.776293 3.176930 -1.973544
C 1.359447 3.117189 1.670025	C 1.452098 3.065865 1.446701	C 1.147333 3.208904 0.471909
H 1.845778 2.287127 3.570487	H 1.998437 2.448178 3.408834	H 1.522832 3.112496 2.564761
C 0.745024 3.721623 -1.951391	C 0.944807 3.380619 -2.228774	C 0.973419 2.594387 -3.197002
H 1.241956 2.373872 -3.570448	H 1.717980 2.038464 -3.741934	H 1.938124 0.976558 -4.264315
H 0.384999 4.831428 -0.162247	H 0.335518 4.514171 -0.523473	H 0.171670 4.074854 -1.879710
H 0.994847 4.052376 2.085122	H 0.901877 3.936461 1.792014	H 0.545706 4.108950 0.545184
H 0.318933 4.475644 -2.603953	H 0.452000 4.032391 -2.942876	H 0.524992 3.021574 -4.087717
N 2.801787 -0.056045 2.838266	N 3.436104 0.314587 2.856304	N 3.050439 0.971782 2.679867
H 2.662362 -1.009617 2.527320	H 3.487910 -0.665457 2.607252	H 3.160467 -0.034828 2.682049
H 2.521585 0.063247 3.800624	H 3.089114 0.430512 3.797458	H 2.609107 1.290333 3.530241
H -7.181162 -0.476687 0.140559	H -6.261954 -2.431023 0.041138	H -6.310050 -2.303195 0.004730
C -1.630404 1.733178 1.539716	C -3.278178 3.349776 0.068124	C -3.198611 3.395696 0.664281
H -0.714761 1.211679 1.268632	C -2.221981 2.309539 0.566456	C -3.567847 2.229214 1.637017
H -2.432043 1.020465 1.722866	C -3.822902 2.161431 -0.802950	C -2.411569 2.293071 -0.128941
H -1.467876 2.398079 2.381335	C -2.376506 1.599424 -0.797744	C -2.237045 1.554185 1.222159
O -2.269427 1.947189 -0.666505	C -2.806760 1.398257 1.653419	C -4.737032 1.403421 1.082887
O -2.093193 3.760551 0.507737	C -4.676799 1.249107 0.057719	C -3.369560 1.467122 -0.965494
N -2.030026 2.552090 0.366869	C -2.651603 4.446102 -0.795404	C -2.266870 4.412749 1.325804
	C -4.194413 4.016692 1.086316	C -4.315809 4.162342 -0.032066
	C -4.158576 0.873068 1.229588	C -4.475012 1.029290 -0.358302
	C -6.043302 0.874482 -0.426400	C -3.019321 1.198422 -2.394975
	H -1.234582 2.684447 0.844493	H -3.719162 2.484540 2.690894
	H -4.296324 2.403594 -1.759101	H -1.520537 2.591866 -0.683415
	H -2.276222 0.514079 -0.805108	H -2.221743 0.465672 1.178299
	H -1.732779 2.020186 -1.569318	H -1.374952 1.898453 1.792761
	H -2.901784 1.942063 2.602531	H -5.673108 1.972453 1.157190
	H -2.113439 0.569385 1.848331	H -4.884721 0.508268 1.699877
	H -2.101804 5.154817 -0.166574	H -2.828311 5.035950 2.029559
	H -3.432542 5.008010 -1.318103	H -1.831494 5.078191 0.572366
	H -1.956769 4.063377 -1.543573	H -1.445955 3.951620 1.876590
	H -4.945181 4.632019 0.579008	H -3.896281 4.878027 -0.747295
	H -3.613428 4.680418 1.735711	H -4.895780 4.734182 0.700182
	H -4.721235 3.301279 1.716899	H -5.000514 3.512552 -0.576012
	H -4.712692 0.237173 1.915793	H -5.216384 0.442750 -0.895027
	H -5.999383 0.334426 -1.378520	H -2.031061 0.731029 -2.471765
	H -6.644056 1.774318 -0.601794	H -2.961620 2.135774 -2.960384
	H -6.571097 0.252359 0.300902	H -3.752428 0.546790 -2.877043
pin-a3	pin-a4	pin-a5
C -2.896047 -1.471485 -2.441757	C -2.392498 -1.980102 -2.291840	C -2.765696 -1.796479 -2.445935
C -1.500284 -1.335370 -2.532771	C -0.998844 -1.843164 -2.415988	C -1.366503 -1.698212 -2.547599
C -0.704733 -1.636853 -1.452384	C -0.198313 -1.879832 -1.298023	C -0.580068 -1.850634 -1.429732
C -0.472355 -2.409858 0.870778	C 0.043539 -2.067446 1.142019	C -0.368290 -2.299441 0.979013
C -1.044562 -2.851802 2.040977	C -0.524400 -2.213846 2.385980	C -0.950211 -2.587703 2.191662
C -2.441450 -2.972550 2.143230	C -1.918623 -2.340321 2.516771	C -2.347460 -2.703911 2.296163
C -3.244311 -2.657056 1.071795	C -2.722635 -2.329642 1.401837	C -3.142150 -2.527728 1.187749
C -3.475129 -1.901717 -1.271349	C -2.964423 -2.152113 -1.053244	C -3.355964 -2.054039 -1.230790
C -2.676957 -2.217566 -0.147306	C -2.161316 -2.187721 0.111043	C -2.565059 -2.248330 -0.073573
C -1.276450 -2.089629 -0.244347	C -0.764391 -2.047507 -0.016050	C -1.162949 -2.138793 -0.176716
C -4.952562 -2.012698 -1.176224	C -4.435907 -2.288791 -0.930553	C -4.835403 -2.093461 -1.120624
C -4.719692 -2.774867 1.194520	C -4.194557 -2.439987 1.551176	C -4.618986 -2.608479 1.317405
C 0.764650 -1.474471 -1.560291	C 1.269032 -1.723494 -1.445886	C 0.887860 -1.666121 -1.538310
C 0.999329 -2.246635 0.792177	C 1.514550 -1.916409 1.022439	C 1.103689 -2.134885 0.893954
O -5.284761 -3.132983 2.206433	O -4.762380 -2.517209 2.620658	O -5.192607 -2.849871 2.358130
O -5.708213 -1.746914 -2.086905	O -5.199165 -2.259103 -1.875599	O -5.585921 -1.885403 -2.051786
O 1.308417 -1.124822 -2.586466	O 1.805987 -1.621581 -2.528743	O 1.437467 -1.431108 -2.593470
O 1.730224 -2.533633 1.722065	O 2.250523 -1.984929 1.987652	O 1.826433 -2.308693 1.856895
N -5.437070 -2.442054 0.053039	N -4.910880 -2.451327 0.361640	N -5.329604 -2.373103 0.146260
N 1.512890 -1.744418 -0.404049	N 2.018724 -1.684755 -0.260636	N 1.623950 -1.765953 -0.347568
H -3.539648 -1.231238 -3.280205	H -3.038786 -1.951267 -3.161502	H -3.402632 -1.649247 -3.310518
H -1.027495 -0.986689 -3.443359	H -0.531219 -1.704077 -3.383537	H -0.886306 -1.473065 -3.492498
H -0.398105 -3.088758 2.877648	H 0.123891 -2.221523 3.254127	H -0.311171 -2.706565 3.058531
H -2.910761 -3.308172 3.060900	H -2.384349 -2.442663 3.490227	H -2.823631 -2.916648 3.246373
C 2.939974 -1.558515 -0.490718	C 3.420149 -1.371096 -0.378963	C 3.046132 -1.545588 -0.438249
C 3.520367 -0.429937 0.032377	C 3.862210 -0.132262 0.022169	C 3.591440 -0.370257 0.016951
C 3.689902 -2.569029 -1.129487	C 4.284949 -2.347016 -0.914060	C 3.828102 -2.571337 -1.010994
C 4.942015 -0.288578 -0.079990	C 5.256325 0.162610 -0.106162	C 5.009326 -0.195468 -0.100110
C 5.042200 -2.442858 -1.242990	C 5.614983 -2.073796 -1.043398	C 5.176714 -2.413746 -1.127168
H 3.169260 -3.432958 -1.526584	H 3.871606 -3.300109 -1.221971	H 3.334384 -3.471941 -1.357380
C 5.620722 0.846591 0.431765	C 5.793458 1.415915 0.284024	C 5.653528 0.988551 0.340826
C 5.703440 -1.302256 -0.722701	C 6.135058 -0.817396 -0.644055	C 5.802464 -1.224610 -0.676541
H 5.627646 -3.213382 -1.734499	H 6.290716 -2.816105 -1.456201	H 5.786271 -3.196010 -1.568210
C 6.978771 0.966093 0.309562	C 7.129199 1.679092 0.146142	C 7.008032 1.139349 0.214486
H 5.043895 1.620737 0.922709	H 5.124852 2.162843 0.694228	H 5.053068 1.775323 0.780461
C 7.108051 -1.149980 -0.832144	C 7.513812 -0.514295 -0.772182	C 7.202660 -1.039327 -0.791615
C 7.733446 -0.042442 -0.328644	C 8.001490 0.704073 -0.386740	C 7.794134 0.114946 -0.356954
H 7.481285 1.842019 0.706278	H 7.523672 2.643478 0.448621	H 7.483393 2.052779 0.556282
H 7.678528 -1.931186 -1.325595	H 8.175753 -1.269824 -1.184765	H 7.797110 -1.833151 -1.233983
H 8.809471 0.064982 -0.418260	H 9.058615 0.926104 -0.489763	H 8.867029 0.247462 -0.450416
C 2.733808 0.643238 0.709007	C 2.922458 0.875787 0.594622	C 2.772216 0.716994 0.630649
C 2.168673 1.709188 -0.054185	C 2.042952 1.613501 -0.253046	C 2.130043 1.691083 -0.191756
C 2.672765 0.670510 2.095051	C 2.910647 1.092236 1.964848	C 2.743250 0.845359 2.011935
C 2.198538 1.724683 -1.474571	C 2.007634 1.427121 -1.660509	C 2.116307 1.598729 -1.609329
C 1.568127 2.811755 0.613621	C 1.159720 2.573128 0.314500	C 1.487429 2.808790 0.410603
C 2.048957 1.772344 2.745342	C 2.032902 2.067533 2.513503	C 2.079434 1.959592 2.597881

C 1.663471 2.772760 -2.177857	C 1.125590 2.125845 -2.442755	C 1.503153 2.559463 -2.370800
H 2.634242 0.886186 -2.003517	H 2.676321 0.708004 -2.116813	H 2.584103 0.747561 -2.089189
C 1.042409 3.886402 -0.141632	C 0.274551 3.292000 -0.522832	C 0.873298 3.788918 -0.404788
C 1.526792 2.807276 2.030378	C 1.189588 2.780721 1.716640	C 1.485256 2.910593 1.824395
H 2.009531 1.780522 3.830877	H 2.042705 2.230942 3.587327	H 2.067600 2.047264 3.680516
C 1.083571 3.872818 -1.510230	C 0.244207 3.069893 -1.873644	C 0.878291 3.672666 -1.769013
H 1.691203 2.756736 -3.262624	H 1.110463 1.953530 -3.514114	H 1.499661 2.461506 -3.451551
H 0.603187 4.725851 0.389031	H -0.386354 4.023553 -0.068676	H 0.389524 4.634442 0.075574
H 1.062164 3.644577 2.542874	H 0.521079 3.517395 2.152443	H 0.990343 3.759213 2.287606
H 0.674285 4.700243 -2.080212	H -0.446874 3.618641 -2.504791	H 0.401382 4.427031 -2.385920
N 3.272436 -0.313858 2.863204	N 3.768318 0.405048 2.810531	N 3.408264 -0.050490 2.833274
H 3.267775 -1.234754 2.441936	H 3.968867 -0.538335 2.503053	H 3.426781 -0.999226 2.480005
H 2.956832 -0.345303 3.821487	H 3.483867 0.414239 3.779452	H 3.115418 -0.024731 3.799072
H -6.444902 -2.511274 0.129411	H -5.917583 -2.507696 0.456573	H -6.339018 -2.401436 0.229190
C -3.738942 3.438745 0.353419	C -3.375850 2.508271 0.675390	C -2.432686 1.888028 0.580080
C -3.870222 2.829768 -1.080673	C -3.316271 3.103865 -0.769491	C -2.529437 3.414528 0.902862
C -3.577584 1.946444 0.814778	C -4.923028 2.438368 0.425160	C -3.996647 1.915607 0.667432
C -4.442272 1.557020 -0.413019	C -4.746353 3.645696 -0.534914	C -3.847551 3.103848 1.654272
C -2.494939 2.485388 -1.670885	C -3.372693 1.987312 -1.820696	C -2.867761 4.223967 -0.355554
C -2.160620 1.473178 0.545425	C -5.276125 1.246807 -0.445551	C -4.580629 2.471532 -0.618527
C -5.052236 4.054990 0.838947	C -3.017130 3.538023 1.748285	C -1.809462 1.091762 1.727552
C -2.616114 4.428188 0.633088	C -2.610674 1.226623 0.974915	C -1.794021 1.425546 -0.721867
C -1.667833 1.715039 -0.670108	C -4.527588 1.055304 -1.534821	C -4.038519 3.599849 -1.079561
C -1.397087 0.816024 1.651123	C -6.462586 0.414273 -0.069003	C -5.709878 1.739788 -1.274586
H -4.496410 3.369801 -1.798042	H -2.521011 3.820880 -0.988135	H -1.701506 3.860455 1.463073
H -3.928912 1.681438 1.816799	H -5.583760 2.538988 1.291465	H -4.503961 1.004741 1.003364
H -4.207540 0.608492 -0.894837	H -5.416306 3.682511 -1.396926	H -4.631878 3.863620 1.621823
H -5.515256 1.610538 -0.230444	H -4.758063 4.611534 -0.029722	H -3.688766 2.794711 2.687238
H -1.955754 3.396525 -1.961344	H -2.423207 1.435680 -1.840352	H -1.994937 4.272641 -1.019309
H -2.624617 1.903206 -2.593057	H -3.486512 2.427481 -2.820005	H -3.100317 5.260335 -0.077403
H -5.239508 5.001671 0.321567	H -1.935354 3.704017 1.765301	H -0.727685 1.249049 1.757450
H -4.996974 4.269178 1.911260	H -3.307923 3.170322 2.737701	H -1.988360 0.023703 1.582555
H -5.920105 3.414063 0.675384	H -3.498004 4.506515 1.601341	H -2.209894 1.357896 2.707594
H -2.579007 4.670021 1.701081	H -2.854770 0.861317 1.978555	H -2.098878 0.397540 -0.923040
H -2.793670 5.363950 0.091906	H -1.531686 1.407906 0.939817	H -0.704201 1.446311 -0.655248
H -1.640138 4.044968 0.341458	H -2.842640 0.433440 0.267719	H -2.090728 2.023683 -1.581745
H -0.655919 1.429563 -0.942567	H -4.737644 0.243291 -2.225227	H -4.405958 4.073107 -1.986910
H -1.918310 -0.074722 2.019246	H -7.368162 1.030717 -0.029390	H -6.549072 1.610911 -0.581014
H -1.294518 1.496515 2.503927	H -6.336119 -0.013501 0.933179	H -5.407407 0.733607 -1.587527
H -0.390457 0.532047 1.336384	H -6.627722 -0.394980 -0.784055	H -6.075588 2.268930 -2.157846
pin-a6	pin-a7	pin-a8
C -3.059074 -1.535943 -2.574687	C 2.228881 1.290688 -2.380502	C -2.460295 -1.864801 -2.499386
C -1.653082 -1.512642 -2.550709	C 0.850704 1.092158 -2.577039	C -1.060184 -1.733170 -2.529618
C -0.978342 -1.703074 -1.367186	C -0.049050 1.501625 -1.621235	C -0.333203 -1.806524 -1.364569
C -1.008099 -2.110449 1.057453	C -0.512466 2.518546 0.570189	C -0.249391 -2.076496 1.078466
C -1.711573 -2.330256 2.219134	C -0.063591 3.131898 1.716240	C -0.894270 -2.285819 2.275505
C -3.116911 -2.370304 2.198197	C 1.310128 3.366225 1.901716	C -2.291594 -2.436659 2.309298
C -3.797496 -2.186760 1.017684	C 2.215038 2.974933 0.943338	C -3.023722 -2.373096 1.146726
C -3.768905 -1.756966 -1.417561	C 2.685249 1.906650 -1.238211	C -3.110052 -2.080360 -1.307215
C -3.096137 -1.961797 -0.189744	C 1.777208 2.344161 -0.245228	C -2.382137 -2.166894 -0.096408
C -1.686723 -1.927165 -0.167032	C 0.397727 2.123632 -0.434995	C -0.980552 -2.019126 -0.127661
C -5.253276 -1.766350 -1.451444	C 4.140941 2.096492 -1.030889	C -4.591377 -2.173644 -1.278740
C -5.282367 -2.198197 1.012895	C 3.665946 3.193419 1.165905	C -4.502683 -2.496205 1.198112
C 0.505976 -1.701624 -1.361938	C -1.498176 1.257524 -1.828613	C 1.143474 -1.668138 -1.413426
C 0.473866 -2.062664 1.096983	C -1.958382 2.222094 0.417578	C 1.223407 -1.897928 1.061479
O -5.957270 -2.377133 2.004356	O 4.125404 3.739692 2.146129	O -5.128629 -2.666287 2.222546
O -5.905555 -1.597132 -2.459665	O 4.989785 1.741115 -1.824623	O -5.292236 -2.059890 -2.263849
O 1.158513 -1.597740 -2.378015	O -1.942740 0.806318 -2.862001	O 1.751790 -1.560520 -2.456632
O 1.093678 -2.220094 2.132872	O -2.773971 2.532218 1.264210	O 1.889546 -1.955155 2.077798
N -5.872433 -1.982850 -0.226277	N 4.497383 2.710742 0.161047	N -5.149035 -2.389050 -0.026872
N 1.130662 -1.847954 -0.113804	N -2.339448 1.543433 -0.742642	N 1.815042 -1.668606 -0.181654
H -3.607114 -1.378537 -3.496550	H 2.953028 0.960220 -3.116322	H -3.050806 -1.791145 -3.405392
H -1.078023 -1.344491 -3.453716	H 0.473717 0.607568 -3.469764	H -0.531136 -1.566237 -3.460519
H -1.160025 -2.465992 3.141932	H -0.788510 3.415258 2.470082	H -0.303697 -2.323353 3.183207
H -3.687719 -2.535658 3.104727	H 1.682243 3.843246 2.801152	H -2.817347 -2.593258 3.244256
C 2.572905 -1.900287 -0.103538	C -3.672925 0.998511 -0.777930	C 3.243221 -1.470349 -0.211614
C 3.314356 -0.778756 0.169467	C -3.932028 -0.126780 -0.028097	C 3.769234 -0.243008 0.108928
C 3.166744 -3.150290 -0.384342	C -4.652735 1.620247 -1.574895	C 4.050331 -2.568109 -0.576875
C 4.743203 -0.899468 0.167815	C -5.253827 -0.667085 -0.052270	C 5.192272 -0.087902 0.072168
C 4.523664 -3.274306 -0.390872	C -5.916678 1.105534 -1.611190	C 5.405991 -2.429336 -0.618240
H 2.523485 -3.997231 -0.595553	H -4.380903 2.496269 -2.151566	H 3.572050 -3.508599 -0.825342
C 5.583318 0.210032 0.440568	C -5.602895 -1.817350 0.700556	C 5.815297 1.145785 0.389700
C 5.347369 -2.154361 -0.115143	C -6.250988 -0.043800 -0.852531	C 6.012184 -1.189636 -0.294934
H 4.988445 -4.230845 -0.607806	H -6.681959 1.572247 -2.223236	H 6.036374 -3.266615 -0.900429
C 6.945776 0.077748 0.430423	C -6.874409 -2.320758 0.658364	C 7.176966 1.275597 0.343294
H 5.126779 1.167743 0.658057	H -4.841760 -2.288150 1.311257	H 5.191912 1.985847 0.670592
C 6.760399 -2.259492 -0.117405	C -7.558881 -0.590696 -0.873651	C 7.419122 -1.023459 -0.332392
C 7.543663 -1.169915 0.148635	C -7.864981 -1.701674 -0.136899	C 7.990230 0.179915 -0.021046
H 7.572878 0.937745 0.640881	H -7.126840 -3.201368 1.239667	H 7.637328 2.226924 0.588616
H 7.209485 -3.223849 -0.335531	H -8.313784 -0.109370 -1.488176	H 8.035388 -1.871811 -0.614773
H 8.624946 -1.260615 0.143862	H -8.869285 -2.111827 -0.160640	H 9.068517 0.296845 -0.053439
C 2.704817 0.551465 0.461354	C -2.853569 -0.747867 0.796183	C 2.907713 0.914348 0.489173
C 2.482086 1.478791 -0.601019	C -1.838372 -1.536732 0.174961	C 2.324855 1.737749 -0.520372
C 2.507018 0.938859 1.778304	C -2.810240 -0.509489 2.161086	C 2.746868 1.234383 1.828958
C 2.667155 1.126586 -1.964677	C -1.849571 -1.829819 -1.214017	C 2.467232 1.456299 -1.905290
C 2.085491 2.810154 -0.301260	C -0.764245 -2.045371 0.955580	C 1.584644 2.892770 -0.145481

C 2.082533 2.269321 2.057360	C -1.745754 -1.053539 2.933376	C 2.002708 2.393602 2.185598
C 2.465024 2.045133 -2.960842	C -0.834736 -2.547593 -1.790714	C 1.893882 2.265934 -2.850179
H 2.946914 0.110916 -2.214001	H -2.662367 -1.459738 -1.826309	H 3.020147 0.577593 -2.212577
C 1.902997 3.739527 -1.352312	C 0.268742 -2.784931 0.331365	C 1.013782 3.714390 -1.146603
C 1.891950 3.172281 1.055510	C -0.755402 -1.783918 2.349624	C 1.447901 3.193659 1.232728
H 1.929615 2.559411 3.093056	H -1.728149 -0.860908 4.002269	H 1.889928 2.635505 3.238629
C 2.084082 3.370632 -2.658088	C 0.242892 -3.027004 -1.016595	C 1.157717 3.409977 -2.473836
H 2.603229 1.749642 -3.995945	H -0.862428 -2.748539 -2.856878	H 2.010240 2.024061 -3.901736
H 1.613631 4.754596 -1.098865	H 1.093101 -3.141867 0.941935	H 0.453281 4.592004 -0.838731
H 1.576032 4.184645 1.286720	H 0.062901 -2.172411 2.948650	H 0.883773 4.075396 1.522531
H 1.938195 4.089338 -3.457640	H 1.042487 -3.587468 -1.487214	H 0.713372 4.044039 -3.233765
N 2.789179 0.088463 2.834276	N -3.797632 0.226886 2.799024	N 3.349238 0.484849 2.827753
H 2.649035 -0.895896 2.642275	H -4.208228 0.946954 2.218048	H 3.423943 -0.502267 2.614929
H 2.376171 0.359508 3.714208	H -3.515210 0.606204 3.691344	H 2.958250 0.630243 3.747164
H -6.885553 -1.985792 -0.238673	H 5.490962 2.842332 0.310128	H -6.160550 -2.439063 0.002789
C -3.447703 2.306194 0.469146	C 5.316514 -1.801770 -0.682416	C -2.884780 2.154446 0.693957
C -2.345021 1.642738 -0.417496	C 5.211658 -3.304275 -0.264996	C -4.037653 2.327181 1.735711
C -2.242306 2.775339 1.358835	C 3.781736 -1.751123 -0.343567	C -3.882976 1.192519 -0.034554
C -1.481151 1.516865 0.859832	C 3.733217 -3.257262 -0.712748	C -4.501281 0.899129 1.359932
C -1.687021 2.670696 -1.347640	C 5.241429 -3.467425 1.261111	C -5.090311 3.326397 1.235662
C -1.575417 3.984049 0.731830	C 3.586537 -1.646039 1.156831	C -4.913146 1.980827 -0.819213
C -4.316931 1.273730 1.188880	C 5.580206 -1.634651 -2.180617	C -1.685438 1.395541 1.266818
C -4.371721 3.340796 -0.160431	C 6.284437 -0.896835 0.070450	C -2.364663 3.380234 -0.043479
C -1.311305 3.910029 -0.572677	C 4.270564 -2.508947 1.910703	C -5.502033 2.992358 -0.179845
C -1.251861 5.158695 1.600041	C 2.707113 -0.562912 1.697107	C -5.199244 1.570968 -2.230063
H -2.618624 0.734073 -0.958616	H 5.887296 -4.008812 -0.760527	H -3.751791 2.524883 2.773825
H -2.418364 2.897677 2.431882	H 3.152318 -1.053077 -0.901875	H -3.450763 0.377004 -0.616831
H -0.399955 1.610612 0.744730	H 3.029399 -3.877237 -0.153346	H -5.574583 0.696912 1.384184
H -1.705015 0.621725 1.439857	H 3.601120 -3.437321 -1.779481	H -3.979486 0.128448 1.924856
H -2.362773 2.929739 -2.173320	H 6.253147 -3.283553 1.646241	H -4.698503 4.351099 1.282996
H -0.790230 2.235431 -1.807546	H 4.999101 -4.504479 1.527131	H -5.962857 3.304748 1.901285
H -4.971305 0.761922 0.474281	H 6.618936 -1.894730 -2.410068	H -1.132876 2.023770 1.971301
H -4.959715 1.765549 1.925641	H 5.424686 -0.592283 -2.472278	H -0.988633 1.127965 0.465688
H -3.739882 0.513263 1.715172	H 4.940910 -2.261687 -2.805082	H -1.961160 0.476881 1.787491
H -5.005990 3.801702 0.604300	H 6.219594 0.125292 -0.314253	H -1.662947 3.081660 -0.827787
H -5.034253 2.858664 -0.887416	H 7.312746 -1.239395 -0.088012	H -1.818550 4.031397 0.648212
H -3.831357 4.138188 -0.668892	H 6.096749 -0.876347 1.144268	H -3.159275 3.964194 -0.506942
H -0.830513 4.727937 -1.101136	H 4.188111 -2.500842 2.995000	H -6.263118 3.600690 -0.662269
H -0.589863 4.863115 2.422929	H 1.719194 -0.571623 1.226655	H -5.512966 0.522218 -2.285516
H -2.157845 5.569438 2.060573	H 3.151218 0.415753 1.485443	H -4.294562 1.656659 -2.844220
H -0.763326 5.957932 1.036565	H 2.578727 -0.642627 2.779525	H -5.977659 2.189739 -2.682797
pin-a9	pin-a10	pin-a11
C -2.557327 -2.611855 -2.329947	C 1.309096 -4.462966 0.852597	C -1.094933 4.580558 0.396498
C -1.164517 -2.419897 -2.357437	C -0.020203 -4.080292 0.604469	C 0.214388 4.156483 0.108752
C -0.466682 -2.252549 -1.184265	C -0.287650 -2.971823 -0.165000	C 0.427187 2.965924 -0.545912
C -0.433022 -2.105437 1.269862	C 0.502788 -1.060948 -1.496639	C -0.458108 0.930531 -1.606221
C -1.099593 -2.132223 2.473096	C 1.537719 -0.343307 -2.046387	C -1.530607 0.179033 -2.024231
C -2.491219 -2.330228 2.507087	C 2.869257 -0.739625 -1.827311	C -2.841622 0.610556 -1.759534
C -3.194916 -2.499199 1.338471	C 3.148227 -1.835572 -1.046623	C -3.061604 1.777884 -1.068924
C -3.230028 -2.636894 -1.130623	C 2.350267 -3.737224 0.321700	C -2.171467 3.811381 0.020621
C -2.530999 -2.480632 0.089587	C 2.098935 -2.596623 -0.476299	C -1.975792 2.587914 -0.662029
C -1.135629 -2.283160 0.058149	C 0.764326 -2.208066 -0.713555	C -0.661767 2.160024 -0.940917
C -4.704118 -2.814921 -1.114128	C 3.750276 -4.151848 0.585919	C -3.550176 4.260630 0.336009
C -4.668286 -2.676150 1.384052	C 4.556799 -2.220594 -0.785326	C -4.445412 2.175436 -0.715673
C 0.997763 -2.021316 -1.232256	C -1.695663 -2.573522 -0.406879	C 1.814655 2.511393 -0.806858
C 1.035436 -1.895710 1.251032	C -0.894528 -0.606691 -1.703424	C 0.919243 0.415018 -1.804273
O -5.312226 -2.705896 2.411244	O 5.518199 -1.611829 -1.211040	O -5.424206 1.494957 -0.952785
O -5.377328 -2.956244 -2.112973	O 4.055879 -5.113458 1.259085	O -3.809923 5.296610 0.910486
O 1.620158 -2.010176 -2.273124	O -2.635083 -3.236522 -0.021509	O 2.783735 3.196168 -0.559109
O 1.684199 -1.804365 2.276006	O -1.161822 0.378448 -2.364116	O 1.137665 -0.656984 -2.335860
N -5.287598 -2.804993 0.146874	N 4.725721 -3.350521 0.002613	N -4.563538 3.395890 -0.067234
N 1.643563 -1.815946 -0.003140	N -1.896206 -1.364037 -1.089969	N 1.959527 1.212320 -1.320616
H -3.125407 -2.733442 -3.245095	H 1.539405 -5.330501 1.460352	H -1.280673 5.512331 0.918353
H -0.619882 -2.387154 -3.293688	H -0.852988 -4.640680 1.012547	H 1.074165 4.746820 0.402479
H -0.529939 -1.995493 3.384649	H 1.312127 0.539001 -2.632701	H -1.342649 -0.757449 -2.535541
H -3.033823 -2.345040 3.445297	H 3.687002 -0.173957 -2.252138	H -3.697187 0.020048 -2.063932
C 3.067699 -1.592437 -0.042675	C -3.239237 -0.843093 -1.121059	C 3.267796 0.615368 -1.229276
C 3.574831 -0.334182 0.168197	C -3.553069 0.224871 -0.312667	C 3.488825 -0.313196 -0.236734
C 3.892923 -2.706484 -0.307182	C -4.184289 -1.455693 -1.967627	C 4.271568 1.012102 -2.133297
C 4.996761 -0.162379 0.115957	C -4.889940 0.731296 -0.349409	C 4.786142 -0.903548 -0.139453
C 5.245993 -2.552921 -0.359924	C -5.463369 -0.982515 -2.005833	C 5.516217 0.458958 -2.042697
H 3.429129 -3.672281 -0.471619	H -3.876057 -2.301551 -2.570521	H 4.036386 1.758416 -2.882563
C 5.602748 1.103719 0.318209	C -5.293918 1.830858 0.450179	C 5.091404 -1.874175 0.848671
C 5.833367 -1.280127 -0.150667	C -5.849129 0.119506 -1.202635	C 5.805470 -0.510299 -1.050154
H 5.888739 -3.403156 -0.564867	H -6.201740 -1.446865 -2.651786	H 6.300780 0.756988 -2.730973
C 6.962431 1.248589 0.257742	C -6.580260 2.295042 0.403842	C 6.342620 -2.422011 0.927568
H 4.968730 1.957846 0.521497	H -4.562778 2.297193 1.099283	H 4.312090 -2.173968 1.538974
C 7.237673 -1.098219 -0.204854	C -7.173181 0.625276 -1.228383	C 7.091218 -1.097878 -0.941628
C 7.791764 0.136473 -0.005622	C -7.532187 1.687486 -0.445067	C 7.355257 -2.031055 0.022815
H 7.408474 2.225238 0.413795	H -6.873480 3.136741 1.022478	H 6.561210 -3.163237 1.689107
H 7.865710 -1.960117 -0.409605	H -7.898148 0.150517 -1.882793	H 7.863690 -0.790790 -1.640347
H 8.868236 0.265468 -0.050122	H -8.548624 2.066380 -0.472164	H 8.343012 -2.474113 0.096914
C 2.706042 0.845671 0.452593	C -2.519809 0.853701 0.561177	C 2.384606 -0.707362 0.687165
C 2.110449 1.578371 -0.617641	C -2.095592 0.212783 1.764613	C 1.966292 0.167346 1.735732
C 2.578501 1.294074 1.759365	C -1.951727 2.059690 0.180635	C 1.723241 -1.908110 0.480313
C 2.202643 1.156470 -1.970830	C -2.652938 -1.015083 2.208856	C 2.624473 1.395187 2.008335

C	1.411768	2.785978	-0.340165	C	-1.073510	0.805690	2.556436	C	0.846948	-0.182102	2.540658
C	1.860387	2.494248	2.020302	C	-0.967014	2.662560	1.010040	C	0.626202	-2.258584	1.315382
C	1.635061	1.893142	-2.977474	C	-2.192001	-1.629157	3.344066	C	2.170092	2.236800	2.990215
H	2.716118	0.231663	-2.203651	H	-3.441400	-1.479001	1.629479	H	3.487280	1.675272	1.416630
C	0.848340	3.530294	-1.403397	C	-0.622205	0.148903	3.725659	C	0.404105	0.706410	3.548472
C	1.309900	3.217989	1.006382	C	-0.534699	2.051235	2.147573	C	0.199089	-1.420449	2.300052
H	1.773457	2.835600	3.047885	H	-0.550059	3.617793	0.707245	H	0.103761	-3.191267	1.133952
C	0.954549	3.098254	-2.698768	C	-1.161161	-1.048680	4.113370	C	1.043384	1.897589	3.768770
H	1.714156	1.545939	-4.002625	H	-2.629382	-2.572487	3.655040	H	2.687059	3.173774	3.171170
H	0.320934	4.450531	-1.169898	H	0.164279	0.616735	4.311319	H	-0.458043	0.423309	4.145905
H	0.779959	4.141315	1.221976	H	0.237891	2.514051	2.754418	H	-0.655861	-1.695378	2.911245
H	0.518089	3.675476	-3.507252	H	-0.806127	-1.547029	5.009222	H	0.693772	2.574312	4.541287
N	3.196312	0.635892	2.810696	N	-2.344348	2.713010	-0.978804	N	2.120854	-2.790115	-0.513152
H	3.246030	-0.368646	2.693886	H	-2.614074	2.083341	-1.724214	H	2.501427	-2.337068	-1.334198
H	2.836619	0.880626	3.721741	H	-1.674522	3.392743	-1.310163	H	1.417705	-3.468604	-0.766093
H	-6.293627	-2.923248	0.167880	H	5.684744	-3.620517	0.186477	H	-5.505688	3.679103	0.174946
C	-2.922447	1.805347	-0.316747	C	3.907068	3.870348	0.764728	C	-4.814895	-3.090322	-0.241292
C	-3.821194	2.562611	-1.345748	C	2.459605	3.320525	0.560960	C	-4.388663	-2.726188	1.217913
C	-2.438700	3.237612	0.099587	C	4.359721	2.444206	0.290232	C	-3.374778	-2.606544	-0.626872
C	-2.750680	3.680123	-1.355403	C	3.018768	1.910872	0.864989	C	-3.444201	-1.663430	0.604710
C	-5.089808	3.106178	-0.673519	C	2.042201	3.358615	-0.915372	C	-3.540205	-3.844692	1.838470
C	-3.464636	3.902855	0.995237	C	4.274126	2.357960	-1.221665	C	-2.345922	-3.672342	-0.307059
C	-1.813168	1.002469	-0.997158	C	4.222158	4.112331	2.242231	C	-5.894559	-2.149995	-0.783340
C	-3.594127	0.910596	0.716549	C	4.347989	5.098733	-0.020944	C	-5.229086	-4.522930	-0.551623
C	-4.735479	3.849587	0.593681	C	3.137164	2.778798	-1.781068	C	-2.433282	-4.249241	0.892904
C	-2.992354	4.573838	2.246591	C	5.455305	1.831724	-1.975742	C	-1.292473	-3.973095	-1.326485
H	-4.049290	2.048253	-2.285037	H	1.682925	3.727180	1.213942	H	-5.175423	-2.389441	1.900081
H	-1.418044	3.331914	0.476380	H	5.300593	2.047467	0.681973	H	-3.235503	-2.183003	-1.625403
H	-3.102414	4.706518	-1.485742	H	2.557929	1.071544	0.341121	H	-2.498434	-1.465633	1.114362
H	-1.934274	3.484223	-2.048799	H	3.059840	1.684877	1.930277	H	-3.951160	-0.720962	0.403450
H	-5.784932	2.286553	-0.447477	H	1.826011	4.388908	-1.229002	H	-4.166898	-4.712221	2.082882
H	-5.620526	3.769745	-1.368396	H	1.108670	2.793443	-1.044192	H	-3.115194	-3.501354	2.790813
H	-2.233464	0.107866	-1.465791	H	3.698100	5.005117	2.599062	H	-6.854988	-2.364278	-0.303145
H	-1.072735	0.679253	-0.258566	H	5.294963	4.281558	2.379018	H	-6.024948	-2.309691	-1.859053
H	-1.273807	1.558107	-1.764050	H	3.935035	3.281605	2.888992	H	-5.682122	-1.089832	-0.634637
H	-2.849298	0.484375	1.396496	H	5.412699	5.294865	0.144117	H	-5.386619	-4.647618	-1.628168
H	-4.095913	0.075280	0.215741	H	3.796805	5.981305	0.320851	H	-6.176383	-4.756539	-0.054384
H	-4.335146	1.436414	1.317840	H	4.191287	4.994517	-1.094090	H	-4.488851	-5.258238	-0.237333
H	-5.532095	4.313748	1.170085	H	2.995321	2.754065	-2.859529	H	-1.728499	-5.016399	1.205943
H	-2.259471	5.357039	2.018650	H	5.708321	0.813827	-1.658671	H	-0.690927	-3.079667	-1.539104
H	-2.487114	3.855807	2.903356	H	6.337860	2.452593	-1.782969	H	-1.741922	-4.281523	-2.277042
H	-3.816162	5.027158	2.803101	H	5.276923	1.824836	-3.054671	H	-0.625650	-4.774635	-0.994672
